# The impact for causal associations between common diseases and inflammatory bowel disease: a disease-wide bidirectional Mendelian randomization study

**DOI:** 10.1007/s00011-026-02304-8

**Published:** 2026-09-05

**Authors:** Yuxuan Sun, Zixin Liang, Zehua Ou, Lei Gao, Yong-Fei Wang, Jinqiu Yuan, Guangjun Yu, Rui Sun

**Affiliations:** 1 Clinical Big Data Research Center, The Seventh Affiliated Hospital, Sun Yat-sen University, Shenzhen, 518107 People’s Republic of China; 2 Institute for Human Genetics, Clemson University, Self Regional Hall, 114 Gregor Mendel Circle, Greenwood, SC 29646 USA; 3 Scientific Research Center, The Seventh Affiliated Hospital, Sun Yat-sen University, Shenzhen, 518107 People’s Republic of China; 4 School of Public Health, Sun Yat-sen University, Shenzhen, 518107 People’s Republic of China; 5 School of Medicine, The Chinese University of Hong Kong, Shenzhen, 518172 Guangdong People’s Republic of China; 6 Warshel Institute for Computational Biology, School of Medicine, The Chinese University of Hong Kong, Shenzhen, 518172 Guangdong People’s Republic of China; 7 School of Medicine, The Second Affiliated Hospital, The Chinese University of Hong Kong, Shenzhen, 518172 Guangdong People’s Republic of China

**Keywords:** Mendelian randomization, GWAS summary data, Inflammatory bowel disease

## Abstract

**Objectives:**

Observational studies on associations between various diseases and inflammatory bowel disease (IBD) are often limited by confounding and reverse causation. We aimed to assess potential causal relationships between a wide range of diseases and IBD, including Crohn’s disease (CD) and ulcerative colitis (UC).

**Methods:**

We performed a comprehensive bidirectional Mendelian randomization (MR) analysis of 104 common diseases and IBD traits using the generalized summary-data-based MR (GSMR) approach. Genome-wide association study (GWAS) summary statistics for diseases were obtained from the MRC Integrative Epidemiology Unit, and IBD data from the International IBD Genetics Consortium. Summary-data-based MR (SMR) integrating GWAS and expression quantitative trait locus data was applied to identify pleiotropic genes associated with IBD.

**Results:**

MR analyses identified 38, 34, and 52 exposures significantly associated with IBD, UC, and CD, respectively. Childhood- and adult-onset asthma showed distinct causal effects on UC and CD. Reverse MR indicated associations between IBD traits and 15 diseases, including multiple sclerosis. SMR identified *RGS14* and *CARD9* as pleiotropic genes linked to IBD, suggesting shared genetic mechanisms with asthma and multiple sclerosis.

**Conclusions:**

These findings provide evidence for causal links and shared immune-related genetic mechanisms underlying IBD, highlighting potential targets for future research.

**Supplementary Information:**

The online version contains supplementary material available at https://doi.org/10.1007/s00011-026-02304-8.

## Introduction

Inflammatory bowel disease [IBD], including its major subtypes ulcerative colitis [UC] and Crohn’s disease [CD], is an inflammatory condition that affects the gastrointestinal tract and is characterized by chronic and recurring symptoms [1]. IBD, considered as a prototypical complex disease, characterized by a multifactorial etiology involving intricate interactions between genetic predispositions, environmental exposures, gut microbiota, and other diseases [2–4]. The clinical manifestations of IBD vary depending on disease subtype, affected anatomical regions, and disease severity [5]. CD is associated with full-thickness inflammation which can involve in any part of the gastrointestinal tracts with primary occurrence in the ileum [6]. In contrast, UC is usually localized to the large intestine or colon as the inflammation associated with UC is limited to the mucosal layer of colonic tissue [6]. IBD is a significant public health issue with an estimated of more than 6.8 million people living with IBD worldwide [7]. Along with the high disease rate, IBD also poses a large economic burden due to its unpredictable pattern of relapses and remissions, complications, hospitalizations, surgeries, and the need for expensive and necessary substantial therapies [8, 9]. Annual mean direct health care costs for patients with IBD are about $23,000, over 3-fold higher than patients without IBD [10]. Furthermore, IBD is accompanied by various complications and extra-intestinal manifestations such as anemia, mouth sores, and even cancer, which heavily increases the cost [11, 12]. Given the escalating individual and societal costs of IBD, there is a critical need for research aimed at elucidating the complex interplay of risk factors and improving preventive and therapeutic strategies.

The complex bidirectional communication between gastrointestinal tract and other organs, mediated by the gut microbiota, has a fundamental role in the pathogenesis of IBD [13–16]. It has been hypothesized that IBD is related with a variety of diseases in the context of the interaction between gastrointestinal tract and other organs via multiple routes such as the gut–lung [14, 17], gut–skin [18, 19] and gut–brain axes [16, 20]. Numerous observational studies have identified associations between IBD and a range of comorbid conditions, including neurodegenerative diseases [21–25], chronic lung disease [26–28], skin diseases [29–31], and other diseases [27, 32–34]. However, these findings are often inconsistent and may be confounded by methodological limitations such as reverse causation and residual confounding [35], . For example, a cross-sectional study finds no significant association between IBD and diabetes mellitus while another study on Denmark population indicates that IBD is associated with a higher risk of diabetes [36, 37]. Similarly, two studies have suggested that people with IBD are more likely to develop bipolar disorders [24, 38] while a meta-analyzed study finds no consistent associations between bipolar disorders and IBD [39]. These discrepancies underscore the limitations of traditional observational approaches and highlight the need for robust methods to infer bidirectional causality in complex disease networks [40].

Mendelian randomization [MR] offers a potential solution to overcome such limitations. MR is an alternative strategy that mimics the design of randomized clinical trials for causal inference using genetic variants as instruments to test the causality between an exposure and an outcome [40]. This use of genetic data bypasses typical sources of confounding and allows for triangulation of findings [41]. 

In this study, we used a bidirectional two-sample disease-wide MR study design combined with summary data-based MR [SMR] analysis to evaluate potential causal relationships between 104 diseases and IBD, including CD and UC. SMR analysis integrates genetic summary data and gene expression data to further identify pleiotropy genes whose expression levels are associated with IBD., which provides insights to the biological pathway from genetic determinants to transcriptomic features [42, 43]. This study assessed directional effects and causal relationships between diseases and IBD using genetic data, thereby contributing to an enhanced comprehensive understanding of the relationship between IBD and other diseases and facilitating early prevention.

## Methods

### Study design

**Fig. 1:**
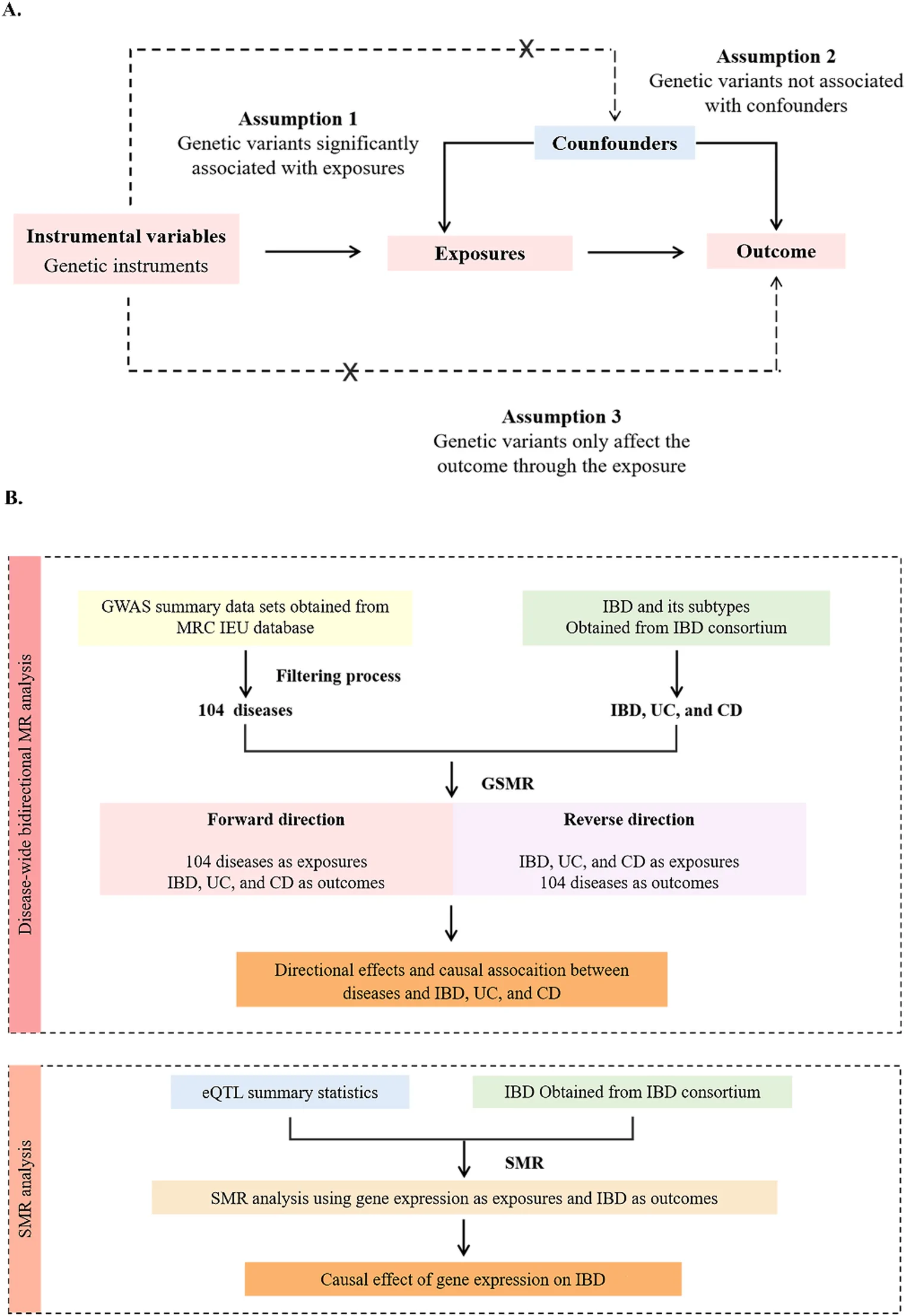
Overview of study design. Schematic illustration of the bidirectional disease-wide Mendelian randomization (MR) workflow evaluating causal relationships between 104 common diseases and inflammatory bowel disease (IBD) traits, including ulcerative colitis (UC) and Crohn’s disease (CD). The figure also summarizes the complementary SMR analysis integrating GWAS and eQTL data to identify pleiotropic genes associated with IBD

We conducted a disease-wide, bidirectional two-sample MR study to assess the causal relationships and directional effects between 104 common diseases [Supplementary Table 1] and IBD, including its subtypes - UC, and CD. In forward direction, IBD traits (i.e., IBD, UC, and CD) were treated as outcomes, while a total of 104 selected diseases were analyzed as exposures [Figure 1]. Conversely, in the reverse direction, the IBD, UC, and CD were treated as exposures, and the selected diseases as outcomes. This bidirectional approach enabled us to assess both the risk factors for IBD and its downstream phenotypic consequences.

To further explore the biological mechanisms underlying the observed associations, we performed a summary-data-based Mendelian randomization (SMR) analysis using both genome-wide association study (GWAS) and expression quantitative trait locus (eQTL) summary data. This integrative method allowed us to identify pleiotropic genes, those whose expression levels are causally associated with IBD, across five distinct tissues or cell lines: lung, thyroid, skeletal, muscle, whole blood, and lymphoblastoid cell lines (LCLs).

### Data source

***GWAS datasets for IBD***,*** UC and CD*** Summary statistics of meta-analyzed data of IBD [*N* = 31,665 cases, 33,997 controls], UC [*N* = 6968 cases, 20,464 controls], and CD [*N* = 5956 cases, 14,927 controls] were obtained from the International IBD Genetics Consortium [44]. Diagnosis of IBD was based on accepted radiological, endoscopic and histopathological evaluation. All included cases fulfilled clinical criteria for IBD. The corresponding effect estimates of SNP on IBD were adjusted for age, sex, and up to 20 genetic principal components and were imputed using HapMap3 reference set to reduce genome-wide inflation. All participants were European ancestry. Informed consent was obtained from all participants in the corresponding studies.

***GWAS datasets for common diseases*** GWAS summary data of disease traits were extracted from the MRC Integrative Epidemiology Unit [IEU] GWAS database supported by the MRC Integrative Epidemiology Unit [IEU] at the University of Bristol [https://gwas.mrcieu.ac.uk/]. The MRC IEU GWAS database, which contains up to 245,633,827,575 genetic associations from 42,346 GWAS summary datasets [as at 8 March 2023], collected publicly available GWAS data from UK Biobank, published articles, and FinnGen Biobank, and made complete GWAS summary datasets available for analyzing and downloading [45, 46]. The flowchart shows the number of disease datasets removed at each step of the quality control pipeline [Supplementary Fig. 1], and finally a total number of 104 out of 42,346 datasets passed the filtering. All the participants remained were European ancestry. The database was accessed via the TwoSampleMR package v0.5.6 [46] in R.

***Expression data*** Five eQTL datasets were used in the analysis. The dataset of the lymphoblastoid cell lines [LCL] was extracted from the study conducted by GEUVADIS consortium, which determines regulatory variation in the human genome from LCL of 462 individuals from European population of the 1000 Genomes Project [47]. We extracted summarized eQTL data of lung, thyroid, muscle skeletal, and whole blood from the V7 release of Genotype-Tissue Expression [GTEx] project, which aim to characterize variation in gene expression levels across 44 different tissues of human body with sample size ranged from 80 to 491 [48, 49].

### Genetic instrument selection

Genetic instruments were selected based on genome-wide significance (*p* < 5 × 10^− 8^) from GWAS summary statistics. To ensure the independence of genetic instruments, SNPs were clumped for linkage disequilibrium [LD] within 1000-kb genomic distance and R-squares above 0.01 in according to the 1000 Genomes European reference panel [50]. Exposures and outcomes were harmonized in terms of effect allele and palindromic SNPs with minor allele frequency above 0.42 were excluded to minimize ambiguity. The procedures for genetic instrument selection were the same in both directions of analysis. Instrument strength was then evaluated by calculating the F-statistics for each SNP to reduce the potential weak instrument bias.

### Statistical analyses

#### Disease-wide bidirectional Mendelian randomization analysis

We performed the bidirectional two-sample MR analyses using the generalized summary-data-based Mendelian Randomization [GSMR] method [42] to estimate the causal effects for associations between IBD together with its subtypes [UC and CD] and the 104 disease-related traits. GSMR has superior power by taking the sampling variation into account and using the LD information of a referenced panel to avoid the power loss inherent in only using uncorrelated SNPs [51]. Meanwhile, the GSMR contains an implementation of the heterogeneity in dependent instruments outlier [HEIDI-outlier] test, which can be used to detect pleiotropy effects and exclude pleiotropy SNPs that are apparently associated with both confounding factors and diseases [42]. LD correlations between SNPs were estimated using European panel from the 1000 Genomes Project [50]. The Bonferroni correction method was used for multiple testing, and *p*-values less than 4.81 × 10^− 4^ [0.05/104] were considered significant. For diseases represented by multiple GWAS datasets from different sources, MR estimates were further meta-analyzed across sources to evaluate the consistency of associations. Estimates were combined on the log odds ratio scale, and pooled odds ratios, 95% confidence intervals, *P* values, number of datasets, I², and heterogeneity *P* values were reported. Meta-analyses were performed separately by MR direction and by IBD subtype outcome, including IBD, UC, and CD. All statistical analyses were conducted using TwoSampleMR v0.5.6 [46] and GSMR v1.1.0 [42] in R.

#### Summary data-based Mendelian randomization using eQTL data

On the basis of the two-sample MR analysis, we further performed SMR analysis using summary statistics from GWAS and eQTL studies to prioritize genes that are potentially causally associated with the risk of IBD [52, 53]. In the SMR analysis, cis-eQTL genetic variants were used as the instrumental variables for gene expression. We performed SMR analysis for gene expressions in lung, thyroid, muscle skeletal, and whole blood, and LCL separately. MR analysis was done using the SMR method followed by a HEIDI-outlier test as implemented in the software SMR [https://cnsgenomics.com/software/smr/] [53]. Top associated eQTL were selected using *P*_*eQTL*_ < 5 × 10^− 8^ and minor allele frequency greater than 0.01 to minimize the risk of weak instruments. We excluded SNP in strong LD [R^2^ > 0.8] with the top associated eQTL and excluded SNPs in low LD or not in LD [R^2^ < 0.05] with the top associated eQTL. Genes passed the HEIDI-outlier test [*p*-value > 0.05] with more than 10 SNPs were considered significant using Bonferroni correction with *p*-value less than 7.8 × 10^− 6^ [0.05/6410].

## Results

### Disease categories

Based on the ICD-10 code and disease descriptions, we divided 104 [49 distinct] exposures into eight major disease categories: blood and circulatory diseases, digestive system diseases, endocrine, nutritional and metabolic diseases, mental and nervous system diseases, musculoskeletal system and connective tissue diseases, neoplasms, respiratory system diseases, and skin diseases [Supplementary Table 1]. Blood and circulatory diseases compromised of 10 vascular and heart diseases and the digestive system category included 13 digestive diseases other than IBD such as celiac diseases. The largest category was endocrine, nutritional and metabolic disease category, which included 40 traits and most of the traits were diabetes-related diseases such as type 1 diabetes and thyroid-related diseases such as hypothyroidism. The skin category was the smallest category that only contained 5 traits such as atopic dermatitis and psoriasis vulgais. A wide range of mental and nervous system diseases such as major depression, Parkinson’s disease, schizophrenia, bipolar disorders, and multiple sclerosis were included in the analysis. The musculoskeletal system and connective tissue category contained 10 diseases, including ankylosing spondylitis [AS], systemic lupus erythematosus [SLE], psoriasis, and rheumatoid arthritis [RA]. There were 10 traits were categorized into respiratory system, which mainly were asthma from different data source or asthma measured under different conditions. Neoplasms was a relatively small category with 7 neoplasms such as prostate cancer, breast cancer and other cancers. This classification framework allowed for a systematic and biologically meaningful analysis of causal associations between IBD and a wide range of common diseases.

### Two-sample MR analysis

**Fig. 2:**
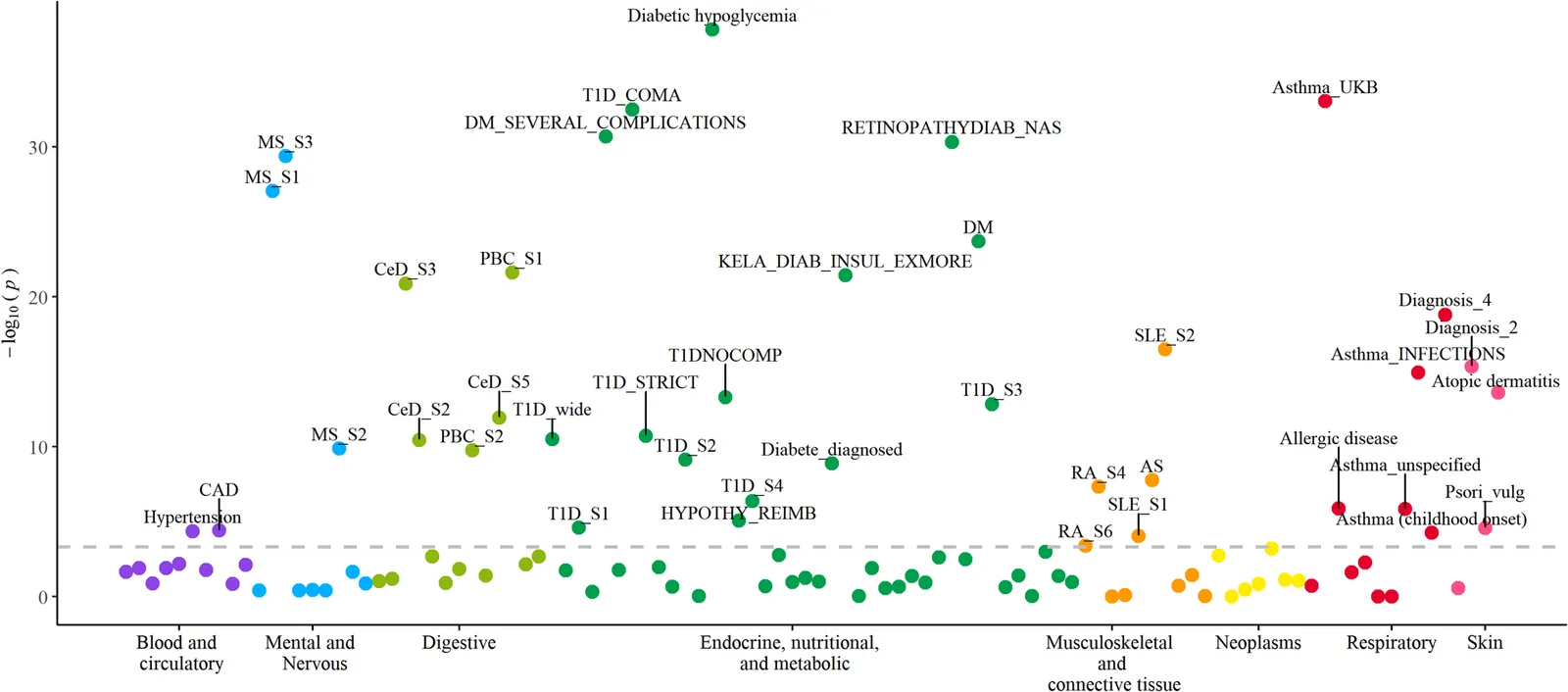
Forward-direction MR associations between 104 diseases and IBD. Results of generalized summary-data–based Mendelian randomization (GSMR) identifying diseases with significant causal effects on IBD after Bonferroni correction (*p* < 4.81 × 10^−4^).Diseases are grouped by clinical category, and effect estimates are presented with corresponding MR-derived significance levels

Of the 104 exposures that passed the filtering process, we found 38 exposures [19 distinct] from multiple categories with significant causal effects on IBD [Figure 2, Supplementary Table 2]. The strongest associations were observed for Diabetes-related traits in the endocrine, nutritional and metabolic diseases category and asthma-related traits in the respiratory system category. In the endocrine, nutritional and metabolic disease category, 14 out of 40 exposures were significantly associated with IBD, majority of which were diabetes-related traits. In digestive system, 5 traits, celiac disease (from 3 data sources) and primary biliary cholangitis (from 2 data sources), showed significant association with IBD. Three out of four traits in the skin category were significantly linked to IBD. However, no neoplasm was significantly associated with IBD. To further evaluate consistency across multiple GWAS sources for the same disease, we performed meta-analyses of MR estimates across data sources in both directions [Supplementary Table 9]. The meta-analysis showed directionally consistent pooled associations for several disease groups, including primary biliary cholangitis [PBC], celiac disease [CeD], multiple sclerosis [MS], rheumatoid arthritis [RA], systemic lupus erythematosus [SLE], and type 2 diabetes [T2D]. In contrast, type 1 diabetes [T1D] showed attenuated pooled estimates with substantial heterogeneity across data sources.

**Fig. 3:**
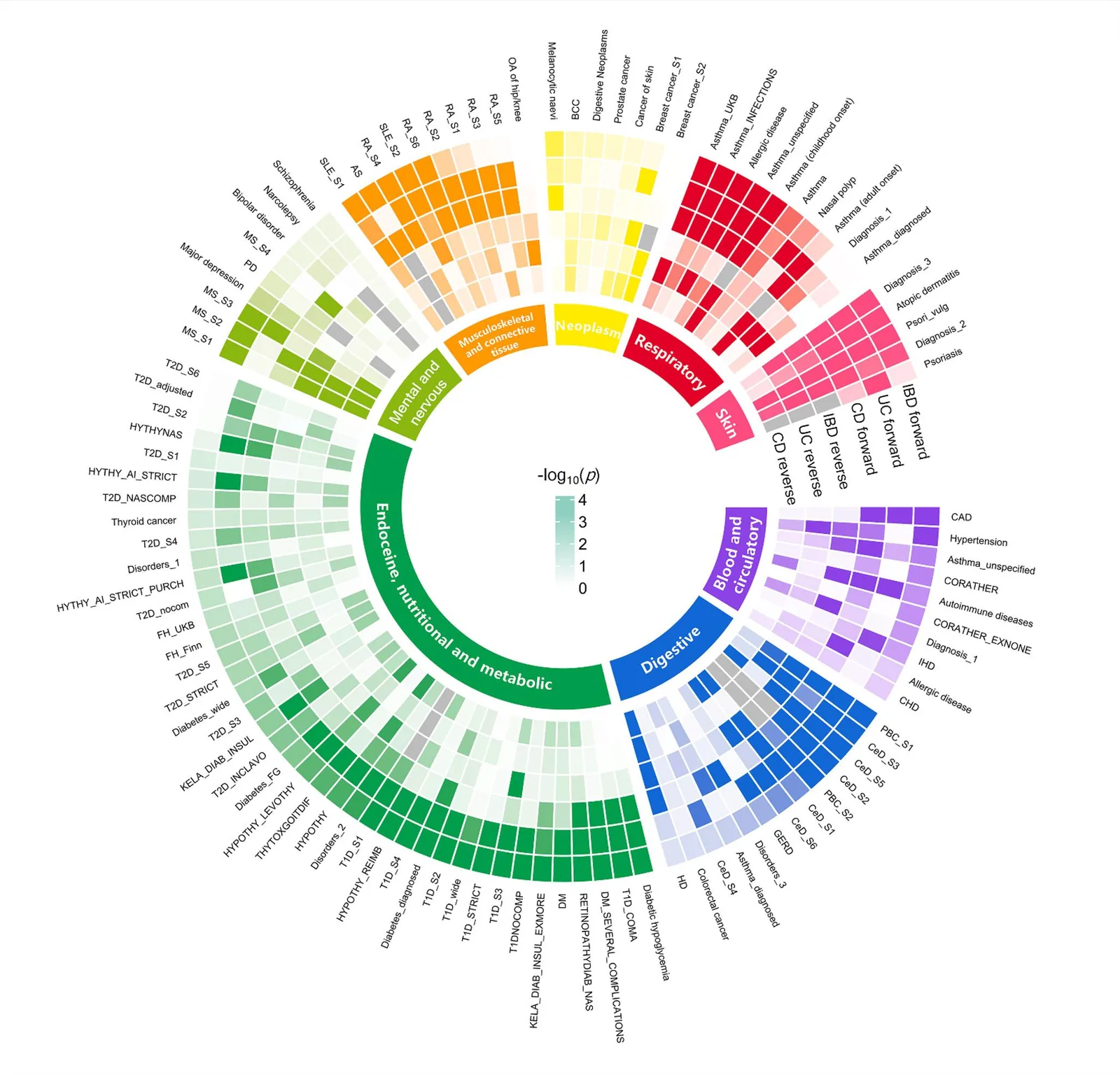
Comparison of forward and reverse MR associations. The figure presents results from both forward and reverse MR analyses, organized by disease category, allowing comparison of causal significance patterns across IBD, UC, and CD

When analyzing UC and CD separately, 34 and 52 significant exposures were identified, respectively [Figure 3]. UC showed a pattern of associations more similar to overall IBD, while CD demonstrated fewer significant associations and a distinct causal profile in the forward direction. For example, in the digestive system and the endocrine/metabolic categories, UC shared more similarities with IBD than CD. In contrast, in the respiratory system category, nearly all the asthma-related traits were significantly associated with IBD, UC and CD, suggesting shared pathophysiological mechanisms. In reverse direction, no clear pattern of causality was observed.

#### Respiratory diseases

**Fig. 4:**
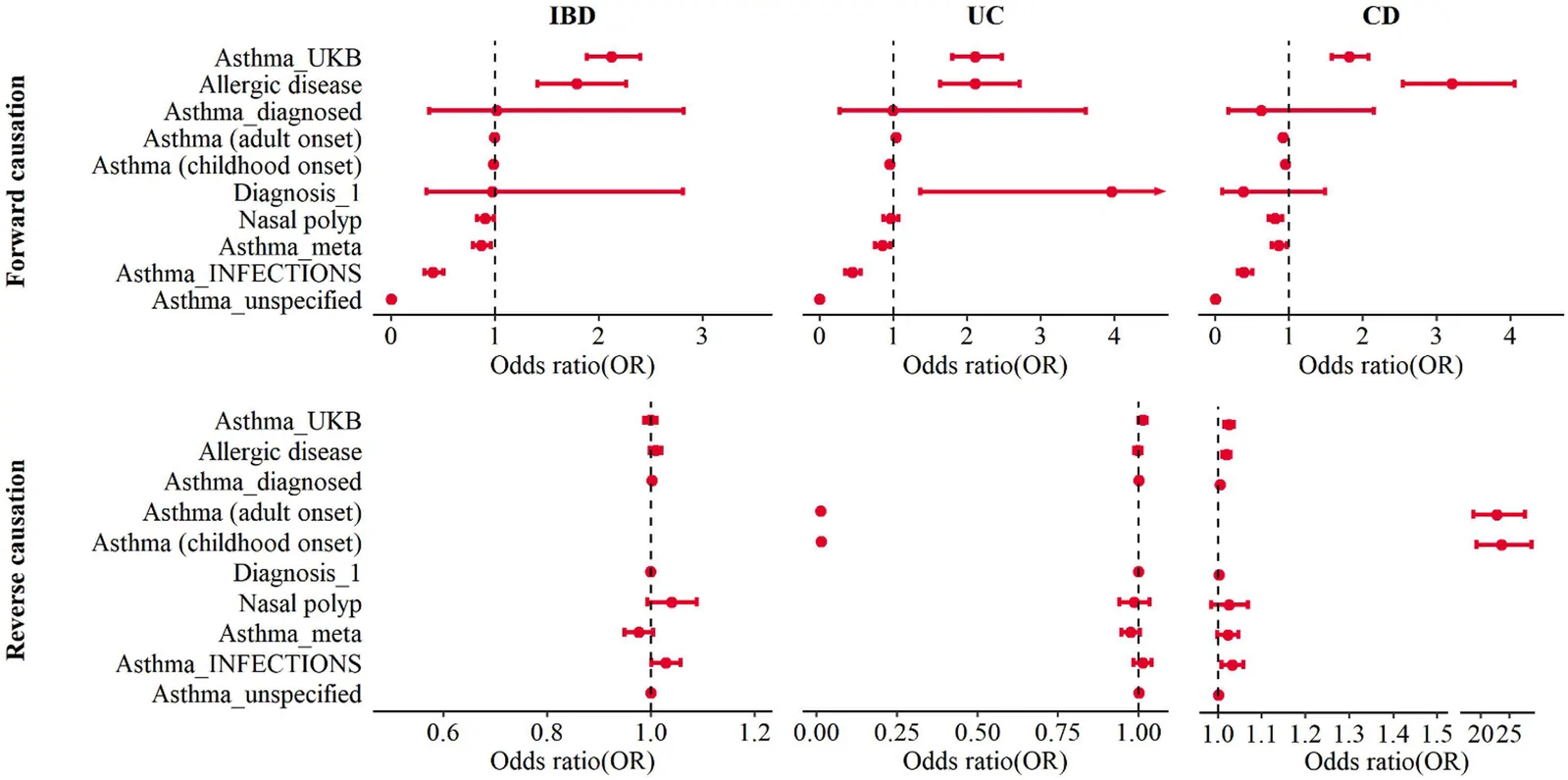
Bidirectional MR results for respiratory system diseases and IBD, UC, and CD

Results of respiratory diseases highlighted a strong and consistent causal relationship between asthma-related traits and IBD [Figure 4, Supplementary Table 2]. For example, Asthma_UKB, defined based on health records and self-reporting, was associated with an increased odds of IBD [*OR* 2.12, 95% CI 1.88, 2.40, *p* = 8.88 × 10^− 34^], UC [*OR* 2.11, 95% CI 1.80, 2.47, *p* = 3.38 × 10^− 20^], and CD [*OR* 1.81, 95% CI 1.58, 2.08, *p* = 1.83 × 10^− 17^]. Notably, unspecified asthma was significantly associated with a decreased risk of IBD, UC and CD, suggesting the importance of phenotypic heterogeneity in asthma. Furthermore, childhood-onset asthma was also significantly associated with a reduced risk of IBD [*OR* 0.98, 95% CI 0.98, 0.99, *p* = 5.43 × 10^− 5^]. Interestingly, asthma onset age significant influenced its effect on IBD subjects: childhood-onset asthma was associated with a decreased risk of both UC (*OR* 0.95, 95% CI 0.95, 0.96, *p* = 7.36 × 10^− 40^] and CD (*OR* 0.95, 95% CI 0.94, 0.96, *p* = 4.20 × 10^− 39^]; while adult-onset asthma was significantly associated with an increased risk of UC (*OR* 1.03, 95% CI 1.02, 1.05, *p* = 3.64 × 10^− 6^] but a decreased risk of CD (*OR* 0.92, 95% CI 0.91, 0.93, *p* = 4.76 × 10^− 33^].

Evidence of reverse causation was also observed [Figure 4, Supplementary Table 3]. CD was significantly associated with increased odds of Asthma_UKB [*OR* 1.03, 95% CI 1.01, 1.04, *p* = 4.37 × 10^− 6^], a dramatically promoted effects of childhood-onset asthma [*OR* 23.62, 95% CI 19.24, 29.00, *p* = 1.20 × 10^− 200^], and adult-onset asthma [*OR* 22.85, 95% CI 18.73, 27.89, *p* = 1.32 × 10^− 208^]. Conversely, UC was significantly associated with a decreased risk of both childhood- and adult-onset asthma [*p* < 10^− 170^]. These findings indicate a complex bidirectional relationship between IBD subtypes and different onsets of asthma, with distinct mechanisms underlying UC and CD in relation to asthma onset.

#### Endocrine, nutritional, and metabolic diseases

**Fig. 5:**
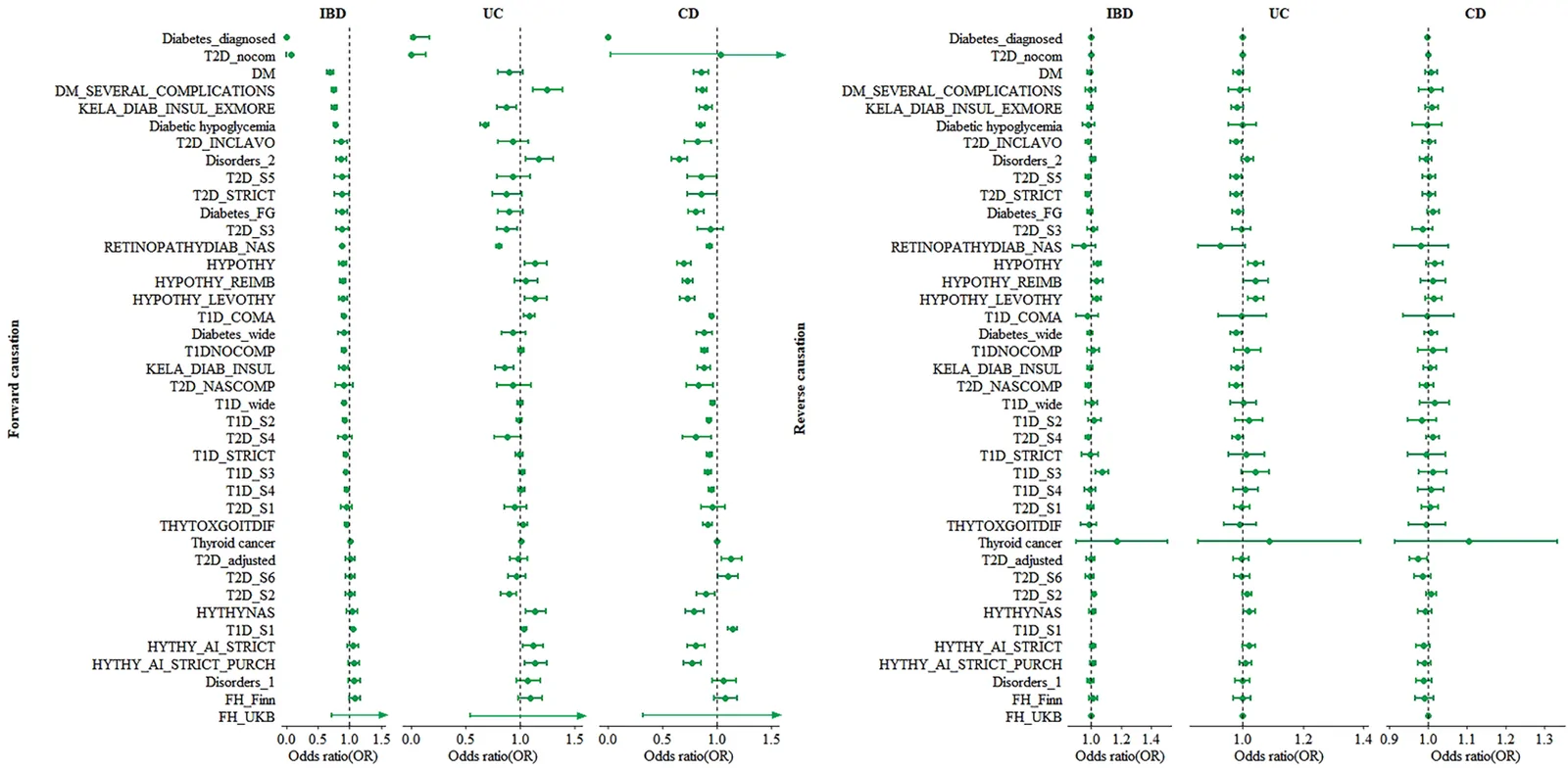
Bidirectional MR results for endocrine, nutritional, and metabolic diseases and IBD, UC, and CD

The strongest causal effect in this category were observed for diabetic hypoglycemia, which was associated with a reduced risk of IBD [*OR* 0.78, 95% CI 0.75, 0.81, *p* = 1.48 × 10^− 38^] [Figure 5, Supplementary Table 2]. Type I diabetes and other diabetic retinopathy were also linked to a decreased risk of IBD. However, no significant associations were found between type II diabetes and IBD.

Hypothyroidisms traits showed consistent trends in terms of the associations with IBD traits. In forward direction, hypothyroidism drug reimbursement [HYPOTHY_REIMB] was significantly associated with decreased odds of IBD [*OR* 0.89, 95% CI 0.75, 0.94, *p* = 8.36 × 10^− 6^]. In IBD subtypes, Hypothyroidism [HYPOTHY] [*OR* 0.73, 95% CI 0.69, 0.78, *p* = 5.47 × 10^− 20^] was found to be associated with decreased risk of CD. No significant reverse causation observed between the endocrine/metabolic diseases and IBD or its subtypes [Figure 5, Supplementary Table 3].

#### Musculoskeletal and connective tissue diseases

**Fig. 6:**
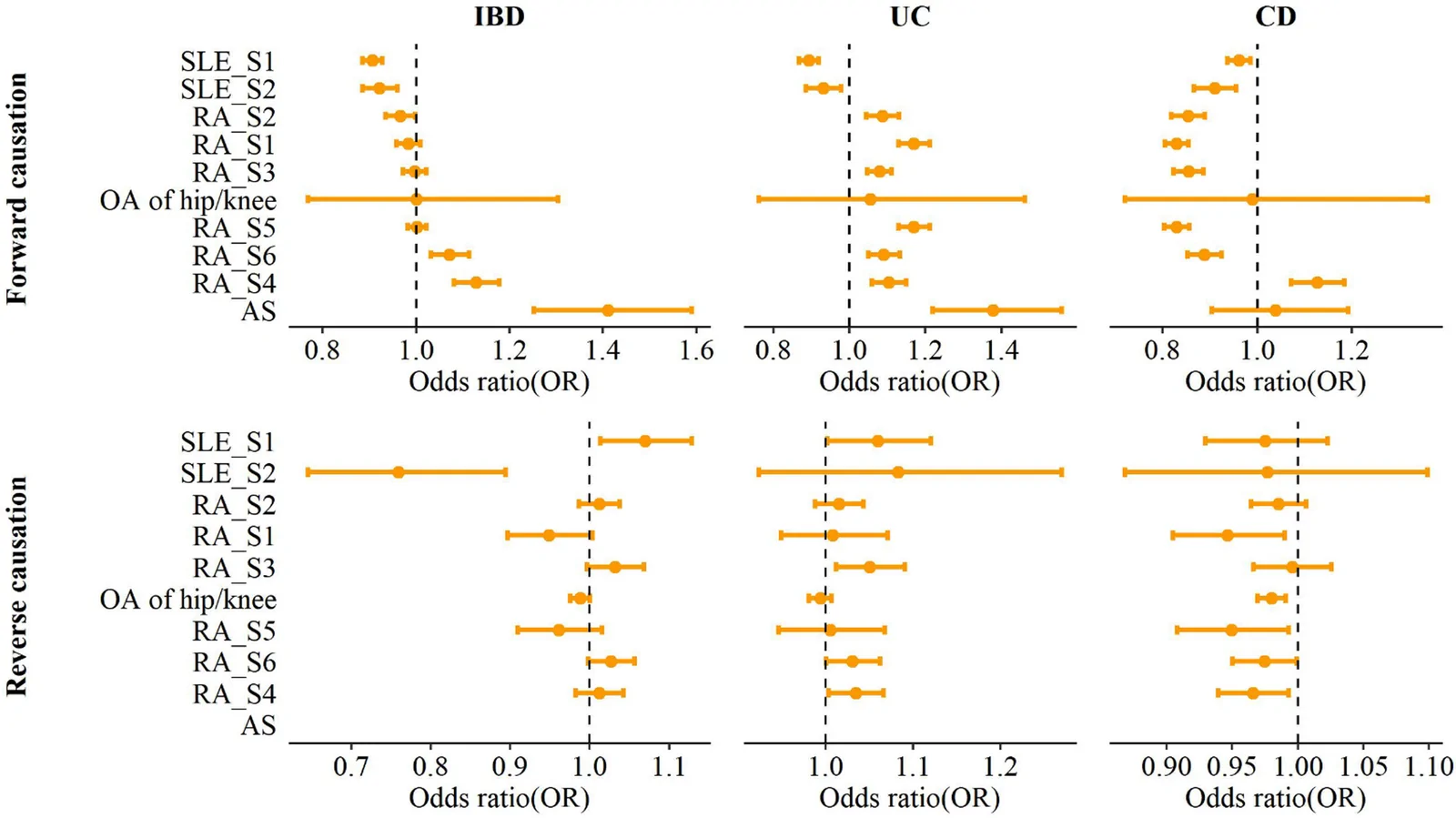
Bidirectional MR results for musculoskeletal and connective tissue diseases and IBD, UC, and CD

Rheumatoid arthritis (RA) was significantly associated with increased risk of IBD and UC, but with a decreased risk of CD [Figure 6, Supplementary Table 2]. For example, RA_S6 was associated 1.07 folds of odds of IBD [*OR* 1.07, 95% CI 1.03, 1.11, *p* = 3.97 × 10^− 4^], and 1.09 fold of odds of UC [*OR* = 1.09, 95% CI 1.05, 1.13, *p* = 8.74 × 10^− 6^], while associated with 11% decreased odds of CD [*OR* 0.89, 95% CI 0.85, 0.92, *p* = 7.55 × 10^− 9^]. In contrast, systemic lupus erythematosus [SLE] was significantly associated with a decreased risk of IBD, UC, and CD. Ankylosing spondylitis [AS] was associated with an increased risk of IBD [*OR* 1.41, 95% CI 1.25, 1.59, *p* = 1.68 × 10^− 8^], UC [*OR* 1.38, 95% CI 1.22, 1.56, *p* = 2.93 × 10^− 7^], but no significant association with CD. In reversion MR analysis, only OA of hip/knee showed evidence with CD [*OR* 0.89, 95% CI 0.97, 0.99, *p* = 2.76 × 10^− 4^] [Supplementary Table 3].

#### Mental and nervous system diseases

**Fig. 7:**
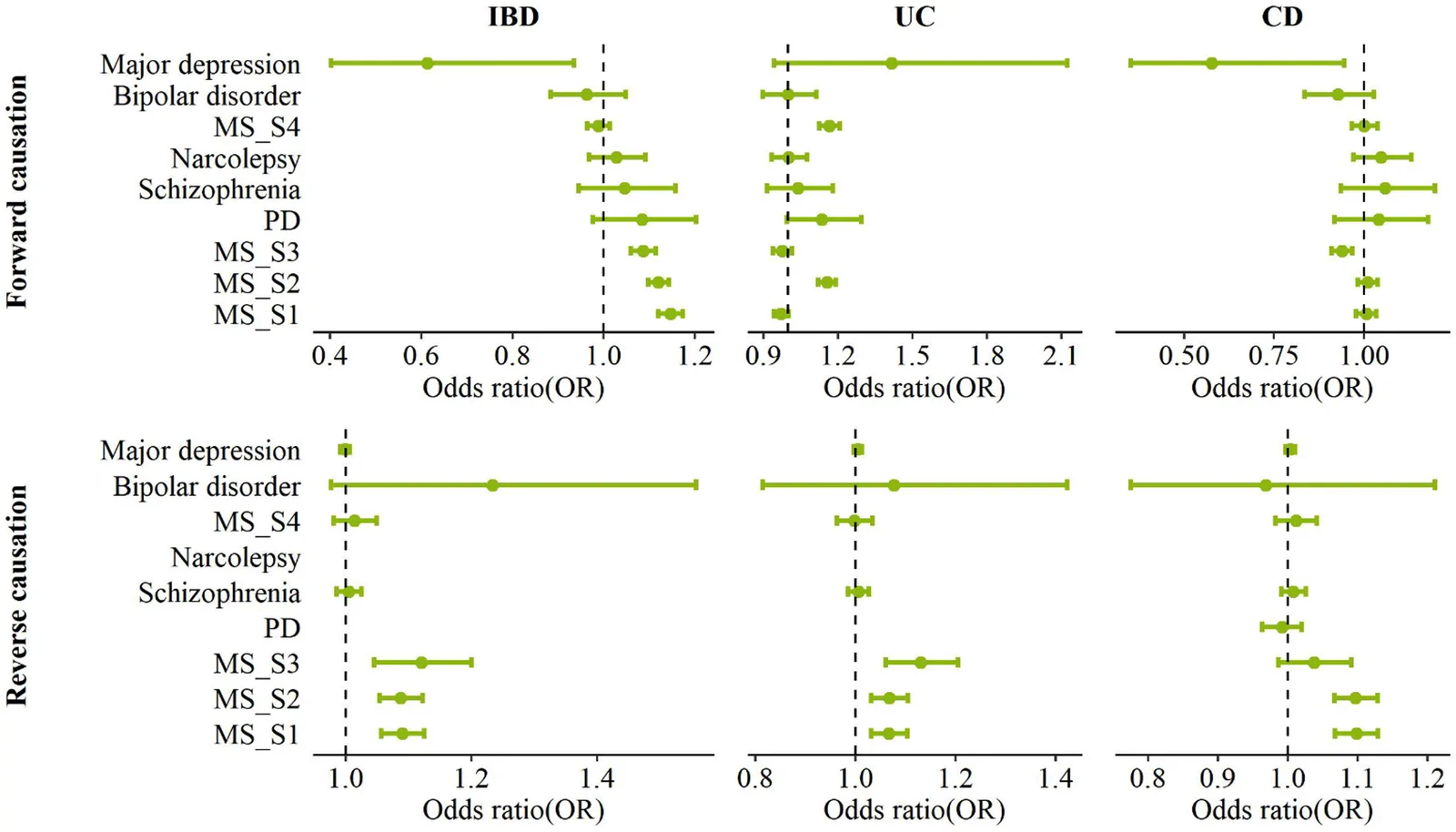
Bidirectional MR results for mental and nervous system diseases and IBD, UC, and CD

All three data sources of multiple sclerosis [MS_S2, S3, S4] showed the most consistent and significant risk associations with IBD [Figure 7, Supplementary Table 2]. Among them, MS_S2 and MS_S4 from a were significantly associated with an increased risk of UC, while MS_S3 was associated with a decreased risk of CD. In the reverse direction, all IBD subtypes were significantly associated with increased risk of MS [Figure 7, Supplementary Table 3].

#### Other diseases

Other than the above four main categories, several other diseases were also significantly associated with IBD or its subtypes [Supplementary Table 2]. For example, primary biliary cholangitis [PBC] from two data sources was significantly associated with increased risk of IBD and subtypes; psoriasis vulgaris, from the skin disease category, was associated with reduced risk of IBD and its subtypes. A noticeable finding was that high blood pressure was associated with a decreased risk of IBD and UC, though the association with CD was not significant.

### SMR analysis identified pleiotropic genes associated with IBD

Using summary-data-based Mendelian randomization (SMR) and integrating GWAS and eQTL data, we identified 168 genes [284 probes] significantly associated with IBD across five tissues or cell lines (lung, whole blood, muscle skeletal tissue, thyroid, and LCL), of which 52 genes [52 probes] passed the HEIDI-outlier test, indicating potential pleiotropy or causal associations [Supplementary Tables 4–8].

The majority of these genes were identified in whole blood [*N* = 22], followed by thyroid [*N* = 21], muscle skeletal tissue [*N* = 15], LCL [*N* = 5], and lung [*N* = 1]. Notable findings include: *RGS14* [ENSG00000169220.13], a regulator of G-protein family, was significantly associated with both MS [*P*_SMR_ = 3.07 × 10^− 6^] [Table 1] and IBD in muscle, skeletal tissue [*P*_SMR_ = 1.89 × 10^8^], and thyroid [*P*_SMR_ = 6.48 × 10^8^] [Supplementary Tables 6 and 7]; [54] *EARP2* [ENSG00000164308.12] was significantly associated with IBD in muscle skeletal tissue [*P*_SMR_ = 4.46 × 10^− 12^] and thyroid [*P*_SMR_ = 5.03 × 10^− 12^]; Notably, *CARD9* [ENSG00000187796.9], a gene involves in asthma-related signaling pathway, was the most significant gene associated with IBD in whole blood [*P*_SMR_ = 6.51 × 10^− 19^] and LCL [*P*_SMR_ = 8.88 × 10^− 11^] [55, 56]. These results highlight shared genetic mechanisms between IBD and immune-related diseases, particularly asthma and multiple sclerosis.

**Table 1:** Most significant gene passed HEIDI-outlier test in 5 tissues or cell lines in SMR analyses

Tissues	Top probe	Top gene	Top SNP	CHR	SNP Position	*P*_SMR_
Lung	ENSG00000169220.13	*RGS14*	rs35716097	5	176,806,636	3.07 × 10^− 6^
Whole Blood	ENSG00000187796.9	*CARD9*	rs4078099	9	139,267,533	6.51 × 10^− 19^
Muscle skeletal	ENSG00000164308.12	*ERAP2*	rs2910686	5	96,252,589	4.46 × 10^− 12^
Thyroid	ENSG00000164308.12	*ERAP2*	rs2910686	5	96,252,589	5.03 × 10^− 12^
LCL	ENSG00000187796.9	*CARD9*	rs10870165	9	139,316,601	8.88 × 10^− 11^

## Discussion

The disease-wide, bidirectional, two-sample MR analysis offers novel insights into the causal relationships between a broad spectrum of common diseases and IBD, including its subtypes UC and CD. By leveraging GWAS and eQTL data, we identified multiple diseases and pleiotropic genes significantly associated with IBD, thereby uncovering potential targets for early prevention and therapeutic intervention. Importantly, we observed distinct causal patterns between UC and CD, highlighting the heterogeneity in their underlying pathophysiological mechanisms.

### Asthma and IBD: a complex bidirectional relationship

The causal association between asthma and IBD were comprehensively investigated from multi-perspectives in this study. We considered various asthma phenotypes, including unspecified asthma, physician-diagnosed asthma, childhood- vs. adult-onset asthma, as well as data from different sources. These analyses were conducted within a bidirectional MR framework, which may have direct implications for the clinical management of patients with IBD subtypes.

Our results showed asthma phenotypic heterogeneity may lead to divergent effects on IBD. Asthma_UKB, comprised of health records and self-reported asthma, was associated with an increased risk of IBD. In contrast, unspecified asthma showed a protective effect of IBD. Moreover, childhood-onset asthma was associated with a reduced risk of both UC and CD, whereas adult-onset asthma was linked to an increased risk of UC but a reduced risk of CD, indicating mechanistic differences between IBD subtypes and asthma onset age.

Evidence of reverse causation was also observed. CD was associated with increased risk of asthma, while UC was associated with decreased risk, suggesting bidirectional crosstalk between the gut and respiratory systems. Early-life environment exposures have been linked to respiratory tract infections and changes in the gut bacterial community structure [57]. A study finds that pulmonary allergy could release signals that direct alterations in the intestinal bacterial community structure [58]. Notably, the cross-talk or the gut-lung axis is bidirectional [59, 60]. A pilot study finds significant relationship between gut microbiota and lung function linked with asthma phenotype, which supports the bidirectional association of IBD and asthma found in our analysis [61]. 

The observed differential effects of childhood-onset versus adult-onset asthma on IBD subtypes may be explained by the timing of immune system modulation and the nature of underlying immune responses. The hygiene hypothesis posits that early-life exposure to co-evolved microbial agents is critical for the proper development of immunoregulatory circuits, with organisms such as commensal bacteria inducing tolerance through pathways involving toll-like receptor-2 (TLR-2) signaling [62, 63]. Childhood-onset asthma may not simply reflect immune dysregulation, but could instead be a marker of early immune engagement with such tolerogenic microbes—resulting in a broadly regulated immune phenotype that confers protection against both UC and CD. In contrast, adult-onset asthma often arises in individuals with reduced early microbial exposure [64], leading to a poorly regulated immune system prone to exaggerated Th2 and Th17 responses upon later environmental challenge [65]. This Th17-skewed inflammation may share pathogenic mechanisms with UC, thereby increasing its risk, while potentially suppressing Th1-dominated responses and exerting a protective effect against CD [66]. Consistent with this interpretation, UC is considered to arise from the interaction of genetic predisposition, environmental exposures, epithelial barrier defects, gut microbiota alterations, and dysregulated immune responses [67]. Thus, the divergent associations may reflect fundamental differences in immune ontogeny, where early-life immune experiences shape the trajectory of chronic inflammatory disease risk in adulthood.

Furthermore, the SMR analysis identified *CARD9*, a gene implicated in asthma-related signaling pathways [55, 68], as significantly associated with IBD in whole blood, lymphoblastoid cell lines (LCLs), and muscle skeletal tissue. This provides genetic evidence supporting a shared mechanism between asthma and IBD. Additionally, other respiratory and allergic diseases, such as nasal polyps, showed significant causal association with IBD, reinforcing the notion of pulmonary involvement in IBD pathogenesis.

Our findings align with an MR study [69] suggesting that childhood-onset asthma reduces the risk of IBD, particularly UC, in adulthood. The differential effects observed between childhood- and adult-onset asthma may reflect distinct immunological and developmental pathways, underscoring the importance of age-stratified analyses in future studies.

### Autoimmune diseases and IBD: conflicting observational evidence

The association between autoimmune diseases, such as type I diabetes [T1D] and hypothyroidisms [HYPOTHY], and IBD was inconsistent in observational studies. For instance, a study [70] on Korean population reported an increased risk of diabetes in IBD patients, while others found no significant association [37] or even a modestly increased risk of T1D prior to IBD diagnosis, particularly in UC patients [71].

In our study, T1D was associated with a decreased risk of IBD, which may be attributed to the protective effects of certain T1D risk alleles, such as *PTPN22* and interleukin-27, particularly against CD [72]. These findings suggest that genetic susceptibility profiles may differ between diseases and highlight the importance of disentangling causal mechanisms through genetically informed methods like MR. However, the across-source meta-analysis showed that the pooled association between T1D and IBD-related outcomes was attenuated toward the null with substantial heterogeneity across GWAS datasets [Supplementary Table 9]. Thus, the protective association between T1D and IBD should be interpreted cautiously and may reflect source-specific differences in GWAS design, disease definition, or instrument selection.

Similarly, we observed a protective effect of hypothyroidism on IBD, which contrasts with findings from observational studies reporting an increased risk of IBD in patients with congenital hypothyroidism [73]. These discrepancies may stem from sex differences, as our analysis included both sexes, and hormonal or immunological factors may differentially influence disease susceptibility [74]. 

### SLE, IBD, and shared gastrointestinal symptom

SLE has been reported to increase the risk of IBD in many observational studies [75–77]. However, our MR analysis revealed a protective effect of SLE on IBD and its subtypes, suggesting that confounding due to overlapping gastrointestinal [GI] symptoms may have influenced previous findings. For instance, drug-induced lupus, a known side effect of certain IBD therapies, and GI manifestations of SLE may complicate the clinical differentiation of these diseases and introduce diagnostic bias [77–81]. By using MR, which minimizes confounding, we provide evidence that SLE may have a protective role in IBD, further demonstrating the utility of MR in clarifying complex disease relationships.

### Mental and nervous system diseases: limited evidence of association

While the gut-brain axis has been widely discussed in the context of IBD and mental health [13, 16, 20], our analysis did not find robust evidence linking IBD with common mental disorders, such as bipolar disorder, major depressive disorder, Parkinson’s disease, and schizophrenia. However, we did find a significant association between MS and increased risk of IBD and UC, but not CD, using multiple MS data sources. This is consistent with a recent meta-analysis [23] indicating that patients with MS have a higher prevalence of IBD compared to controls. Meanwhile, our SMR analysis identified *RGS14*, a gene that has been reported to be downregulated in T and non-T cells in MS patients [82], as significantly associated with IBD. This suggests that shared genetic and immune pathways may underlie the observed relationship between MS and IBD.

### Strengths and limitations

Our study has several notable strengths: Firstly, to our knowledge, this is the largest bidirectional disease-wide MR analysis between IBD and 104 common diseases, spanning eight major disease categories. Recent targeted Mendelian randomization MR studies have examined selected traits related to IBD, including spleen volume and plant-based food preferences rich in polyphenols, while other MR studies have focused on related gastrointestinal outcomes such as colorectal cancer [83–85]. In contrast, our study applied a disease-wide bidirectional MR framework across multiple disease categories and evaluated IBD, UC and CD separately. This broad scope enables the identification of novel risk factors and comorbidities. Secondly, the use of GSMR method with HEIDI-outlier testing enhances the reliability of our findings by detecting and excluding pleiotropic instruments. Thirdly, by incorporating GWAS and eQTL data through SMR analysis, we were able to identify pleiotropic genes that may mediate the causal relationships between diseases and IBD. This provides biological insights into the molecular mechanisms underlying these associations. Lastly, the bidirectional MR framework allows for the detection of both risk and protective effects, as well as reverse causation, offering a comprehensive view of disease interplay.

Despite these strengths, our study has several limitations: First, we were limited by available variables in the IEU database, which may not include all the diseases that may have risk or protective effects on IBD. Secondly, the use of correction for multiple testing may also conceal some possible important associations. For example, there are possible bidirectional effects between MD and IBD according to some studies while due to the conservative threshold, the relationship between MD and IBD were claimed to be insignificant in this study. While we highlight some of the significantly associated diseases, the full results should be reviewed in Supplementary Table 2, for other possible not significant yet attractive associations. Moreover, all GWAS datasets included in this study were restricted to individuals of European ancestry, which limits the generalizability of our findings to other populations. The genetic architecture of IBD may differ across ancestries. Therefore, causal estimates derived from European-ancestry GWAS may not fully reflect the relationship between IBD and related diseases in non-European populations. Future studies incorporating large-scale multi-ancestry GWAS datasets are needed to validate these findings and better characterize ancestry-specific or shared causal relationships involving IBD. Finally, potential sample overlap represents an additional limitation of this study. Because we used publicly available GWAS summary statistics, the degree of overlap between the International IBD Genetics Consortium and UK Biobank-derived datasets could not be directly quantified. Previous methodological study [86] has shown that participant overlap in two-sample MR can introduce bias, particularly in the presence of weak instruments, and that the magnitude of bias depends on both the proportion of sample overlap and instrument strength. However, the impact of sample overlap is expected to be less pronounced when genetic instruments are strong and GWAS sample sizes are large [87]. However, it is unlikely to substantially compromise the overall reliability of our main findings given the use of large GWAS summary statistics and the consistently high F-statistics of all genetic instruments, which suggest a low risk of weak instrument bias [Supplementary Tables 10–11]. Meanwhile, we note that certain estimates with unusually large effect sizes (e.g., the reverse association between CD and childhood-onset asthma, OR ~ 23.6) should be interpreted with caution. The extreme magnitude may partly reflect sample overlap or model instability, which could inflate the effect size. However, these factors are more likely to affect the precise effect-size estimate than the overall causal direction; notably, the positive association aligns with biological evidence supporting gut–lung crosstalk. Future studies using independent, non-overlapping GWAS datasets or summary-statistics methods that account for sample overlap are warranted to validate these findings.

## Conclusions

In this study, we comprehensively screened the causes and consequences of IBD traits to a total of 104 disease-related traits. Our bidirectional MR and SMR analyses provide robust evidence for causal relationships between asthma, multiple sclerosis, and IBD, as well as distinct mechanisms underlying UC and CD. The identification of pleiotropic genes, such as *CARD*9 and *RGS*14, offers promising therapeutic targets and biomarkers for early detection and intervention. These findings contribute to a deeper understanding of IBD pathogenesis, highlight the importance of immune-mediated diseases, and pave the way for targeted prevention and treatment strategies.

## Supplementary Information

Below is the link to the electronic supplementary material.

Supplementary Material 1

Supplementary Material 2

## Data Availability

This study used publicly available summary-level data of GWAS. The GWAS summary statistics for IBD, UC and CD is available on the websites https://www.ebi.ac.uk/gwas/downloads/summary-statistics using accession number GCST003043, GCST003045, and GCST003044. The 104 exposure datasets are available on the websites https://gwas.mrcieu.ac.uk/datasets/ using the ID listed in the ID column in Supplementary Table 2. The summary-level data used in the SMR analysis were obtained from the GTEx consortium and GEUVADIS consortium and can be accessed on the website https://yanglab.westlake.edu.cn/software/smr/#DataResource .
