# Supplementary material for: The impact for causal associations between common diseases and inflammatory bowel disease: a disease-wide bidirectional Mendelian randomization study
Source: Inflamm Res. 2026 Sep 5;75(1):207. doi: 10.1007/s00011-026-02304-8 (PMC13546168; doi:10.1007/s00011-026-02304-8)
Supplement: Supplementary file 1 [file 11_2026_2304_MOESM1_ESM.pdf]

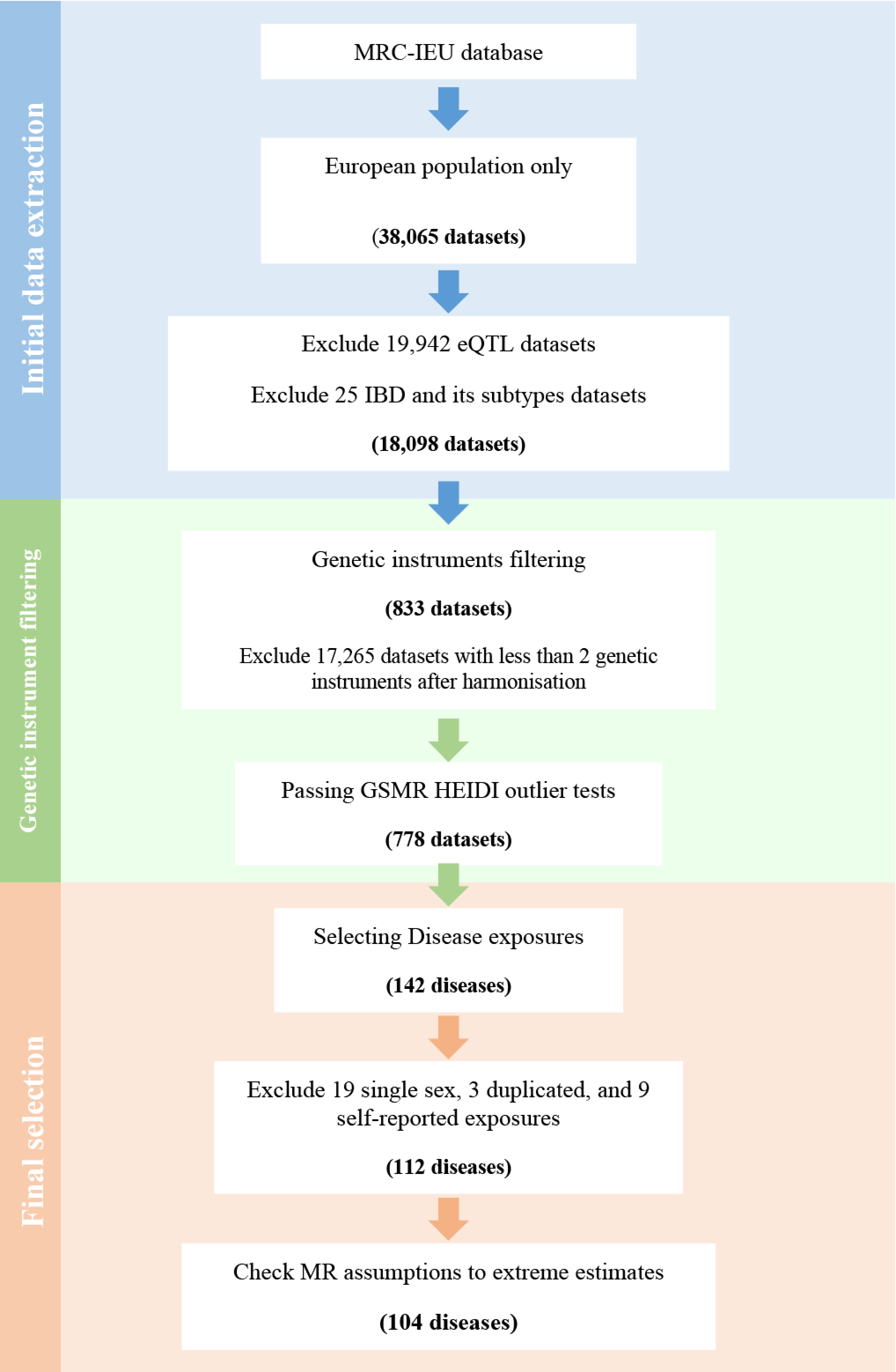
Initial data extraction
MRC-IEU database
European population only
(38,065 datasets)
Exclude 19,942 eQTL datasets
Exclude 25 IBD and its subtypes datasets
(18,098 datasets)
Genetic instrument filtering
Genetic instruments filtering
(833 datasets)
Exclude 17,265 datasets with less than 2 genetic instruments after harmonisation
Passing GSMR HEIDI outlier tests
(778 datasets)
Final selection
Selecting Disease exposures
(142 diseases)
Exclude 19 single sex, 3 duplicated, and 9 self-reported exposures
(112 diseases)
Check MR assumptions to extreme estimates
(104 diseases)
