# Supplementary material for: The impact for causal associations between common diseases and inflammatory bowel disease: a disease-wide bidirectional Mendelian randomization study
Source: Inflamm Res. 2026 Sep 5;75(1):207. doi: 10.1007/s00011-026-02304-8 (PMC13546168; doi:10.1007/s00011-026-02304-8)
Supplement: Supplementary file 2 [file 11_2026_2304_MOESM2_ESM.pdf]

**Supplementary Table 1. Disease code and category**

| Trait | ICD10 | Category |
|---|---|---|
| Diabetic hypoglycemia | E11 | Endocrine |
| Asthma | J45 | Respiratory |
| Type 1 diabetes with coma | E10 | Endocrine |
| Diabetes, several complications | E11 | Endocrine |
| Other diabetic retinopathy | E11 | Endocrine |
| Multiple sclerosis | G35 | Mental and Nervous |
| Multiple sclerosis | G35 | Mental and Nervous |
| Diabetes mellitus | E08.9 | Endocrine |
| Primary biliary cholangitis | K74.3 | Digestive |
| excluded) | E11 | Endocrine |
| Celiac disease | K90.0 | Digestive |
| Blood clot DVT bronchitis emphysema asthma rhinitis eczema allergy diagnosed by doctor: None of the above | Not applicable | Skin |
| Systemic lupus erythematosus | M32.9 | Musculoskeletal system and connective tissue |
| Asthma-related infections | Not applicable | Respiratory |
| Atopic dermatitis | L20.9 | Skin |
| Type 1 diabetes without complications | E10 | Endocrine |
| Type 1 diabetes | E10 | Endocrine |
| Celiac disease | K90.0 | Digestive |
| Type 1 diabetes, strict definition | E10 | Endocrine |
| Type 1 diabetes, wide definition, subgroup 1 | E10 | Endocrine |
| Celiac disease | K90.0 | Digestive |
| Multiple sclerosis | G35 | Mental and Nervous |
| Primary biliary cirrhosis | K74.3 | Digestive |
| Type 1 diabetes | E10 | Endocrine |

| | | |
|---|---|---|
| Diabetes diagnosed by doctor | Not applicable | Endocrine |
| Ankylosing spondylitis | M45 | Musculoskeletal system and connective tissue |
| Rheumatoid arthritis | M06.9 | Musculoskeletal system and connective tissue |
| Type 1 diabetes | E10 | Endocrine |
| Allergic disease (asthma, hay fever or eczema) | Not applicable | Respiratory |
| Diagnoses - secondary ICD10: J45.9 Asthma, unspecified | J45.9 | Respiratory |
| Hypothyroidism, drug reimbursement | E03.2 | Endocrine |
| Type 1 diabetes | E10 | Endocrine |
| Psoriasis vulgaris | L40.0 | Skin |
| Coronary artery disease | I25.10 | Blood and circulatory |
| Vascular/heart problems diagnosed by doctor: High blood pressure | Not applicable | Blood and circulatory |
| Asthma (childhood onset) | J45 | Respiratory |
| Systemic lupus erythematosus | M32.9 | Musculoskeletal system and connective tissue |
| Rheumatoid Arthritis | M06.9 | Musculoskeletal system and connective tissue |
| Blood clot DVT bronchitis emphysema asthma rhinitis eczema allergy diagnosed by doctor: Hayfever allergic rhinitis or eczema | Not applicable | Skin |
| Disorders of the thyroid gland | E07.9 | Endocrine |
| Hypothyroidism (congenital or acquired) | E03.2 | Endocrine |
| Diagnoses - main ICD10: D22 Melanocytic naevi | D22 | Neoplasms |
| Celiac disease | K90.0 | Digestive |
| Celiac disease | K90.0 | Digestive |
| Thyrotoxicosis with diffuse goitre | E05.0 | Endocrine |
| Hypothyroidism, levothyroxin purchases | E03.2 | Endocrine |
| Asthma | J45 | Respiratory |
| Myocardial infarction | I21 | Blood and circulatory |
| Gastroesophageal reflux disease | K21 | Digestive |
| Coronary atherosclerosis | I25 | Blood and circulatory |
| Diabetes, varying definitions | Not applicable | Endocrine |

| | | |
|---|---|---|
| Autoimmune diseases | D89.9 | Blood and circulatory |
| Coronary atherosclerosis (no controls excluded) | I25 | Blood and circulatory |
| Type 2 diabetes, definitions combined, including avohilmo | E11 | Endocrine |
| Disorders of gallbladder, biliary tract and pancreas | K87 | Digestive |
| Vascular/heart problems diagnosed by doctor: None of the above | Not applicable | Blood and circulatory |
| Diabetes, insuline treatment (Kela reimbursement) | Z79.4 | Endocrine |
| Type 2 diabetes | E11 | Endocrine |
| Ischaemic heart disease, wide definition | I20-I25 | Blood and circulatory |
| Major depression | F32.0–F33.3 | Mental and Nervous |
| Nasal polyp | J33.9 | Respiratory |
| Rheumatoid arthritis | M06.9 | Musculoskeletal system and connective tissue |
| Other diabetes, wide definition | Not applicable | Endocrine |
| Mouth/teeth dental problems: Mouth ulcers | K12.30 | Digestive |
| Type 2 diabetes, strict (exclude DM1) | E11 | Endocrine |
| Type 2 diabetes, definitions combined | E11 | Endocrine |
| Pure hypercholesterolaemia | E78.0 | Endocrine |
| Celiac disease | K90.0 | Digestive |
| Basal cell carcinoma | C44.91 | Neoplasms |
| and anal canal | D12 | Neoplasms |
| Colorectal cancer | C18.9 | Digestive |
| Diagnoses - secondary ICD10: E78.0 Pure hypercholesterolaemia | E78.0 | Endocrine |
| Diagnoses - secondary ICD10: E11.9 Without complications | E11.9 | Endocrine |
| Hypothyroidism, strict autoimmune, 3 medication purchases required | E03.2 | Endocrine |
| Disorders of lipoprotein metabolism and other lipidaemias | E78 | Endocrine |
| Haemorrhoidal disease | K64 | Digestive |
| Myocardial infarction | I21 | Blood and circulatory |
| Parkinson's disease | G20 | Mental and Nervous |
| Coronary heart disease | I25 | Blood and circulatory |

| | | |
|---|---|---|
| Prostate cancer | C61 | Neoplasms |
| Rheumatoid arthritis | M06.9 | Musculoskeletal system and connective tissue |
| Asthma (adult onset) | J45 | Respiratory |
| Cancer of skin | C44 | Neoplasms |
| Type 2 diabetes | E11 | Endocrine |
| Thyroid cancer | C73 | Endocrine |
| Type 2 diabetes with other specified/multiple/unspecified complications | E11 | Endocrine |
| Hypothyroidism, strict autoimmune | E03.2 | Endocrine |
| Type 2 diabetes | E11 | Endocrine |
| Psoriasis | L40.0 | Skin |
| Breast cancer | C50 | Neoplasms |
| Multiple sclerosis | G35.9 | Mental and Nervous |
| Bipolar disorder | F31 | Mental and Nervous |
| Narcolepsy | G47.4 | Mental and Nervous |
| Schizophrenia | F20 | Mental and Nervous |
| Hypothyroidism, other/unspecified | E03.2 | Endocrine |
| Rheumatoid arthritis | M06.9 | Musculoskeletal system and connective tissue |
| Rheumatoid arthritis | M06.9 | Musculoskeletal system and connective tissue |
| Type 2 diabetes | E11 | Endocrine |
| Type 2 diabetes (adjusted for BMI) | E11 | Endocrine |
| Type 2 diabetes | E11 | Endocrine |
| Blood clot DVT bronchitis emphysema asthma rhinitis eczema allergy diagnosed by doctor: Asthma | Not applicable | Respiratory |
| Breast cancer | C50 | Neoplasms |
| Doctor diagnosed asthma | Not applicable | Respiratory |
| Osteoarthritis of the hip or knee | M16 | Musculoskeletal system and connective tissue |

**Supplementary Table 2. Disease-wide MR analysis results in forward direction**

| | | IBD | | UC | | CD | | | |
|---|---|---|---|---|---|---|---|---|---|
| **Trait** | **Name** | **OR [95%CI]** | **pval** | **OR [95%CI]** | **pval** | **OR [95%CI]** | **pval** | **Category** | **GWAS-ID** |
| Diabetic hypoglycemia | Diabetic hypoglycemia | 0.78 [0.75, 0.81] | 1.480E-38 | 0.68 [0.64, 0.71] | 1.723E-42 | 0.85 [0.82, 0.89] | 1.310E-13 | Endocrine, nutritional and metabolic | finn-b-DM_HYPOGLYC |
| Asthma | Asthma_UKB | 2.12 [1.88, 2.40] | 8.880E-34 | 2.11 [1.80, 2.47] | 3.385E-20 | 1.81 [1.58, 2.08] | 1.857E-17 | Respiratory | ebi-a-GCST90014325 |
| Type 1 diabetes with coma | T1D_COMA | 0.90 [0.88, 0.92] | 3.090E-33 | 1.08 [1.04, 1.13] | 4.706E-04 | 0.95 [0.93, 0.97] | 8.012E-08 | Endocrine, nutritional and metabolic | finn-b-E4_DM1COMA |
| Diabetes, several complications | DM_SEVERAL_COMPLICATIONS | 0.75 [0.71, 0.78] | 2.000E-31 | 1.25 [1.12, 1.39] | 8.204E-05 | 0.86 [0.82, 0.91] | 6.053E-08 | Endocrine, nutritional and metabolic | finn-b-DM_SEVERAL_COMPLICATIONS |
| Other diabetic retinopathy | RETINOPATHYDIAB_NAS | 0.88 [0.86, 0.90] | 4.620E-31 | 0.81 [0.78, 0.83] | 1.192E-38 | 0.93 [0.91, 0.96] | 2.069E-07 | Endocrine, nutritional and metabolic | finn-b-H7_RETINOPATHYDIAB_NAS |
| Multiple sclerosis | MS_S1 | 1.15 [1.12, 1.17] | 4.150E-30 | 0.97 [0.94, 1.00] | 7.516E-02 | 1.01 [0.98, 1.04] | 6.343E-01 | Mental and Nervous | ieu-a-1025 |
| Multiple sclerosis | MS_S2 | 1.12 [1.10, 1.14] | 8.690E-28 | 1.16 [1.12, 1.19] | 2.750E-20 | 1.01 [0.98, 1.04] | 4.505E-01 | Mental and Nervous | ebi-a-GCST005531 |
| Diabetes mellitus | DM | 0.69 [0.65, 0.74] | 2.000E-24 | 0.90 [0.79, 1.03] | 1.170E-01 | 0.85 [0.79, 0.93] | 1.579E-04 | Endocrine, nutritional and metabolic | finn-b-E4_DIABETES |
| Primary biliary cholangitis | PBC_S1 | 1.15 [1.12, 1.19] | 2.360E-22 | 1.15 [1.11, 1.19] | 1.853E-15 | 1.11 [1.08, 1.14] | 8.809E-13 | Digestive | ebi-a-GCST003129 |
| Diabetes, insuline treatment (Kela reimbursement) (more controls excluded) | KELA_DIAB_INSUL_EXMORE | 0.76 [0.71, 0.80] | 3.590E-22 | 0.87 [0.79, 0.97] | 8.067E-03 | 0.90 [0.84, 0.96] | 1.496E-03 | Endocrine, nutritional and metabolic | finn-b-KELA_DIAB_INSUL_EXMORE |
| Celiac disease | CeD_S3 | 0.96 [0.95, 0.96] | 1.280E-21 | 0.98 [0.97, 0.99] | 2.658E-03 | 0.92 [0.91, 0.93] | 2.231E-41 | Digestive | ieu-a-1058 |
| Blood clot DVT bronchitis emphysema asthma rhinitis eczema allergy diagnosed by doctor: None of | Diagnosis_3 | 0.05 [0.03, 0.10] | 1.610E-19 | 0.19 [0.10, 0.37] | 1.063E-06 | 0.00 [0.00, 0.01] | 1.373E-55 | Skin | ukb-a-442 |
| Systemic lupus erythematosus | SLE_S1 | 0.91 [0.89, 0.93] | 3.090E-17 | 0.89 [0.87, 0.92] | 7.077E-15 | 0.96 [0.94, 0.99] | 1.938E-03 | Musculoskeletal | ebi-a-GCST003156 |
| Asthma-related infections | Asthma_INFECTIONS | 0.40 [0.32, 0.50] | 1.110E-15 | 0.44 [0.35, 0.56] | 5.270E-12 | 0.39 [0.31, 0.50] | 8.805E-14 | Respiratory | finn-b-ASTHMA_INFECTIONS |
| Atopic dermatitis | Atopic dermatitis | 1.52 [1.37, 1.70] | 2.490E-14 | 1.26 [1.13, 1.41] | 4.936E-05 | 1.81 [1.57, 2.08] | 2.148E-16 | Skin | finn-b-L12_ATOPIC |
| Type 1 diabetes without complications | T1DNOCOMP | 0.91 [0.88, 0.93] | 4.890E-14 | 1.01 [0.98, 1.04] | 4.701E-01 | 0.89 [0.86, 0.92] | 2.090E-12 | Endocrine, nutritional and metabolic | finn-b-E4_DM1NOCOMP |
| Type 1 diabetes | T1D_S3 | 0.94 [0.92, 0.95] | 1.410E-13 | 1.02 [0.99, 1.05] | 1.334E-01 | 0.92 [0.89, 0.95] | 3.142E-07 | Endocrine, nutritional and metabolic | ebi-a-GCST90000529 |
| Celiac disease | CeD_S5 | 1.18 [1.12, 1.23] | 1.120E-12 | 1.12 [1.06, 1.19] | 5.594E-05 | 1.14 [1.08, 1.20] | 1.683E-06 | Digestive | ieu-a-1060 |
| Type 1 diabetes, strict definition | T1D_STRICT | 0.93 [0.91, 0.95] | 1.830E-11 | 0.99 [0.96, 1.03] | 6.223E-01 | 0.93 [0.91, 0.96] | 5.966E-08 | Endocrine, nutritional and metabolic | finn-b-T1D_STRICT |
| Type 1 diabetes, wide definition, subgroup 1 | T1D_wide | 0.91 [0.89, 0.94] | 3.060E-11 | 1.00 [0.98, 1.03] | 7.818E-01 | 0.96 [0.94, 0.99] | 1.741E-03 | Endocrine, nutritional and metabolic | finn-b-T1D_WIDE1 |
| Celiac disease | CeD_S2 | 1.06 [1.04, 1.08] | 3.560E-11 | 1.08 [1.06, 1.10] | 6.591E-18 | 1.03 [1.02, 1.05] | 6.649E-06 | Digestive | ebi-a-GCST005523 |
| Multiple sclerosis | MS_S3 | 1.09 [1.06, 1.11] | 1.320E-10 | 0.98 [0.94, 1.02] | 2.444E-01 | 0.94 [0.91, 0.97] | 7.688E-05 | Mental and Nervous | ieu-a-1024 |
| Primary biliary cirrhosis | PBC_S2 | 1.08 [1.06, 1.11] | 1.710E-10 | 1.04 [1.02, 1.07] | 1.995E-03 | 1.08 [1.05, 1.11] | 3.024E-08 | Digestive | ebi-a-GCST005581 |
| Type 1 diabetes | T1D_S2 | 0.92 [0.90, 0.95] | 6.990E-10 | 0.99 [0.97, 1.01] | 4.482E-01 | 0.93 [0.90, 0.95] | 1.718E-10 | Endocrine, nutritional and metabolic | ebi-a-GCST010681 |
| Diabetes diagnosed by doctor | Diabetes_diagnosed | 0.00 [0.00, 0.01] | 1.320E-09 | 0.01 [0.00, 0.17] | 7.673E-04 | 0.00 [0.00, 0.00] | 3.207E-15 | Endocrine, nutritional and metabolic | ukb-a-306 |
| Ankylosing spondylitis | AS | 1.41 [1.25, 1.59] | 1.680E-08 | 1.38 [1.22, 1.56] | 2.934E-07 | 1.04 [0.90, 1.19] | 5.986E-01 | Musculoskeletal | ebi-a-GCST005529 |
| Rheumatoid arthritis | RA_S4 | 1.13 [1.08, 1.18] | 4.610E-08 | 1.10 [1.06, 1.15] | 2.381E-06 | 1.13 [1.07, 1.18] | 2.536E-06 | Musculoskeletal | ieu-a-832 |
| Type 1 diabetes | T1D_S4 | 0.95 [0.93, 0.97] | 4.130E-07 | 1.01 [0.98, 1.04] | 4.630E-01 | 0.95 [0.92, 0.97] | 6.276E-05 | Endocrine, nutritional and metabolic | finn-b-E4_DM1 |
| Allergic disease (asthma, hay fever or eczema) | Allergic disease | 1.79 [1.41, 2.26] | 1.350E-06 | 2.10 [1.63, 2.71] | 8.663E-09 | 3.21 [2.54, 4.05] | 1.215E-22 | Respiratory | ebi-a-GCST005038 |
| Diagnoses - secondary ICD10: J45.9 Asthma, | Asthma_unspecified | 0.00 [0.00, 0.00] | 1.390E-06 | 0.00 [0.00, 0.00] | 2.278E-05 | 0.00 [0.00, 0.01] | 2.630E-05 | Respiratory | ukb-b-17219 |
| Hypothyroidism, drug reimbursement | HYPOTHY_REIMB | 0.89 [0.85, 0.94] | 8.360E-06 | 1.05 [0.95, 1.16] | 3.485E-01 | 0.73 [0.69, 0.78] | 5.473E-20 | Endocrine, nutritional and metabolic | finn-b-HYPOTHY_REIMB |
| Type 1 diabetes | T1D_S1 | 1.05 [1.03, 1.07] | 2.440E-05 | 1.04 [1.01, 1.06] | 7.211E-03 | 1.14 [1.10, 1.19] | 4.324E-13 | Endocrine, nutritional and metabolic | ebi-a-GCST005536 |
| Psoriasis vulgaris | Psori_vulg | 1.07 [1.04, 1.11] | 2.620E-05 | 1.32 [1.22, 1.44] | 1.938E-11 | 1.09 [1.05, 1.13] | 1.854E-05 | Skin | finn-b-L12_PSORI_VULG |
| Coronary artery disease | CAD | 1.40 [1.19, 1.64] | 3.930E-05 | 1.42 [1.17, 1.73] | 4.036E-04 | 1.41 [1.16, 1.71] | 4.767E-04 | Blood and circulatory | ebi-a-GCST003116 |
| Vascular/heart problems diagnosed by doctor: High blood pressure | Hypertension | 0.10 [0.03, 0.31] | 4.370E-05 | 0.19 [0.06, 0.59] | 4.125E-03 | 0.91 [0.31, 2.65] | 8.618E-01 | Blood and circulatory | ukb-b-14177 |
| Asthma (childhood onset) | Asthma (childhood onset) | 0.98 [0.98, 0.99] | 5.430E-05 | 0.95 [0.95, 0.96] | 7.357E-40 | 0.95 [0.94, 0.96] | 4.197E-39 | Respiratory | ebi-a-GCST007800 |
| Systemic lupus erythematosus | SLE_S2 | 0.92 [0.88, 0.96] | 8.870E-05 | 0.93 [0.89, 0.98] | 5.418E-03 | 0.91 [0.87, 0.96] | 1.625E-04 | Musculoskeletal | ieu-a-815 |
| Rheumatoid Arthritis | RA_S6 | 1.07 [1.03, 1.11] | 3.974E-04 | 1.09 [1.05, 1.13] | 8.742E-06 | 0.89 [0.85, 0.92] | 7.546E-09 | Musculoskeletal | bbj-a-73 |
| Blood clot DVT bronchitis emphysema asthma rhinitis eczema allergy diagnosed by doctor: Hayfever allergic rhinitis or eczema | Diagnosis_2 | 12.53 [4.05, 38.71] | 7.293E-04 | 3.65 [1.75, 7.61] | 5.396E-04 | 14.54 [3.93, 53.79] | 2.052E-03 | Skin | ukb-a-447 |
| Disorders of the thyroid gland | Disorders_2 | 0.86 [0.79, 0.94] | 1.050E-03 | 1.17 [1.05, 1.31] | 5.434E-03 | 0.65 [0.59, 0.73] | 3.180E-14 | Endocrine, nutritional and metabolic | finn-b-E4_THYROID |
| Hypothyroidism (congenital or acquired) | HYPOTHY | 0.89 [0.83, 0.96] | 1.696E-03 | 1.14 [1.04, 1.24] | 5.351E-03 | 0.70 [0.64, 0.77] | 3.098E-14 | Endocrine, nutritional and metabolic | finn-b-HYPOTHYROIDISM |
| Diagnoses - main ICD10: D22 Melanocytic naevi | Melanocytic naevi | 0.00 [0.00, 0.00] | 1.828E-03 | 0.00 [0.00, 0.00] | 1.678E-04 | 0.00 [0.00, 0.09] | 1.872E-02 | Neoplasms | ukb-d-D22 |
| Celiac disease | CeD_S1 | 1.07 [1.03, 1.12] | 2.026E-03 | 1.22 [1.15, 1.29] | 3.949E-11 | 1.14 [1.07, 1.20] | 7.445E-06 | Digestive | ebi-a-GCST000612 |
| Celiac disease | CeD_S6 | 1.07 [1.03, 1.12] | 2.030E-03 | 1.05 [1.00, 1.10] | 5.366E-02 | 1.14 [1.07, 1.20] | 7.615E-06 | Digestive | ieu-a-276 |
| Thyrotoxicosis with diffuse goitre | THYTOXGOITDIF | 0.95 [0.92, 0.98] | 2.384E-03 | 1.02 [0.98, 1.07] | 3.112E-01 | 0.91 [0.87, 0.96] | 1.561E-04 | Endocrine, nutritional and metabolic | finn-b-E4_THYTOXGOITDIF |

| | | | | | | | | | |
|---|---|---|---|---|---|---|---|---|---|
| Hypothyroidism, levothyroxin purchases | HYPOTHY_LEVOTHY | 0.90 [0.84, 0.96] | 3.108E-03 | 1.14 [1.04, 1.24] | 5.351E-03 | 0.73 [0.67, 0.80] | 1.539E-11 | Endocrine, nutritional and metabolic | finn-b-HYPOTHY_LEVOTHY |
| Asthma | Asthma_meta | 0.87 [0.79, 0.96] | 5.191E-03 | 0.85 [0.75, 0.95] | 6.427E-03 | 0.86 [0.77, 0.97] | 1.524E-02 | Respiratory | ebi-a-GCST006862 |
| Myocardial infarction | Asthma_unspecified | 1.23 [1.06, 1.44] | 6.569E-03 | 1.41 [1.20, 1.67] | 4.487E-05 | 1.21 [1.01, 1.46] | 3.804E-02 | Blood and circulatory | ebi-a-GCST011365 |
| Gastroesophageal reflux disease | GERD | 0.69 [0.52, 0.90] | 7.075E-03 | 0.93 [0.69, 1.27] | 6.585E-01 | 0.53 [0.41, 0.69] | 1.751E-06 | Digestive | ebi-a-GCST90000514 |
| Coronary atherosclerosis | CORATHER | 1.28 [1.07, 1.53] | 7.604E-03 | 1.28 [1.03, 1.59] | 2.578E-02 | 1.01 [0.77, 1.32] | 9.565E-01 | Blood and circulatory | finn-b-I9_CORATHER |
| Diabetes, varying definitions | Diabetes_FG | 0.88 [0.79, 0.97] | 1.094E-02 | 0.90 [0.79, 1.02] | 1.068E-01 | 0.81 [0.74, 0.88] | 7.667E-07 | Endocrine, nutritional and metabolic | finn-b-DIABETES_FG |
| Autoimmune diseases | Autoimmune diseases | 1.10 [1.02, 1.18] | 1.222E-02 | 1.21 [1.11, 1.31] | 9.127E-06 | 0.68 [0.63, 0.74] | 5.498E-19 | Blood and circulatory | finn-b-AUTOIMMUNE |
| Coronary atherosclerosis (no controls excluded) | CORATHER_EXNONE | 1.29 [1.06, 1.57] | 1.228E-02 | 1.30 [1.03, 1.66] | 3.027E-02 | 1.01 [0.75, 1.35] | 9.720E-01 | Blood and circulatory | finn-b-I9_CORATHER_EXNONE |
| Type 2 diabetes, definitions combined, including | T2D_INCLAVO | 0.86 [0.76, 0.97] | 1.241E-02 | 0.93 [0.80, 1.08] | 3.323E-01 | 0.82 [0.71, 0.95] | 9.433E-03 | Endocrine, nutritional and metabolic | finn-b-T2D_INCLAVO |
| Disorders of gallbladder, biliary tract and pancreas | Disorders_3 | 0.00 [0.00, 0.27] | 1.420E-02 | 0.00 [0.00, 0.00] | 5.688E-05 | 0.11 [0.00, 44.41] | 4.685E-01 | Digestive | ukb-d-K11_GALLBILPANC |
| Vascular/heart problems diagnosed by doctor: None of the above | Diagnosis_1 | 2.79 [1.21, 6.42] | 1.612E-02 | 2.00 [0.74, 5.42] | 1.721E-01 | 1.15 [0.44, 3.03] | 7.785E-01 | Blood and circulatory | ukb-b-13352 |
| Diabetes, insuline treatment (Kela reimbursement) | KELA_DIAB_INSUL | 0.91 [0.84, 0.98] | 1.679E-02 | 0.85 [0.77, 0.94] | 1.438E-03 | 0.88 [0.83, 0.94] | 2.733E-04 | Endocrine, nutritional and metabolic | finn-b-KELA_DIAB_INSUL |
| Type 2 diabetes | T2D_S3 | 0.88 [0.79, 0.98] | 1.804E-02 | 0.88 [0.79, 0.98] | 1.716E-02 | 0.94 [0.83, 1.06] | 3.211E-01 | Endocrine, nutritional and metabolic | ebi-a-GCST007517 |
| Ischaemic heart disease, wide definition | IHD | 1.22 [1.03, 1.45] | 2.221E-02 | 1.21 [0.98, 1.49] | 8.089E-02 | 1.38 [1.15, 1.67] | 7.501E-04 | Blood and circulatory | finn-b-I9_IHD |
| Major depression | Major depression | 0.61 [0.40, 0.93] | 2.270E-02 | 1.42 [0.94, 2.12] | 9.278E-02 | 0.58 [0.35, 0.95] | 2.903E-02 | Mental and Nervous | ieu-b-102 |
| Nasal polyp | Nasal polyp | 0.90 [0.83, 0.99] | 2.393E-02 | 0.97 [0.87, 1.07] | 5.141E-01 | 0.81 [0.73, 0.91] | 2.951E-04 | Respiratory | finn-b-J10_NASALPOLYP |
| Rheumatoid arthritis | RA_S2 | 0.97 [0.93, 1.00] | 3.740E-02 | 1.09 [1.04, 1.13] | 3.777E-05 | 0.85 [0.82, 0.89] | 1.090E-13 | Musculoskeletal | ebi-a-GCST002318 |
| Other diabetes, wide definition | Diabetes_wide | 0.90 [0.82, 1.00] | 3.924E-02 | 0.93 [0.83, 1.05] | 2.570E-01 | 0.88 [0.82, 0.96] | 2.368E-03 | Endocrine, nutritional and metabolic | finn-b-DM_OTHER_WIDE |
| Mouth/teeth dental problems: Mouth ulcers | Asthma_diagnosed | 2.52 [1.05, 6.05] | 3.934E-02 | 6.61 [2.21, 19.74] | 7.273E-04 | 0.65 [0.22, 1.90] | 4.292E-01 | Digestive | ukb-a-427 |
| Type 2 diabetes, strict (exclude DM1) | T2D_STRICT | 0.87 [0.76, 1.00] | 4.219E-02 | 0.87 [0.75, 1.02] | 7.903E-02 | 0.85 [0.73, 1.00] | 5.268E-02 | Endocrine, nutritional and metabolic | finn-b-E4_DM2_STRICT |
| Type 2 diabetes, definitions combined | T2D_S5 | 0.87 [0.76, 1.00] | 4.245E-02 | 0.93 [0.79, 1.10] | 3.972E-01 | 0.85 [0.73, 1.00] | 5.334E-02 | Endocrine, nutritional and metabolic | finn-b-T2D |
| Pure hypercholesterolaemia | FH_Finn | 1.08 [1.00, 1.17] | 5.580E-02 | 1.09 [0.98, 1.21] | 9.749E-02 | 1.08 [0.98, 1.19] | 1.224E-01 | Endocrine, nutritional and metabolic | finn-b-E4_HYPERCHOL |
| Celiac disease | CeD_S4 | 1.04 [1.00, 1.09] | 6.244E-02 | 1.03 [0.98, 1.08] | 3.110E-01 | 1.10 [1.04, 1.17] | 8.547E-04 | Digestive | ieu-a-1059 |
| Basal cell carcinoma | BCC | 1.09 [0.99, 1.20] | 7.574E-02 | 1.04 [0.94, 1.16] | 4.488E-01 | 1.11 [0.99, 1.25] | 7.136E-02 | Neoplasms | ebi-a-GCST90013410 |
| Diagnoses - main ICD10: D12 Benign neoplasm of colon, rectum, anus and anal canal | Digestive Neoplasms | 0.03 [0.00, 1.66] | 8.687E-02 | 0.06 [0.00, 8.59] | 2.664E-01 | 0.14 [0.00, 16.43] | 4.139E-01 | Neoplasms | ukb-d-D12 |
| Colorectal cancer | Colorectal cancer | 0.01 [0.00, 2.09] | 9.296E-02 | 0.02 [0.00, 9.48] | 2.028E-01 | 0.06 [0.00, 28.22] | 3.656E-01 | Digestive | ieu-b-4965 |
| Diagnoses - secondary ICD10: E78.0 Pure hypercholesterolaemia | FH_UKB | 6.44 [0.72, 58.07] | 9.667E-02 | 31.32 [2.38, 412.08] | 8.803E-03 | 4.49 [0.32, 63.37] | 2.659E-01 | Endocrine, nutritional and metabolic | ukb-b-12651 |
| Diagnoses - secondary ICD10: E11.9 Without | T2D_nocom | 0.08 [0.00, 1.67] | 1.029E-01 | 0.00 [0.00, 0.14] | 2.597E-03 | 1.03 [0.03, 40.91] | 9.855E-01 | Endocrine, nutritional and metabolic | ukb-b-970 |
| Hypothyroidism, strict autoimmune, 3 medication purchases required | HYTHY_AI_STRICT_PURCH | 1.07 [0.99, 1.16] | 1.077E-01 | 1.14 [1.04, 1.24] | 3.594E-03 | 0.77 [0.69, 0.86] | 2.701E-06 | Endocrine, nutritional and metabolic | finn-b-E4_HYTHY_AI_STRICT_PURCH |
| Disorders of lipoprotein metabolism and other | Disorders_1 | 1.07 [0.98, 1.16] | 1.124E-01 | 1.07 [0.96, 1.19] | 1.956E-01 | 1.06 [0.96, 1.18] | 2.236E-01 | Endocrine, nutritional and metabolic | finn-b-E4_LIPOPROT |
| Haemorrhoidal disease | HD | 1.26 [0.94, 1.68] | 1.200E-01 | 2.64 [1.93, 3.63] | 1.742E-09 | 0.72 [0.51, 1.02] | 6.729E-02 | Digestive | ebi-a-GCST90014033 |
| Myocardial infarction | Allergic disease | 1.15 [0.96, 1.38] | 1.275E-01 | 1.41 [1.16, 1.71] | 4.520E-04 | 1.08 [0.87, 1.34] | 5.032E-01 | Blood and circulatory | ebi-a-GCST011364 |
| Parkinson's disease | PD | 1.08 [0.98, 1.20] | 1.321E-01 | 1.13 [0.99, 1.29] | 6.057E-02 | 1.04 [0.92, 1.18] | 5.293E-01 | Mental and Nervous | ieu-b-7 |
| Coronary heart disease | CHD | 1.10 [0.97, 1.25] | 1.383E-01 | 1.13 [0.96, 1.32] | 1.423E-01 | 1.04 [0.89, 1.21] | 6.084E-01 | Blood and circulatory | ieu-a-8 |
| Prostate cancer | Prostate cancer | 0.94 [0.86, 1.02] | 1.460E-01 | 0.95 [0.86, 1.04] | 2.769E-01 | 0.97 [0.88, 1.08] | 6.216E-01 | Neoplasms | ebi-a-GCST006085 |
| Rheumatoid arthritis | RA_S1 | 0.98 [0.96, 1.01] | 1.955E-01 | 1.17 [1.13, 1.21] | 1.835E-18 | 0.83 [0.80, 0.85] | 6.117E-34 | Musculoskeletal | ebi-a-GCST000679 |
| Asthma (adult onset) | Asthma (adult onset) | 0.99 [0.98, 1.00] | 1.957E-01 | 1.03 [1.02, 1.05] | 3.643E-06 | 0.92 [0.91, 0.93] | 4.765E-33 | Respiratory | ebi-a-GCST007799 |
| Cancer of skin | Cancer of skin | 6.90 [0.95, 49.88] | 1.958E-01 | 2.54 [0.15, 41.86] | 5.143E-01 | 40.15 [1.20, 1342.63] | 1.753E-01 | Neoplasms | ukb-d-C_SKIN |
| Type 2 diabetes | T2D_S4 | 0.93 [0.82, 1.04] | 1.983E-01 | 0.88 [0.77, 1.01] | 6.841E-02 | 0.81 [0.69, 0.95] | 9.598E-03 | Endocrine, nutritional and metabolic | finn-b-E4_DM2 |
| Thyroid cancer | Thyroid cancer | 1.00 [1.00, 1.01] | 2.108E-01 | 1.00 [1.00, 1.01] | 1.418E-01 | 1.00 [1.00, 1.01] | 1.756E-01 | Endocrine, nutritional and metabolic | ieu-a-1082 |
| Type 2 diabetes with other specified/multiple/unspecified complications | T2D_NASCOMP | 0.91 [0.78, 1.06] | 2.144E-01 | 0.93 [0.79, 1.10] | 3.997E-01 | 0.83 [0.72, 0.97] | 1.536E-02 | Endocrine, nutritional and metabolic | finn-b-E4_DM2NASCOMP |
| Hypothyroidism, strict autoimmune | HYTHY_AI_STRICT | 1.05 [0.97, 1.14] | 2.403E-01 | 1.12 [1.03, 1.21] | 1.011E-02 | 0.81 [0.73, 0.89] | 2.161E-05 | Endocrine, nutritional and metabolic | finn-b-E4_HYTHY_AI_STRICT |
| Type 2 diabetes | T2D_S1 | 0.95 [0.86, 1.04] | 2.634E-01 | 0.95 [0.85, 1.06] | 3.469E-01 | 0.96 [0.86, 1.07] | 4.690E-01 | Endocrine, nutritional and metabolic | ebi-a-GCST005047 |
| Psoriasis | Psoriasis | 1.01 [0.99, 1.02] | 2.720E-01 | 0.98 [0.97, 1.00] | 6.202E-02 | 1.09 [1.07, 1.11] | 6.365E-22 | Skin | ebi-a-GCST005527 |
| Breast cancer | Breast cancer_S1 | 0.91 [0.76, 1.10] | 3.324E-01 | 1.06 [0.87, 1.31] | 5.485E-01 | 1.62 [1.26, 2.08] | 1.592E-04 | Neoplasms | ebi-a-GCST004988 |
| multiple sclerosis | MS_S4 | 0.99 [0.96, 1.01] | 3.577E-01 | 1.17 [1.13, 1.21] | 4.169E-18 | 1.00 [0.97, 1.04] | 9.345E-01 | Mental and Nervous | ieu-b-18 |
| Bipolar disorder | Bipolar disorder | 0.96 [0.88, 1.05] | 3.745E-01 | 1.00 [0.90, 1.11] | 9.933E-01 | 0.93 [0.84, 1.03] | 1.523E-01 | Mental and Nervous | ieu-a-800 |
| Narcolepsy | Narcolepsy | 1.03 [0.97, 1.09] | 3.802E-01 | 1.00 [0.93, 1.08] | 9.376E-01 | 1.05 [0.97, 1.13] | 2.279E-01 | Mental and Nervous | ebi-a-GCST005522 |
| schizophrenia | Schizophrenia | 1.05 [0.94, 1.16] | 3.901E-01 | 1.04 [0.92, 1.18] | 5.482E-01 | 1.06 [0.94, 1.20] | 3.679E-01 | Mental and Nervous | ieu-b-42 |

| | | | | | | | | | |
|---|---|---|---|---|---|---|---|---|---|
| Hypothyroidism, other/unspecified | HYTHYNAS | 1.03 [0.95, 1.12] | 4.753E-01 | 1.14 [1.05, 1.24] | 1.684E-03 | 0.79 [0.71, 0.88] | 1.838E-05 | Endocrine, nutritional and metabolic | finn-b-E4_HYTHYNAS |
| Rheumatoid arthritis | RA_S3 | 1.00 [0.97, 1.02] | 7.660E-01 | 1.08 [1.05, 1.11] | 4.573E-07 | 0.85 [0.82, 0.89] | 6.439E-17 | Musculoskeletal | ebi-a-GCST005569 |
| Rheumatoid arthritis | RA_S5 | 1.00 [0.98, 1.02] | 8.795E-01 | 1.17 [1.13, 1.21] | 1.959E-18 | 0.83 [0.80, 0.86] | 2.957E-30 | Musculoskeletal | ieu-a-834 |
| Type 2 diabetes | T2D_S2 | 1.00 [0.94, 1.08] | 8.869E-01 | 0.89 [0.83, 0.97] | 6.933E-03 | 0.90 [0.81, 0.99] | 2.503E-02 | Endocrine, nutritional and metabolic | ebi-a-GCST006867 |
| Type 2 diabetes (adjusted for BMI) | T2D_adjusted | 1.00 [0.93, 1.08] | 9.259E-01 | 0.98 [0.91, 1.07] | 7.196E-01 | 1.13 [1.04, 1.23] | 3.311E-03 | Endocrine, nutritional and metabolic | ebi-a-GCST007518 |
| Type 2 diabetes | T2D_S6 | 1.00 [0.93, 1.08] | 9.263E-01 | 0.97 [0.89, 1.06] | 4.608E-01 | 1.10 [1.01, 1.20] | 2.898E-02 | Endocrine, nutritional and metabolic | ebi-a-GCST007515 |
| Blood clot DVT bronchitis emphysema asthma rhinitis eczema allergy diagnosed by doctor: Asthma | Diagnosis_1 | 0.98 [0.34, 2.81] | 9.640E-01 | 3.96 [1.36, 11.48] | 1.135E-02 | 0.38 [0.10, 1.49] | 1.653E-01 | Respiratory | ukb-a-446 |
| Breast cancer | Breast cancer_S2 | 1.00 [0.99, 1.01] | 9.716E-01 | 1.00 [0.99, 1.01] | 6.417E-01 | 1.00 [0.99, 1.01] | 9.749E-01 | Neoplasms | ebi-a-GCST007236 |
| Doctor diagnosed asthma | Asthma_diagnosed | 1.01 [0.37, 2.82] | 9.778E-01 | 0.99 [0.27, 3.60] | 9.937E-01 | 0.62 [0.18, 2.15] | 4.521E-01 | Respiratory | ukb-a-255 |
| Osteoarthritis of the hip or knee | OA of hip/knee | 1.00 [0.77, 1.30] | 9.943E-01 | 1.06 [0.76, 1.46] | 7.462E-01 | 0.99 [0.72, 1.36] | 0.9487527 | Musculoskeletal | ebi-a-GCST007092 |

**Supplementary Table 3. Disease-wide MR analysis results in reverse direction**

| | | IBD | | UC | | CD | | | |
|---|---|---|---|---|---|---|---|---|---|
| Trait | Name | OR [95%CI] | pval | OR [95%CI] | pval | OR [95%CI] | pval | Category | GWAS-ID |
| Diabetic hypoglycemia | Diabetic hypoglycemia | 0.98 [0.94, 1.03] | 4.179E-01 | 1.00 [0.96, 1.05] | 9.952E-01 | 1.00 [0.96, 1.04] | 8.908E-01 | Endocrine, nutritional and metabolic | finn-b-DM_HYPOGLYC |
| Asthma | Asthma_UKB | 1.00 [0.99, 1.01] | 8.595E-01 | 1.01 [1.00, 1.03] | 4.714E-02 | 1.03 [1.01, 1.04] | 4.371E-06 | Respiratory | ebi-a-GCST90014325 |
| Type 1 diabetes with coma | T1D_COMA | 0.97 [0.90, 1.05] | 4.741E-01 | 1.00 [0.92, 1.08] | 9.523E-01 | 1.00 [0.94, 1.07] | 9.631E-01 | Endocrine, nutritional and metabolic | finn-b-E4_DM1COMA |
| Diabetes, several complications | DM_SEVERAL_COMPLICATIONS | 1.00 [0.96, 1.03] | 8.090E-01 | 0.99 [0.95, 1.03] | 5.695E-01 | 1.01 [0.98, 1.04] | 6.760E-01 | Endocrine, nutritional and metabolic | finn-b-DM_SEVERAL_COMPLICATIONS |
| Other diabetic retinopathy | RETINOPATHYDIAB_NAS | 0.95 [0.88, 1.03] | 2.274E-01 | 0.93 [0.85, 1.01] | 8.512E-02 | 0.98 [0.91, 1.05] | 5.885E-01 | Endocrine, nutritional and metabolic | finn-b-H7_RETINOPATHYDIAB_NAS |
| Multiple sclerosis | MS_S1 | 1.09 [1.06, 1.12] | 7.906E-08 | 1.07 [1.03, 1.10] | 1.810E-04 | 1.10 [1.07, 1.13] | 8.476E-11 | Mental and Nervous | ieu-a-1025 |
| Multiple sclerosis | MS_S2 | 1.09 [1.05, 1.12] | 1.697E-07 | 1.07 [1.03, 1.10] | 1.908E-04 | 1.10 [1.07, 1.13] | 1.251E-10 | Mental and Nervous | ebi-a-GCST005531 |
| Diabetes mellitus | DM | 0.99 [0.97, 1.01] | 2.900E-01 | 0.99 [0.97, 1.00] | 1.420E-01 | 1.01 [0.99, 1.02] | 2.983E-01 | Endocrine, nutritional and metabolic | finn-b-E4_DIABETES |
| Primary biliary cholangitis | PBC_S1 | 1.24 [1.14, 1.35] | 6.449E-07 | 1.09 [0.99, 1.19] | 6.979E-02 | 1.12 [1.05, 1.20] | 6.510E-04 | Digestive | ebi-a-GCST003129 |
| Diabetes, insuline treatment (Kela reimbursement) (more controls excluded) | KELA_DIAB_INSUL_EXMORE | 0.99 [0.98, 1.01] | 5.013E-01 | 0.98 [0.96, 1.00] | 8.896E-02 | 1.01 [0.99, 1.03] | 2.836E-01 | Endocrine, nutritional and metabolic | finn-b-KELA_DIAB_INSUL_EXMORE |
| Celiac disease | CeD_S3 | 1.20 [1.09, 1.32] | 2.128E-04 | 1.07 [0.98, 1.17] | 1.109E-01 | 1.26 [1.16, 1.37] | 9.187E-08 | Digestive | ieu-a-1058 |
| Blood clot DVT bronchitis emphysema asthma rhinitis eczema allergy diagnosed by doctor: None of | Diagnosis_3 | 1.00 [0.99, 1.00] | 5.958E-04 | 1.00 [1.00, 1.00] | 1.857E-01 | 0.99 [0.99, 0.99] | 2.522E-14 | Skin | ukb-a-442 |
| Systemic lupus erythematosus | SLE_S1 | 1.07 [1.01, 1.13] | 1.431E-02 | 1.06 [1.00, 1.12] | 4.213E-02 | 0.98 [0.93, 1.02] | 2.998E-01 | Musculoskeletal | ebi-a-GCST003156 |
| Asthma-related infections | Asthma_INFECTIONS | 1.03 [1.00, 1.06] | 4.213E-02 | 1.01 [0.98, 1.04] | 3.828E-01 | 1.03 [1.01, 1.06] | 8.727E-03 | Respiratory | finn-b-ASTHMA_INFECTIONS |
| Atopic dermatitis | Atopic dermatitis | 1.05 [1.02, 1.08] | 1.883E-03 | 1.02 [0.99, 1.05] | 2.422E-01 | 1.04 [1.01, 1.07] | 1.174E-02 | Skin | finn-b-L12_ATOPIC |
| Type 1 diabetes without complications | T1DNOCOMP | 1.01 [0.97, 1.06] | 5.284E-01 | 1.02 [0.97, 1.06] | 5.028E-01 | 1.01 [0.98, 1.05] | 5.425E-01 | Endocrine, nutritional and metabolic | finn-b-E4_DM1NOCOMP |
| Type 1 diabetes | T1D_S3 | 1.07 [1.03, 1.12] | 4.825E-04 | 1.04 [1.00, 1.09] | 6.163E-02 | 1.01 [0.98, 1.05] | 5.345E-01 | Endocrine, nutritional and metabolic | ebi-a-GCST90000529 |
| Celiac disease | CeD_S5 | 1.42 [1.18, 1.69] | 1.496E-04 | 1.40 [1.18, 1.67] | 1.283E-04 | 1.11 [0.93, 1.33] | 2.275E-01 | Digestive | ieu-a-1060 |
| Type 1 diabetes, strict definition | T1D_STRICT | 0.99 [0.94, 1.05] | 8.037E-01 | 1.01 [0.95, 1.07] | 6.982E-01 | 1.00 [0.95, 1.05] | 8.620E-01 | Endocrine, nutritional and metabolic | finn-b-T1D_STRICT |
| Type 1 diabetes, wide definition, subgroup 1 | T1D_wide | 1.00 [0.96, 1.04] | 8.879E-01 | 1.00 [0.96, 1.05] | 9.237E-01 | 1.02 [0.98, 1.05] | 3.746E-01 | Endocrine, nutritional and metabolic | finn-b-T1D_WIDE1 |
| Celiac disease | CeD_S2 | NA | NA | NA | NA | NA | NA | Digestive | ebi-a-GCST005523 |
| Multiple sclerosis | MS_S3 | 1.12 [1.05, 1.20] | 1.279E-03 | 1.13 [1.06, 1.20] | 1.671E-04 | 1.04 [0.99, 1.09] | 1.546E-01 | Mental and Nervous | ieu-a-1024 |
| Primary biliary cirrhosis | PBC_S2 | NA | NA | NA | NA | NA | NA | Digestive | ebi-a-GCST005581 |
| Type 1 diabetes | T1D_S2 | 1.02 [0.98, 1.07] | 3.343E-01 | 1.02 [0.98, 1.07] | 3.559E-01 | 0.99 [0.95, 1.02] | 4.303E-01 | Endocrine, nutritional and metabolic | ebi-a-GCST010681 |
| Diabetes diagnosed by doctor | Diabetes_diagnosed | 1.00 [1.00, 1.00] | 3.743E-01 | 1.00 [1.00, 1.00] | 4.277E-01 | 1.00 [1.00, 1.00] | 1.965E-02 | Endocrine, nutritional and metabolic | ukb-a-306 |
| Ankylosing spondylitis | AS | NA | NA | NA | NA | NA | NA | Musculoskeletal | ebi-a-GCST005529 |
| Rheumatoid arthritis | RA_S4 | 1.01 [0.98, 1.04] | 4.259E-01 | 1.03 [1.00, 1.07] | 2.944E-02 | 0.97 [0.94, 0.99] | 1.435E-02 | Musculoskeletal | ieu-a-832 |
| Type 1 diabetes | T1D_S4 | 0.99 [0.96, 1.03] | 6.570E-01 | 1.01 [0.97, 1.05] | 6.427E-01 | 1.01 [0.97, 1.04] | 6.779E-01 | Endocrine, nutritional and metabolic | finn-b-E4_DM1 |
| Allergic disease (asthma, hay fever or eczema) | Allergic disease | 1.01 [1.00, 1.02] | 8.263E-02 | 1.00 [0.99, 1.01] | 6.658E-01 | 1.02 [1.01, 1.03] | 7.000E-05 | Respiratory | ebi-a-GCST005038 |
| Diagnoses - secondary ICD10: J45.9 Asthma, | Asthma_unspecified | 1.00 [1.00, 1.00] | 3.964E-01 | 1.00 [1.00, 1.00] | 4.786E-02 | 1.00 [1.00, 1.00] | 1.069E-02 | Respiratory | ukb-b-17219 |
| Hypothyroidism, drug reimbursement | HYPOTHY_REIMB | 1.04 [1.00, 1.08] | 4.087E-02 | 1.04 [1.00, 1.09] | 3.059E-02 | 1.01 [0.98, 1.05] | 4.418E-01 | Endocrine, nutritional and metabolic | finn-b-HYPOTHY_REIMB |
| Type 1 diabetes | T1D_S1 | NA | NA | NA | NA | NA | NA | Endocrine, nutritional and metabolic | ebi-a-GCST005536 |
| Psoriasis vulgaris | Psori_vulg | 1.14 [1.08, 1.19] | 3.964E-07 | 1.12 [1.06, 1.18] | 2.771E-05 | 1.08 [1.03, 1.13] | 5.278E-04 | Skin | finn-b-L12_PSORI_VULG |
| Coronary artery disease | CAD | 1.01 [0.99, 1.02] | 5.266E-01 | 1.00 [0.98, 1.01] | 6.805E-01 | 1.00 [0.98, 1.01] | 6.979E-01 | Blood and circulatory | ebi-a-GCST003116 |
| Vascular/heart problems diagnosed by doctor: High blood pressure | Hypertension | 1.00 [1.00, 1.00] | 1.970E-03 | 1.00 [1.00, 1.00] | 4.333E-02 | 1.00 [1.00, 1.00] | 2.473E-04 | Blood and circulatory | ukb-b-14177 |
| Asthma (childhood onset) | Asthma (childhood onset) | NA | NA | 0.01 [0.01, 0.02] | 4.073E-196 | 3.62 [19.24, 29.0( | 1.246E-200 | Respiratory | ebi-a-GCST007800 |
| Systemic lupus erythematosus | SLE_S2 | 0.76 [0.65, 0.89] | 9.211E-04 | 1.08 [0.92, 1.27] | 3.246E-01 | 0.98 [0.87, 1.10] | 6.950E-01 | Musculoskeletal | ieu-a-815 |
| Rheumatoid Arthritis | RA_S6 | 1.03 [1.00, 1.06] | 6.655E-02 | 1.03 [1.00, 1.06] | 4.570E-02 | 0.97 [0.95, 1.00] | 4.456E-02 | Musculoskeletal | bbj-a-73 |
| Blood clot DVT bronchitis emphysema asthma rhinitis eczema allergy diagnosed by doctor: Hayfever allergic rhinitis or eczema | Diagnosis_2 | 1.01 [1.00, 1.01] | 5.119E-08 | 1.00 [1.00, 1.01] | 9.143E-04 | 1.01 [1.01, 1.01] | 3.644E-15 | Skin | ukb-a-447 |
| Disorders of the thyroid gland | Disorders_2 | 1.01 [0.99, 1.03] | 2.590E-01 | 1.02 [1.00, 1.04] | 8.075E-02 | 0.99 [0.98, 1.01] | 5.084E-01 | Endocrine, nutritional and metabolic | finn-b-E4_THYROID |
| Hypothyroidism (congenital or acquired) | HYPOTHY | 1.04 [1.02, 1.07] | 1.182E-03 | 1.04 [1.02, 1.07] | 8.470E-04 | 1.02 [0.99, 1.04] | 1.439E-01 | Endocrine, nutritional and metabolic | finn-b-HYPOTHYROIDISM |
| Diagnoses - main ICD10: D22 Melanocytic naevi | Melanocytic naevi | 1.00 [1.00, 1.00] | 5.060E-01 | 1.00 [1.00, 1.00] | 5.752E-01 | 1.00 [1.00, 1.00] | 9.634E-01 | Neoplasms | ukb-d-D22 |
| Celiac disease | CeD_S1 | 1.27 [1.15, 1.39] | 7.474E-07 | 1.17 [1.07, 1.28] | 3.281E-04 | 1.02 [0.96, 1.09] | 5.249E-01 | Digestive | ebi-a-GCST000612 |

| | | | | | | | | | | |
|---|---|---|---|---|---|---|---|---|---|---|
| Celiac disease | CeD_S6 | 1.27 [1.16, 1.40] | 5.439E-07 | 1.17 [1.07, 1.28] | 3.354E-04 | 1.02 [0.96, 1.09] | 5.305E-01 | Digestive | ieu-a-276 |
| Thyrotoxicosis with diffuse goitre | THYTOXGOITDIF | 0.99 [0.94, 1.04] | 6.019E-01 | 0.99 [0.94, 1.05] | 7.511E-01 | 1.00 [0.95, 1.04] | 8.944E-01 | Endocrine, nutritional and metabolic | finn-b-E4_THYTOXGOITDIF |
| Hypothyroidism, levothyroxin purchases | HYPOTHY_LEVOTHY | 1.04 [1.01, 1.07] | 1.689E-03 | 1.04 [1.02, 1.07] | 1.067E-03 | 1.01 [0.99, 1.04] | 1.885E-01 | Endocrine, nutritional and metabolic | finn-b-HYPOTHY_LEVOTHY |
| Asthma | Asthma_meta | 0.98 [0.95, 1.00] | 1.050E-01 | 0.98 [0.95, 1.01] | 1.050E-01 | 1.02 [1.00, 1.05] | 5.833E-02 | Respiratory | ebi-a-GCST006862 |
| Myocardial infarction | Asthma_unspecified | 1.02 [1.01, 1.03] | 1.185E-03 | 1.01 [0.99, 1.02] | 4.281E-01 | 1.01 [1.00, 1.02] | 1.128E-01 | Blood and circulatory | ebi-a-GCST011365 |
| Gastroesophageal reflux disease | GERD | 1.01 [1.00, 1.02] | 7.164E-02 | 1.01 [1.00, 1.02] | 7.097E-02 | 1.01 [1.00, 1.02] | 1.600E-01 | Digestive | ebi-a-GCST90000514 |
| Coronary atherosclerosis | CORATHER | 1.00 [0.98, 1.03] | 6.692E-01 | 1.01 [0.98, 1.03] | 5.655E-01 | 1.01 [0.99, 1.03] | 3.687E-01 | Blood and circulatory | finn-b-I9_CORATHER |
| Diabetes, varying definitions | Diabetes_FG | 0.99 [0.98, 1.01] | 3.978E-01 | 0.98 [0.97, 1.00] | 8.616E-02 | 1.01 [1.00, 1.03] | 1.085E-01 | Endocrine, nutritional and metabolic | finn-b-DIABETES_FG |
| Autoimmune diseases | Autoimmune diseases | 1.03 [1.01, 1.05] | 2.601E-04 | 1.02 [1.01, 1.04] | 4.889E-03 | 1.00 [0.99, 1.02] | 5.875E-01 | Blood and circulatory | finn-b-AUTOIMMUNE |
| Coronary atherosclerosis (no controls excluded) | CORATHER_EXNONE | 1.00 [0.98, 1.02] | 8.322E-01 | 1.00 [0.98, 1.02] | 9.984E-01 | 1.01 [0.99, 1.03] | 2.738E-01 | Blood and circulatory | finn-b-I9_CORATHER_EXNONE |
| Type 2 diabetes, definitions combined, including | T2D_INCLAVO | 0.98 [0.97, 1.00] | 8.151E-02 | 0.98 [0.96, 1.00] | 2.835E-02 | 1.00 [0.99, 1.02] | 7.614E-01 | Endocrine, nutritional and metabolic | finn-b-T2D_INCLAVO |
| Disorders of gallbladder, biliary tract and pancreas | Disorders_3 | 1.00 [1.00, 1.00] | 3.615E-01 | 1.00 [1.00, 1.00] | 8.600E-01 | 1.00 [1.00, 1.00] | 6.179E-01 | Digestive | ukb-d-K11_GALLBILPANC |
| Vascular/heart problems diagnosed by doctor: None of the above | Diagnosis_1 | 1.00 [0.99, 1.00] | 6.445E-07 | 1.00 [1.00, 1.00] | 1.607E-02 | 1.00 [1.00, 1.00] | 9.607E-02 | Blood and circulatory | ukb-b-13352 |
| Diabetes, insuline treatment (Kela reimbursement) | KELA_DIAB_INSUL | 0.99 [0.98, 1.01] | 5.253E-01 | 0.98 [0.96, 1.00] | 8.440E-02 | 1.01 [0.99, 1.02] | 4.752E-01 | Endocrine, nutritional and metabolic | finn-b-KELA_DIAB_INSUL |
| Type 2 diabetes | T2D_S3 | 1.01 [0.98, 1.04] | 5.922E-01 | 1.00 [0.97, 1.03] | 8.522E-01 | 0.99 [0.96, 1.01] | 2.630E-01 | Endocrine, nutritional and metabolic | ebi-a-GCST007517 |
| Ischaemic heart disease, wide definition | IHD | 0.99 [0.98, 1.01] | 5.752E-01 | 1.00 [0.98, 1.02] | 6.626E-01 | 1.00 [0.98, 1.02] | 8.705E-01 | Blood and circulatory | finn-b-I9_IHD |
| Major depression | Major depression | 1.00 [0.99, 1.01] | 8.052E-01 | 1.00 [1.00, 1.01] | 2.690E-01 | 1.00 [1.00, 1.01] | 3.649E-01 | Mental and Nervous | ieu-b-102 |
| Nasal polyp | Nasal polyp | 1.04 [0.99, 1.09] | 9.799E-02 | 0.99 [0.94, 1.03] | 5.668E-01 | 1.03 [0.98, 1.07] | 2.348E-01 | Respiratory | finn-b-J10_NASALPOLYP |
| Rheumatoid arthritis | RA_S2 | 1.01 [0.99, 1.04] | 3.607E-01 | 1.02 [0.99, 1.04] | 2.667E-01 | 0.99 [0.96, 1.01] | 1.686E-01 | Musculoskeletal | ebi-a-GCST002318 |
| Other diabetes, wide definition | Diabetes_wide | 0.99 [0.98, 1.01] | 5.236E-01 | 0.98 [0.96, 1.00] | 2.734E-02 | 1.01 [0.99, 1.02] | 3.752E-01 | Endocrine, nutritional and metabolic | finn-b-DM_OTHER_WIDE |
| Mouth/teeth dental problems: Mouth ulcers | Asthma_diagnosed | 1.00 [1.00, 1.00] | 6.367E-02 | 1.00 [1.00, 1.00] | 1.467E-01 | 1.00 [1.00, 1.00] | 1.286E-02 | Digestive | ukb-a-427 |
| Type 2 diabetes, strict (exclude DM1) | T2D_STRICT | 0.98 [0.96, 1.00] | 1.794E-02 | 0.98 [0.96, 1.00] | 2.902E-02 | 1.00 [0.99, 1.02] | 6.918E-01 | Endocrine, nutritional and metabolic | finn-b-E4_DM2_STRICT |
| Type 2 diabetes, definitions combined | T2D_S5 | 0.98 [0.97, 1.00] | 8.814E-02 | 0.98 [0.96, 1.00] | 3.339E-02 | 1.00 [0.99, 1.02] | 6.651E-01 | Endocrine, nutritional and metabolic | finn-b-T2D |
| Pure hypercholesterolaemia | FH_Finn | 1.01 [0.98, 1.04] | 3.819E-01 | 1.00 [0.97, 1.03] | 9.422E-01 | 0.99 [0.97, 1.01] | 4.424E-01 | Endocrine, nutritional and metabolic | finn-b-E4_HYPERCHOL |
| Celiac disease | CeD_S4 | 1.12 [1.01, 1.25] | 3.463E-02 | 1.10 [1.00, 1.22] | 5.197E-02 | 1.00 [0.93, 1.07] | 9.247E-01 | Digestive | ieu-a-1059 |
| Basal cell carcinoma | BCC | 1.02 [1.00, 1.04] | 7.276E-02 | 1.03 [1.01, 1.05] | 6.061E-03 | 1.02 [1.00, 1.04] | 1.696E-02 | Neoplasms | ebi-a-GCST90013410 |
| Diagnoses - main ICD10: D12 Benign neoplasm of colon, rectum, anus and anal canal | Digestive Neoplasms | 1.00 [1.00, 1.00] | 9.260E-02 | 1.00 [1.00, 1.00] | 3.828E-01 | 1.00 [1.00, 1.00] | 1.883E-01 | Neoplasms | ukb-d-D12 |
| Colorectal cancer | Colorectal cancer | 1.00 [1.00, 1.00] | 2.475E-01 | 1.00 [1.00, 1.00] | 4.442E-01 | 1.00 [1.00, 1.00] | 1.547E-01 | Digestive | ieu-b-4965 |
| Diagnoses - secondary ICD10: E78.0 Pure hypercholesterolaemia | FH_UKB | 1.00 [1.00, 1.00] | 6.387E-01 | 1.00 [1.00, 1.00] | 5.951E-01 | 1.00 [1.00, 1.00] | 1.400E-01 | Endocrine, nutritional and metabolic | ukb-b-12651 |
| Diagnoses - secondary ICD10: E11.9 Without | T2D_nocom | 1.00 [1.00, 1.00] | 4.996E-02 | 1.00 [1.00, 1.00] | 6.285E-02 | 1.00 [1.00, 1.00] | 4.938E-01 | Endocrine, nutritional and metabolic | ukb-b-970 |
| Hypothyroidism, strict autoimmune, 3 medication purchases required | HYTHY_AI_STRICT_PURCH | 1.01 [0.99, 1.03] | 3.070E-01 | 1.01 [0.99, 1.03] | 3.330E-01 | 0.99 [0.97, 1.01] | 2.715E-01 | Endocrine, nutritional and metabolic | finn-b-E4_HYTHY_AI_STRICT_PURCH |
| Disorders of lipoprotein metabolism and other | Disorders_1 | 1.00 [0.97, 1.02] | 7.180E-01 | 1.00 [0.98, 1.02] | 9.763E-01 | 0.99 [0.97, 1.01] | 2.906E-01 | Endocrine, nutritional and metabolic | finn-b-E4_LIPOPROT |
| Haemorrhoidal disease | HD | 1.02 [1.02, 1.03] | 5.069E-11 | 1.01 [1.01, 1.02] | 5.086E-05 | 1.02 [1.02, 1.03] | 1.544E-13 | Digestive | ebi-a-GCST90014033 |
| Myocardial infarction | Allergic disease | 1.01 [0.99, 1.03] | 2.645E-01 | 1.00 [0.98, 1.02] | 9.489E-01 | 1.01 [1.00, 1.03] | 1.447E-01 | Blood and circulatory | ebi-a-GCST011364 |
| Parkinson's disease | PD | NA | NA | NA | NA | 0.99 [0.96, 1.02] | 5.377E-01 | Mental and Nervous | ieu-b-7 |
| Coronary heart disease | CHD | 1.02 [0.99, 1.05] | 1.370E-01 | 1.02 [0.98, 1.05] | 3.719E-01 | 1.02 [0.99, 1.04] | 1.428E-01 | Blood and circulatory | ieu-a-8 |
| Prostate cancer | Prostate cancer | 0.98 [0.97, 1.00] | 1.621E-02 | 1.00 [0.98, 1.01] | 7.249E-01 | 0.99 [0.98, 1.01] | 3.879E-01 | Neoplasms | ebi-a-GCST006085 |
| Rheumatoid arthritis | RA_S1 | 0.95 [0.90, 1.00] | 6.539E-02 | 1.01 [0.95, 1.07] | 7.874E-01 | 0.95 [0.90, 0.99] | 1.684E-02 | Musculoskeletal | ebi-a-GCST000679 |
| Asthma (adult onset) | Asthma (adult onset) | NA | NA | 0.01 [0.01, 0.02] | 1.144E-179 | 2.85 [18.73, 27.8 | 1.320E-208 | Respiratory | ebi-a-GCST007799 |
| Cancer of skin | Cancer of skin | 1.00 [1.00, 1.00] | 1.408E-01 | 1.00 [1.00, 1.00] | 7.436E-03 | 1.00 [1.00, 1.00] | 1.619E-01 | Neoplasms | ukb-d-C_SKIN |
| Type 2 diabetes | T2D_S4 | 0.98 [0.96, 1.00] | 5.218E-02 | 0.98 [0.97, 1.00] | 1.116E-01 | 1.01 [1.00, 1.03] | 1.535E-01 | Endocrine, nutritional and metabolic | finn-b-E4_DM2 |
| Thyroid cancer | Thyroid cancer | 1.17 [0.90, 1.51] | 2.335E-01 | 1.09 [0.85, 1.39] | 4.943E-01 | 1.11 [0.92, 1.33] | 2.964E-01 | Endocrine, nutritional and metabolic | ieu-a-1082 |
| Type 2 diabetes with other specified/multiple/unspecified complications | T2D_NASCOMP | 0.98 [0.96, 1.00] | 4.448E-02 | 0.98 [0.96, 1.00] | 3.733E-02 | 1.00 [0.98, 1.01] | 6.333E-01 | Endocrine, nutritional and metabolic | finn-b-E4_DM2NASCOMP |
| Hypothyroidism, strict autoimmune | HYTHY_AI_STRICT | 1.01 [0.99, 1.03] | 2.765E-01 | 1.02 [1.00, 1.04] | 5.048E-02 | 0.99 [0.97, 1.01] | 1.897E-01 | Endocrine, nutritional and metabolic | finn-b-E4_HYTHY_AI_STRICT |
| Type 2 diabetes | T2D_S1 | 1.00 [0.97, 1.02] | 7.573E-01 | 1.00 [0.97, 1.02] | 8.856E-01 | 1.01 [0.99, 1.03] | 5.809E-01 | Endocrine, nutritional and metabolic | ebi-a-GCST005047 |
| Psoriasis | Psoriasis | NA | NA | NA | NA | NA | NA | Skin | ebi-a-GCST005527 |
| Breast cancer | Breast cancer_S1 | 1.02 [1.01, 1.03] | 3.374E-05 | 1.02 [1.01, 1.03] | 2.792E-03 | 1.01 [1.00, 1.02] | 6.935E-02 | Neoplasms | ebi-a-GCST004988 |

| | | | | | | | | | |
|---|---|---|---|---|---|---|---|---|---|
| multiple sclerosis | MS_S4 | 1.01 [0.98, 1.05] | 4.121E-01 | 1.00 [0.96, 1.03] | 8.784E-01 | 1.01 [0.98, 1.04] | 4.399E-01 | Mental and Nervous | ieu-b-18 |
| Bipolar disorder | Bipolar disorder | 1.23 [0.98, 1.56] | 7.773E-02 | 1.08 [0.81, 1.42] | 6.043E-01 | 0.97 [0.77, 1.21] | 7.775E-01 | Mental and Nervous | ieu-a-800 |
| Narcolepsy | Narcolepsy | NA | NA | NA | NA | NA | NA | Mental and Nervous | ebi-a-GCST005522 |
| schizophrenia | Schizophrenia | 1.00 [0.99, 1.02] | 6.394E-01 | 1.01 [0.98, 1.03] | 6.179E-01 | 1.01 [0.99, 1.03] | 3.802E-01 | Mental and Nervous | ieu-b-42 |
| Hypothyroidism, other/unspecified | HYTHYNAS | 1.01 [0.99, 1.03] | 3.545E-01 | 1.02 [1.00, 1.04] | 2.962E-02 | 0.99 [0.98, 1.01] | 3.681E-01 | Endocrine, nutritional and metabolic | finn-b-E4_HYTHYNAS |
| Rheumatoid arthritis | RA_S3 | 1.03 [1.00, 1.07] | 7.811E-02 | 1.05 [1.01, 1.09] | 9.466E-03 | 1.00 [0.97, 1.03] | 7.694E-01 | Musculoskeletal | ebi-a-GCST005569 |
| Rheumatoid arthritis | RA_S5 | 0.96 [0.91, 1.02] | 1.568E-01 | 1.01 [0.95, 1.07] | 8.607E-01 | 0.95 [0.91, 0.99] | 2.328E-02 | Musculoskeletal | ieu-a-834 |
| Type 2 diabetes | T2D_S2 | 1.02 [1.00, 1.03] | 1.755E-02 | 1.02 [1.00, 1.03] | 5.685E-02 | 1.01 [1.00, 1.02] | 1.906E-01 | Endocrine, nutritional and metabolic | ebi-a-GCST006867 |
| Type 2 diabetes (adjusted for BMI) | T2D_adjusted | 1.00 [0.97, 1.03] | 9.112E-01 | 1.00 [0.97, 1.02] | 7.456E-01 | 0.98 [0.95, 1.00] | 2.735E-02 | Endocrine, nutritional and metabolic | ebi-a-GCST007518 |
| Type 2 diabetes | T2D_S6 | 0.99 [0.97, 1.02] | 5.930E-01 | 1.00 [0.97, 1.02] | 8.315E-01 | 0.99 [0.97, 1.01] | 2.277E-01 | Endocrine, nutritional and metabolic | ebi-a-GCST007515 |
| Blood clot DVT bronchitis emphysema asthma rhinitis eczema allergy diagnosed by doctor: Asthma | Diagnosis_1 | 1.00 [1.00, 1.00] | 4.533E-01 | 1.00 [1.00, 1.00] | 8.392E-01 | 1.00 [1.00, 1.00] | 1.471E-05 | Respiratory | ukb-a-446 |
| Breast cancer | Breast cancer_S2 | NA | NA | 51.97 [41.98, 64.34] | 4.656E-288 | 0.05 [0.04, 0.06] | 1.544E-286 | Neoplasms | ebi-a-GCST007236 |
| Doctor diagnosed asthma | Asthma_diagnosed | 1.00 [1.00, 1.01] | 7.044E-02 | 1.00 [1.00, 1.00] | 2.855E-01 | 1.01 [1.00, 1.01] | 5.934E-05 | Respiratory | ukb-a-255 |
| Osteoarthritis of the hip or knee | OA of hip/knee | 0.99 [0.98, 1.00] | 5.200E-02 | 0.99 [0.98, 1.01] | 3.665E-01 | 0.98 [0.97, 0.99] | 2.760E-04 | Musculoskeletal | ebi-a-GCST007092 |

**Supplementary Table 4. SMR analysis in lung tissue**

| probeID | ProbeChr | Gene | Probe_bp | topSNP | topSNP_chr | topSNP_bp | b_SMR | se_SMR | p_SMR | p_HEIDI | nsnp_HEIDI |
|---|---|---|---|---|---|---|---|---|---|---|---|
| ENSG00000138031.10 | 2 | ADCY3 | 25092373 | rs13407913 | 2 | 25097644 | -0.35 | 0.07 | 1.43E-06 | NA | NA |
| ENSG00000169220.13 | 5 | RGS14 | 176792220 | rs35716097 | 5 | 176806636 | 0.30 | 0.06 | 3.07E-06 | 3.82E-01 | 5 |
| ENSG00000122873.7 | 10 | CISD1 | 60039082 | rs2440858 | 10 | 59965710 | -0.27 | 0.05 | 6.30E-07 | 1.10E-02 | 13 |
| ENSG00000165688.7 | 9 | PMPCA | 139311661 | rs35873563 | 9 | 139324029 | -0.64 | 0.13 | 1.58E-06 | 9.31E-05 | 20 |
| ENSG00000161180.6 | 22 | CCDC116 | 21989310 | rs2266963 | 22 | 21947467 | 0.16 | 0.03 | 3.51E-08 | 7.05E-05 | 5 |
| ENSG00000182179.6 | 3 | UBA7 | 49847009 | rs2247036 | 3 | 49882349 | -0.72 | 0.15 | 1.17E-06 | 4.11E-05 | 10 |
| ENSG00000187796.9 | 9 | CARD9 | 139263270 | rs10781499 | 9 | 139266405 | -0.99 | 0.18 | 6.07E-08 | 3.92E-05 | 20 |
| ENSG00000161395.8 | 17 | PGAP3 | 37840212 | rs2934956 | 17 | 37830447 | -0.37 | 0.07 | 6.81E-08 | 2.10E-05 | 20 |
| ENSG00000026297.11 | 6 | RNASET2 | 167356832 | rs2149093 | 6 | 167373045 | 0.20 | 0.03 | 4.85E-10 | 3.23E-06 | 20 |
| ENSG00000138092.6 | 2 | CENPO | 25030748 | rs13407913 | 2 | 25097644 | -0.30 | 0.05 | 3.49E-08 | 3.22E-06 | 3 |
| ENSG00000068745.10 | 3 | IP6K2 | 48751611 | rs17595772 | 3 | 49409704 | 0.46 | 0.09 | 9.04E-07 | 2.43E-06 | 7 |
| ENSG00000113522.9 | 5 | RAD50 | 131936012 | rs2070722 | 5 | 131824486 | 0.38 | 0.07 | 6.19E-08 | 1.66E-06 | 9 |
| ENSG00000251417.1 | 16 | RP11-1348G14.4 | 28821623 | rs62036622 | 16 | 28837203 | 0.20 | 0.04 | 2.44E-08 | 9.55E-07 | 20 |
| ENSG00000169682.13 | 16 | SPNS1 | 28990705 | rs62036622 | 16 | 28837203 | -0.42 | 0.08 | 7.07E-07 | 8.34E-07 | 18 |
| ENSG00000135926.8 | 2 | TMBIM1 | 219148112 | rs736731 | 2 | 219120588 | -0.44 | 0.09 | 8.62E-07 | 6.38E-08 | 11 |
| ENSG00000244731.3 | 6 | C4A | 31960129 | rs2105902 | 6 | 32395698 | -0.13 | 0.02 | 2.64E-08 | 1.96E-08 | 20 |
| ENSG00000233006.2 | 5 | AC034220.3 | 131676159 | rs272894 | 5 | 131662460 | 0.17 | 0.03 | 3.21E-07 | 1.45E-08 | 17 |
| ENSG00000160766.10 | 1 | GBAP1 | 155190415 | rs2990245 | 1 | 155197462 | 0.16 | 0.03 | 2.41E-08 | 1.22E-08 | 20 |
| ENSG00000204351.7 | 6 | SKIV2L | 31932210 | rs389883 | 6 | 31947460 | -0.30 | 0.06 | 4.23E-06 | 8.83E-09 | 20 |
| ENSG00000008838.13 | 17 | MED24 | 38196409 | rs3935280 | 17 | 38189055 | 0.24 | 0.05 | 6.10E-06 | 2.98E-09 | 9 |
| ENSG00000101216.6 | 20 | GMEB2 | 62238674 | rs13043255 | 20 | 62218916 | 0.33 | 0.07 | 3.37E-06 | 1.99E-09 | 12 |
| ENSG00000196502.7 | 16 | SULT1A1 | 28625924 | rs3020803 | 16 | 28618914 | 0.21 | 0.04 | 4.03E-09 | 7.76E-10 | 18 |
| ENSG00000115604.6 | 2 | IL18R1 | 102993803 | rs7568913 | 2 | 102920037 | 0.30 | 0.05 | 5.88E-09 | 2.96E-10 | 20 |
| ENSG00000203963.7 | 1 | C1orf141 | 67627692 | rs12060309 | 1 | 67604219 | -0.47 | 0.06 | 6.22E-15 | 1.05E-10 | 12 |
| ENSG00000164068.11 | 3 | RNF123 | 49740422 | rs9855505 | 3 | 49792158 | -0.38 | 0.06 | 1.39E-09 | 3.82E-11 | 20 |
| ENSG00000145020.10 | 3 | AMT | 49457198 | rs6446275 | 3 | 49466871 | -0.13 | 0.03 | 7.08E-07 | 3.00E-11 | 20 |
| ENSG00000073605.14 | 17 | GSDMB | 38067875 | rs1008723 | 17 | 38066267 | -0.47 | 0.07 | 1.88E-10 | 1.92E-11 | 20 |
| ENSG00000101017.9 | 20 | CD40 | 44752706 | rs4239702 | 20 | 44749251 | -0.22 | 0.05 | 2.75E-06 | 6.33E-12 | 20 |
| ENSG00000176998.3 | 6 | HCG4 | 29759514 | rs1632995 | 6 | 29769621 | -0.07 | 0.01 | 6.32E-08 | 3.98E-12 | 20 |
| ENSG00000198502.5 | 6 | HLA-DRB5 | 32491592 | rs9271255 | 6 | 32580357 | 0.14 | 0.02 | 6.31E-18 | 8.70E-13 | 20 |
| ENSG00000169231.9 | 1 | THBS3 | 155171543 | rs914615 | 1 | 155175892 | 0.25 | 0.05 | 7.46E-07 | 4.21E-13 | 20 |
| ENSG00000260302.1 | 18 | RP11-973H7.1 | 12775286 | rs2542148 | 18 | 12777573 | 0.23 | 0.04 | 2.31E-09 | 2.76E-13 | 11 |
| ENSG00000054219.9 | 2 | LY75 | 160710563 | rs4664304 | 2 | 160794008 | -0.09 | 0.02 | 1.49E-07 | 1.82E-13 | 7 |
| ENSG00000172057.5 | 17 | ORMDL3 | 38080574 | rs7224129 | 17 | 38075426 | 0.43 | 0.07 | 3.75E-10 | 1.31E-13 | 20 |
| ENSG00000138111.10 | 10 | TMEM180 | 104228975 | rs2274351 | 10 | 104264107 | -0.22 | 0.04 | 8.16E-07 | 3.34E-14 | 7 |
| ENSG00000197146.2 | 6 | AL133458.1 | 167359002 | rs434093 | 6 | 167382449 | -0.25 | 0.04 | 8.16E-09 | 3.14E-14 | 20 |
| ENSG00000163466.11 | 2 | ARPC2 | 219100448 | rs12694432 | 2 | 219082330 | -0.41 | 0.07 | 3.96E-08 | 1.99E-14 | 17 |
| ENSG00000197083.7 | 5 | ZNF300P1 | 150318029 | rs59980321 | 5 | 150241813 | -0.13 | 0.02 | 7.98E-19 | 3.69E-15 | 4 |
| ENSG00000178952.4 | 16 | TUFM | 28855730 | rs7187776 | 16 | 28857645 | -0.38 | 0.06 | 4.25E-12 | 3.59E-15 | 20 |
| ENSG00000197375.8 | 5 | SLC22A5 | 131718375 | rs2631360 | 5 | 131707429 | -0.25 | 0.04 | 3.11E-12 | 7.70E-16 | 20 |
| ENSG00000196301.3 | 6 | HLA-DRB9 | 32434437 | rs9271255 | 6 | 32580357 | -0.17 | 0.02 | 5.16E-16 | 3.78E-19 | 20 |
| ENSG00000241106.2 | 6 | HLA-DOB | 32782682 | rs2621326 | 6 | 32783896 | 0.09 | 0.02 | 2.15E-08 | 4.68E-20 | 20 |
| ENSG00000004534.10 | 3 | RBM6 | 50046061 | rs9866695 | 3 | 50069452 | -0.17 | 0.03 | 1.35E-11 | 4.47E-20 | 20 |
| ENSG00000259982.1 | 16 | CDC37P1 | 28712238 | rs62036620 | 16 | 28833097 | -0.12 | 0.02 | 1.10E-13 | 1.36E-20 | 20 |
| ENSG00000237541.3 | 6 | HLA-DQA2 | 32712055 | rs9268835 | 6 | 32428115 | 0.12 | 0.01 | 2.48E-19 | 1.51E-23 | 20 |
| ENSG00000261338.1 | 2 | RP11-378A13.1 | 219121064 | rs736730 | 2 | 219120255 | -0.12 | 0.02 | 1.28E-11 | 4.71E-31 | 20 |
| ENSG00000173209.18 | 2 | AHSA2 | 61411445 | rs2167565 | 2 | 61476458 | -0.16 | 0.03 | 2.47E-08 | 3.91E-31 | 20 |
| ENSG00000167914.6 | 17 | GSDMA | 38126622 | rs3859191 | 17 | 38128714 | -0.10 | 0.01 | 1.57E-12 | 2.50E-32 | 20 |
| ENSG00000115602.12 | 2 | IL1RL1 | 102948229 | rs1420102 | 2 | 102948819 | 0.17 | 0.02 | 1.35E-13 | 7.78E-33 | 20 |
| ENSG00000229391.3 | 6 | HLA-DRB6 | 32524144 | rs9268838 | 6 | 32428715 | 0.14 | 0.02 | 2.20E-18 | 1.93E-34 | 20 |
| ENSG00000164308.12 | 5 | ERAP2 | 96233531 | rs2910686 | 5 | 96252589 | -0.06 | 0.01 | 5.33E-12 | 8.57E-60 | 20 |

**Supplementary Table 5. SMR analysis in whole blood**

| probeID | ProbeChr | Gene | Probe_bp | topSNP | topSNP_chr | topSNP_bp | b_SMR | se_SMR | p_SMR | p_HEIDI | nsnp_HEIDI |
|---|---|---|---|---|---|---|---|---|---|---|---|
| ENSG00000187796.9 | 9 | CARD9 | 139263270 | rs4078099 | 9 | 139267533 | 0.51 | 0.06 | 6.51E-19 | 1.61E-01 | 20 |
| ENSG00000026297.11 | 6 | RNASET2 | 167356832 | rs434093 | 6 | 167382449 | -0.17 | 0.02 | 9.26E-13 | 6.90E-02 | 20 |
| ENSG00000197146.2 | 6 | AL133458.1 | 167359002 | rs444210 | 6 | 167390242 | -0.18 | 0.03 | 3.34E-12 | 1.50E-01 | 20 |
| ENSG00000073605.14 | 17 | GSDMB | 38067875 | rs8067378 | 17 | 38051348 | -0.34 | 0.04 | 6.39E-18 | 5.20E-04 | 20 |
| ENSG00000229391.3 | 6 | HLA-DRB6 | 32524144 | rs9268838 | 6 | 32428715 | -0.17 | 0.02 | 9.18E-18 | 9.67E-04 | 20 |
| ENSG00000148396.14 | 9 | SEC16A | 139353345 | rs11145760 | 9 | 139382594 | -0.36 | 0.04 | 3.71E-17 | 2.74E-04 | 20 |
| ENSG00000197165.6 | 16 | SULT1A2 | 28605847 | rs180744 | 16 | 28508048 | 0.10 | 0.01 | 1.58E-16 | 9.35E-03 | 20 |
| ENSG00000178952.4 | 16 | TUFM | 28855730 | rs7187776 | 16 | 28857645 | 0.17 | 0.02 | 3.91E-16 | 9.78E-04 | 20 |
| ENSG00000164308.12 | 5 | ERAP2 | 96233531 | rs2910686 | 5 | 96252589 | 0.07 | 0.01 | 6.96E-12 | 1.12E-01 | 20 |
| ENSG00000261338.1 | 2 | RP11-378A13.1 | 219121064 | rs11677953 | 2 | 219121663 | -0.22 | 0.03 | 1.14E-10 | 2.51E-01 | 20 |
| ENSG00000177628.11 | 1 | GBA | 155209366 | rs1045253 | 1 | 155201235 | 0.22 | 0.05 | 1.61E-06 | 2.03E-02 | 20 |
| ENSG00000172057.5 | 17 | ORMDL3 | 38080574 | rs7224129 | 17 | 38075426 | -0.26 | 0.03 | 1.23E-14 | 5.05E-05 | 20 |
| ENSG00000197375.8 | 5 | SLC22A5 | 131718375 | rs2631360 | 5 | 131707429 | -0.22 | 0.03 | 7.14E-14 | 4.94E-04 | 20 |
| ENSG00000182179.6 | 3 | UBA7 | 49847009 | rs7372725 | 3 | 49790544 | 0.32 | 0.04 | 2.45E-13 | 5.06E-09 | 20 |
| ENSG00000198502.5 | 6 | HLA-DRB5 | 32491592 | rs9270986 | 6 | 32574060 | 0.12 | 0.02 | 2.54E-12 | 1.30E-06 | 20 |
| ENSG00000225851.1 | 6 | HLA-S | 31349958 | rs9264942 | 6 | 31274380 | 0.12 | 0.02 | 4.39E-12 | 5.94E-10 | 20 |
| ENSG00000138092.6 | 2 | CENPO | 25030748 | rs13407913 | 2 | 25097644 | 0.15 | 0.02 | 5.15E-13 | 4.22E-02 | 4 |
| ENSG00000138031.10 | 2 | ADCY3 | 25092373 | rs13407913 | 2 | 25097644 | 0.11 | 0.01 | 6.84E-16 | 1.17E-02 | 5 |
| ENSG00000115137.7 | 2 | DNAJC27 | 25180734 | rs13407913 | 2 | 25097644 | 0.23 | 0.04 | 1.93E-10 | 2.86E-02 | 4 |
| ENSG00000235888.1 | 21 | AF064858.8 | 40369356 | rs9977672 | 21 | 40463283 | 0.64 | 0.10 | 5.04E-10 | 7.30E-02 | 7 |
| ENSG00000196301.3 | 6 | HLA-DRB9 | 32434437 | rs9271255 | 6 | 32580357 | -0.24 | 0.03 | 7.99E-12 | 4.15E-03 | 20 |
| ENSG00000004534.10 | 3 | RBM6 | 50046061 | rs7621352 | 3 | 50070763 | 0.13 | 0.02 | 1.82E-11 | 2.64E-08 | 20 |
| ENSG00000100321.10 | 22 | SYNGR1 | 39763761 | rs61616683 | 22 | 39755773 | 0.20 | 0.03 | 1.93E-09 | 1.56E-07 | 20 |
| ENSG00000165689.12 | 9 | SDCCAG3 | 139300719 | rs10747043 | 9 | 139318310 | -0.33 | 0.06 | 3.88E-09 | 7.56E-06 | 20 |
| ENSG00000237721.1 | 21 | AF064858.11 | 40380914 | rs9977672 | 21 | 40463283 | 0.62 | 0.10 | 7.41E-10 | 6.80E-02 | 6 |
| ENSG00000115607.5 | 2 | IL18RAP | 103052087 | rs2272128 | 2 | 103039929 | -0.25 | 0.04 | 5.53E-09 | 7.62E-12 | 20 |
| ENSG00000122873.7 | 10 | CISD1 | 60039082 | rs2790167 | 10 | 60024470 | 0.13 | 0.02 | 3.76E-09 | 5.01E-01 | 20 |
| ENSG00000169231.9 | 1 | THBS3 | 155171543 | rs4072037 | 1 | 155162067 | 0.19 | 0.03 | 1.72E-08 | 6.82E-01 | 20 |
| ENSG00000173209.18 | 2 | AHSA2 | 61411445 | rs2167565 | 2 | 61476458 | -0.14 | 0.03 | 3.80E-08 | 5.81E-01 | 20 |
| ENSG00000233232.2 | 16 | NPIPB7 | 28474780 | rs240702 | 16 | 28531353 | -0.22 | 0.04 | 6.78E-09 | 9.87E-03 | 20 |

| | | | | | | | | | | | | |
|---|---|---|---|---|---|---|---|---|---|---|---|---|
| ENSG00000169682.13 | 16 | SPNS1 | 28990705 | rs7140 | 16 | 28995757 | 0.23 | 0.04 | 8.51E-09 | 2.45E-05 | 20 |
| ENSG00000176476.4 | 16 | CCDC101 | 28584179 | rs480400 | 16 | 28574615 | -0.37 | 0.06 | 8.66E-09 | 1.20E-03 | 20 |
| ENSG00000196126.6 | 6 | HLA-DRB1 | 32552085 | rs9268614 | 6 | 32402778 | 0.42 | 0.07 | 1.57E-08 | 1.49E-07 | 20 |
| ENSG00000255389.1 | 6 | C6orf3 | 111922288 | rs33980500 | 6 | 111913262 | -0.19 | 0.03 | 7.08E-08 | 6.45E-03 | 20 |
| ENSG00000204528.3 | 6 | PSORS1C3 | 31143594 | rs1052989 | 6 | 31146403 | -0.12 | 0.02 | 8.53E-08 | 1.44E-04 | 20 |
| ENSG00000223534.1 | 6 | HLA-DQB1-AS1 | 32628319 | rs9270986 | 6 | 32574060 | 0.27 | 0.05 | 1.68E-07 | 7.37E-05 | 20 |
| ENSG00000196502.7 | 16 | SULT1A1 | 28625924 | rs75301646 | 16 | 28617885 | -0.23 | 0.04 | 1.88E-07 | 4.68E-04 | 18 |
| ENSG00000236525.1 | 2 | AC007278.2 | 103051108 | rs1420106 | 2 | 103035044 | -0.33 | 0.06 | 2.63E-07 | 1.96E-06 | 20 |
| ENSG00000231852.2 | 6 | CYP21A2 | 32007744 | rs2856674 | 6 | 32659645 | -0.17 | 0.04 | 1.34E-06 | 5.65E-04 | 20 |
| ENSG00000234745.5 | 6 | HLA-B | 31322934 | rs13218306 | 6 | 31240932 | 0.25 | 0.05 | 1.56E-06 | 3.03E-06 | 20 |
| ENSG00000236935.1 | 11 | AP003774.1 | 64094749 | rs694739 | 11 | 64097233 | -0.07 | 0.02 | 2.05E-06 | 7.37E-04 | 5 |
| ENSG00000213066.7 | 6 | FGFR1OP | 167439435 | rs7760495 | 6 | 167506795 | 0.33 | 0.07 | 2.32E-06 | 4.12E-03 | 14 |
| ENSG00000237669.1 | 6 | HCG4P3 | 29974395 | rs2523971 | 6 | 29938258 | -0.11 | 0.02 | 2.70E-06 | 3.78E-08 | 20 |
| ENSG00000081985.6 | 1 | IL12RB2 | 67817815 | rs17129794 | 1 | 67794918 | -0.21 | 0.04 | 2.88E-06 | 5.79E-03 | 20 |
| ENSG00000206341.6 | 6 | HLA-H | 29857251 | rs2523962 | 6 | 29939544 | -0.13 | 0.03 | 5.46E-06 | 6.76E-04 | 20 |
| ENSG00000127838.9 | 2 | PNKD | 219173315 | rs10932766 | 2 | 219122090 | -0.29 | 0.05 | 5.14E-08 | 1.49E-01 | 19 |
| ENSG00000267654.1 | 18 | RP11-973H7.4 | 12744454 | rs2542157 | 18 | 12787247 | 0.10 | 0.02 | 5.65E-06 | 8.73E-12 | 16 |
| ENSG00000234290.2 | 5 | AC116366.6 | 131806658 | rs2070729 | 5 | 131819921 | 0.43 | 0.07 | 7.34E-11 | 1.30E-02 | 20 |
| ENSG00000204531.11 | 6 | POU5F1 | 31140313 | rs1052989 | 6 | 31146403 | -0.22 | 0.05 | 4.53E-06 | 2.43E-02 | 20 |
| ENSG00000204267.9 | 6 | TAP2 | 32798083 | rs241449 | 6 | 32796653 | 0.23 | 0.05 | 4.57E-06 | 3.98E-02 | 20 |
| ENSG00000197457.5 | 20 | STMN3 | 62277920 | rs3787088 | 20 | 62316634 | 0.47 | 0.09 | 7.28E-08 | 1.00E-01 | 20 |
| ENSG00000116288.8 | 1 | PARK7 | 8029958 | rs3766606 | 1 | 8022197 | -0.58 | 0.11 | 1.01E-07 | 1.86E-01 | 19 |
| ENSG00000185651.10 | 22 | UBE2L3 | 21950079 | rs2070512 | 22 | 21949411 | 0.53 | 0.10 | 3.74E-07 | 4.92E-01 | 4 |
| ENSG00000054219.9 | 2 | LY75 | 160710563 | rs4664304 | 2 | 160794008 | -0.13 | 0.02 | 3.85E-07 | 1.22E-01 | 4 |
| ENSG00000132823.6 | 20 | OSER1 | 42832283 | rs2143606 | 20 | 42838550 | -0.24 | 0.05 | 5.76E-07 | 5.27E-01 | 4 |
| ENSG00000121281.8 | 16 | ADCY7 | 50313497 | rs7184802 | 16 | 50355996 | -0.45 | 0.08 | 2.83E-09 | NA | NA |
| ENSG00000166164.11 | 16 | BRD7 | 50375121 | rs7184802 | 16 | 50355996 | -0.16 | 0.02 | 1.36E-12 | NA | NA |
| ENSG00000011485.10 | 19 | PPP5C | 46873244 | rs4802307 | 19 | 46849806 | 0.14 | 0.03 | 2.01E-07 | NA | NA |
| ENSG00000160766.10 | 1 | GBAP1 | 155190415 | rs2075570 | 1 | 155182164 | -0.14 | 0.03 | 8.39E-07 | 2.70E-01 | 20 |
| ENSG00000151151.5 | 10 | IPMK | 59989486 | rs2590322 | 10 | 59999931 | 0.51 | 0.10 | 9.12E-07 | 6.47E-01 | 20 |
| ENSG00000157557.7 | 21 | ETS2 | 40187055 | rs2836882 | 21 | 40466570 | 1.10 | 0.21 | 1.82E-07 | 3.62E-02 | 5 |
| ENSG00000180871.3 | 2 | CXCR2 | 218995994 | rs4674259 | 2 | 218991005 | -0.69 | 0.14 | 2.08E-06 | 8.19E-02 | 8 |
| ENSG00000176022.3 | 1 | B3GALT6 | 1169025 | rs6697886 | 1 | 1173611 | -0.45 | 0.10 | 3.02E-06 | 8.40E-01 | 5 |

| | | | | | | | | | | | |
|---|---|---|---|---|---|---|---|---|---|---|---|
| ENSG00000186891.9 | 1 | TNFRSF18 | 1140479 | rs6697886 | 1 | 1173611 | -0.33 | 0.07 | 3.97E-06 | 3.75E-01 | 4 |
| ENSG00000025708.8 | 22 | TYMP | 50966333 | rs470119 | 22 | 50966914 | -0.22 | 0.05 | 4.32E-06 | 3.56E-01 | 5 |

**Supplementary Table 6. SMR analysis in muscle skeletal tissue**

| probeID | ProbeChr | Gene | Probe_bp | topSNP | topSNP_chr | topSNP_bp | b_SMR | se_SMR | p_SMR | p_HEIDI | nsnp_HEIDI |
|---|---|---|---|---|---|---|---|---|---|---|---|
| ENSG00000197083.7 | 5 | ZNF300P1 | 150318029 | rs12659118 | 5 | 150324622 | 0.12 | 0.01 | 9.43E-24 | 3.67E-05 | 13 |
| ENSG00000237541.3 | 6 | HLA-DQA2 | 32712055 | rs9268835 | 6 | 32428115 | -0.13 | 0.01 | 3.56E-21 | 7.82E-10 | 20 |
| ENSG00000229391.3 | 6 | HLA-DRB6 | 32524144 | rs9268838 | 6 | 32428715 | -0.14 | 0.02 | 2.74E-20 | 2.06E-06 | 20 |
| ENSG00000185614.4 | 3 | FAM212A | 49841575 | rs9836291 | 3 | 49697459 | -0.48 | 0.06 | 3.51E-17 | 1.11E-02 | 20 |
| ENSG00000196126.6 | 6 | HLA-DRB1 | 32552085 | rs9271255 | 6 | 32580357 | 0.29 | 0.04 | 3.67E-15 | 3.48E-03 | 20 |
| ENSG00000197165.6 | 16 | SULT1A2 | 28605847 | rs62034319 | 16 | 28532188 | 0.13 | 0.02 | 2.27E-14 | 4.49E-03 | 20 |
| ENSG00000198502.5 | 6 | HLA-DRB5 | 32491592 | rs9270986 | 6 | 32574060 | 0.08 | 0.01 | 2.73E-13 | 2.84E-07 | 20 |
| ENSG00000260442.1 | 16 | RP11-22P6.3 | 28891073 | rs8062405 | 16 | 28837906 | -0.22 | 0.03 | 5.51E-13 | 3.13E-02 | 20 |
| ENSG00000181915.3 | 10 | ADO | 64566377 | rs224090 | 10 | 64541319 | 0.37 | 0.05 | 4.22E-12 | 3.72E-05 | 20 |
| ENSG00000164308.12 | 5 | ERAP2 | 96233531 | rs2910686 | 5 | 96252589 | 0.07 | 0.01 | 4.46E-12 | 6.16E-02 | 20 |
| ENSG00000261338.1 | 2 | RP11-378A13.1 | 219121064 | rs736730 | 2 | 219120255 | -0.10 | 0.01 | 6.18E-12 | 8.20E-02 | 20 |
| ENSG00000189067.8 | 16 | LITAF | 11686045 | rs11641184 | 16 | 11704651 | -0.13 | 0.02 | 2.39E-11 | 1.85E-01 | 6 |
| ENSG00000131507.9 | 5 | NDFIP1 | 141511037 | rs449454 | 5 | 141533062 | 0.16 | 0.02 | 4.05E-11 | 9.42E-02 | 20 |
| ENSG00000234132.1 | 1 | RP11-168O16.2 | 201008212 | rs11807626 | 1 | 201000354 | 0.31 | 0.05 | 5.79E-11 | 3.72E-08 | 20 |
| ENSG00000232629.4 | 6 | HLA-DQB2 | 32727593 | rs3891175 | 6 | 32634467 | -0.10 | 0.02 | 1.16E-10 | 4.34E-09 | 20 |
| ENSG00000225697.6 | 3 | SLC26A6 | 48668041 | rs9868809 | 3 | 48681053 | 0.33 | 0.05 | 1.44E-10 | 1.97E-02 | 20 |
| ENSG00000004534.10 | 3 | RBM6 | 50046061 | rs4688755 | 3 | 50064424 | 0.20 | 0.03 | 2.04E-10 | 3.04E-05 | 20 |
| ENSG00000163466.11 | 2 | ARPC2 | 219100448 | rs6752254 | 2 | 219058743 | -0.22 | 0.04 | 3.19E-10 | 2.61E-02 | 20 |
| ENSG00000233006.2 | 5 | AC034220.3 | 131676159 | rs272886 | 5 | 131665840 | 0.14 | 0.02 | 4.21E-10 | 2.40E-10 | 20 |
| ENSG00000168610.10 | 17 | STAT3 | 40502964 | rs7211777 | 17 | 40534075 | 0.41 | 0.07 | 5.30E-10 | 1.85E-03 | 20 |
| ENSG00000175643.7 | 16 | RMI2 | 11394562 | rs9936459 | 16 | 11437779 | -0.14 | 0.02 | 6.26E-10 | 7.52E-03 | 20 |
| ENSG00000255389.1 | 6 | C6orf3 | 111922288 | rs33980500 | 6 | 111913262 | -0.17 | 0.03 | 1.81E-09 | 1.19E-02 | 20 |
| ENSG00000135926.8 | 2 | TMBIM1 | 219148112 | rs7559416 | 2 | 219150211 | -0.26 | 0.05 | 1.08E-08 | 4.42E-02 | 15 |
| ENSG00000167914.6 | 17 | GSDMA | 38126622 | rs4458030 | 17 | 38121706 | 0.15 | 0.03 | 1.20E-08 | 3.36E-03 | 20 |
| ENSG00000204528.3 | 6 | PSORS1C3 | 31143594 | rs2074488 | 6 | 31240431 | -0.09 | 0.02 | 1.70E-08 | 4.25E-06 | 20 |
| ENSG00000169220.13 | 5 | RGS14 | 176792220 | rs4976646 | 5 | 176788570 | -0.22 | 0.04 | 1.89E-08 | 4.79E-01 | 6 |
| ENSG00000176476.4 | 16 | CCDC101 | 28584179 | rs480400 | 16 | 28574615 | -0.54 | 0.10 | 2.07E-08 | 6.61E-03 | 20 |
| ENSG00000161395.8 | 17 | PGAP3 | 37840212 | rs903502 | 17 | 37829604 | -0.35 | 0.06 | 2.16E-08 | 5.65E-03 | 20 |
| ENSG00000197208.5 | 5 | SLC22A4 | 131655017 | rs10463891 | 5 | 131597392 | -0.35 | 0.06 | 2.98E-08 | 2.14E-04 | 20 |
| ENSG00000251417.1 | 16 | RP11-1348G14.4 | 28821623 | rs153106 | 16 | 28526897 | -0.26 | 0.05 | 3.59E-08 | 1.44E-01 | 20 |
| ENSG00000204257.10 | 6 | HLA-DMA | 32926630 | rs9276931 | 6 | 32928984 | -0.16 | 0.03 | 4.80E-08 | 6.77E-03 | 20 |
| ENSG00000241106.2 | 6 | HLA-DOB | 32782682 | rs2621326 | 6 | 32783896 | -0.11 | 0.02 | 7.45E-08 | 2.02E-02 | 20 |
| ENSG00000196502.7 | 16 | SULT1A1 | 28625924 | rs3743963 | 16 | 28604686 | -0.27 | 0.05 | 9.19E-08 | 3.67E-02 | 20 |
| ENSG00000164078.8 | 3 | MST1R | 49932856 | rs9821675 | 3 | 49902544 | -0.29 | 0.06 | 1.77E-07 | 1.65E-03 | 20 |

| | | | | | | | | | | | |
|---|---|---|---|---|---|---|---|---|---|---|---|
| ENSG00000173209.18 | 2 | AHSA2 | 61411445 | rs2167565 | 2 | 61476458 | -0.23 | 0.04 | 1.80E-07 | 1.41E-01 | 20 |
| ENSG00000132357.9 | 5 | CARD6 | 40850780 | rs1070444 | 5 | 40839644 | -0.27 | 0.05 | 2.21E-07 | 6.36E-04 | 12 |
| ENSG00000228078.1 | 6 | HLA-U | 29901970 | rs2844806 | 6 | 29933439 | -0.11 | 0.02 | 4.42E-07 | 8.28E-05 | 20 |
| ENSG00000185651.10 | 22 | UBE2L3 | 21950079 | rs738129 | 22 | 21971041 | 0.39 | 0.08 | 4.49E-07 | 1.68E-01 | 6 |
| ENSG00000187796.9 | 9 | CARD9 | 139263270 | rs11145988 | 9 | 139321545 | -0.26 | 0.05 | 5.11E-07 | 6.41E-02 | 11 |
| ENSG00000204338.4 | 6 | CYP21A1P | 31974821 | rs3129716 | 6 | 32657436 | 0.22 | 0.04 | 5.24E-07 | 8.73E-05 | 20 |
| ENSG00000054219.9 | 2 | LY75 | 160710563 | rs4664304 | 2 | 160794008 | -0.12 | 0.02 | 8.36E-07 | 8.36E-03 | 5 |
| ENSG00000116288.8 | 1 | PARK7 | 8029958 | rs172811 | 1 | 8039949 | -0.18 | 0.04 | 8.86E-07 | 9.74E-04 | 20 |
| ENSG00000145020.10 | 3 | AMT | 49457198 | rs6446275 | 3 | 49466871 | 0.19 | 0.04 | 1.09E-06 | 4.32E-14 | 20 |
| ENSG00000115234.6 | 2 | SNX17 | 27596692 | rs715326 | 2 | 27725761 | 0.11 | 0.02 | 1.32E-06 | 6.23E-04 | 9 |
| ENSG00000270820.1 | 2 | RP11-355B11.2 | 61704794 | rs811372 | 2 | 61429568 | -0.24 | 0.05 | 2.39E-06 | 1.03E-01 | 20 |
| ENSG00000160360.7 | 9 | GPSM1 | 139237931 | rs28635228 | 9 | 139245901 | -0.36 | 0.08 | 3.68E-06 | 7.17E-04 | 18 |
| ENSG00000108443.9 | 17 | RPS6KB1 | 57999186 | rs1292035 | 17 | 57989557 | 0.39 | 0.08 | 3.80E-06 | 5.87E-02 | 4 |
| ENSG00000159128.10 | 21 | IFNGR2 | 34813428 | rs2284553 | 21 | 34776695 | 0.24 | 0.05 | 4.41E-06 | 1.26E-01 | 3 |
| ENSG00000169885.5 | 1 | CALML6 | 1847499 | rs9660180 | 1 | 1723031 | -0.15 | 0.03 | 5.06E-06 | 6.38E-01 | 7 |
| ENSG00000244731.3 | 6 | C4A | 31960129 | rs6901158 | 6 | 32205942 | 0.11 | 0.02 | 5.71E-06 | 7.31E-03 | 20 |
| ENSG00000149485.12 | 11 | FADS1 | 61581944 | rs174550 | 11 | 61571478 | -0.25 | 0.06 | 6.13E-06 | 4.25E-01 | 3 |
| ENSG00000264968.1 | 17 | RP11-387H17.4 | 38089924 | rs4795402 | 17 | 38085385 | 0.10 | 0.02 | 6.61E-06 | 3.34E-06 | 20 |
| ENSG00000204301.5 | 6 | NOTCH4 | 32177232 | rs434841 | 6 | 32191041 | 0.15 | 0.03 | 6.95E-06 | 3.74E-02 | 20 |
| ENSG00000212978.5 | 2 | AC016747.3 | 61370418 | rs1177274 | 2 | 61368532 | -0.26 | 0.06 | 7.50E-06 | 4.21E-01 | 20 |

**Supplementary Table 7. SMR analysis in thyroid tissue**

| probeID | ProbeChr | Gene | Probe_bp | topSNP | topSNP_chr | topSNP_bp | b_SMR | se_SMR | p_SMR | p_HEIDI | nsnp_HEIDI |
|---|---|---|---|---|---|---|---|---|---|---|---|
| ENSG00000148384.11 | 9 | INPP5E | 139328672 | rs7628 | 9 | 139318071 | -0.23 | 0.02 | 2.00E-22 | 2.97E-06 | 20 |
| ENSG00000197083.7 | 5 | ZNF300P1 | 150318029 | rs12659118 | 5 | 150324622 | 0.12 | 0.01 | 3.45E-21 | 7.95E-08 | 16 |
| ENSG00000072682.14 | 5 | P4HA2 | 131579269 | rs7701414 | 5 | 131585958 | 0.23 | 0.03 | 1.29E-18 | 2.48E-06 | 20 |
| ENSG00000259982.1 | 16 | CDC37P1 | 28712238 | rs62034323 | 16 | 28536473 | 0.11 | 0.01 | 1.39E-14 | 2.99E-04 | 20 |
| ENSG00000119919.9 | 10 | NKX2-3 | 101294484 | rs10748781 | 10 | 101283330 | 0.35 | 0.05 | 6.26E-14 | 1.79E-02 | 13 |
| ENSG00000196126.6 | 6 | HLA-DRB1 | 32552085 | rs9271255 | 6 | 32580357 | 0.28 | 0.04 | 9.42E-14 | 2.36E-04 | 20 |
| ENSG00000229391.3 | 6 | HLA-DRB6 | 32524144 | rs9271640 | 6 | 32592200 | -0.12 | 0.02 | 3.26E-13 | 1.37E-03 | 20 |
| ENSG00000198502.5 | 6 | HLA-DRB5 | 32491592 | rs9270986 | 6 | 32574060 | 0.07 | 0.01 | 3.75E-13 | 2.64E-06 | 20 |
| ENSG00000178952.4 | 16 | TUFM | 28855730 | rs7187776 | 16 | 28857645 | 0.33 | 0.05 | 9.25E-13 | 5.52E-03 | 20 |
| ENSG00000271623.1 | 16 | RP11-435I10.5 | 28376337 | rs151174 | 16 | 28508069 | -0.14 | 0.02 | 2.63E-12 | 4.33E-03 | 20 |
| ENSG00000196301.3 | 6 | HLA-DRB9 | 32434437 | rs9271255 | 6 | 32580357 | -0.20 | 0.03 | 3.03E-12 | 2.13E-03 | 20 |
| ENSG00000164308.12 | 5 | ERAP2 | 96233531 | rs2910686 | 5 | 96252589 | 0.07 | 0.01 | 5.03E-12 | 8.29E-02 | 20 |
| ENSG00000261338.1 | 2 | RP11-378A13.1 | 219121064 | rs736730 | 2 | 219120255 | -0.08 | 0.01 | 5.58E-12 | 2.12E-02 | 20 |
| ENSG00000004534.10 | 3 | RBM6 | 50046061 | rs4688755 | 3 | 50064424 | 0.14 | 0.02 | 6.84E-12 | 4.19E-09 | 20 |
| ENSG00000225851.1 | 6 | HLA-S | 31349958 | rs9264942 | 6 | 31274380 | 0.15 | 0.02 | 2.43E-11 | 4.08E-10 | 20 |
| ENSG00000166949.11 | 15 | SMAD3 | 67421817 | rs72743461 | 15 | 67441750 | -0.22 | 0.03 | 3.94E-11 | 1.31E-01 | 16 |
| ENSG00000255389.1 | 6 | C6orf3 | 111922288 | rs33980500 | 6 | 111913262 | -0.15 | 0.02 | 4.28E-11 | 5.59E-03 | 20 |
| ENSG00000173531.11 | 3 | MST1 | 49724164 | rs11130213 | 3 | 49712297 | 0.53 | 0.08 | 8.31E-11 | 1.79E-03 | 18 |
| ENSG00000167914.6 | 17 | GSDMA | 38126622 | rs56326707 | 17 | 38130139 | 0.14 | 0.02 | 1.34E-10 | 4.96E-03 | 20 |
| ENSG00000197375.8 | 5 | SLC22A5 | 131718375 | rs11950562 | 5 | 131652529 | -0.47 | 0.07 | 1.85E-10 | 3.83E-03 | 20 |
| ENSG00000165689.12 | 9 | SDCCAG3 | 139300719 | rs10747043 | 9 | 139318310 | -0.31 | 0.05 | 2.75E-10 | 9.86E-07 | 20 |
| ENSG00000270424.1 | 16 | RP11-1348G14.6 | 28761598 | rs4787458 | 16 | 28531287 | 0.16 | 0.03 | 5.13E-10 | 9.25E-02 | 20 |
| ENSG00000251417.1 | 16 | RP11-1348G14.4 | 28821623 | rs12325113 | 16 | 28848668 | -0.22 | 0.03 | 6.37E-10 | 6.50E-03 | 20 |
| ENSG00000233006.2 | 5 | AC034220.3 | 131676159 | rs270601 | 5 | 131656997 | 0.11 | 0.02 | 1.47E-09 | 1.09E-09 | 20 |
| ENSG00000204338.4 | 6 | CYP21A1P | 31974821 | rs2856674 | 6 | 32659645 | 0.17 | 0.03 | 2.07E-09 | 6.80E-09 | 20 |
| ENSG00000161395.8 | 17 | PGAP3 | 37840212 | rs407307 | 17 | 37827163 | -0.21 | 0.04 | 2.21E-09 | 7.14E-06 | 20 |
| ENSG00000244731.3 | 6 | C4A | 31960129 | rs3135353 | 6 | 32392877 | 0.15 | 0.02 | 2.86E-09 | 2.14E-04 | 20 |
| ENSG00000196502.7 | 16 | SULT1A1 | 28625924 | rs75301646 | 16 | 28617885 | -0.13 | 0.02 | 3.48E-09 | 5.77E-06 | 20 |
| ENSG00000225697.6 | 3 | SLC26A6 | 48668041 | rs3821876 | 3 | 48668394 | 0.33 | 0.06 | 3.81E-09 | 7.71E-03 | 20 |

| | | | | | | | | | | | |
|---|---|---|---|---|---|---|---|---|---|---|---|
| ENSG00000175643.7 | 16 | RMI2 | 11394562 | rs243327 | 16 | 11353304 | -0.09 | 0.02 | 5.63E-09 | 1.20E-07 | 20 |
| ENSG00000142233.7 | 19 | NTN5 | 49170501 | rs516246 | 19 | 49206172 | 0.23 | 0.04 | 6.63E-09 | 5.52E-05 | 20 |
| ENSG00000234936.1 | 2 | AC010883.5 | 43458622 | rs13419550 | 2 | 43468636 | 0.22 | 0.04 | 9.25E-09 | 1.59E-02 | 20 |
| ENSG00000164068.11 | 3 | RNF123 | 49740422 | rs2247036 | 3 | 49882349 | -0.36 | 0.06 | 9.79E-09 | 8.19E-03 | 20 |
| ENSG00000213066.7 | 6 | FGFR1OP | 167439435 | rs720325 | 6 | 167467349 | 0.30 | 0.05 | 1.03E-08 | 3.99E-02 | 20 |
| ENSG00000231852.2 | 6 | CYP21A2 | 32007744 | rs1048709 | 6 | 31914935 | -0.26 | 0.04 | 1.05E-08 | 2.99E-05 | 20 |
| ENSG00000131435.8 | 5 | PDLIM4 | 131601255 | rs10076701 | 5 | 131596419 | -0.34 | 0.06 | 1.24E-08 | 9.34E-05 | 20 |
| ENSG00000026297.11 | 6 | RNASET2 | 167356832 | rs398278 | 6 | 167388169 | -0.37 | 0.07 | 1.28E-08 | 2.36E-01 | 20 |
| ENSG00000204528.3 | 6 | PSORS1C3 | 31143594 | rs2074488 | 6 | 31240431 | -0.08 | 0.01 | 1.48E-08 | 9.00E-06 | 20 |
| ENSG00000224431.1 | 5 | AC063976.7 | 131537164 | rs72793278 | 5 | 131561372 | 0.37 | 0.07 | 1.55E-08 | 2.19E-03 | 20 |
| ENSG00000173209.18 | 2 | AHSA2 | 61411445 | rs2167565 | 2 | 61476458 | -0.13 | 0.02 | 2.44E-08 | 1.02E-02 | 20 |
| ENSG00000085978.17 | 2 | ATG16L1 | 234161508 | rs56049444 | 2 | 234157688 | 0.70 | 0.13 | 2.82E-08 | 2.15E-02 | 20 |
| ENSG00000204257.10 | 6 | HLA-DMA | 32926630 | rs9276931 | 6 | 32928984 | -0.12 | 0.02 | 2.85E-08 | 6.61E-02 | 20 |
| ENSG00000127838.9 | 2 | PNKD | 219173315 | rs4672884 | 2 | 219182481 | -0.25 | 0.04 | 3.04E-08 | 8.86E-02 | 20 |
| ENSG00000260302.1 | 18 | RP11-973H7.1 | 12775286 | rs2542151 | 18 | 12779947 | 0.26 | 0.05 | 3.15E-08 | 3.45E-01 | 16 |
| ENSG00000233232.2 | 16 | NPIPB7 | 28474780 | rs151174 | 16 | 28508069 | -0.23 | 0.04 | 3.66E-08 | 6.66E-02 | 20 |
| ENSG00000176998.3 | 6 | HCG4 | 29759514 | rs1362070 | 6 | 29742299 | 0.07 | 0.01 | 3.81E-08 | 1.83E-02 | 20 |
| ENSG00000160766.10 | 1 | GBAP1 | 155190415 | rs2974929 | 1 | 155197268 | -0.15 | 0.03 | 4.09E-08 | 1.93E-01 | 20 |
| ENSG00000169231.9 | 1 | THBS3 | 155171543 | rs4072037 | 1 | 155162067 | 0.26 | 0.05 | 6.00E-08 | 8.38E-01 | 20 |
| ENSG00000169220.13 | 5 | RGS14 | 176792220 | rs4976646 | 5 | 176788570 | -0.27 | 0.05 | 6.48E-08 | 5.62E-01 | 6 |
| ENSG00000163466.11 | 2 | ARPC2 | 219100448 | rs10169718 | 2 | 219103580 | -0.36 | 0.07 | 8.55E-08 | 8.10E-02 | 20 |
| ENSG00000223534.1 | 6 | HLA-DQB1-AS1 | 32628319 | rs9270986 | 6 | 32574060 | 0.25 | 0.05 | 1.15E-07 | 3.81E-07 | 20 |
| ENSG00000115226.5 | 2 | FNDC4 | 27716431 | rs780094 | 2 | 27741237 | -0.27 | 0.05 | 1.27E-07 | 1.52E-01 | 3 |
| ENSG00000125520.9 | 20 | SLC2A4RG | 62373036 | rs2427532 | 20 | 62363817 | 0.44 | 0.08 | 1.60E-07 | 1.31E-02 | 20 |
| ENSG00000197146.2 | 6 | AL133458.1 | 167359002 | rs7760495 | 6 | 167506795 | -0.27 | 0.05 | 1.60E-07 | 1.27E-02 | 20 |
| ENSG00000008838.13 | 17 | MED24 | 38196409 | rs11078934 | 17 | 38189793 | 0.16 | 0.03 | 2.22E-07 | 9.38E-07 | 20 |
| ENSG00000160360.7 | 9 | GPSM1 | 139237931 | rs10870202 | 9 | 139257411 | -0.54 | 0.11 | 2.53E-07 | 1.90E-02 | 20 |
| ENSG00000137312.10 | 6 | FLOT1 | 30702998 | rs1264372 | 6 | 30769726 | -0.18 | 0.04 | 2.61E-07 | 5.16E-03 | 20 |
| ENSG00000204929.7 | 2 | AC074391.1 | 65987809 | rs11692813 | 2 | 65662306 | 0.07 | 0.01 | 3.38E-07 | 5.45E-04 | 20 |
| ENSG00000138111.10 | 10 | TMEM180 | 104228975 | rs3740415 | 10 | 104232716 | -0.07 | 0.01 | 3.48E-07 | 2.59E-02 | 9 |
| ENSG00000241106.2 | 6 | HLA-DOB | 32782682 | rs2621326 | 6 | 32783896 | -0.16 | 0.03 | 4.36E-07 | 1.45E-02 | 20 |
| ENSG00000232871.4 | 19 | SEC1P | 49162931 | rs516246 | 19 | 49206172 | 0.20 | 0.04 | 4.58E-07 | 4.11E-04 | 20 |

| | | | | | | | | | | | | |
|---|---|---|---|---|---|---|---|---|---|---|---|---|
| ENSG00000054219.9 | 2 | LY75 | 160710563 | rs4664304 | 2 | 160794008 | -0.10 | 0.02 | 4.64E-07 | 2.01E-03 | 6 |
| ENSG00000145020.10 | 3 | AMT | 49457198 | rs6446275 | 3 | 49466871 | 0.11 | 0.02 | 4.97E-07 | 6.30E-19 | 20 |
| ENSG00000091140.8 | 7 | DLD | 107551795 | rs6965674 | 7 | 107528102 | -0.58 | 0.12 | 5.22E-07 | 1.35E-04 | 20 |
| ENSG00000101246.15 | 20 | ARFRP1 | 62334707 | rs2315008 | 20 | 62343956 | 0.54 | 0.11 | 6.41E-07 | 1.70E-01 | 11 |
| ENSG00000145721.7 | 5 | LIX1 | 96453075 | rs38030 | 5 | 96353684 | -0.30 | 0.06 | 7.38E-07 | 1.08E-01 | 11 |
| ENSG00000204616.6 | 6 | TRIM31 | 30075778 | rs9261394 | 6 | 30064562 | 0.15 | 0.03 | 7.42E-07 | 2.05E-03 | 20 |
| ENSG00000145029.7 | 3 | NICN1 | 49463859 | rs6446275 | 3 | 49466871 | 0.12 | 0.02 | 9.13E-07 | 1.81E-14 | 20 |
| ENSG00000270210.1 | 2 | RP11-373D23.3 | 28649199 | rs6547850 | 2 | 28629343 | 0.21 | 0.04 | 1.05E-06 | 1.54E-01 | 4 |
| ENSG00000271889.1 | 2 | RP11-493E12.1 | 61383904 | rs3889717 | 2 | 61390791 | 0.14 | 0.03 | 1.34E-06 | 4.67E-01 | 20 |
| ENSG00000204301.5 | 6 | NOTCH4 | 32177232 | rs2071287 | 6 | 32170433 | 0.19 | 0.04 | 1.57E-06 | 3.97E-03 | 20 |
| ENSG00000164078.8 | 3 | MST1R | 49932856 | rs7372725 | 3 | 49790544 | -0.38 | 0.08 | 1.93E-06 | 6.45E-08 | 20 |
| ENSG00000235908.1 | 3 | RHOA-IT1 | 49403508 | rs7621003 | 3 | 49411404 | 0.31 | 0.07 | 2.35E-06 | 6.22E-05 | 17 |
| ENSG00000263080.1 | 16 | RP11-485G7.5 | 11437367 | rs243330 | 16 | 11350991 | -0.18 | 0.04 | 2.45E-06 | 5.15E-05 | 20 |
| ENSG00000230795.2 | 6 | HLA-K | 29895982 | rs12193110 | 6 | 29937104 | 0.06 | 0.01 | 3.20E-06 | 2.28E-03 | 20 |
| ENSG00000270820.1 | 2 | RP11-355B11.2 | 61704794 | rs778140 | 2 | 61682976 | 0.27 | 0.06 | 3.66E-06 | 6.25E-02 | 20 |
| ENSG00000204625.6 | 6 | HCG9 | 29944536 | rs3823376 | 6 | 29944184 | -0.17 | 0.04 | 3.85E-06 | 1.07E-04 | 20 |
| ENSG00000161180.6 | 22 | CCDC116 | 21989310 | rs7444 | 22 | 21976934 | -0.20 | 0.04 | 3.95E-06 | 2.18E-01 | 4 |
| ENSG00000112977.11 | 5 | DAP | 10720363 | rs267949 | 5 | 10743929 | -0.23 | 0.05 | 5.06E-06 | 5.49E-02 | 8 |
| ENSG00000160752.10 | 1 | FDPS | 155284066 | rs2297480 | 1 | 155279482 | 0.17 | 0.04 | 5.31E-06 | 1.31E-01 | 20 |
| ENSG00000220201.3 | 19 | ZGLP1 | 10418017 | rs73510898 | 19 | 10416444 | -0.52 | 0.11 | 5.39E-06 | 2.60E-06 | 7 |
| ENSG00000176022.3 | 1 | B3GALT6 | 1169025 | rs6697886 | 1 | 1173611 | -0.44 | 0.10 | 6.65E-06 | NA | NA |
| ENSG00000213760.6 | 6 | ATP6V1G2 | 31513432 | rs2239705 | 6 | 31513402 | -0.09 | 0.02 | 6.67E-06 | 3.33E-05 | 20 |

**Supplementary Table 8. SMR analysis in lymphoblastoid cell lines**

| probeID | ProbeChr | Gene | Probe_bp | topSNP | opSNP_ch | topSNP_bp | b_SMR | se_SMR | p_SMR | p_HEIDI | snp_HEIDI |
|---|---|---|---|---|---|---|---|---|---|---|---|
| ENSG00000237541.3 | 6 | HLA-DQA2 | 32709119 | rs660895 | 6 | 32577380 | -0.14 | 0.02 | 2.96E-18 | 3.25E-07 | 20 |
| ENSG00000187796.9 | 9 | CARD9 | 139268133 | s1087016 | 9 | 139316601 | -0.24 | 0.04 | 8.88E-11 | 1.47E-01 | 20 |
| ENSG00000184110.9 | 16 | EIF3C | 28699879 | s6203665 | 16 | 28850371 | 0.14 | 0.02 | 1.83E-10 | 7.18E-02 | 20 |
| ENSG00000212978.4 | 2 | ENSG00000212978 | 61372104 | rs1729662 | 2 | 61391305 | -0.09 | 0.02 | 1.94E-08 | 6.11E-02 | 20 |
| ENSG00000095794.14 | 10 | CREM | 35415719 | s1758300 | 10 | 35320915 | -0.21 | 0.04 | 3.71E-08 | 5.28E-01 | 18 |
| ENSG00000172057.4 | 17 | ORMDL3 | 38083854 | rs8067378 | 17 | 38051348 | -0.14 | 0.02 | 1.75E-17 | 2.65E-04 | 20 |
| ENSG00000196735.6 | 6 | HLA-DQA1 | 32595956 | rs9271100 | 6 | 32576478 | 0.17 | 0.02 | 2.08E-13 | 3.17E-08 | 20 |
| ENSG00000146535.8 | 7 | GNA12 | 2883958 | rs1182188 | 7 | 2869985 | -0.09 | 0.02 | 1.78E-07 | 2.26E-01 | 4 |
| ENSG00000164308.12 | 5 | ERAP2 | 96211643 | rs2910686 | 5 | 96252589 | 0.06 | 0.01 | 8.71E-12 | 1.85E-04 | 20 |
| ENSG00000148384.11 | 9 | INPP5E | 139334274 | s1329073 | 9 | 139330701 | -0.24 | 0.05 | 8.31E-07 | 8.16E-02 | 20 |
| ENSG00000135926.6 | 2 | TMBIM1 | 219157309 | rs2168704 | 2 | 219142492 | -0.16 | 0.03 | 2.85E-06 | 2.90E-01 | 20 |
| ENSG00000112977.11 | 5 | DAP | 10761384 | rs267949 | 5 | 10743929 | -0.09 | 0.02 | 3.89E-06 | 9.88E-02 | 8 |
| ENSG00000131435.8 | 5 | PDLIM4 | 131593364 | rs1979981 | 5 | 131601720 | -0.11 | 0.02 | 6.60E-11 | 3.33E-07 | 20 |
| ENSG00000232629.4 | 6 | HLA-DQB2 | 32731311 | rs9271100 | 6 | 32576478 | -0.21 | 0.03 | 1.79E-10 | 8.24E-06 | 20 |
| ENSG00000179344.11 | 6 | HLA-DQB1 | 32636160 | rs3891175 | 6 | 32634467 | 0.08 | 0.01 | 3.39E-10 | 7.89E-06 | 20 |
| ENSG00000175643.7 | 16 | RMI2 | 11343476 | rs9936459 | 16 | 11437779 | 0.09 | 0.01 | 5.14E-10 | 1.45E-06 | 20 |
| ENSG00000204267.8 | 6 | TAP2 | 32806557 | rs2621323 | 6 | 32788707 | 0.10 | 0.02 | 6.65E-09 | 6.60E-04 | 20 |
| ENSG00000169914.5 | 1 | OTUD3 | 20209006 | rs7541059 | 1 | 20211670 | 0.14 | 0.03 | 1.24E-08 | 2.62E-10 | 15 |
| ENSG00000237651.2 | 2 | C2orf74 | 61372243 | rs1729673 | 2 | 61389799 | 0.08 | 0.01 | 2.80E-08 | 3.25E-03 | 20 |
| ENSG00000162929.9 | 2 | KIAA1841 | 61293006 | rs1729653 | 2 | 61377008 | 0.09 | 0.02 | 6.47E-08 | 3.53E-03 | 20 |
| ENSG00000196126.6 | 6 | HLA-DRB1 | 32557625 | rs532098 | 6 | 32578052 | 0.11 | 0.02 | 1.73E-07 | 1.91E-12 | 15 |
| ENSG00000100311.12 | 22 | PDGFB | 39640756 | rs968451 | 22 | 39670851 | -0.21 | 0.04 | 3.10E-07 | 5.80E-04 | 19 |
| ENSG00000171522.5 | 5 | PTGER4 | 40679600 | s1044063 | 5 | 40490790 | 0.31 | 0.05 | 1.94E-09 | 2.87E-02 | 20 |
| ENSG00000108771.6 | 17 | DHX58 | 40264751 | rs730086 | 17 | 40271757 | 0.12 | 0.03 | 2.25E-06 | 2.21E-03 | 8 |
| ENSG00000173214.5 | 6 | KIAA1919 | 111580551 | s7156228 | 6 | 111555571 | 0.14 | 0.03 | 2.41E-06 | 3.18E-03 | 20 |
| ENSG00000100321.10 | 22 | SYNGR1 | 39745930 | rs2069235 | 22 | 39747780 | 0.05 | 0.01 | 3.15E-06 | 1.83E-12 | 20 |
| ENSG00000137310.7 | 6 | TCF19 | 31126319 | rs1966 | 6 | 31107733 | 0.12 | 0.03 | 5.37E-06 | 2.11E-03 | 15 |
| ENSG00000204531.9 | 6 | POU5F1 | 31148508 | rs1966 | 6 | 31107733 | 0.12 | 0.03 | 5.40E-06 | 7.96E-04 | 15 |

| | | | | | | | | | | | |
|---|---|---|---|---|---|---|---|---|---|---|---|
| ENSG00000204536.8 | 6 | CCHCR1 | 31126015 | rs1966 | 6 | 31107733 | 0.12 | 0.03 | 6.10E-06 | 3.23E-03 | 7 |
| ENSG00000205609.7 | 16 | EIF3CL | 28415200 | s5618613' | 16 | 28825953 | 0.13 | 0.02 | 5.23E-12 | 3.86E-02 | 20 |
| ENSG00000065989.9 | 19 | PDE4A | 10527449 | s1297810: | 19 | 10520514 | -0.14 | 0.03 | 7.51E-06 | 1.91E-02 | 13 |

**Supplemenantary Table 9. Meta-analysis of MR esti**

| direction | disease_group | IBD type |
|---|---|---|
| Forward | Breast cancer | CD |
| Forward | Breast cancer | IBD |
| Forward | Breast cancer | UC |
| Forward | CeD | CD |
| Forward | CeD | IBD |
| Forward | CeD | UC |
| Forward | MS | CD |
| Forward | MS | IBD |
| Forward | MS | UC |
| Forward | PBC | CD |
| Forward | PBC | IBD |
| Forward | PBC | UC |
| Forward | RA | CD |
| Forward | RA | IBD |
| Forward | RA | UC |
| Forward | SLE | CD |
| Forward | SLE | IBD |
| Forward | SLE | UC |
| Forward | T1D | CD |
| Forward | T1D | IBD |
| Forward | T1D | UC |
| Forward | T2D | CD |
| Forward | T2D | IBD |
| Forward | T2D | UC |
| Inverse | Breast cancer | CD |
| Inverse | Breast cancer | UC |
| Inverse | CeD | CD |
| Inverse | CeD | IBD |
| Inverse | CeD | UC |
| Inverse | MS | CD |
| Inverse | MS | IBD |
| Inverse | MS | UC |
| Inverse | RA | CD |
| Inverse | RA | IBD |
| Inverse | RA | UC |
| Inverse | SLE | CD |
| Inverse | SLE | IBD |
| Inverse | SLE | UC |
| Inverse | T1D | CD |
| Inverse | T1D | IBD |
| Inverse | T1D | UC |
| Inverse | T2D | CD |
| Inverse | T2D | IBD |
| Inverse | T2D | UC |

**imates across data source in both directions**

| included_traits |
|---|
| Breast cancer_S1; Breast cancer_S2 |
| Breast cancer_S1; Breast cancer_S2 |
| Breast cancer_S1; Breast cancer_S2 |
| CeD_S3; CeD_S5; CeD_S2; CeD_S1; CeD_S6; CeD_S4 |
| CeD_S3; CeD_S5; CeD_S2; CeD_S1; CeD_S6; CeD_S4 |
| CeD_S3; CeD_S5; CeD_S2; CeD_S1; CeD_S6; CeD_S4 |
| MS_S1; MS_S2; MS_S3; MS_S4 |
| MS_S1; MS_S2; MS_S3; MS_S4 |
| MS_S1; MS_S2; MS_S3; MS_S4 |
| PBC_S1; PBC_S2 |
| PBC_S1; PBC_S2 |
| PBC_S1; PBC_S2 |
| RA_S4; RA_S6; RA_S2; RA_S1; RA_S3; RA_S5 |
| RA_S4; RA_S6; RA_S2; RA_S1; RA_S3; RA_S5 |
| RA_S4; RA_S6; RA_S2; RA_S1; RA_S3; RA_S5 |
| SLE_S1; SLE_S2 |
| SLE_S1; SLE_S2 |
| SLE_S1; SLE_S2 |
| T1D_S3; T1D_S2; T1D_S4; T1D_S1 |
| T1D_S3; T1D_S2; T1D_S4; T1D_S1 |
| T1D_S3; T1D_S2; T1D_S4; T1D_S1 |
| T2D_S3; T2D_S5; T2D_S4; T2D_S1; T2D_S2; T2D_S6 |
| T2D_S3; T2D_S5; T2D_S4; T2D_S1; T2D_S2; T2D_S6 |
| T2D_S3; T2D_S5; T2D_S4; T2D_S1; T2D_S2; T2D_S6 |
| Breast cancer_S1; Breast cancer_S2 |
| Breast cancer_S1; Breast cancer_S2 |
| CeD_S3; CeD_S5; CeD_S1; CeD_S6; CeD_S4 |
| CeD_S3; CeD_S5; CeD_S1; CeD_S6; CeD_S4 |
| CeD_S3; CeD_S5; CeD_S1; CeD_S6; CeD_S4 |
| MS_S1; MS_S2; MS_S3; MS_S4 |
| MS_S1; MS_S2; MS_S3; MS_S4 |
| MS_S1; MS_S2; MS_S3; MS_S4 |
| RA_S4; RA_S6; RA_S2; RA_S1; RA_S3; RA_S5 |
| RA_S4; RA_S6; RA_S2; RA_S1; RA_S3; RA_S5 |
| RA_S4; RA_S6; RA_S2; RA_S1; RA_S3; RA_S5 |
| SLE_S1; SLE_S2 |
| SLE_S1; SLE_S2 |
| SLE_S1; SLE_S2 |
| T1D_S3; T1D_S2; T1D_S4 |
| T1D_S3; T1D_S2; T1D_S4 |
| T1D_S3; T1D_S2; T1D_S4 |
| T2D_S3; T2D_S5; T2D_S4; T2D_S1; T2D_S2; T2D_S6 |
| T2D_S3; T2D_S5; T2D_S4; T2D_S1; T2D_S2; T2D_S6 |
| T2D_S3; T2D_S5; T2D_S4; T2D_S1; T2D_S2; T2D_S6 |

| included_gwas_ids |
| --- |
| ebi-a-GCST004988; ebi-a-GCST007236 |
| ebi-a-GCST004988; ebi-a-GCST007236 |
| ebi-a-GCST004988; ebi-a-GCST007236 |
| ieu-a-1058; ieu-a-1060; ebi-a-GCST005523; ebi-a-GCST000612; ieu-a-276; ieu-a-1059 |
| ieu-a-1058; ieu-a-1060; ebi-a-GCST005523; ebi-a-GCST000612; ieu-a-276; ieu-a-1059 |
| ieu-a-1058; ieu-a-1060; ebi-a-GCST005523; ebi-a-GCST000612; ieu-a-276; ieu-a-1059 |
| ieu-a-1025; ebi-a-GCST005531; ieu-a-1024; ieu-b-18 |
| ieu-a-1025; ebi-a-GCST005531; ieu-a-1024; ieu-b-18 |
| ieu-a-1025; ebi-a-GCST005531; ieu-a-1024; ieu-b-18 |
| ebi-a-GCST003129; ebi-a-GCST005581 |
| ebi-a-GCST003129; ebi-a-GCST005581 |
| ebi-a-GCST003129; ebi-a-GCST005581 |
| ieu-a-832; bbj-a-73; ebi-a-GCST002318; ebi-a-GCST000679; ebi-a-GCST005569; ieu-a-834 |
| ieu-a-832; bbj-a-73; ebi-a-GCST002318; ebi-a-GCST000679; ebi-a-GCST005569; ieu-a-834 |
| ieu-a-832; bbj-a-73; ebi-a-GCST002318; ebi-a-GCST000679; ebi-a-GCST005569; ieu-a-834 |
| ebi-a-GCST003156; ieu-a-815 |
| ebi-a-GCST003156; ieu-a-815 |
| ebi-a-GCST003156; ieu-a-815 |
| ebi-a-GCST90000529; ebi-a-GCST010681; finn-b-E4_DM1; ebi-a-GCST005536 |
| ebi-a-GCST90000529; ebi-a-GCST010681; finn-b-E4_DM1; ebi-a-GCST005536 |
| ebi-a-GCST90000529; ebi-a-GCST010681; finn-b-E4_DM1; ebi-a-GCST005536 |
| ebi-a-GCST007517; finn-b-T2D; finn-b-E4_DM2; ebi-a-GCST005047; ebi-a-GCST006867; ebi-a-G |
| ebi-a-GCST007517; finn-b-T2D; finn-b-E4_DM2; ebi-a-GCST005047; ebi-a-GCST006867; ebi-a-G |
| ebi-a-GCST007517; finn-b-T2D; finn-b-E4_DM2; ebi-a-GCST005047; ebi-a-GCST006867; ebi-a-G |
| ebi-a-GCST004988; ebi-a-GCST007236 |
| ebi-a-GCST004988; ebi-a-GCST007236 |
| ieu-a-1058; ieu-a-1060; ebi-a-GCST000612; ieu-a-276; ieu-a-1059 |
| ieu-a-1058; ieu-a-1060; ebi-a-GCST000612; ieu-a-276; ieu-a-1059 |
| ieu-a-1058; ieu-a-1060; ebi-a-GCST000612; ieu-a-276; ieu-a-1059 |
| ieu-a-1025; ebi-a-GCST005531; ieu-a-1024; ieu-b-18 |
| ieu-a-1025; ebi-a-GCST005531; ieu-a-1024; ieu-b-18 |
| ieu-a-1025; ebi-a-GCST005531; ieu-a-1024; ieu-b-18 |
| ieu-a-832; bbj-a-73; ebi-a-GCST002318; ebi-a-GCST000679; ebi-a-GCST005569; ieu-a-834 |
| ieu-a-832; bbj-a-73; ebi-a-GCST002318; ebi-a-GCST000679; ebi-a-GCST005569; ieu-a-834 |
| ieu-a-832; bbj-a-73; ebi-a-GCST002318; ebi-a-GCST000679; ebi-a-GCST005569; ieu-a-834 |
| ebi-a-GCST003156; ieu-a-815 |
| ebi-a-GCST003156; ieu-a-815 |
| ebi-a-GCST003156; ieu-a-815 |
| ebi-a-GCST90000529; ebi-a-GCST010681; finn-b-E4_DM1 |
| ebi-a-GCST90000529; ebi-a-GCST010681; finn-b-E4_DM1 |
| ebi-a-GCST90000529; ebi-a-GCST010681; finn-b-E4_DM1 |
| ebi-a-GCST007517; finn-b-T2D; finn-b-E4_DM2; ebi-a-GCST005047; ebi-a-GCST006867; ebi-a-G |
| ebi-a-GCST007517; finn-b-T2D; finn-b-E4_DM2; ebi-a-GCST005047; ebi-a-GCST006867; ebi-a-G |
| ebi-a-GCST007517; finn-b-T2D; finn-b-E4_DM2; ebi-a-GCST005047; ebi-a-GCST006867; ebi-a-G |

| category | n_dataset | pooled_logOR | pooled_SE | pooled_OR |
|---|---|---|---|---|
| Neoplasms | 2 | 0.224293302565066 | 0.24061892835395 | 1.25143801558527 |
| Neoplasms | 2 | -0.000275151 | 0.00509476261132 | 0.999724886571578 |
| Neoplasms | 2 | 0.00013881276383787 | 0.00509612982718 | 1.00013882239878 |
| Digestive | 6 | 0.0695437961713526 | 0.03933138092758 | 1.07201901050494 |
| Digestive | 6 | 0.0580811962743033 | 0.03279833784168 | 1.05980104411312 |
| Digestive | 6 | 0.0717688753539396 | 0.03089860087168 | 1.07440699342886 |
| Mental and Nervous | 4 | -0.010289346 | 0.01728431057182 | 0.989763408617786 |
| Mental and Nervous | 4 | 0.0826732269921136 | 0.02976583581481 | 1.08618681401774 |
| Mental and Nervous | 4 | 0.0639469485137318 | 0.05196594400201 | 1.06603584247546 |
| Digestive | 2 | 0.0908561751519577 | 0.01369808997350 | 1.09511148968782 |
| Digestive | 2 | 0.107557908125072 | 0.03139016649298 | 1.11355534278157 |
| Digestive | 2 | 0.0886650821397949 | 0.05026382408382 | 1.09271462539045 |
| Musculoskeletal | 6 | -0.116404373 | 0.04091806489508 | 0.890115212036749 |
| Musculoskeletal | 6 | 0.0205008564282223 | 0.01837060543102 | 1.02071244240976 |
| Musculoskeletal | 6 | 0.110024784706938 | 0.01589587429830 | 1.11630573742657 |
| Musculoskeletal | 2 | -0.063305255 | 0.02640270879528 | 0.938656900564467 |
| Musculoskeletal | 2 | -0.092088056 | 0.01000993676212 | 0.912024836988181 |
| Musculoskeletal | 2 | -0.099109291 | 0.02150401065765 | 0.905643723943931 |
| Endocrine, nutriti | 4 | -0.019989509 | 0.04183455848819 | 0.980208956494008 |
| Endocrine, nutriti | 4 | -0.036656999 | 0.02967178133796 | 0.964006733827335 |
| Endocrine, nutriti | 4 | 0.0141199169835799 | 0.01172470857657 | 1.01422007385844 |
| Endocrine, nutriti | 6 | -0.069731295 | 0.04636589494901 | 0.932644392202941 |
| Endocrine, nutriti | 6 | -0.048653211 | 0.02390290200407 | 0.952511392449185 |
| Endocrine, nutriti | 6 | -0.086530166 | 0.02166554956965 | 0.917107882685002 |
| Neoplasms | 2 | -1.491115455 | 1.50284025337093 | 0.225121402550058 |
| Neoplasms | 2 | 1.9837286288818 | 1.96543142388102 | 7.26979889521696 |
| Digestive | 5 | 0.069392839332911 | 0.04310380044046 | 1.07185719411832 |
| Digestive | 5 | 0.211038428227768 | 0.03144708054403 | 1.23495981146647 |
| Digestive | 5 | 0.142678787826774 | 0.03383748461296 | 1.15335926912207 |
| Mental and Nervous | 4 | 0.0611255420870077 | 0.02267047130878 | 1.06303236111159 |
| Mental and Nervous | 4 | 0.0707384450067261 | 0.02135133111483 | 1.07330046205724 |
| Mental and Nervous | 4 | 0.0602653752861186 | 0.02197747234110 | 1.06211836911543 |
| Musculoskeletal | 6 | -0.024631766 | 0.00755234628489 | 0.975669120089483 |
| Musculoskeletal | 6 | 0.0057564605803004 | 0.01089919952648 | 1.00577306083713 |
| Musculoskeletal | 6 | 0.026748041156863 | 0.00724924231562 | 1.02710898096802 |
| Musculoskeletal | 2 | -0.020202707 | 0.02192580385128 | 0.98 |
| Musculoskeletal | 2 | -0.095195051 | 0.17085136629956 | 0.90919557772531 |
| Musculoskeletal | 2 | 0.0603245833975455 | 0.02727437345685 | 1.06218125699989 |
| Endocrine, nutriti | 3 | 0.00350463547356767 | 0.01029606495887 | 1.00351078388902 |
| Endocrine, nutriti | 3 | 0.0248294569445906 | 0.02333652110208 | 1.02514027506078 |
| Endocrine, nutriti | 3 | 0.0222728909649704 | 0.01239740236799 | 1.02252278362966 |
| Endocrine, nutriti | 6 | 0.00474769425740205 | 0.00342618114469 | 1.00475898241495 |
| Endocrine, nutriti | 6 | -0.003728065 | 0.00810333807445 | 0.996278875397489 |
| Endocrine, nutriti | 6 | -0.003522152 | 0.00790127319664 | 0.99648404303712 |

| lower_CI | upper_CI | p_value | I2 | heterogeneity_p |
|---|---|---|---|---|
| 0.780890927 | 2.00552606346857 | 0.351 | 92.96 | 0.000163 |
| 0.989791577 | 1.00975788413632 | 0.957 | 0 | 0.318 |
| 0.990198746 | 1.01017868113922 | 0.978 | 0 | 0.577 |
| 0.992482686 | 1.15792927573333 | 0.077 | 98.16 | 1.5e-56 |
| 0.993815656 | 1.13016759789088 | 0.0766 | 97.71 | 3.42e-45 |
| 1.011270658 | 1.14148509904156 | 0.0202 | 96.45 | 1.25e-28 |
| 0.956794547 | 1.0238682980013 | 0.552 | 78.44 | 0.00302 |
| 1.0246306321 | 1.15144107339231 | 0.00548 | 96.52 | 1.45e-18 |
| 0.962803149 | 1.18033724531388 | 0.218 | 97.27 | 1.2e-23 |
| 1.066100840 | 1.12491157379948 | 3.29e-11 | 47.89 | 0.166 |
| 1.047109155 | 1.18421799208491 | 0.000611 | 90.43 | 0.00123 |
| 0.990196359 | 1.20584694303442 | 0.0777 | 95.41 | 3.07e-06 |
| 0.821516069 | 0.964442595558877 | 0.00444 | 96.13 | 3.59e-26 |
| 0.984614061 | 1.05813427891633 | 0.264 | 88.98 | 1.21e-08 |
| 1.082062423 | 1.15163272729685 | 4.47e-12 | 78.56 | 0.000293 |
| 0.891317508 | 0.988510568066095 | 0.0165 | 71.85 | 0.0595 |
| 0.894305775 | 0.930094970077926 | 3.59e-20 | 0 | 0.66 |
| 0.8682660067 | 0.944630502996706 | 4.05e-06 | 58.24 | 0.122 |
| 0.903042877 | 1.06396896844969 | 0.633 | 96.5 | 1.86e-18 |
| 0.909542375 | 1.02173247597527 | 0.217 | 96.98 | 1.99e-21 |
| 0.991178626 | 1.0377971541925 | 0.228 | 69.2 | 0.0209 |
| 0.851625460 | 1.02137101675915 | 0.133 | 72.09 | 0.00306 |
| 0.908915726 | 0.998198101823067 | 0.0418 | 31.28 | 0.201 |
| 0.878978673 | 0.956891098123946 | 6.5e-05 | 0 | 0.625 |
| 0.011835138 | 4.28213381903183 | 0.321 | 99.88 | 3.27e-185 |
| 0.154352402 | 342.39814262919 | 0.313 | 99.92 | 1.4e-284 |
| 0.9850226831 | 1.16634658701272 | 0.107 | 82 | 0.000181 |
| 1.1611398481 | 1.31347291059049 | 1.93e-11 | 39.02 | 0.161 |
| 1.0793481757 | 1.23244531609215 | 2.48e-05 | 52.74 | 0.076 |
| 1.016831498 | 1.11133241114013 | 0.00701 | 87.35 | 2.86e-05 |
| 1.029311195 | 1.11916968086036 | 0.000923 | 80.69 | 0.00141 |
| 1.017338119 | 1.10886971424507 | 0.0061 | 80.02 | 0.0018 |
| 0.961333048 | 0.990218980754763 | 0.00111 | 28.27 | 0.223 |
| 0.984515171 | 1.02748995548031 | 0.597 | 55.6 | 0.0465 |
| 1.0126184757 | 1.04180684440526 | 0.000224 | 0 | 0.811 |
| 0.938777029 | 1.02303312656685 | 0.357 | 0 | 1 |
| 0.650467639 | 1.27083431663802 | 0.577 | 93.81 | 5.85e-05 |
| 1.006890442 | 1.12050822574857 | 0.027 | 0 | 0.83 |
| 0.9834626171 | 1.02396763819524 | 0.734 | 0 | 0.662 |
| 0.979306976 | 1.0731186531659 | 0.287 | 74.22 | 0.0207 |
| 0.997976033 | 1.04767329889846 | 0.0724 | 0 | 0.613 |
| 0.9980343137 | 1.0115289613542 | 0.166 | 9.79 | 0.353 |
| 0.980580429 | 1.01222864325438 | 0.645 | 72 | 0.00313 |
| 0.9811708771 | 1.01203620204736 | 0.656 | 70.45 | 0.00465 |

| pooled_OR_CI |
|---|
| 1.251 [0.781, 2.006] |
| 1.000 [0.990, 1.010] |
| 1.000 [0.990, 1.010] |
| 1.072 [0.992, 1.158] |
| 1.060 [0.994, 1.130] |
| 1.074 [1.011, 1.141] |
| 0.990 [0.957, 1.024] |
| 1.086 [1.025, 1.151] |
| 1.066 [0.963, 1.180] |
| 1.095 [1.066, 1.125] |
| 1.114 [1.047, 1.184] |
| 1.093 [0.990, 1.206] |
| 0.890 [0.822, 0.964] |
| 1.021 [0.985, 1.058] |
| 1.116 [1.082, 1.152] |
| 0.939 [0.891, 0.989] |
| 0.912 [0.894, 0.930] |
| 0.906 [0.868, 0.945] |
| 0.980 [0.903, 1.064] |
| 0.964 [0.910, 1.022] |
| 1.014 [0.991, 1.038] |
| 0.933 [0.852, 1.021] |
| 0.953 [0.909, 0.998] |
| 0.917 [0.879, 0.957] |
| 0.225 [0.012, 4.282] |
| 7.270 [0.154, 342.398] |
| 1.072 [0.985, 1.166] |
| 1.235 [1.161, 1.313] |
| 1.153 [1.079, 1.232] |
| 1.063 [1.017, 1.111] |
| 1.073 [1.029, 1.119] |
| 1.062 [1.017, 1.109] |
| 0.976 [0.961, 0.990] |
| 1.006 [0.985, 1.027] |
| 1.027 [1.013, 1.042] |
| 0.980 [0.939, 1.023] |
| 0.909 [0.650, 1.271] |
| 1.062 [1.007, 1.121] |
| 1.004 [0.983, 1.024] |
| 1.025 [0.979, 1.073] |
| 1.023 [0.998, 1.048] |
| 1.005 [0.998, 1.012] |
| 0.996 [0.981, 1.012] |
| 0.996 [0.981, 1.012] |

**mentary Table 10. Forward direction MR analysis F-st**

| Outcome | SNP | F_statistic |
|---|---|---|
| IBD | rs2261033 | 1309.70595 |
| IBD | rs34521355 | 619.4259078 |
| IBD | rs3891175 | 340.6548467 |
| IBD | rs17312661 | 329.0355684 |
| IBD | rs2074475 | 251.7351789 |
| IBD | rs27529 | 208.270196 |
| IBD | rs4672505 | 207.3744313 |
| IBD | rs9380120 | 202.8458428 |
| IBD | rs7453920 | 187.3840192 |
| IBD | rs2523443 | 163.8322774 |
| IBD | rs7746199 | 153.3312682 |
| IBD | rs17194627 | 146.3295001 |
| IBD | rs3857546 | 143.5570397 |
| IBD | rs7771335 | 111.647554 |
| IBD | rs362521 | 104.9229818 |
| IBD | rs9262222 | 90.92558971 |
| IBD | rs2836883 | 69.83216632 |
| IBD | rs9393646 | 68.78743718 |
| IBD | rs41299637 | 63.25861617 |
| IBD | rs6600247 | 62.56710603 |
| IBD | rs9901869 | 60.88986422 |
| IBD | rs2239842 | 59.29114202 |
| IBD | rs11190133 | 56.79210306 |
| IBD | rs4129267 | 53.00824023 |
| IBD | rs11624293 | 41.03960526 |
| IBD | rs1041926 | 40.96172978 |
| IBD | rs1860545 | 39.82087767 |
| IBD | rs35164067 | 39.41475927 |
| IBD | rs2927613 | 38.00117864 |
| IBD | rs12615545 | 37.26600201 |
| IBD | rs1801274 | 36.74091877 |
| IBD | rs1250550 | 36.5824539 |
| IBD | rs7191548 | 34.00021582 |
| IBD | rs7282490 | 33.76681929 |
| IBD | rs1128905 | 33.54905971 |
| IBD | rs6915101 | 33.53582202 |
| IBD | rs4676410 | 32.86114726 |
| IBD | rs11065898 | 29.83296349 |
| IBD | rs6881706 | 122.0334473 |
| IBD | rs56375023 | 115.5625 |
| IBD | rs7224129 | 88.09633733 |
| IBD | rs848 | 67.78025896 |
| IBD | rs12365699 | 57.60420776 |
| IBD | rs2228145 | 41.35820452 |
| IBD | rs6011033 | 40.11111111 |
| IBD | rs249677 | 38.48204539 |
| IBD | rs45613035 | 37.73469388 |

| | | |
|---|---|---|
| IBD | rs4845604 | 34.16680839 |
| IBD | rs17664743 | 33.34655822 |
| IBD | rs12740374 | 74.62841521 |
| IBD | rs10774625 | 55.96924896 |
| IBD | rs11591147 | 42.23149781 |
| IBD | rs943689 | 15331.36608 |
| IBD | rs174535 | 14925.55714 |
| IBD | rs11742240 | 13095.58337 |
| IBD | rs6866614 | 12003.57035 |
| IBD | rs4771332 | 10973.31764 |
| IBD | rs72743461 | 8550.412667 |
| IBD | rs17454584 | 8543.773292 |
| IBD | rs17668708 | 6537.486118 |
| IBD | rs57585717 | 5200.061381 |
| IBD | rs62626322 | 4884.227913 |
| IBD | rs4795399 | 12651.68545 |
| IBD | rs11065979 | 8666.64348 |
| IBD | rs7100984 | 7384.392732 |
| IBD | rs9391997 | 6447.57544 |
| IBD | rs912425 | 6359.282939 |
| IBD | rs10786050 | 6096.274685 |
| IBD | rs449454 | 6010.474415 |
| IBD | rs705700 | 5925.1174 |
| IBD | rs10995245 | 5865.850642 |
| IBD | rs35570272 | 5785.519507 |
| IBD | rs3997849 | 5673.511839 |
| IBD | rs12365699 | 5421.749137 |
| IBD | rs4917131 | 5333.142509 |
| IBD | rs11236797 | 5009.390268 |
| IBD | rs4845604 | 4729.476999 |
| IBD | rs55743914 | 4442.172618 |
| IBD | rs2738783 | 4177.728664 |
| IBD | rs72743461 | 3725.129751 |
| IBD | rs9275602 | 3457.002739 |
| IBD | rs17611877 | 2190.374927 |
| IBD | rs4705869 | 2030.281908 |
| IBD | rs45613035 | 1816.028893 |
| IBD | rs10118244 | 1220.032642 |
| IBD | rs4065275 | 60.0625 |
| IBD | rs4851582 | 37.83680368 |
| IBD | rs17228058 | 31.19476369 |
| IBD | rs56375023 | 180.8381491 |
| IBD | rs848 | 129.3111611 |
| IBD | rs943689 | 105.8320661 |
| IBD | rs35570272 | 57.79741471 |
| IBD | rs17454584 | 57.42021622 |
| IBD | rs2229094 | 46.45601899 |
| IBD | rs2296618 | 41.30204088 |
| IBD | rs11742240 | 37.99541679 |
| IBD | rs6922431 | 37.29855251 |
| IBD | rs12365699 | 37.04297066 |

| | | |
|---|---|---|
| IBD | rs3130196 | 36.07438813 |
| IBD | rs1800797 | 35.8317146 |
| IBD | rs2255088 | 35.22255884 |
| IBD | rs11042902 | 33.78044012 |
| IBD | rs7219483 | 33.40236018 |
| IBD | rs2275806 | 30.53114472 |
| IBD | rs10912564 | 30.48300256 |
| IBD | rs4787426 | 30.05105399 |
| IBD | rs11679146 | 75.00572944 |
| IBD | rs12949100 | 70.13310017 |
| IBD | rs10900809 | 45.65419851 |
| IBD | rs72743461 | 40.34409125 |
| IBD | rs34972832 | 34.07344531 |
| IBD | rs3781094 | 32.19627276 |
| IBD | rs76830965 | 751.4926184 |
| IBD | rs2523554 | 95.59110318 |
| IBD | rs17552787 | 73.16962599 |
| IBD | rs17282175 | 62.12034288 |
| IBD | rs913678 | 56.484542 |
| IBD | rs9261425 | 48.62943421 |
| IBD | rs436857 | 42.88770149 |
| IBD | rs10513548 | 32.5228789 |
| IBD | rs2305479 | 187.7155729 |
| IBD | rs3771180 | 86.39709541 |
| IBD | rs17293632 | 64.6798294 |
| IBD | rs2155219 | 62.33605557 |
| IBD | rs20541 | 59.28971257 |
| IBD | rs2596464 | 54.22461405 |
| IBD | rs2325291 | 51.14465884 |
| IBD | rs2855812 | 39.80707175 |
| IBD | rs7705042 | 37.63508136 |
| IBD | rs2523716 | 32.21965276 |
| IBD | rs241429 | 30.72053745 |
| IBD | rs12740374 | 137.8754864 |
| IBD | rs10774625 | 99.02658713 |
| IBD | rs11591147 | 67.90742618 |
| IBD | rs2681472 | 50.46833153 |
| IBD | rs72743461 | 43.38995462 |
| IBD | rs17115100 | 37.29062542 |
| IBD | rs13402621 | 37.12083809 |
| IBD | rs748431 | 30.2782117 |
| IBD | rs2160203 | 101.5586578 |
| IBD | rs20541 | 52.13554725 |
| IBD | rs879606 | 36.57155192 |
| IBD | rs10806425 | 34.9088659 |
| IBD | rs62322662 | 33.49912916 |
| IBD | rs6010620 | 63.17508918 |
| IBD | rs17513531 | 34.8043543 |
| IBD | rs61839660 | 33.13625904 |
| IBD | rs2476601 | 695.271424 |
| IBD | rs3184504 | 255.6445679 |

| | | |
|---|---|---|
| IBD | rs11066320 | 212.835679 |
| IBD | rs2596503 | 156.4693752 |
| IBD | rs11571297 | 145.3073015 |
| IBD | rs9277557 | 128.376993 |
| IBD | rs28780083 | 77.82161548 |
| IBD | rs10905669 | 66.97251145 |
| IBD | rs1990760 | 56.08345679 |
| IBD | rs229541 | 54.76 |
| IBD | rs13203715 | 50.30862245 |
| IBD | rs4073285 | 39.33471172 |
| IBD | rs75438091 | 37.34567901 |
| IBD | rs2293158 | 33.0625 |
| IBD | rs7205474 | 30.53515161 |
| IBD | rs9392026 | 100.500894 |
| IBD | rs3778607 | 81.22837799 |
| IBD | rs3087243 | 76.72820712 |
| IBD | rs72928038 | 71.13282576 |
| IBD | rs162298 | 54.06345131 |
| IBD | rs2049218 | 46.42533891 |
| IBD | rs2476601 | 39.76934144 |
| IBD | rs4682103 | 32.62425174 |
| IBD | rs10994397 | 38.50900594 |
| IBD | rs7296288 | 32.95192315 |
| IBD | rs12576775 | 30.86928367 |
| IBD | rs7258465 | 120.6668962 |
| IBD | rs7017073 | 58.16604444 |
| IBD | rs9952980 | 54.07905325 |
| IBD | rs4971059 | 43.890625 |
| IBD | rs3749971 | 33.78515625 |
| IBD | rs61744064 | 32.92125773 |
| IBD | rs17426269 | 32.06719849 |
| IBD | rs666930 | 30.33736458 |
| IBD | rs414851 | 10679.99029 |
| IBD | rs17076598 | 8944.242115 |
| IBD | rs3759333 | 7956.728479 |
| IBD | rs26433 | 7862.218435 |
| IBD | rs3800393 | 6135.983387 |
| IBD | rs12606193 | 4627.748927 |
| IBD | rs11864719 | 4513.373015 |
| IBD | rs17450969 | 4142.522261 |
| IBD | rs592719 | 3790.1959 |
| IBD | rs6880335 | 2343.940734 |
| IBD | rs2109897 | 2066.055573 |
| IBD | rs12650313 | 1930.49442 |
| IBD | rs10519288 | 1613.473299 |
| IBD | rs16839999 | 578.9153819 |
| IBD | rs17238590 | 527.3553142 |
| IBD | rs16941631 | 341.6692449 |
| IBD | rs72905734 | 32.99332008 |
| IBD | rs4468572 | 65.71142838 |
| IBD | rs2681472 | 42.76643653 |

| | | |
|---|---|---|
| IBD | rs11065979 | 40.54015268 |
| IBD | rs11191416 | 34.33208586 |
| IBD | rs515135 | 30.64899745 |
| IBD | rs599839 | 39.75093937 |
| IBD | rs1122608 | 37.37777487 |
| IBD | rs2306374 | 30.49755064 |
| IBD | rs11591147 | 45.84997549 |
| IBD | rs653178 | 45.11184822 |
| IBD | rs12705390 | 42.67808621 |
| IBD | rs11591147 | 42.77237626 |
| IBD | rs653178 | 37.6372512 |
| IBD | rs12705390 | 35.29240055 |
| IBD | rs9392026 | 108.0394805 |
| IBD | rs3778607 | 84.45035746 |
| IBD | rs72928038 | 53.06870018 |
| IBD | rs3087243 | 52.44553257 |
| IBD | rs204295 | 42.22106729 |
| IBD | rs2476601 | 33.29128367 |
| IBD | rs9851967 | 95.52135384 |
| IBD | rs17810546 | 75.06089693 |
| IBD | rs13151961 | 74.42239571 |
| IBD | rs653178 | 56.36192249 |
| IBD | rs2816316 | 50.11751 |
| IBD | rs13098911 | 44.51210622 |
| IBD | rs11221335 | 43.53962169 |
| IBD | rs1018326 | 34.73256097 |
| IBD | rs1738074 | 30.61653045 |
| IBD | rs13003464 | 29.74740267 |
| IBD | rs416352 | 1479.738185 |
| IBD | rs1265099 | 1479.736498 |
| IBD | rs1264622 | 1479.735003 |
| IBD | rs3117326 | 1479.733708 |
| IBD | rs521977 | 1479.72934 |
| IBD | rs9366717 | 1290.54482 |
| IBD | rs2523534 | 1240.027985 |
| IBD | rs1264344 | 1172.828436 |
| IBD | rs13219354 | 1156.538829 |
| IBD | rs2523693 | 1137.865427 |
| IBD | rs13198474 | 920.7933941 |
| IBD | rs3130357 | 843.5232897 |
| IBD | rs23544 | 841.2168935 |
| IBD | rs16869648 | 647.6503087 |
| IBD | rs1383265 | 557.4883494 |
| IBD | rs9296068 | 324.1139529 |
| IBD | rs2030519 | 217.6129001 |
| IBD | rs13132308 | 168.1506364 |
| IBD | rs2235982 | 165.2298147 |
| IBD | rs4947332 | 154.9639815 |
| IBD | rs2017525 | 143.4783783 |
| IBD | rs17264332 | 129.6121552 |
| IBD | rs76830965 | 117.2233821 |

| | | |
|---|---|---|
| IBD | rs1323292 | 107.0991357 |
| IBD | rs4148881 | 102.2553076 |
| IBD | rs3184504 | 88.37382666 |
| IBD | rs2097282 | 86.92046105 |
| IBD | rs2296748 | 83.2303368 |
| IBD | rs6934794 | 83.09811547 |
| IBD | rs499384 | 81.213784 |
| IBD | rs55743914 | 77.79128697 |
| IBD | rs1250552 | 69.41738357 |
| IBD | rs990171 | 68.56934627 |
| IBD | rs12530421 | 66.9080154 |
| IBD | rs1018326 | 66.76226128 |
| IBD | rs13003464 | 66.0759525 |
| IBD | rs182429 | 64.75391564 |
| IBD | rs1980422 | 63.72965887 |
| IBD | rs6910898 | 58.48921424 |
| IBD | rs11066320 | 55.09582463 |
| IBD | rs17404424 | 53.86812827 |
| IBD | rs61907765 | 52.94579441 |
| IBD | rs4947256 | 52.88211844 |
| IBD | rs1929848 | 52.16257946 |
| IBD | rs16894128 | 49.55212246 |
| IBD | rs4445406 | 47.52775009 |
| IBD | rs10892258 | 45.25034448 |
| IBD | rs7104791 | 45.08247447 |
| IBD | rs4821124 | 42.9125254 |
| IBD | rs2267632 | 41.90765533 |
| IBD | rs12068671 | 41.16791264 |
| IBD | rs11875687 | 40.54953979 |
| IBD | rs1570760 | 40.35551091 |
| IBD | rs1050976 | 36.13798535 |
| IBD | rs6933319 | 35.44836417 |
| IBD | rs1893592 | 35.20800313 |
| IBD | rs7616215 | 33.45953222 |
| IBD | rs1378938 | 33.32651768 |
| IBD | rs1107943 | 33.28780669 |
| IBD | rs6715106 | 33.18500134 |
| IBD | rs61579022 | 32.85683346 |
| IBD | rs16894722 | 31.86578322 |
| IBD | rs11801183 | 31.82516941 |
| IBD | rs79758729 | 31.38524655 |
| IBD | rs10800746 | 31.00741953 |
| IBD | rs744254 | 30.68406801 |
| IBD | rs10513548 | 30.03920125 |
| IBD | rs11851414 | 29.834606 |
| IBD | rs3130380 | 2488.026097 |
| IBD | rs3130834 | 1895.744874 |
| IBD | rs9469220 | 1626.502217 |
| IBD | rs9268368 | 1390.544471 |
| IBD | rs9276612 | 1193.007016 |
| IBD | rs13219354 | 1156.678473 |

| | | |
|---|---|---|
| IBD | rs4540292 | 1067.331181 |
| IBD | rs13198474 | 920.515888 |
| IBD | rs16869648 | 647.6977466 |
| IBD | rs2395296 | 549.4338273 |
| IBD | rs2395349 | 405.216881 |
| IBD | rs3734840 | 291.3839027 |
| IBD | rs2229092 | 230.1286855 |
| IBD | rs11753326 | 209.9604293 |
| IBD | rs3097657 | 171.3483583 |
| IBD | rs13205699 | 137.8277567 |
| IBD | rs9258557 | 131.7905808 |
| IBD | rs9380294 | 129.0496726 |
| IBD | rs9501234 | 118.5916253 |
| IBD | rs17351888 | 100 |
| IBD | rs2296748 | 83.22947696 |
| IBD | rs6934794 | 83.10086982 |
| IBD | rs12530421 | 66.91249607 |
| IBD | rs12527282 | 54.34636206 |
| IBD | rs16894128 | 49.54785641 |
| IBD | rs9658119 | 49.18193628 |
| IBD | rs10892258 | 45.25278712 |
| IBD | rs7104791 | 45.07798795 |
| IBD | rs12665489 | 44.715982 |
| IBD | rs2267632 | 41.91254141 |
| IBD | rs7761745 | 38.36563639 |
| IBD | rs1611697 | 36.36086417 |
| IBD | rs10752747 | 34.17580435 |
| IBD | rs7162232 | 33.28148309 |
| IBD | rs16894722 | 31.86599681 |
| IBD | rs2441467 | 31.80993363 |
| IBD | rs11757750 | 31.25940326 |
| IBD | rs13030124 | 31.13618677 |
| IBD | rs11979905 | 31.0695865 |
| IBD | rs9851967 | 95.32510474 |
| IBD | rs17810546 | 74.65691017 |
| IBD | rs13151961 | 73.67023001 |
| IBD | rs653178 | 56.0625039 |
| IBD | rs2816316 | 49.85991365 |
| IBD | rs13098911 | 44.23540633 |
| IBD | rs1018326 | 34.4837366 |
| IBD | rs1738074 | 30.77181067 |
| IBD | rs13003464 | 29.37484965 |
| IBD | rs13091753 | 66.34364786 |
| IBD | rs6933404 | 51.49762868 |
| IBD | rs13132933 | 46.43165397 |
| IBD | rs1468791 | 45.04994231 |
| IBD | rs11221335 | 43.39365361 |
| IBD | rs9819226 | 38.95214004 |
| IBD | rs1323292 | 34.38686922 |
| IBD | rs9851967 | 95.32510474 |
| IBD | rs17810546 | 74.65691017 |

| | | |
|---|---|---|
| IBD | rs13151961 | 73.67023001 |
| IBD | rs653178 | 56.0625039 |
| IBD | rs2816316 | 49.85991365 |
| IBD | rs13098911 | 44.23540633 |
| IBD | rs11221335 | 43.39365361 |
| IBD | rs1018326 | 34.4837366 |
| IBD | rs1738074 | 30.77181067 |
| IBD | rs13003464 | 29.37484965 |
| IBD | rs6983267 | 91.83428037 |
| IBD | rs4939827 | 70.97770955 |
| IBD | rs16892766 | 35.04778755 |
| IBD | rs3957146 | 670.2345679 |
| IBD | rs2523504 | 82.30249168 |
| IBD | rs2237897 | 76.41833716 |
| IBD | rs2476601 | 66.35707816 |
| IBD | rs11558471 | 45.99794138 |
| IBD | rs10938397 | 43.04966681 |
| IBD | rs17187735 | 38.90640625 |
| IBD | rs76177300 | 36.51612245 |
| IBD | rs10974438 | 36.23801588 |
| IBD | rs34337125 | 32.2624 |
| IBD | rs9765960 | 30.15685148 |
| IBD | rs5215 | 30.13785923 |
| IBD | rs3957146 | 537.1139442 |
| IBD | rs2476601 | 77.30128082 |
| IBD | rs34171662 | 69.12311447 |
| IBD | rs17187735 | 44.72780591 |
| IBD | rs2296329 | 34.84133448 |
| IBD | rs878898 | 33.08058423 |
| IBD | rs3957146 | 659.0029432 |
| IBD | rs2523504 | 80.02994887 |
| IBD | rs2237897 | 77.04938272 |
| IBD | rs2476601 | 64.58958694 |
| IBD | rs11558471 | 46.1041 |
| IBD | rs10938397 | 45.21876302 |
| IBD | rs11708067 | 41.95053254 |
| IBD | rs17187735 | 37.98478668 |
| IBD | rs10974438 | 37.07724733 |
| IBD | rs76177300 | 35.31430141 |
| IBD | rs56258397 | 32.76333321 |
| IBD | rs34337125 | 29.97285991 |
| IBD | rs5215 | 29.58027905 |
| IBD | rs9273363 | 219.6369847 |
| IBD | rs1421085 | 95.67277557 |
| IBD | rs849142 | 62.07355484 |
| IBD | rs1260326 | 51.3808817 |
| IBD | rs697239 | 47.68540027 |
| IBD | rs5215 | 41.04837291 |
| IBD | rs1800961 | 36.67443398 |
| IBD | rs3131064 | 33.03226561 |
| IBD | rs2476601 | 30.65820455 |

| | | |
|---|---|---|
| IBD | rs646123 | 29.81313658 |
| IBD | rs3957146 | 609.261834 |
| IBD | rs2237897 | 81.56643184 |
| IBD | rs2523504 | 78.25443787 |
| IBD | rs2476601 | 65.34027778 |
| IBD | rs4402960 | 54.57349241 |
| IBD | rs10938397 | 47.24893958 |
| IBD | rs11558471 | 42.25 |
| IBD | rs76177300 | 36.1630802 |
| IBD | rs17187735 | 34.8239935 |
| IBD | rs853974 | 34.56629013 |
| IBD | rs5215 | 31.49062117 |
| IBD | rs9765960 | 30.74881374 |
| IBD | rs3957146 | 446.3578212 |
| IBD | rs2476601 | 67.27738411 |
| IBD | rs34018127 | 64.05307899 |
| IBD | rs3873444 | 58.75570162 |
| IBD | rs2107202 | 35.36190334 |
| IBD | rs17187735 | 33.03913015 |
| IBD | rs4795399 | 213.1525795 |
| IBD | rs2197415 | 166.0926748 |
| IBD | rs72743461 | 164.0231007 |
| IBD | rs1321859 | 104.035551 |
| IBD | rs2621322 | 76.05759964 |
| IBD | rs1117490 | 66.23317075 |
| IBD | rs17454584 | 59.77524667 |
| IBD | rs917115 | 49.25872218 |
| IBD | rs35570272 | 48.80394015 |
| IBD | rs72799426 | 43.89843058 |
| IBD | rs174535 | 39.46309793 |
| IBD | rs2338819 | 37.18354855 |
| IBD | rs429916 | 36.72699383 |
| IBD | rs7771953 | 36.00951948 |
| IBD | rs13208164 | 35.02525737 |
| IBD | rs41284471 | 34.87325903 |
| IBD | rs805287 | 34.14574701 |
| IBD | rs2296618 | 33.32483733 |
| IBD | rs3781094 | 32.70625219 |
| IBD | rs6729966 | 31.35010616 |
| IBD | rs12365699 | 29.98577268 |
| IBD | rs9375459 | 159.8205842 |
| IBD | rs11072508 | 124.9436252 |
| IBD | rs1052486 | 89.3645148 |
| IBD | rs7838131 | 58.06523158 |
| IBD | rs11864054 | 45.13135868 |
| IBD | rs169080 | 44.7912553 |
| IBD | rs2246490 | 39.93143088 |
| IBD | rs11229555 | 37.49582107 |
| IBD | rs27433 | 37.12780233 |
| IBD | rs35633336 | 37.00116955 |
| IBD | rs57786342 | 32.27754701 |

| | | |
|---|---|---|
| IBD | rs10072700 | 32.14303234 |
| IBD | rs6905288 | 31.8339008 |
| IBD | rs74580656 | 31.26109983 |
| IBD | rs4146810 | 31.07499639 |
| IBD | rs696 | 30.38757938 |
| IBD | rs13003198 | 29.72938169 |
| IBD | rs11690644 | 247.8241064 |
| IBD | rs11742240 | 198.5205289 |
| IBD | rs61839660 | 137.9976273 |
| IBD | rs72743461 | 90.09936414 |
| IBD | rs4845604 | 81.45904723 |
| IBD | rs45613035 | 77.33398792 |
| IBD | rs2844787 | 69.45999167 |
| IBD | rs11171739 | 67.83227124 |
| IBD | rs653178 | 63.85847713 |
| IBD | rs2228145 | 57.33363979 |
| IBD | rs905670 | 52.97274528 |
| IBD | rs10912564 | 52.80210761 |
| IBD | rs17057868 | 49.83749708 |
| IBD | rs10903115 | 47.60590435 |
| IBD | rs16858573 | 46.35375184 |
| IBD | rs5029949 | 36.47550325 |
| IBD | rs12365699 | 35.85211005 |
| IBD | rs4939489 | 34.84480371 |
| IBD | rs2255088 | 33.70206102 |
| IBD | rs2893907 | 32.23625983 |
| IBD | rs647397 | 31.11630209 |
| IBD | rs2422254 | 30.01641747 |
| IBD | rs34463936 | 177.358569 |
| IBD | rs72743461 | 152.2769204 |
| IBD | rs962993 | 121.0171896 |
| IBD | rs61839660 | 106.7358089 |
| IBD | rs4845604 | 79.72036465 |
| IBD | rs905670 | 77.67955622 |
| IBD | rs849335 | 68.72827897 |
| IBD | rs45613035 | 66.44838124 |
| IBD | rs10903115 | 58.32674249 |
| IBD | rs2228145 | 56.70650655 |
| IBD | rs6025 | 51.33053693 |
| IBD | rs17057868 | 51.16765851 |
| IBD | rs10912564 | 45.20357895 |
| IBD | rs249677 | 44.6207053 |
| IBD | rs12365699 | 43.94522771 |
| IBD | rs6011033 | 43.92054271 |
| IBD | rs5029949 | 41.58508412 |
| IBD | rs4410871 | 39.48207075 |
| IBD | rs2255088 | 38.23508331 |
| IBD | rs16858573 | 38.20229185 |
| IBD | rs2893907 | 35.54250255 |
| IBD | rs11256311 | 33.1705323 |
| IBD | rs1177289 | 31.81903734 |

| | | |
|---|---|---|
| IBD | rs28360011 | 31.51135145 |
| IBD | rs72799426 | 31.00951592 |
| IBD | rs114420077 | 30.05833404 |
| IBD | rs17538393 | 29.7510625 |
| IBD | rs4939827 | 89.14101097 |
| IBD | rs6983267 | 62.09929775 |
| IBD | rs10936599 | 32.11321132 |
| IBD | rs11591147 | 118.0998178 |
| IBD | rs646776 | 82.44194362 |
| IBD | rs499883 | 68.2473268 |
| IBD | rs1367117 | 65.30387144 |
| IBD | rs4953023 | 48.02547037 |
| IBD | rs12916 | 36.36180677 |
| IBD | rs2476601 | 401.7161224 |
| IBD | rs11571297 | 97.87548308 |
| IBD | rs115084931 | 50.83753946 |
| IBD | rs1990760 | 40.90932262 |
| IBD | rs10905668 | 38.66578512 |
| IBD | rs28780083 | 37.02999096 |
| IBD | rs11964080 | 33.75907955 |
| IBD | rs11102634 | 32.18297214 |
| IBD | rs4409785 | 30.37418735 |
| IBD | rs1800961 | 47.48195767 |
| IBD | rs1260326 | 37.88932141 |
| IBD | rs1169286 | 34.98169542 |
| IBD | rs11591147 | 81.82562262 |
| IBD | rs646776 | 54.243225 |
| IBD | rs1367117 | 46.10485028 |
| IBD | rs499883 | 45.39801457 |
| IBD | rs688 | 32.57346817 |
| IBD | rs12916 | 31.60633551 |
| IBD | rs646776 | 188.9443931 |
| IBD | rs1367117 | 69.49341629 |
| IBD | rs1260326 | 68.65754422 |
| IBD | rs11591147 | 60.64810791 |
| IBD | rs4245791 | 58.01732088 |
| IBD | rs12916 | 36.35360002 |
| IBD | rs2244608 | 31.25966387 |
| IBD | rs2240326 | 96.02888374 |
| IBD | rs773109 | 55.63788661 |
| IBD | rs2232423 | 45.70598484 |
| IBD | rs1716171 | 42.30125524 |
| IBD | rs6722661 | 41.55473241 |
| IBD | rs205262 | 41.19322308 |
| IBD | rs329122 | 35.14855924 |
| IBD | rs3172494 | 33.61569586 |
| IBD | rs9615905 | 32.46700519 |
| IBD | rs2164300 | 30.0858308 |
| IBD | rs17293632 | 139.3426276 |
| IBD | rs9847710 | 99.500625 |
| IBD | rs72707023 | 53.31755073 |

| | | |
|---|---|---|
| IBD | rs3072 | 42.40868531 |
| IBD | rs1262776 | 41.24493827 |
| IBD | rs10460504 | 38.9138322 |
| IBD | rs10838738 | 36.58774539 |
| IBD | rs2476601 | 269.7717944 |
| IBD | rs3087243 | 80.62801427 |
| IBD | rs9277557 | 47.87360086 |
| IBD | rs2111485 | 33.52242176 |
| IBD | rs10974437 | 31.90387634 |
| IBD | rs7599564 | 31.55092802 |
| IBD | rs28780083 | 29.84229219 |
| IBD | rs2476601 | 273.398267 |
| IBD | rs3087243 | 82.113389 |
| IBD | rs9277557 | 47.24587132 |
| IBD | rs117846960 | 34.64073817 |
| IBD | rs10974437 | 31.82431474 |
| IBD | rs7599564 | 31.81913311 |
| IBD | rs28780083 | 30.25 |
| IBD | rs2476601 | 259.5679012 |
| IBD | rs3184504 | 103.8145002 |
| IBD | rs11066320 | 85.7971076 |
| IBD | rs11571297 | 65.74141709 |
| IBD | rs9277557 | 56.33244777 |
| IBD | rs10174238 | 34.62689481 |
| IBD | rs2523719 | 32.02530992 |
| IBD | rs2476601 | 438.5166638 |
| IBD | rs11571297 | 109.5888321 |
| IBD | rs9277542 | 72.78222656 |
| IBD | rs229541 | 61.12396694 |
| IBD | rs1990760 | 49.64430603 |
| IBD | rs7757496 | 47.48803739 |
| IBD | rs28780083 | 45.47419205 |
| IBD | rs10905669 | 42.41402443 |
| IBD | rs877636 | 39.78698225 |
| IBD | rs10748781 | 38.37418749 |
| IBD | rs4409785 | 38.28515625 |
| IBD | rs210179 | 32.49974432 |
| IBD | rs11102634 | 29.96121884 |
| IBD | rs2476601 | 475.24 |
| IBD | rs11571297 | 117.9034028 |
| IBD | rs9277542 | 80.21359689 |
| IBD | rs229541 | 61.44965527 |
| IBD | rs1990760 | 59.69873621 |
| IBD | rs28780083 | 43.34027778 |
| IBD | rs10748781 | 41.61307444 |
| IBD | rs4409785 | 40.74418114 |
| IBD | rs41295131 | 39.76626436 |
| IBD | rs10517086 | 39.55230713 |
| IBD | rs210192 | 30.25 |
| IBD | rs2476601 | 437.9855611 |
| IBD | rs11571297 | 110.4375797 |

| | | |
|---|---|---|
| IBD | rs9277542 | 78.765625 |
| IBD | rs229541 | 62.98586777 |
| IBD | rs1990760 | 51.73436579 |
| IBD | rs28780083 | 47.55251736 |
| IBD | rs2442719 | 44.72265625 |
| IBD | rs10748781 | 41.1173438 |
| IBD | rs877636 | 41.09138725 |
| IBD | rs4409785 | 40.02319637 |
| IBD | rs6448432 | 38.95840278 |
| IBD | rs210179 | 32.49974432 |
| IBD | rs11102634 | 30.82967382 |
| IBD | rs12567136 | 174.6834486 |
| IBD | rs11072508 | 135.5905439 |
| IBD | rs7838131 | 67.47701147 |
| IBD | rs3764769 | 46.70771246 |
| IBD | rs169080 | 42.21912725 |
| IBD | rs35633336 | 36.17134696 |
| IBD | rs736801 | 34.30657189 |
| IBD | rs10409243 | 33.90872489 |
| IBD | rs4713488 | 33.54699275 |
| IBD | rs27433 | 32.72883438 |
| IBD | rs696 | 31.46061655 |
| IBD | rs11229555 | 31.07838325 |
| IBD | rs11591147 | 57.5872865 |
| IBD | rs12705390 | 53.29 |
| IBD | rs653178 | 34.93654337 |
| IBD | rs2495477 | 33.45215486 |
| IBD | rs1044506 | 33.04486432 |
| IBD | rs3957146 | 779.2267995 |
| IBD | rs1121980 | 104.1940192 |
| IBD | rs2523504 | 96.85840278 |
| IBD | rs2237897 | 77.00957934 |
| IBD | rs2476601 | 76.44430241 |
| IBD | rs10938397 | 50.73202207 |
| IBD | rs9765960 | 43.71837714 |
| IBD | rs28624681 | 41.5308642 |
| IBD | rs11558471 | 41.00698594 |
| IBD | rs76177300 | 38.86915577 |
| IBD | rs4148881 | 36.39290296 |
| IBD | rs7100984 | 32.33560758 |
| IBD | rs34337125 | 31.75901825 |
| IBD | rs4273712 | 31.66446423 |
| IBD | rs3957146 | 778.6116998 |
| IBD | rs1121980 | 104.3452703 |
| IBD | rs2523504 | 97.8627826 |
| IBD | rs2237897 | 80.90911642 |
| IBD | rs2476601 | 76.23985406 |
| IBD | rs10938397 | 51.51751245 |
| IBD | rs11558471 | 42.72404959 |
| IBD | rs9765960 | 41.58026159 |
| IBD | rs76177300 | 39.43181862 |

| | | |
|---|---|---|
| IBD | rs4148881 | 34.65790843 |
| IBD | rs34337125 | 33.16906722 |
| IBD | rs853974 | 31.17361111 |
| IBD | rs7100984 | 30.8025 |
| IBD | rs9271100 | 1153.78976 |
| IBD | rs2857700 | 930.1440187 |
| IBD | rs9469220 | 808.7316572 |
| IBD | rs9266629 | 238.0532385 |
| IBD | rs722788 | 129.5756482 |
| IBD | rs12927355 | 114.0780906 |
| IBD | rs9277641 | 104.8002141 |
| IBD | rs2104286 | 95.65667554 |
| IBD | rs13194491 | 79.16415234 |
| IBD | rs1813375 | 74.64878281 |
| IBD | rs2394423 | 72.05492404 |
| IBD | rs41286801 | 64.78177037 |
| IBD | rs1800693 | 64.71669962 |
| IBD | rs8070345 | 64.71669962 |
| IBD | rs1920296 | 59.88255844 |
| IBD | rs11554159 | 53.94065835 |
| IBD | rs1077667 | 53.28033723 |
| IBD | rs2255214 | 52.36224806 |
| IBD | rs4976646 | 51.53151101 |
| IBD | rs3748817 | 50.68870328 |
| IBD | rs7762619 | 49.77234597 |
| IBD | rs3129269 | 49.43856505 |
| IBD | rs17066096 | 47.45176818 |
| IBD | rs12087340 | 47.25406297 |
| IBD | rs11172342 | 46.2049889 |
| IBD | rs2071292 | 46.07892099 |
| IBD | rs74796499 | 41.9955859 |
| IBD | rs6498184 | 39.62359077 |
| IBD | rs34383631 | 39.44266262 |
| IBD | rs1870071 | 38.54918016 |
| IBD | rs1021156 | 38.15633434 |
| IBD | rs2288904 | 37.9201961 |
| IBD | rs1014486 | 36.84544637 |
| IBD | rs9967792 | 36.2725284 |
| IBD | rs11154801 | 36.20743486 |
| IBD | rs4410871 | 36.14245546 |
| IBD | rs55838263 | 36.0921596 |
| IBD | rs842639 | 35.61351815 |
| IBD | rs71624119 | 35.10542979 |
| IBD | rs7923837 | 34.81640662 |
| IBD | rs941816 | 34.81320201 |
| IBD | rs6881706 | 34.0714384 |
| IBD | rs6880778 | 32.36681908 |
| IBD | rs9380294 | 32.22777182 |
| IBD | rs917116 | 31.70531526 |
| IBD | rs4796791 | 31.5258197 |
| IBD | rs6933319 | 30.42722174 |

| | | |
|---|---|---|
| IBD | rs3748080 | 29.73972414 |
| IBD | rs67297943 | 29.52202688 |
| IBD | rs9271100 | 1151.631086 |
| IBD | rs2857700 | 921.3435307 |
| IBD | rs9469220 | 814.5751003 |
| IBD | rs9266629 | 240.2826901 |
| IBD | rs722788 | 128.8075972 |
| IBD | rs12927355 | 114.9205234 |
| IBD | rs9277641 | 105.0513448 |
| IBD | rs2104286 | 96.81623181 |
| IBD | rs3135333 | 88.71538787 |
| IBD | rs13194491 | 78.0008487 |
| IBD | rs1813375 | 74.60467438 |
| IBD | rs2394423 | 73.02581602 |
| IBD | rs8070345 | 65.6342256 |
| IBD | rs1800693 | 65.15622698 |
| IBD | rs41286801 | 64.88992935 |
| IBD | rs212407 | 62.57878754 |
| IBD | rs57271503 | 61.6464492 |
| IBD | rs1920296 | 60.66954042 |
| IBD | rs11554159 | 53.5042742 |
| IBD | rs1077667 | 52.88306485 |
| IBD | rs1323292 | 52.80705178 |
| IBD | rs2255214 | 52.07945023 |
| IBD | rs4976646 | 50.76723335 |
| IBD | rs3748817 | 50.28489445 |
| IBD | rs7762619 | 49.75718908 |
| IBD | rs12087340 | 47.63652841 |
| IBD | rs17066096 | 47.3591021 |
| IBD | rs11172342 | 46.43729685 |
| IBD | rs2071292 | 46.00408962 |
| IBD | rs74796499 | 42.14598704 |
| IBD | rs6498184 | 40.39956714 |
| IBD | rs1021156 | 38.45620754 |
| IBD | rs1870071 | 38.42876024 |
| IBD | rs34383631 | 38.42542586 |
| IBD | rs2745400 | 37.80488558 |
| IBD | rs2288904 | 37.41094708 |
| IBD | rs1014486 | 37.03575872 |
| IBD | rs2364482 | 36.66893084 |
| IBD | rs55838263 | 36.65497369 |
| IBD | rs842639 | 36.29036119 |
| IBD | rs9967792 | 36.17935672 |
| IBD | rs4410871 | 35.99321002 |
| IBD | rs11154801 | 35.65969173 |
| IBD | rs71624119 | 35.38911173 |
| IBD | rs941816 | 34.40743197 |
| IBD | rs7923837 | 34.36020519 |
| IBD | rs6881706 | 34.24060615 |
| IBD | rs9380294 | 32.18739863 |
| IBD | rs6880778 | 31.8103382 |

| | | |
|---|---|---|
| IBD | rs4796791 | 31.68833603 |
| IBD | rs917116 | 31.42801881 |
| IBD | rs1059288 | 31.3452092 |
| IBD | rs6933319 | 30.45318115 |
| IBD | rs67297943 | 29.78362904 |
| IBD | rs3134954 | 1116.320754 |
| IBD | rs2844670 | 388.3801045 |
| IBD | rs16896742 | 229.2913396 |
| IBD | rs404860 | 163.5528652 |
| IBD | rs9276711 | 122.2844658 |
| IBD | rs9348876 | 79.48075962 |
| IBD | rs17500468 | 62.48042532 |
| IBD | rs4648356 | 57.68892884 |
| IBD | rs7200786 | 56.26968676 |
| IBD | rs4613763 | 56.06171561 |
| IBD | rs13194491 | 53.66838073 |
| IBD | rs11154801 | 50.04225264 |
| IBD | rs1077667 | 49.37731631 |
| IBD | rs669607 | 44.17564017 |
| IBD | rs1738074 | 43.06640625 |
| IBD | rs396090 | 42.91176937 |
| IBD | rs1800693 | 40.65524787 |
| IBD | rs17066096 | 39.43963441 |
| IBD | rs2119704 | 39.39924413 |
| IBD | rs11581062 | 39.23439198 |
| IBD | rs2293370 | 37.18024985 |
| IBD | rs9282641 | 36.49615105 |
| IBD | rs650258 | 36.2064142 |
| IBD | rs1335532 | 35.95597677 |
| IBD | rs3118470 | 35.93785068 |
| IBD | rs2248359 | 34.11927075 |
| IBD | rs11129295 | 32.58482078 |
| IBD | rs4902647 | 30.27350884 |
| IBD | rs874628 | 29.99117201 |
| IBD | rs2857700 | 1043.603238 |
| IBD | rs4148874 | 686.7725682 |
| IBD | rs2844787 | 124.5897171 |
| IBD | rs11256593 | 115.2953852 |
| IBD | rs28732150 | 94.64364232 |
| IBD | rs2071463 | 86.33740546 |
| IBD | rs11749040 | 71.01785716 |
| IBD | rs438613 | 69.08375957 |
| IBD | rs1026916 | 55.32806096 |
| IBD | rs6032662 | 53.31217807 |
| IBD | rs1077667 | 51.19208668 |
| IBD | rs2546890 | 50.76912939 |
| IBD | rs35540610 | 48.76956315 |
| IBD | rs631204 | 47.18824872 |
| IBD | rs17124032 | 47.00466038 |
| IBD | rs1860545 | 46.81876026 |
| IBD | rs1738074 | 46.34664316 |

| | | |
|---|---|---|
| IBD | rs701006 | 45.74151198 |
| IBD | rs13327021 | 45.25801287 |
| IBD | rs1250551 | 44.41118521 |
| IBD | rs59655222 | 43.73604334 |
| IBD | rs72928038 | 42.02554183 |
| IBD | rs1014486 | 41.21439181 |
| IBD | rs7592560 | 39.75841812 |
| IBD | rs12365699 | 39.58166659 |
| IBD | rs9843355 | 38.78762253 |
| IBD | rs10063294 | 37.0867685 |
| IBD | rs35703946 | 36.03508161 |
| IBD | rs3130171 | 34.77546197 |
| IBD | rs354033 | 32.47876532 |
| IBD | rs7855251 | 30.03961833 |
| IBD | rs2232423 | 78.44897959 |
| IBD | rs2568958 | 75.37396694 |
| IBD | rs13037326 | 40.02498959 |
| IBD | rs4141983 | 32.93761815 |
| IBD | rs17641524 | 32.03987184 |
| IBD | rs9831648 | 31.53254438 |
| IBD | rs2671427 | 38.71455701 |
| IBD | rs2666957 | 35.19131503 |
| IBD | rs7153386 | 29.98211979 |
| IBD | rs7553711 | 48.40858921 |
| IBD | rs34593439 | 36.4330083 |
| IBD | rs10995245 | 29.79195949 |
| IBD | rs962993 | 42.02303291 |
| IBD | rs17027258 | 39.09494618 |
| IBD | rs34540843 | 30.8216409 |
| IBD | rs3774354 | 46.03364845 |
| IBD | rs56116847 | 32.06390625 |
| IBD | rs10758594 | 32.0620678 |
| IBD | rs7774434 | 250.1841762 |
| IBD | rs6679356 | 119.6655099 |
| IBD | rs10488631 | 97.61563466 |
| IBD | rs10947260 | 67.70253747 |
| IBD | rs2293370 | 61.57651862 |
| IBD | rs12924729 | 58.18593463 |
| IBD | rs4938573 | 55.0940682 |
| IBD | rs243325 | 48.73339793 |
| IBD | rs17641524 | 46.30242557 |
| IBD | rs2069235 | 44.75387889 |
| IBD | rs9303277 | 44.48022503 |
| IBD | rs860413 | 43.28748258 |
| IBD | rs2304256 | 41.72722106 |
| IBD | rs7665090 | 37.70934918 |
| IBD | rs1800693 | 36.1412363 |
| IBD | rs911263 | 35.74633426 |
| IBD | rs11065987 | 30.58355034 |
| IBD | rs7775055 | 220.8673499 |
| IBD | rs72678531 | 167.6055727 |

| | | |
|---|---|---|
| IBD | rs522127 | 94.11121064 |
| IBD | rs35188261 | 92.56254669 |
| IBD | rs9295947 | 65.38650962 |
| IBD | rs2293370 | 65.18043975 |
| IBD | rs17122453 | 64.94228033 |
| IBD | rs1646019 | 60.6790643 |
| IBD | rs1800693 | 59.57663455 |
| IBD | rs10931468 | 56.99870129 |
| IBD | rs12708715 | 56.80966557 |
| IBD | rs8067378 | 56.35437544 |
| IBD | rs7665090 | 55.69208254 |
| IBD | rs2267407 | 54.86217665 |
| IBD | rs6871748 | 53.76214643 |
| IBD | rs12134279 | 48.13705887 |
| IBD | rs911263 | 41.83114872 |
| IBD | rs1014486 | 41.68009932 |
| IBD | rs3998159 | 38.752453 |
| IBD | rs17564829 | 35.83475138 |
| IBD | rs11065979 | 35.26890051 |
| IBD | rs34725611 | 34.50840924 |
| IBD | rs11064157 | 34.07174106 |
| IBD | rs11117431 | 32.86547623 |
| IBD | rs2523647 | 30.51916156 |
| IBD | rs10847864 | 50.65622172 |
| IBD | rs620490 | 38.17939058 |
| IBD | rs4488803 | 32.58745991 |
| IBD | rs6983267 | 643.4891844 |
| IBD | rs10840603 | 302.6889838 |
| IBD | rs651164 | 157.9591942 |
| IBD | rs1058319 | 103.4157778 |
| IBD | rs17749618 | 51.33301038 |
| IBD | rs2305498 | 46.5124 |
| IBD | rs2523733 | 114.6894657 |
| IBD | rs2071463 | 68.61710623 |
| IBD | rs9263875 | 55.15399286 |
| IBD | rs74817271 | 43.29014502 |
| IBD | rs12713428 | 33.00060592 |
| IBD | rs13201757 | 1019.640594 |
| IBD | rs9391734 | 397.5892951 |
| IBD | rs28780084 | 205.7241026 |
| IBD | rs2199874 | 103.7710131 |
| IBD | rs7741091 | 87.83161454 |
| IBD | rs926070 | 66.26200654 |
| IBD | rs4845454 | 63.07969313 |
| IBD | rs6457109 | 62.21317999 |
| IBD | rs17728338 | 60.48454624 |
| IBD | rs6833586 | 55.95279491 |
| IBD | rs2546890 | 50.60564096 |
| IBD | rs141417497 | 47.92730231 |
| IBD | rs72669160 | 46.61059326 |
| IBD | rs1416173 | 45.30658757 |

| | | |
|---|---|---|
| IBD | rs114835245 | 44.79300763 |
| IBD | rs78580783 | 42.2007555 |
| IBD | rs9461362 | 42.00074802 |
| IBD | rs68191 | 39.83862541 |
| IBD | rs77840275 | 39.65505709 |
| IBD | rs892085 | 39.02536227 |
| IBD | rs30376 | 38.63362758 |
| IBD | rs9399137 | 37.54827919 |
| IBD | rs3868078 | 34.72041834 |
| IBD | rs80174646 | 34.37441395 |
| IBD | rs9262155 | 33.9142097 |
| IBD | rs2074478 | 32.98833642 |
| IBD | rs62421044 | 31.808472 |
| IBD | rs1990760 | 31.54190303 |
| IBD | rs11652075 | 31.27935367 |
| IBD | rs12447534 | 31.0861904 |
| IBD | rs117950837 | 30.31483062 |
| IBD | rs11564177 | 30.03806355 |
| IBD | rs2476601 | 317.0948351 |
| IBD | rs8283 | 226.3014613 |
| IBD | rs2074488 | 178.4507845 |
| IBD | rs4711211 | 144.0976519 |
| IBD | rs106287 | 115.8059851 |
| IBD | rs6922431 | 76.20597332 |
| IBD | rs456261 | 61.10810475 |
| IBD | rs2532919 | 53.34238971 |
| IBD | rs6901724 | 51.3848525 |
| IBD | rs6920220 | 49.05173894 |
| IBD | rs10040327 | 42.61910456 |
| IBD | rs3909134 | 40.93414998 |
| IBD | rs4810485 | 33.93781964 |
| IBD | rs3087243 | 31.27446019 |
| IBD | rs11757182 | 30.80494096 |
| IBD | rs2267632 | 30.76140683 |
| IBD | rs2476601 | 869.340315 |
| IBD | rs3130379 | 272.8979258 |
| IBD | rs1571878 | 165.34496 |
| IBD | rs3893538 | 140.3479735 |
| IBD | rs6456964 | 133.7007259 |
| IBD | rs8026898 | 129.8023912 |
| IBD | rs7752903 | 126.8912923 |
| IBD | rs34695944 | 100.9924468 |
| IBD | rs4239702 | 100.9924468 |
| IBD | rs29243 | 76.07991245 |
| IBD | rs9348832 | 68.80725792 |
| IBD | rs1858036 | 66.93076603 |
| IBD | rs3087243 | 64.82443647 |
| IBD | rs17404424 | 61.55848945 |
| IBD | rs11933540 | 60.50338484 |
| IBD | rs2561477 | 57.74592653 |
| IBD | rs2301888 | 55.86258863 |

| | | |
|---|---|---|
| IBD | rs1893592 | 55.73265308 |
| IBD | rs6712515 | 55.73265308 |
| IBD | rs11889341 | 55.45614566 |
| IBD | rs3806624 | 55.28948982 |
| IBD | rs8032939 | 44.37181597 |
| IBD | rs3128935 | 43.43043586 |
| IBD | rs11574914 | 40.06789519 |
| IBD | rs62097857 | 38.20344257 |
| IBD | rs4409785 | 37.10507058 |
| IBD | rs1953126 | 36.62845648 |
| IBD | rs947474 | 33.38311413 |
| IBD | rs73081554 | 31.68823116 |
| IBD | rs9268145 | 1121.704453 |
| IBD | rs2476601 | 275.0492786 |
| IBD | rs10484565 | 159.9238633 |
| IBD | rs1029237 | 143.5033085 |
| IBD | rs1431403 | 138.0943099 |
| IBD | rs544167 | 132.6617645 |
| IBD | rs2524084 | 117.9188209 |
| IBD | rs13196329 | 106.592422 |
| IBD | rs6922431 | 91.49959803 |
| IBD | rs2267644 | 89.36491131 |
| IBD | rs620202 | 85.36086947 |
| IBD | rs71624119 | 83.75814367 |
| IBD | rs2296330 | 69.52736813 |
| IBD | rs2531804 | 54.63181621 |
| IBD | rs361525 | 53.6871644 |
| IBD | rs462618 | 53.19226861 |
| IBD | rs6920220 | 40.63258014 |
| IBD | rs17630466 | 40.07416584 |
| IBD | rs13426947 | 37.96899322 |
| IBD | rs194679 | 37.77708423 |
| IBD | rs8026898 | 35.76860251 |
| IBD | rs10209110 | 32.6104239 |
| IBD | rs2812378 | 32.32107356 |
| IBD | rs2228145 | 32.30745226 |
| IBD | rs2240339 | 32.24777667 |
| IBD | rs2476601 | 678.2551357 |
| IBD | rs3749946 | 98.08874685 |
| IBD | rs3087243 | 82.77339454 |
| IBD | rs17264332 | 78.73552445 |
| IBD | rs68191 | 73.15273165 |
| IBD | rs8026898 | 71.78450094 |
| IBD | rs34046593 | 69.13361899 |
| IBD | rs17404424 | 66.33736053 |
| IBD | rs1571878 | 61.30077778 |
| IBD | rs4239702 | 57.07239393 |
| IBD | rs34695944 | 56.98157996 |
| IBD | rs11574914 | 54.57048351 |
| IBD | rs12764378 | 54.10525646 |
| IBD | rs13426947 | 49.1263451 |

| | | |
|---|---|---|
| IBD | rs8032939 | 49.12630299 |
| IBD | rs9653442 | 48.33154658 |
| IBD | rs12539741 | 45.97130691 |
| IBD | rs2069235 | 39.67457119 |
| IBD | rs2561477 | 38.60115167 |
| IBD | rs2451258 | 38.13561104 |
| IBD | rs9368696 | 37.32477138 |
| IBD | rs2240336 | 36.66918076 |
| IBD | rs10796038 | 35.97381764 |
| IBD | rs12232497 | 34.82901639 |
| IBD | rs592390 | 34.7234128 |
| IBD | rs61828284 | 33.11201994 |
| IBD | rs1893592 | 32.8805961 |
| IBD | rs10985070 | 31.81033859 |
| IBD | rs9310852 | 30.58230767 |
| IBD | rs624988 | 29.87842993 |
| IBD | rs4936059 | 29.87840789 |
| IBD | rs73081554 | 29.83663305 |
| IBD | rs9268145 | 1581.710785 |
| IBD | rs2476601 | 302.4266715 |
| IBD | rs8283 | 224.880682 |
| IBD | rs2074488 | 168.2536436 |
| IBD | rs4711211 | 143.8448016 |
| IBD | rs106287 | 119.7872959 |
| IBD | rs6922431 | 74.83083318 |
| IBD | rs456261 | 57.33781905 |
| IBD | rs2532919 | 52.74731825 |
| IBD | rs6901724 | 49.46524787 |
| IBD | rs6920220 | 46.04012992 |
| IBD | rs10040327 | 42.6826205 |
| IBD | rs3909134 | 40.09318172 |
| IBD | rs3087243 | 35.19521763 |
| IBD | rs2267632 | 30.81277589 |
| IBD | rs11757182 | 30.51698516 |
| IBD | rs4810485 | 29.25598476 |
| IBD | rs2476601 | 678.1835787 |
| IBD | rs8192585 | 231.0436375 |
| IBD | rs3087243 | 82.81707269 |
| IBD | rs17264332 | 78.80610491 |
| IBD | rs68191 | 73.16315931 |
| IBD | rs8026898 | 71.82587412 |
| IBD | rs11933540 | 69.06149303 |
| IBD | rs7738250 | 68.84871266 |
| IBD | rs17404424 | 66.29695683 |
| IBD | rs1571878 | 61.29400579 |
| IBD | rs4239702 | 57.07597389 |
| IBD | rs34695944 | 56.92861801 |
| IBD | rs11574914 | 54.61975304 |
| IBD | rs12764378 | 54.04717811 |
| IBD | rs13426947 | 49.11796565 |
| IBD | rs8032939 | 49.11329476 |

| | | |
|---|---|---|
| IBD | rs9653442 | 48.37975309 |
| IBD | rs2069235 | 39.62299901 |
| IBD | rs2561477 | 38.57806634 |
| IBD | rs2451258 | 38.19013214 |
| IBD | rs9368696 | 37.29145521 |
| IBD | rs2240336 | 36.59887734 |
| IBD | rs10796038 | 35.95196798 |
| IBD | rs592390 | 34.74349906 |
| IBD | rs61828284 | 33.11008605 |
| IBD | rs9469591 | 32.9190277 |
| IBD | rs1893592 | 32.88498791 |
| IBD | rs10985070 | 31.79931288 |
| IBD | rs9310852 | 30.57771989 |
| IBD | rs624988 | 29.90069577 |
| IBD | rs4936059 | 29.85748686 |
| IBD | rs73081554 | 29.83558431 |
| IBD | rs3957146 | 389.9863469 |
| IBD | rs2071463 | 46.07410256 |
| IBD | rs2476601 | 41.76513956 |
| IBD | rs1077394 | 41.62166062 |
| IBD | rs1419675 | 34.68512776 |
| IBD | rs4274624 | 293.2513845 |
| IBD | rs1143679 | 212.0016865 |
| IBD | rs35000415 | 200.2294151 |
| IBD | rs6889239 | 76.51123259 |
| IBD | rs58721818 | 75.6557835 |
| IBD | rs2299851 | 61.01868032 |
| IBD | rs2431697 | 58.01521611 |
| IBD | rs6679677 | 52.39196829 |
| IBD | rs7768653 | 48.6189269 |
| IBD | rs353608 | 44.22170731 |
| IBD | rs58688157 | 44.19823106 |
| IBD | rs4916215 | 43.15163271 |
| IBD | rs7097397 | 42.11576829 |
| IBD | rs78517564 | 40.56838264 |
| IBD | rs2459611 | 33.36980543 |
| IBD | rs73068668 | 29.96640854 |
| IBD | rs2187668 | 91.39273059 |
| IBD | rs11229 | 72.57258846 |
| IBD | rs7574865 | 57.2268362 |
| IBD | rs10488631 | 53.6121581 |
| IBD | rs3132631 | 52.23451543 |
| IBD | rs16891725 | 50.9436309 |
| IBD | rs853676 | 40.64794627 |
| IBD | rs3749971 | 38.3082367 |
| IBD | rs1024582 | 73.96598273 |
| IBD | rs1615350 | 47.86839844 |
| IBD | rs11682175 | 45.46470849 |
| IBD | rs2414718 | 40.26133231 |
| IBD | rs11874716 | 37.32210249 |
| IBD | rs11210892 | 34.75563863 |

| | | |
|---|---|---|
| IBD | rs3849046 | 32.87391647 |
| IBD | rs10108725 | 30.29467814 |
| IBD | rs9268145 | 983.2118523 |
| IBD | rs485502 | 413.8945438 |
| IBD | rs2476601 | 255.0132983 |
| IBD | rs2071463 | 222.2042068 |
| IBD | rs1419675 | 109.3444597 |
| IBD | rs3184504 | 65.09399728 |
| IBD | rs11066320 | 50.67509336 |
| IBD | rs705702 | 45.1584 |
| IBD | rs11571297 | 32.42802122 |
| IBD | rs3957146 | 721.0693728 |
| IBD | rs2071463 | 80.77267157 |
| IBD | rs2476601 | 75.94293215 |
| IBD | rs2256539 | 59.24542041 |
| IBD | rs6905949 | 52.83961648 |
| IBD | rs3842727 | 892.1879561 |
| IBD | rs6679677 | 554.8636935 |
| IBD | rs3184504 | 196.000884 |
| IBD | rs61839660 | 171.9010403 |
| IBD | rs1701704 | 137.7539958 |
| IBD | rs11066320 | 120.9991893 |
| IBD | rs12927355 | 94.09039781 |
| IBD | rs3087243 | 87.76771775 |
| IBD | rs8056814 | 80.41995498 |
| IBD | rs2111485 | 75.41625824 |
| IBD | rs1893217 | 64 |
| IBD | rs11203203 | 62.32704479 |
| IBD | rs12416116 | 61.73474733 |
| IBD | rs516246 | 56.64517909 |
| IBD | rs72928038 | 56.24953121 |
| IBD | rs34593439 | 55.57010009 |
| IBD | rs4820830 | 50.48474486 |
| IBD | rs6827756 | 47.53720135 |
| IBD | rs56994090 | 46.09686261 |
| IBD | rs7239671 | 45.18840856 |
| IBD | rs2611215 | 45.15835268 |
| IBD | rs2304256 | 43.81151828 |
| IBD | rs151233 | 43.25597164 |
| IBD | rs6043409 | 39.6899937 |
| IBD | rs72727394 | 39.34692084 |
| IBD | rs2045258 | 37.91982455 |
| IBD | rs1574285 | 36.63460047 |
| IBD | rs41295121 | 33.99306255 |
| IBD | rs7893467 | 33.23180199 |
| IBD | rs12150079 | 32.49014245 |
| IBD | rs229533 | 31.71466584 |
| IBD | rs60918750 | 31.59219765 |
| IBD | rs62447205 | 31.0406647 |
| IBD | rs9585056 | 30.5125418 |
| IBD | rs4849135 | 29.98855034 |

| | | |
|---|---|---|
| IBD | rs113010081 | 29.88453674 |
| IBD | rs402072 | 29.82858008 |
| IBD | rs2596560 | 687.9581449 |
| IBD | rs6679677 | 355.8566023 |
| IBD | rs3129809 | 273.9222027 |
| IBD | rs6913635 | 214.3114623 |
| IBD | rs9275601 | 199.7820048 |
| IBD | rs9276943 | 189.7241355 |
| IBD | rs9267536 | 117.9153838 |
| IBD | rs9467704 | 117.0559971 |
| IBD | rs11571297 | 68.67304029 |
| IBD | rs741172 | 62.15305571 |
| IBD | rs2394392 | 61.12668492 |
| IBD | rs12722495 | 59.41840278 |
| IBD | rs3094138 | 41.00227505 |
| IBD | rs8056814 | 40.49865583 |
| IBD | rs2111485 | 40.43523998 |
| IBD | rs202520 | 37.75526428 |
| IBD | rs194749 | 33.9793569 |
| IBD | rs2596560 | 1498.833347 |
| IBD | rs1894406 | 838.3230436 |
| IBD | rs387608 | 465.5808626 |
| IBD | rs2535319 | 391.6576889 |
| IBD | rs2071463 | 366.3098416 |
| IBD | rs2476601 | 331.0880737 |
| IBD | rs149943 | 288.6743954 |
| IBD | rs3871466 | 150.5002563 |
| IBD | rs4759229 | 104.2639664 |
| IBD | rs12722496 | 103.4254097 |
| IBD | rs12927355 | 72.04860879 |
| IBD | rs80054410 | 53.56278678 |
| IBD | rs2111485 | 45.43818071 |
| IBD | rs12982646 | 43.70844043 |
| IBD | rs151233 | 43.61333085 |
| IBD | rs7122407 | 43.17035171 |
| IBD | rs13415465 | 39.54962553 |
| IBD | rs4238595 | 36.37755316 |
| IBD | rs34725611 | 35.43176379 |
| IBD | rs2281808 | 30.63143581 |
| IBD | rs9911533 | 30.57604133 |
| IBD | rs3024505 | 30.00770331 |
| IBD | rs9268145 | 949.1737935 |
| IBD | rs485502 | 392.5124358 |
| IBD | rs2476601 | 233.2373085 |
| IBD | rs2071463 | 197.2503956 |
| IBD | rs1419675 | 99.79392071 |
| IBD | rs3184504 | 60.28203987 |
| IBD | rs11066320 | 44.06965108 |
| IBD | rs705699 | 42.94820984 |
| IBD | rs9277932 | 40.11111111 |
| IBD | rs34337125 | 30.32751438 |

| | | |
|---|---|---|
| IBD | rs10484565 | 461.6877968 |
| IBD | rs34171662 | 190.7876263 |
| IBD | rs2476601 | 190.1168939 |
| IBD | rs3135333 | 131.6059263 |
| IBD | rs2256539 | 107.9631666 |
| IBD | rs3025643 | 86.4151886 |
| IBD | rs9268145 | 978.2427824 |
| IBD | rs485502 | 404.8277578 |
| IBD | rs2476601 | 241.5913733 |
| IBD | rs2071463 | 205.091692 |
| IBD | rs2256539 | 122.6913534 |
| IBD | rs3184504 | 57.63071073 |
| IBD | rs11066320 | 44.08734918 |
| IBD | rs11571297 | 36.55441957 |
| IBD | rs41176 | 33.42431445 |
| IBD | rs2237897 | 97.08044983 |
| IBD | rs11558471 | 53.82105098 |
| IBD | rs10938397 | 48.61892562 |
| IBD | rs80134320 | 32.86787202 |
| IBD | rs5215 | 32.59371901 |
| IBD | rs10974438 | 32.47991229 |
| IBD | rs28624681 | 54.80590109 |
| IBD | rs10938397 | 50.32507853 |
| IBD | rs11558471 | 49.11577078 |
| IBD | rs76177300 | 42.17234647 |
| IBD | rs498475 | 32.4526337 |
| IBD | rs1053924 | 31.09893974 |
| IBD | rs2383208 | 199.2238757 |
| IBD | rs13266634 | 92.73731617 |
| IBD | rs11708067 | 45.64088074 |
| IBD | rs864745 | 45.64088074 |
| IBD | rs5215 | 36.62845648 |
| IBD | rs7756992 | 276.4972058 |
| IBD | rs10811661 | 256.3266347 |
| IBD | rs849135 | 192.515625 |
| IBD | rs11708067 | 125.9092753 |
| IBD | rs17334919 | 119.2873535 |
| IBD | rs780094 | 87.4477721 |
| IBD | rs5215 | 86.26083693 |
| IBD | rs7729395 | 73.63785156 |
| IBD | rs340874 | 73.5364984 |
| IBD | rs17168486 | 62.30918968 |
| IBD | rs10974438 | 62.0944 |
| IBD | rs6795735 | 58.42822293 |
| IBD | rs2857605 | 55.75111111 |
| IBD | rs12970134 | 48.12890625 |
| IBD | rs10401969 | 47.95302165 |
| IBD | rs853974 | 46.64269112 |
| IBD | rs2071479 | 42.48040175 |
| IBD | rs67232546 | 38.54340278 |
| IBD | rs2237892 | 37.38894073 |

| | | |
|---|---|---|
| IBD | rs1265099 | 37.30898466 |
| IBD | rs9940149 | 37.27423823 |
| IBD | rs940904 | 36.14472347 |
| IBD | rs17405722 | 35.50853819 |
| IBD | rs16988333 | 32.84171598 |
| IBD | rs7561798 | 30.86419753 |
| IBD | rs7756992 | 164.8529188 |
| IBD | rs13266634 | 107.9001563 |
| IBD | rs864745 | 72.94083272 |
| IBD | rs11708067 | 72.90532544 |
| IBD | rs5215 | 65.69529086 |
| IBD | rs2943641 | 58.51256536 |
| IBD | rs60980157 | 55.99747507 |
| IBD | rs1260326 | 54.60634171 |
| IBD | rs2191349 | 50.52520088 |
| IBD | rs7202877 | 46.97263534 |
| IBD | rs340874 | 44.44444444 |
| IBD | rs1077394 | 43.32639 |
| IBD | rs35658696 | 40.86371257 |
| IBD | rs35720761 | 37.52849375 |
| IBD | rs4607517 | 33.4084 |
| IBD | rs10146997 | 32.87111111 |
| IBD | rs17782313 | 32.15405475 |
| IBD | rs6795735 | 30.39490997 |
| IBD | rs10758593 | 29.73884444 |
| IBD | rs1727307 | 24.51457466 |
| IBD | rs2237897 | 86.14160156 |
| IBD | rs3129871 | 55.65424074 |
| IBD | rs11558471 | 49.52476199 |
| IBD | rs10938397 | 43.0033284 |
| IBD | rs76177300 | 40.2824649 |
| IBD | rs2256594 | 38.14080425 |
| IBD | rs498475 | 33.48979767 |
| IBD | rs5215 | 31.74889053 |
| IBD | rs10974438 | 29.78915189 |
| IBD | rs2237897 | 96.74337269 |
| IBD | rs11558471 | 54.04236669 |
| IBD | rs10938397 | 47.07484568 |
| IBD | rs76177300 | 35.02394842 |
| IBD | rs5215 | 32.53223594 |
| IBD | rs7901695 | 952.6896859 |
| IBD | rs7756992 | 248.6686391 |
| IBD | rs13266634 | 176.733564 |
| IBD | rs864745 | 136.232666 |
| IBD | rs7501939 | 95.85041623 |
| IBD | rs1260326 | 93.44444444 |
| IBD | rs11708067 | 91.25277008 |
| IBD | rs2943641 | 77.02027178 |
| IBD | rs2191349 | 66.15111111 |
| IBD | rs10758593 | 61.6611126 |
| IBD | rs60980157 | 60.0625 |

| | | |
|---|---|---|
| IBD | rs35658696 | 56.79347511 |
| IBD | rs340874 | 55.39263639 |
| IBD | rs35720761 | 52.67022841 |
| IBD | rs2395163 | 46.63452216 |
| IBD | rs7202877 | 43.53278776 |
| IBD | rs17782313 | 39.87020408 |
| IBD | rs10146997 | 36.30834977 |
| IBD | rs730497 | 34.5337601 |
| IBD | rs1727307 | 34.17751479 |
| IBD | rs4607103 | 31.58123388 |
| IBD | rs9388489 | 26.88023669 |
| IBD | rs1065461 | 26.69444444 |
| IBD | rs665268 | 26.01 |
| IBD | rs1800961 | 23.73854938 |
| IBD | rs2237897 | 97.23333581 |
| IBD | rs11558471 | 52.82174745 |
| IBD | rs10938397 | 47.20198903 |
| IBD | rs76177300 | 35.69032402 |
| IBD | rs5215 | 32.84987997 |
| IBD | rs7901695 | 979.3217993 |
| IBD | rs7756992 | 256.4778347 |
| IBD | rs4402960 | 193.8845271 |
| IBD | rs13266634 | 174.0071367 |
| IBD | rs864745 | 116.307929 |
| IBD | rs11708067 | 115.7003616 |
| IBD | rs7501939 | 94.45410156 |
| IBD | rs2943641 | 76.37240076 |
| IBD | rs1260326 | 69.78674556 |
| IBD | rs60980157 | 60.60326871 |
| IBD | rs340874 | 59.51020408 |
| IBD | rs7202877 | 53.77777778 |
| IBD | rs35658696 | 48.76029388 |
| IBD | rs35720761 | 48.06222263 |
| IBD | rs10758593 | 40.17924037 |
| IBD | rs2395163 | 39.1426693 |
| IBD | rs6905288 | 38.05940828 |
| IBD | rs4607103 | 36.17412308 |
| IBD | rs1727307 | 32.84829583 |
| IBD | rs7961581 | 31.29510607 |
| IBD | rs1065461 | 27.49159971 |
| IBD | rs9388489 | 26.51481399 |
| IBD | rs1800961 | 25.43430241 |
| IBD | rs730497 | 24.17361111 |
| IBD | rs1421085 | 72.49375232 |
| IBD | rs13266634 | 64.67040509 |
| IBD | rs2383208 | 51.83339471 |
| IBD | rs11708067 | 46.60171771 |
| IBD | rs115505614 | 41.62012014 |
| IBD | rs72802342 | 37.12698404 |
| IBD | rs2014830 | 31.99926034 |
| IBD | rs12912777 | 31.52083868 |

| | | |
|---|---|---|
| IBD | rs515071 | 30.72245884 |
| IBD | rs1635852 | 30.60674781 |
| IBD | rs179252 | 124.0609175 |
| IBD | rs2476601 | 93.1268274 |
| IBD | rs3094067 | 84.34972299 |
| IBD | rs11571297 | 52.87566475 |
| IBD | rs2855426 | 44.11338828 |
| IBD | rs7516666 | 443.202828 |
| IBD | rs9869760 | 373.559354 |
| IBD | rs2836836 | 368.6366667 |
| IBD | rs12428112 | 72.89417023 |
| IBD | rs12421579 | 53.9085254 |
| IBD | rs12151689 | 50.28327229 |
| IBD | rs6516819 | 46.83519595 |
| IBD | rs2001823 | 46.74806385 |
| IBD | rs965513 | 46.40110627 |
| IBD | rs6060124 | 44.50478237 |
| IBD | rs4149570 | 44.15782176 |
| IBD | rs869987 | 43.57851929 |
| IBD | rs3809983 | 41.5960671 |
| IBD | rs10801825 | 41.44580956 |
| IBD | rs932311 | 40.65684167 |
| IBD | rs9290550 | 39.40310821 |
| IBD | rs241449 | 39.16755826 |
| IBD | rs2526401 | 38.62141278 |
| IBD | rs1868534 | 38.11722773 |
| IBD | rs12782349 | 37.66344954 |
| IBD | rs3828193 | 36.26520651 |
| IBD | rs2516677 | 34.65804705 |
| IBD | rs8049383 | 33.47773309 |
| IBD | rs9355255 | 32.72510609 |
| IBD | rs950910 | 30.34197834 |
| IBD | rs406113 | 30.18413893 |
| CD | rs2261033 | 1309.70595 |
| CD | rs34521355 | 619.4259078 |
| CD | rs3891175 | 340.6548467 |
| CD | rs17312661 | 329.0355684 |
| CD | rs2074475 | 251.7351789 |
| CD | rs27529 | 208.270196 |
| CD | rs4672505 | 207.3744313 |
| CD | rs9380120 | 202.8458428 |
| CD | rs7453920 | 187.3840192 |
| CD | rs2523443 | 163.8322774 |
| CD | rs7746199 | 153.3312682 |
| CD | rs17194627 | 146.3295001 |
| CD | rs3857546 | 143.5570397 |
| CD | rs7771335 | 111.647554 |
| CD | rs362521 | 104.9229818 |
| CD | rs9262222 | 90.92558971 |
| CD | rs2836883 | 69.83216632 |
| CD | rs9393646 | 68.78743718 |

| | | |
|---|---|---|
| CD | rs41299637 | 63.25861617 |
| CD | rs6600247 | 62.56710603 |
| CD | rs9901869 | 60.88986422 |
| CD | rs2239842 | 59.29114202 |
| CD | rs11190133 | 56.79210306 |
| CD | rs4129267 | 53.00824023 |
| CD | rs11624293 | 41.03960526 |
| CD | rs1041926 | 40.96172978 |
| CD | rs1860545 | 39.82087767 |
| CD | rs35164067 | 39.41475927 |
| CD | rs2927613 | 38.00117864 |
| CD | rs12615545 | 37.26600201 |
| CD | rs1801274 | 36.74091877 |
| CD | rs1250550 | 36.5824539 |
| CD | rs7191548 | 34.00021582 |
| CD | rs7282490 | 33.76681929 |
| CD | rs1128905 | 33.54905971 |
| CD | rs6915101 | 33.53582202 |
| CD | rs4676410 | 32.86114726 |
| CD | rs11065898 | 29.83296349 |
| CD | rs6881706 | 122.0334473 |
| CD | rs56375023 | 115.5625 |
| CD | rs7224129 | 88.09633733 |
| CD | rs848 | 67.78025896 |
| CD | rs12365699 | 57.60420776 |
| CD | rs2228145 | 41.35820452 |
| CD | rs6011033 | 40.11111111 |
| CD | rs249677 | 38.48204539 |
| CD | rs45613035 | 37.73469388 |
| CD | rs4845604 | 34.16680839 |
| CD | rs17664743 | 33.34655822 |
| CD | rs12740374 | 74.62841521 |
| CD | rs10774625 | 55.96924896 |
| CD | rs11591147 | 42.23149781 |
| CD | rs943689 | 15331.36608 |
| CD | rs174535 | 14925.55714 |
| CD | rs11742240 | 13095.58337 |
| CD | rs6866614 | 12003.57035 |
| CD | rs4771332 | 10973.31764 |
| CD | rs72743461 | 8550.412667 |
| CD | rs17454584 | 8543.773292 |
| CD | rs17668708 | 6537.486118 |
| CD | rs57585717 | 5200.061381 |
| CD | rs62626322 | 4884.227913 |
| CD | rs4795399 | 12651.68545 |
| CD | rs11065979 | 8666.64348 |
| CD | rs7100984 | 7384.392732 |
| CD | rs9391997 | 6447.57544 |
| CD | rs912425 | 6359.282939 |
| CD | rs10786050 | 6096.274685 |
| CD | rs449454 | 6010.474415 |

| | | |
|---|---|---|
| CD | rs705700 | 5925.1174 |
| CD | rs10995245 | 5865.850642 |
| CD | rs35570272 | 5785.519507 |
| CD | rs3997849 | 5673.511839 |
| CD | rs12365699 | 5421.749137 |
| CD | rs4917131 | 5333.142509 |
| CD | rs11236797 | 5009.390268 |
| CD | rs4845604 | 4729.476999 |
| CD | rs55743914 | 4442.172618 |
| CD | rs2738783 | 4177.728664 |
| CD | rs72743461 | 3725.129751 |
| CD | rs9275602 | 3457.002739 |
| CD | rs17611877 | 2190.374927 |
| CD | rs4705869 | 2030.281908 |
| CD | rs45613035 | 1816.028893 |
| CD | rs10118244 | 1220.032642 |
| CD | rs4065275 | 60.0625 |
| CD | rs4851582 | 37.83680368 |
| CD | rs17228058 | 31.19476369 |
| CD | rs56375023 | 180.8381491 |
| CD | rs848 | 129.3111611 |
| CD | rs943689 | 105.8320661 |
| CD | rs35570272 | 57.79741471 |
| CD | rs17454584 | 57.42021622 |
| CD | rs2229094 | 46.45601899 |
| CD | rs2296618 | 41.30204088 |
| CD | rs11742240 | 37.99541679 |
| CD | rs6922431 | 37.29855251 |
| CD | rs12365699 | 37.04297066 |
| CD | rs3130196 | 36.07438813 |
| CD | rs1800797 | 35.8317146 |
| CD | rs2255088 | 35.22255884 |
| CD | rs11042902 | 33.78044012 |
| CD | rs7219483 | 33.40236018 |
| CD | rs2275806 | 30.53114472 |
| CD | rs10912564 | 30.48300256 |
| CD | rs4787426 | 30.05105399 |
| CD | rs11679146 | 75.00572944 |
| CD | rs12949100 | 70.13310017 |
| CD | rs10900809 | 45.65419851 |
| CD | rs72743461 | 40.34409125 |
| CD | rs34972832 | 34.07344531 |
| CD | rs3781094 | 32.19627276 |
| CD | rs76830965 | 751.4926184 |
| CD | rs2523554 | 95.59110318 |
| CD | rs17552787 | 73.16962599 |
| CD | rs17282175 | 62.12034288 |
| CD | rs913678 | 56.484542 |
| CD | rs9261425 | 48.62943421 |
| CD | rs436857 | 42.88770149 |
| CD | rs10513548 | 32.5228789 |

| | | |
|---|---|---|
| CD | rs2305479 | 187.7155729 |
| CD | rs3771180 | 86.39709541 |
| CD | rs17293632 | 64.6798294 |
| CD | rs2155219 | 62.33605557 |
| CD | rs20541 | 59.28971257 |
| CD | rs2596464 | 54.22461405 |
| CD | rs2325291 | 51.14465884 |
| CD | rs2855812 | 39.80707175 |
| CD | rs7705042 | 37.63508136 |
| CD | rs2523716 | 32.21965276 |
| CD | rs241429 | 30.72053745 |
| CD | rs12740374 | 137.8754864 |
| CD | rs10774625 | 99.02658713 |
| CD | rs11591147 | 67.90742618 |
| CD | rs2681472 | 50.46833153 |
| CD | rs72743461 | 43.38995462 |
| CD | rs17115100 | 37.29062542 |
| CD | rs13402621 | 37.12083809 |
| CD | rs748431 | 30.2782117 |
| CD | rs2160203 | 101.5586578 |
| CD | rs20541 | 52.13554725 |
| CD | rs879606 | 36.57155192 |
| CD | rs10806425 | 34.9088659 |
| CD | rs62322662 | 33.49912916 |
| CD | rs6010620 | 63.17508918 |
| CD | rs17513531 | 34.8043543 |
| CD | rs61839660 | 33.13625904 |
| CD | rs2476601 | 695.271424 |
| CD | rs3184504 | 255.6445679 |
| CD | rs11066320 | 212.835679 |
| CD | rs2596503 | 156.4693752 |
| CD | rs11571297 | 145.3073015 |
| CD | rs9277557 | 128.376993 |
| CD | rs28780083 | 77.82161548 |
| CD | rs10905669 | 66.97251145 |
| CD | rs1990760 | 56.08345679 |
| CD | rs229541 | 54.76 |
| CD | rs13203715 | 50.30862245 |
| CD | rs4073285 | 39.33471172 |
| CD | rs75438091 | 37.34567901 |
| CD | rs2293158 | 33.0625 |
| CD | rs7205474 | 30.53515161 |
| CD | rs9392026 | 100.500894 |
| CD | rs3778607 | 81.22837799 |
| CD | rs3087243 | 76.72820712 |
| CD | rs72928038 | 71.13282576 |
| CD | rs162298 | 54.06345131 |
| CD | rs2049218 | 46.42533891 |
| CD | rs2476601 | 39.76934144 |
| CD | rs4682103 | 32.62425174 |
| CD | rs10994397 | 38.50900594 |

| | | |
|---|---|---|
| CD | rs7296288 | 32.95192315 |
| CD | rs12576775 | 30.86928367 |
| CD | rs7258465 | 120.6668962 |
| CD | rs7017073 | 58.16604444 |
| CD | rs9952980 | 54.07905325 |
| CD | rs4971059 | 43.890625 |
| CD | rs3749971 | 33.78515625 |
| CD | rs61744064 | 32.92125773 |
| CD | rs17426269 | 32.06719849 |
| CD | rs666930 | 30.33736458 |
| CD | rs414851 | 10679.99029 |
| CD | rs17076598 | 8944.242115 |
| CD | rs3759333 | 7956.728479 |
| CD | rs26433 | 7862.218435 |
| CD | rs3800393 | 6135.983387 |
| CD | rs12606193 | 4627.748927 |
| CD | rs11864719 | 4513.373015 |
| CD | rs17450969 | 4142.522261 |
| CD | rs592719 | 3790.1959 |
| CD | rs6880335 | 2343.940734 |
| CD | rs2109897 | 2066.055573 |
| CD | rs12650313 | 1930.49442 |
| CD | rs10519288 | 1613.473299 |
| CD | rs16839999 | 578.9153819 |
| CD | rs17238590 | 527.3553142 |
| CD | rs16941631 | 341.6692449 |
| CD | rs72905734 | 32.99332008 |
| CD | rs4468572 | 65.71142838 |
| CD | rs2681472 | 42.76643653 |
| CD | rs11065979 | 40.54015268 |
| CD | rs11191416 | 34.33208586 |
| CD | rs515135 | 30.64899745 |
| CD | rs599839 | 39.75093937 |
| CD | rs1122608 | 37.37777487 |
| CD | rs2306374 | 30.49755064 |
| CD | rs11591147 | 45.84997549 |
| CD | rs653178 | 45.11184822 |
| CD | rs12705390 | 42.67808621 |
| CD | rs11591147 | 42.77237626 |
| CD | rs653178 | 37.6372512 |
| CD | rs12705390 | 35.29240055 |
| CD | rs9392026 | 108.0394805 |
| CD | rs3778607 | 84.45035746 |
| CD | rs72928038 | 53.06870018 |
| CD | rs3087243 | 52.44553257 |
| CD | rs204295 | 42.22106729 |
| CD | rs2476601 | 33.29128367 |
| CD | rs9851967 | 95.52135384 |
| CD | rs17810546 | 75.06089693 |
| CD | rs13151961 | 74.42239571 |
| CD | rs653178 | 56.36192249 |

| | | |
|---|---|---|
| CD | rs2816316 | 50.11751 |
| CD | rs13098911 | 44.51210622 |
| CD | rs11221335 | 43.53962169 |
| CD | rs1018326 | 34.73256097 |
| CD | rs1738074 | 30.61653045 |
| CD | rs13003464 | 29.74740267 |
| CD | rs416352 | 1479.738185 |
| CD | rs1265099 | 1479.736498 |
| CD | rs1264622 | 1479.735003 |
| CD | rs3117326 | 1479.733708 |
| CD | rs521977 | 1479.72934 |
| CD | rs9366717 | 1290.54482 |
| CD | rs2523534 | 1240.027985 |
| CD | rs1264344 | 1172.828436 |
| CD | rs13219354 | 1156.538829 |
| CD | rs2523693 | 1137.865427 |
| CD | rs13198474 | 920.7933941 |
| CD | rs3130357 | 843.5232897 |
| CD | rs23544 | 841.2168935 |
| CD | rs16869648 | 647.6503087 |
| CD | rs1383265 | 557.4883494 |
| CD | rs9296068 | 324.1139529 |
| CD | rs2030519 | 217.6129001 |
| CD | rs13132308 | 168.1506364 |
| CD | rs2235982 | 165.2298147 |
| CD | rs4947332 | 154.9639815 |
| CD | rs2017525 | 143.4783783 |
| CD | rs17264332 | 129.6121552 |
| CD | rs76830965 | 117.2233821 |
| CD | rs1323292 | 107.0991357 |
| CD | rs4148881 | 102.2553076 |
| CD | rs3184504 | 88.37382666 |
| CD | rs2097282 | 86.92046105 |
| CD | rs2296748 | 83.2303368 |
| CD | rs6934794 | 83.09811547 |
| CD | rs499384 | 81.213784 |
| CD | rs55743914 | 77.79128697 |
| CD | rs1250552 | 69.41738357 |
| CD | rs990171 | 68.56934627 |
| CD | rs12530421 | 66.9080154 |
| CD | rs1018326 | 66.76226128 |
| CD | rs13003464 | 66.0759525 |
| CD | rs182429 | 64.75391564 |
| CD | rs1980422 | 63.72965887 |
| CD | rs6910898 | 58.48921424 |
| CD | rs11066320 | 55.09582463 |
| CD | rs17404424 | 53.86812827 |
| CD | rs61907765 | 52.94579441 |
| CD | rs4947256 | 52.88211844 |
| CD | rs1929848 | 52.16257946 |
| CD | rs16894128 | 49.55212246 |

| | | |
|---|---|---|
| CD | rs4445406 | 47.52775009 |
| CD | rs10892258 | 45.25034448 |
| CD | rs7104791 | 45.08247447 |
| CD | rs4821124 | 42.9125254 |
| CD | rs2267632 | 41.90765533 |
| CD | rs12068671 | 41.16791264 |
| CD | rs11875687 | 40.54953979 |
| CD | rs1570760 | 40.35551091 |
| CD | rs1050976 | 36.13798535 |
| CD | rs6933319 | 35.44836417 |
| CD | rs1893592 | 35.20800313 |
| CD | rs7616215 | 33.45953222 |
| CD | rs1378938 | 33.32651768 |
| CD | rs1107943 | 33.28780669 |
| CD | rs6715106 | 33.18500134 |
| CD | rs61579022 | 32.85683346 |
| CD | rs16894722 | 31.86578322 |
| CD | rs11801183 | 31.82516941 |
| CD | rs79758729 | 31.38524655 |
| CD | rs10800746 | 31.00741953 |
| CD | rs744254 | 30.68406801 |
| CD | rs10513548 | 30.03920125 |
| CD | rs11851414 | 29.834606 |
| CD | rs3130380 | 2488.026097 |
| CD | rs3130834 | 1895.744874 |
| CD | rs9469220 | 1626.502217 |
| CD | rs9268368 | 1390.544471 |
| CD | rs9276612 | 1193.007016 |
| CD | rs13219354 | 1156.678473 |
| CD | rs4540292 | 1067.331181 |
| CD | rs13198474 | 920.515888 |
| CD | rs16869648 | 647.6977466 |
| CD | rs2395296 | 549.4338273 |
| CD | rs2395349 | 405.216881 |
| CD | rs3734840 | 291.3839027 |
| CD | rs2229092 | 230.1286855 |
| CD | rs11753326 | 209.9604293 |
| CD | rs3097657 | 171.3483583 |
| CD | rs13205699 | 137.8277567 |
| CD | rs9258557 | 131.7905808 |
| CD | rs9380294 | 129.0496726 |
| CD | rs9501234 | 118.5916253 |
| CD | rs17351888 | 100 |
| CD | rs2296748 | 83.22947696 |
| CD | rs6934794 | 83.10086982 |
| CD | rs12530421 | 66.91249607 |
| CD | rs12527282 | 54.34636206 |
| CD | rs16894128 | 49.54785641 |
| CD | rs9658119 | 49.18193628 |
| CD | rs10892258 | 45.25278712 |
| CD | rs7104791 | 45.07798795 |

| | | |
|---|---|---|
| CD | rs12665489 | 44.715982 |
| CD | rs2267632 | 41.91254141 |
| CD | rs7761745 | 38.36563639 |
| CD | rs1611697 | 36.36086417 |
| CD | rs10752747 | 34.17580435 |
| CD | rs7162232 | 33.28148309 |
| CD | rs16894722 | 31.86599681 |
| CD | rs2441467 | 31.80993363 |
| CD | rs11757750 | 31.25940326 |
| CD | rs13030124 | 31.13618677 |
| CD | rs11979905 | 31.0695865 |
| CD | rs9851967 | 95.32510474 |
| CD | rs17810546 | 74.65691017 |
| CD | rs13151961 | 73.67023001 |
| CD | rs653178 | 56.0625039 |
| CD | rs2816316 | 49.85991365 |
| CD | rs13098911 | 44.23540633 |
| CD | rs1018326 | 34.4837366 |
| CD | rs1738074 | 30.77181067 |
| CD | rs13003464 | 29.37484965 |
| CD | rs13091753 | 66.34364786 |
| CD | rs6933404 | 51.49762868 |
| CD | rs13132933 | 46.43165397 |
| CD | rs1468791 | 45.04994231 |
| CD | rs11221335 | 43.39365361 |
| CD | rs9819226 | 38.95214004 |
| CD | rs1323292 | 34.38686922 |
| CD | rs9851967 | 95.32510474 |
| CD | rs17810546 | 74.65691017 |
| CD | rs13151961 | 73.67023001 |
| CD | rs653178 | 56.0625039 |
| CD | rs2816316 | 49.85991365 |
| CD | rs13098911 | 44.23540633 |
| CD | rs11221335 | 43.39365361 |
| CD | rs1018326 | 34.4837366 |
| CD | rs1738074 | 30.77181067 |
| CD | rs13003464 | 29.37484965 |
| CD | rs6983267 | 91.83428037 |
| CD | rs4939827 | 70.97770955 |
| CD | rs16892766 | 35.04778755 |
| CD | rs3957146 | 670.2345679 |
| CD | rs2523504 | 82.30249168 |
| CD | rs2237897 | 76.41833716 |
| CD | rs2476601 | 66.35707816 |
| CD | rs11558471 | 45.99794138 |
| CD | rs10938397 | 43.04966681 |
| CD | rs17187735 | 38.90640625 |
| CD | rs76177300 | 36.51612245 |
| CD | rs10974438 | 36.23801588 |
| CD | rs34337125 | 32.2624 |
| CD | rs9765960 | 30.15685148 |

| | | |
|---|---|---|
| CD | rs5215 | 30.13785923 |
| CD | rs3957146 | 537.1139442 |
| CD | rs2476601 | 77.30128082 |
| CD | rs34171662 | 69.12311447 |
| CD | rs17187735 | 44.72780591 |
| CD | rs2296329 | 34.84133448 |
| CD | rs878898 | 33.08058423 |
| CD | rs3957146 | 659.0029432 |
| CD | rs2523504 | 80.02994887 |
| CD | rs2237897 | 77.04938272 |
| CD | rs2476601 | 64.58958694 |
| CD | rs11558471 | 46.1041 |
| CD | rs10938397 | 45.21876302 |
| CD | rs11708067 | 41.95053254 |
| CD | rs17187735 | 37.98478668 |
| CD | rs10974438 | 37.07724733 |
| CD | rs76177300 | 35.31430141 |
| CD | rs56258397 | 32.76333321 |
| CD | rs34337125 | 29.97285991 |
| CD | rs5215 | 29.58027905 |
| CD | rs9273363 | 219.6369847 |
| CD | rs1421085 | 95.67277557 |
| CD | rs849142 | 62.07355484 |
| CD | rs1260326 | 51.3808817 |
| CD | rs697239 | 47.68540027 |
| CD | rs5215 | 41.04837291 |
| CD | rs1800961 | 36.67443398 |
| CD | rs3131064 | 33.03226561 |
| CD | rs2476601 | 30.65820455 |
| CD | rs646123 | 29.81313658 |
| CD | rs3957146 | 609.261834 |
| CD | rs2237897 | 81.56643184 |
| CD | rs2523504 | 78.25443787 |
| CD | rs2476601 | 65.34027778 |
| CD | rs4402960 | 54.57349241 |
| CD | rs10938397 | 47.24893958 |
| CD | rs11558471 | 42.25 |
| CD | rs76177300 | 36.1630802 |
| CD | rs17187735 | 34.8239935 |
| CD | rs853974 | 34.56629013 |
| CD | rs5215 | 31.49062117 |
| CD | rs9765960 | 30.74881374 |
| CD | rs3957146 | 446.3578212 |
| CD | rs2476601 | 67.27738411 |
| CD | rs34018127 | 64.05307899 |
| CD | rs3873444 | 58.75570162 |
| CD | rs2107202 | 35.36190334 |
| CD | rs17187735 | 33.03913015 |
| CD | rs4795399 | 213.1525795 |
| CD | rs2197415 | 166.0926748 |
| CD | rs72743461 | 164.0231007 |

| | | |
|---|---|---|
| CD | rs1321859 | 104.035551 |
| CD | rs2621322 | 76.05759964 |
| CD | rs1117490 | 66.23317075 |
| CD | rs17454584 | 59.77524667 |
| CD | rs917115 | 49.25872218 |
| CD | rs35570272 | 48.80394015 |
| CD | rs72799426 | 43.89843058 |
| CD | rs174535 | 39.46309793 |
| CD | rs2338819 | 37.18354855 |
| CD | rs429916 | 36.72699383 |
| CD | rs7771953 | 36.00951948 |
| CD | rs13208164 | 35.02525737 |
| CD | rs41284471 | 34.87325903 |
| CD | rs805287 | 34.14574701 |
| CD | rs2296618 | 33.32483733 |
| CD | rs3781094 | 32.70625219 |
| CD | rs6729966 | 31.35010616 |
| CD | rs12365699 | 29.98577268 |
| CD | rs9375459 | 159.8205842 |
| CD | rs11072508 | 124.9436252 |
| CD | rs1052486 | 89.3645148 |
| CD | rs7838131 | 58.06523158 |
| CD | rs11864054 | 45.13135868 |
| CD | rs169080 | 44.7912553 |
| CD | rs2246490 | 39.93143088 |
| CD | rs11229555 | 37.49582107 |
| CD | rs27433 | 37.12780233 |
| CD | rs35633336 | 37.00116955 |
| CD | rs57786342 | 32.27754701 |
| CD | rs10072700 | 32.14303234 |
| CD | rs6905288 | 31.8339008 |
| CD | rs74580656 | 31.26109983 |
| CD | rs4146810 | 31.07499639 |
| CD | rs696 | 30.38757938 |
| CD | rs13003198 | 29.72938169 |
| CD | rs11690644 | 247.8241064 |
| CD | rs11742240 | 198.5205289 |
| CD | rs61839660 | 137.9976273 |
| CD | rs72743461 | 90.09936414 |
| CD | rs4845604 | 81.45904723 |
| CD | rs45613035 | 77.33398792 |
| CD | rs2844787 | 69.45999167 |
| CD | rs11171739 | 67.83227124 |
| CD | rs653178 | 63.85847713 |
| CD | rs2228145 | 57.33363979 |
| CD | rs905670 | 52.97274528 |
| CD | rs10912564 | 52.80210761 |
| CD | rs17057868 | 49.83749708 |
| CD | rs10903115 | 47.60590435 |
| CD | rs16858573 | 46.35375184 |
| CD | rs5029949 | 36.47550325 |

| | | |
|---|---|---|
| CD | rs12365699 | 35.85211005 |
| CD | rs4939489 | 34.84480371 |
| CD | rs2255088 | 33.70206102 |
| CD | rs2893907 | 32.23625983 |
| CD | rs647397 | 31.11630209 |
| CD | rs2422254 | 30.01641747 |
| CD | rs34463936 | 177.358569 |
| CD | rs72743461 | 152.2769204 |
| CD | rs962993 | 121.0171896 |
| CD | rs61839660 | 106.7358089 |
| CD | rs4845604 | 79.72036465 |
| CD | rs905670 | 77.67955622 |
| CD | rs849335 | 68.72827897 |
| CD | rs45613035 | 66.44838124 |
| CD | rs10903115 | 58.32674249 |
| CD | rs2228145 | 56.70650655 |
| CD | rs6025 | 51.33053693 |
| CD | rs17057868 | 51.16765851 |
| CD | rs10912564 | 45.20357895 |
| CD | rs249677 | 44.6207053 |
| CD | rs12365699 | 43.94522771 |
| CD | rs6011033 | 43.92054271 |
| CD | rs5029949 | 41.58508412 |
| CD | rs4410871 | 39.48207075 |
| CD | rs2255088 | 38.23508331 |
| CD | rs16858573 | 38.20229185 |
| CD | rs2893907 | 35.54250255 |
| CD | rs11256311 | 33.1705323 |
| CD | rs1177289 | 31.81903734 |
| CD | rs28360011 | 31.51135145 |
| CD | rs72799426 | 31.00951592 |
| CD | rs114420077 | 30.05833404 |
| CD | rs17538393 | 29.7510625 |
| CD | rs4939827 | 89.14101097 |
| CD | rs6983267 | 62.09929775 |
| CD | rs10936599 | 32.11321132 |
| CD | rs11591147 | 118.0998178 |
| CD | rs646776 | 82.44194362 |
| CD | rs499883 | 68.2473268 |
| CD | rs1367117 | 65.30387144 |
| CD | rs4953023 | 48.02547037 |
| CD | rs12916 | 36.36180677 |
| CD | rs2476601 | 401.7161224 |
| CD | rs11571297 | 97.87548308 |
| CD | rs115084931 | 50.83753946 |
| CD | rs1990760 | 40.90932262 |
| CD | rs10905668 | 38.66578512 |
| CD | rs28780083 | 37.02999096 |
| CD | rs11964080 | 33.75907955 |
| CD | rs11102634 | 32.18297214 |
| CD | rs4409785 | 30.37418735 |

| | | |
|---|---|---|
| CD | rs1800961 | 47.48195767 |
| CD | rs1260326 | 37.88932141 |
| CD | rs1169286 | 34.98169542 |
| CD | rs11591147 | 81.82562262 |
| CD | rs646776 | 54.243225 |
| CD | rs1367117 | 46.10485028 |
| CD | rs499883 | 45.39801457 |
| CD | rs688 | 32.57346817 |
| CD | rs12916 | 31.60633551 |
| CD | rs646776 | 188.9443931 |
| CD | rs1367117 | 69.49341629 |
| CD | rs1260326 | 68.65754422 |
| CD | rs11591147 | 60.64810791 |
| CD | rs4245791 | 58.01732088 |
| CD | rs12916 | 36.35360002 |
| CD | rs2244608 | 31.25966387 |
| CD | rs2240326 | 96.02888374 |
| CD | rs773109 | 55.63788661 |
| CD | rs2232423 | 45.70598484 |
| CD | rs1716171 | 42.30125524 |
| CD | rs6722661 | 41.55473241 |
| CD | rs205262 | 41.19322308 |
| CD | rs329122 | 35.14855924 |
| CD | rs3172494 | 33.61569586 |
| CD | rs9615905 | 32.46700519 |
| CD | rs2164300 | 30.0858308 |
| CD | rs17293632 | 139.3426276 |
| CD | rs9847710 | 99.500625 |
| CD | rs72707023 | 53.31755073 |
| CD | rs3072 | 42.40868531 |
| CD | rs1262776 | 41.24493827 |
| CD | rs10460504 | 38.9138322 |
| CD | rs10838738 | 36.58774539 |
| CD | rs2476601 | 269.7717944 |
| CD | rs3087243 | 80.62801427 |
| CD | rs9277557 | 47.87360086 |
| CD | rs2111485 | 33.52242176 |
| CD | rs10974437 | 31.90387634 |
| CD | rs7599564 | 31.55092802 |
| CD | rs28780083 | 29.84229219 |
| CD | rs2476601 | 273.398267 |
| CD | rs3087243 | 82.113389 |
| CD | rs9277557 | 47.24587132 |
| CD | rs117846960 | 34.64073817 |
| CD | rs10974437 | 31.82431474 |
| CD | rs7599564 | 31.81913311 |
| CD | rs28780083 | 30.25 |
| CD | rs2476601 | 259.5679012 |
| CD | rs3184504 | 103.8145002 |
| CD | rs11066320 | 85.7971076 |
| CD | rs11571297 | 65.74141709 |

| | | |
|---|---|---|
| CD | rs9277557 | 56.33244777 |
| CD | rs10174238 | 34.62689481 |
| CD | rs2523719 | 32.02530992 |
| CD | rs2476601 | 438.5166638 |
| CD | rs11571297 | 109.5888321 |
| CD | rs9277542 | 72.78222656 |
| CD | rs229541 | 61.12396694 |
| CD | rs1990760 | 49.64430603 |
| CD | rs7757496 | 47.48803739 |
| CD | rs28780083 | 45.47419205 |
| CD | rs10905669 | 42.41402443 |
| CD | rs877636 | 39.78698225 |
| CD | rs10748781 | 38.37418749 |
| CD | rs4409785 | 38.28515625 |
| CD | rs210179 | 32.49974432 |
| CD | rs11102634 | 29.96121884 |
| CD | rs2476601 | 475.24 |
| CD | rs11571297 | 117.9034028 |
| CD | rs9277542 | 80.21359689 |
| CD | rs229541 | 61.44965527 |
| CD | rs1990760 | 59.69873621 |
| CD | rs28780083 | 43.34027778 |
| CD | rs10748781 | 41.61307444 |
| CD | rs4409785 | 40.74418114 |
| CD | rs41295131 | 39.76626436 |
| CD | rs10517086 | 39.55230713 |
| CD | rs210192 | 30.25 |
| CD | rs2476601 | 437.9855611 |
| CD | rs11571297 | 110.4375797 |
| CD | rs9277542 | 78.765625 |
| CD | rs229541 | 62.98586777 |
| CD | rs1990760 | 51.73436579 |
| CD | rs28780083 | 47.55251736 |
| CD | rs2442719 | 44.72265625 |
| CD | rs10748781 | 41.1173438 |
| CD | rs877636 | 41.09138725 |
| CD | rs4409785 | 40.02319637 |
| CD | rs6448432 | 38.95840278 |
| CD | rs210179 | 32.49974432 |
| CD | rs11102634 | 30.82967382 |
| CD | rs12567136 | 174.6834486 |
| CD | rs11072508 | 135.5905439 |
| CD | rs7838131 | 67.47701147 |
| CD | rs3764769 | 46.70771246 |
| CD | rs169080 | 42.21912725 |
| CD | rs35633336 | 36.17134696 |
| CD | rs736801 | 34.30657189 |
| CD | rs10409243 | 33.90872489 |
| CD | rs4713488 | 33.54699275 |
| CD | rs27433 | 32.72883438 |
| CD | rs696 | 31.46061655 |

| | | |
|---|---|---|
| CD | rs11229555 | 31.07838325 |
| CD | rs11591147 | 57.5872865 |
| CD | rs12705390 | 53.29 |
| CD | rs653178 | 34.93654337 |
| CD | rs2495477 | 33.45215486 |
| CD | rs1044506 | 33.04486432 |
| CD | rs3957146 | 779.2267995 |
| CD | rs1121980 | 104.1940192 |
| CD | rs2523504 | 96.85840278 |
| CD | rs2237897 | 77.00957934 |
| CD | rs2476601 | 76.44430241 |
| CD | rs10938397 | 50.73202207 |
| CD | rs9765960 | 43.71837714 |
| CD | rs28624681 | 41.5308642 |
| CD | rs11558471 | 41.00698594 |
| CD | rs76177300 | 38.86915577 |
| CD | rs4148881 | 36.39290296 |
| CD | rs7100984 | 32.33560758 |
| CD | rs34337125 | 31.75901825 |
| CD | rs4273712 | 31.66446423 |
| CD | rs3957146 | 778.6116998 |
| CD | rs1121980 | 104.3452703 |
| CD | rs2523504 | 97.8627826 |
| CD | rs2237897 | 80.90911642 |
| CD | rs2476601 | 76.23985406 |
| CD | rs10938397 | 51.51751245 |
| CD | rs11558471 | 42.72404959 |
| CD | rs9765960 | 41.58026159 |
| CD | rs76177300 | 39.43181862 |
| CD | rs4148881 | 34.65790843 |
| CD | rs34337125 | 33.16906722 |
| CD | rs853974 | 31.17361111 |
| CD | rs7100984 | 30.8025 |
| CD | rs9271100 | 1153.78976 |
| CD | rs2857700 | 930.1440187 |
| CD | rs9469220 | 808.7316572 |
| CD | rs9266629 | 238.0532385 |
| CD | rs722788 | 129.5756482 |
| CD | rs12927355 | 114.0780906 |
| CD | rs9277641 | 104.8002141 |
| CD | rs2104286 | 95.65667554 |
| CD | rs13194491 | 79.16415234 |
| CD | rs1813375 | 74.64878281 |
| CD | rs2394423 | 72.05492404 |
| CD | rs41286801 | 64.78177037 |
| CD | rs1800693 | 64.71669962 |
| CD | rs8070345 | 64.71669962 |
| CD | rs1920296 | 59.88255844 |
| CD | rs11554159 | 53.94065835 |
| CD | rs1077667 | 53.28033723 |
| CD | rs2255214 | 52.36224806 |

| | | |
|---|---|---|
| CD | rs4976646 | 51.53151101 |
| CD | rs3748817 | 50.68870328 |
| CD | rs7762619 | 49.77234597 |
| CD | rs3129269 | 49.43856505 |
| CD | rs17066096 | 47.45176818 |
| CD | rs12087340 | 47.25406297 |
| CD | rs11172342 | 46.2049889 |
| CD | rs2071292 | 46.07892099 |
| CD | rs74796499 | 41.9955859 |
| CD | rs6498184 | 39.62359077 |
| CD | rs34383631 | 39.44266262 |
| CD | rs1870071 | 38.54918016 |
| CD | rs1021156 | 38.15633434 |
| CD | rs2288904 | 37.9201961 |
| CD | rs1014486 | 36.84544637 |
| CD | rs9967792 | 36.2725284 |
| CD | rs11154801 | 36.20743486 |
| CD | rs4410871 | 36.14245546 |
| CD | rs55838263 | 36.0921596 |
| CD | rs842639 | 35.61351815 |
| CD | rs71624119 | 35.10542979 |
| CD | rs7923837 | 34.81640662 |
| CD | rs941816 | 34.81320201 |
| CD | rs6881706 | 34.0714384 |
| CD | rs6880778 | 32.36681908 |
| CD | rs9380294 | 32.22777182 |
| CD | rs917116 | 31.70531526 |
| CD | rs4796791 | 31.5258197 |
| CD | rs6933319 | 30.42722174 |
| CD | rs3748080 | 29.73972414 |
| CD | rs67297943 | 29.52202688 |
| CD | rs9271100 | 1151.631086 |
| CD | rs2857700 | 921.3435307 |
| CD | rs9469220 | 814.5751003 |
| CD | rs9266629 | 240.2826901 |
| CD | rs722788 | 128.8075972 |
| CD | rs12927355 | 114.9205234 |
| CD | rs9277641 | 105.0513448 |
| CD | rs2104286 | 96.81623181 |
| CD | rs3135333 | 88.71538787 |
| CD | rs13194491 | 78.0008487 |
| CD | rs1813375 | 74.60467438 |
| CD | rs2394423 | 73.02581602 |
| CD | rs8070345 | 65.6342256 |
| CD | rs1800693 | 65.15622698 |
| CD | rs41286801 | 64.88992935 |
| CD | rs212407 | 62.57878754 |
| CD | rs57271503 | 61.6464492 |
| CD | rs1920296 | 60.66954042 |
| CD | rs11554159 | 53.5042742 |
| CD | rs1077667 | 52.88306485 |

| | | |
|---|---|---|
| CD | rs1323292 | 52.80705178 |
| CD | rs2255214 | 52.07945023 |
| CD | rs4976646 | 50.76723335 |
| CD | rs3748817 | 50.28489445 |
| CD | rs7762619 | 49.75718908 |
| CD | rs12087340 | 47.63652841 |
| CD | rs17066096 | 47.3591021 |
| CD | rs11172342 | 46.43729685 |
| CD | rs2071292 | 46.00408962 |
| CD | rs74796499 | 42.14598704 |
| CD | rs6498184 | 40.39956714 |
| CD | rs1021156 | 38.45620754 |
| CD | rs1870071 | 38.42876024 |
| CD | rs34383631 | 38.42542586 |
| CD | rs2745400 | 37.80488558 |
| CD | rs2288904 | 37.41094708 |
| CD | rs1014486 | 37.03575872 |
| CD | rs2364482 | 36.66893084 |
| CD | rs55838263 | 36.65497369 |
| CD | rs842639 | 36.29036119 |
| CD | rs9967792 | 36.17935672 |
| CD | rs4410871 | 35.99321002 |
| CD | rs11154801 | 35.65969173 |
| CD | rs71624119 | 35.38911173 |
| CD | rs941816 | 34.40743197 |
| CD | rs7923837 | 34.36020519 |
| CD | rs6881706 | 34.24060615 |
| CD | rs9380294 | 32.18739863 |
| CD | rs6880778 | 31.8103382 |
| CD | rs4796791 | 31.68833603 |
| CD | rs917116 | 31.42801881 |
| CD | rs1059288 | 31.3452092 |
| CD | rs6933319 | 30.45318115 |
| CD | rs67297943 | 29.78362904 |
| CD | rs3134954 | 1116.320754 |
| CD | rs2844670 | 388.3801045 |
| CD | rs16896742 | 229.2913396 |
| CD | rs404860 | 163.5528652 |
| CD | rs9276711 | 122.2844658 |
| CD | rs9348876 | 79.48075962 |
| CD | rs17500468 | 62.48042532 |
| CD | rs4648356 | 57.68892884 |
| CD | rs7200786 | 56.26968676 |
| CD | rs4613763 | 56.06171561 |
| CD | rs13194491 | 53.66838073 |
| CD | rs11154801 | 50.04225264 |
| CD | rs1077667 | 49.37731631 |
| CD | rs669607 | 44.17564017 |
| CD | rs1738074 | 43.06640625 |
| CD | rs396090 | 42.91176937 |
| CD | rs1800693 | 40.65524787 |

| | | |
|---|---|---|
| CD | rs17066096 | 39.43963441 |
| CD | rs2119704 | 39.39924413 |
| CD | rs11581062 | 39.23439198 |
| CD | rs2293370 | 37.18024985 |
| CD | rs9282641 | 36.49615105 |
| CD | rs650258 | 36.2064142 |
| CD | rs1335532 | 35.95597677 |
| CD | rs3118470 | 35.93785068 |
| CD | rs2248359 | 34.11927075 |
| CD | rs11129295 | 32.58482078 |
| CD | rs4902647 | 30.27350884 |
| CD | rs874628 | 29.99117201 |
| CD | rs2857700 | 1043.603238 |
| CD | rs4148874 | 686.7725682 |
| CD | rs2844787 | 124.5897171 |
| CD | rs11256593 | 115.2953852 |
| CD | rs28732150 | 94.64364232 |
| CD | rs2071463 | 86.33740546 |
| CD | rs11749040 | 71.01785716 |
| CD | rs438613 | 69.08375957 |
| CD | rs1026916 | 55.32806096 |
| CD | rs6032662 | 53.31217807 |
| CD | rs1077667 | 51.19208668 |
| CD | rs2546890 | 50.76912939 |
| CD | rs35540610 | 48.76956315 |
| CD | rs631204 | 47.18824872 |
| CD | rs17124032 | 47.00466038 |
| CD | rs1860545 | 46.81876026 |
| CD | rs1738074 | 46.34664316 |
| CD | rs701006 | 45.74151198 |
| CD | rs13327021 | 45.25801287 |
| CD | rs1250551 | 44.41118521 |
| CD | rs59655222 | 43.73604334 |
| CD | rs72928038 | 42.02554183 |
| CD | rs1014486 | 41.21439181 |
| CD | rs7592560 | 39.75841812 |
| CD | rs12365699 | 39.58166659 |
| CD | rs9843355 | 38.78762253 |
| CD | rs10063294 | 37.0867685 |
| CD | rs35703946 | 36.03508161 |
| CD | rs3130171 | 34.77546197 |
| CD | rs354033 | 32.47876532 |
| CD | rs7855251 | 30.03961833 |
| CD | rs2232423 | 78.44897959 |
| CD | rs2568958 | 75.37396694 |
| CD | rs13037326 | 40.02498959 |
| CD | rs4141983 | 32.93761815 |
| CD | rs17641524 | 32.03987184 |
| CD | rs9831648 | 31.53254438 |
| CD | rs2671427 | 38.71455701 |
| CD | rs2666957 | 35.19131503 |

| | | |
|---|---|---|
| CD | rs7153386 | 29.98211979 |
| CD | rs7553711 | 48.40858921 |
| CD | rs34593439 | 36.4330083 |
| CD | rs10995245 | 29.79195949 |
| CD | rs962993 | 42.02303291 |
| CD | rs17027258 | 39.09494618 |
| CD | rs34540843 | 30.8216409 |
| CD | rs3774354 | 46.03364845 |
| CD | rs56116847 | 32.06390625 |
| CD | rs10758594 | 32.0620678 |
| CD | rs7774434 | 250.1841762 |
| CD | rs6679356 | 119.6655099 |
| CD | rs10488631 | 97.61563466 |
| CD | rs10947260 | 67.70253747 |
| CD | rs2293370 | 61.57651862 |
| CD | rs12924729 | 58.18593463 |
| CD | rs4938573 | 55.0940682 |
| CD | rs243325 | 48.73339793 |
| CD | rs17641524 | 46.30242557 |
| CD | rs2069235 | 44.75387889 |
| CD | rs9303277 | 44.48022503 |
| CD | rs860413 | 43.28748258 |
| CD | rs2304256 | 41.72722106 |
| CD | rs7665090 | 37.70934918 |
| CD | rs1800693 | 36.1412363 |
| CD | rs911263 | 35.74633426 |
| CD | rs11065987 | 30.58355034 |
| CD | rs7775055 | 220.8673499 |
| CD | rs72678531 | 167.6055727 |
| CD | rs522127 | 94.11121064 |
| CD | rs35188261 | 92.56254669 |
| CD | rs9295947 | 65.38650962 |
| CD | rs2293370 | 65.18043975 |
| CD | rs17122453 | 64.94228033 |
| CD | rs1646019 | 60.6790643 |
| CD | rs1800693 | 59.57663455 |
| CD | rs10931468 | 56.99870129 |
| CD | rs12708715 | 56.80966557 |
| CD | rs8067378 | 56.35437544 |
| CD | rs7665090 | 55.69208254 |
| CD | rs2267407 | 54.86217665 |
| CD | rs6871748 | 53.76214643 |
| CD | rs12134279 | 48.13705887 |
| CD | rs911263 | 41.83114872 |
| CD | rs1014486 | 41.68009932 |
| CD | rs3998159 | 38.752453 |
| CD | rs17564829 | 35.83475138 |
| CD | rs11065979 | 35.26890051 |
| CD | rs34725611 | 34.50840924 |
| CD | rs11064157 | 34.07174106 |
| CD | rs11117431 | 32.86547623 |

| | | |
|---|---|---|
| CD | rs2523647 | 30.51916156 |
| CD | rs10847864 | 50.65622172 |
| CD | rs620490 | 38.17939058 |
| CD | rs4488803 | 32.58745991 |
| CD | rs6983267 | 643.4891844 |
| CD | rs10840603 | 302.6889838 |
| CD | rs651164 | 157.9591942 |
| CD | rs1058319 | 103.4157778 |
| CD | rs17749618 | 51.33301038 |
| CD | rs2305498 | 46.5124 |
| CD | rs2523733 | 114.6894657 |
| CD | rs2071463 | 68.61710623 |
| CD | rs9263875 | 55.15399286 |
| CD | rs74817271 | 43.29014502 |
| CD | rs12713428 | 33.00060592 |
| CD | rs13201757 | 1019.640594 |
| CD | rs9391734 | 397.5892951 |
| CD | rs28780084 | 205.7241026 |
| CD | rs2199874 | 103.7710131 |
| CD | rs7741091 | 87.83161454 |
| CD | rs926070 | 66.26200654 |
| CD | rs4845454 | 63.07969313 |
| CD | rs6457109 | 62.21317999 |
| CD | rs17728338 | 60.48454624 |
| CD | rs6833586 | 55.95279491 |
| CD | rs2546890 | 50.60564096 |
| CD | rs141417497 | 47.92730231 |
| CD | rs72669160 | 46.61059326 |
| CD | rs1416173 | 45.30658757 |
| CD | rs114835245 | 44.79300763 |
| CD | rs78580783 | 42.2007555 |
| CD | rs9461362 | 42.00074802 |
| CD | rs68191 | 39.83862541 |
| CD | rs77840275 | 39.65505709 |
| CD | rs892085 | 39.02536227 |
| CD | rs30376 | 38.63362758 |
| CD | rs9399137 | 37.54827919 |
| CD | rs3868078 | 34.72041834 |
| CD | rs80174646 | 34.37441395 |
| CD | rs9262155 | 33.9142097 |
| CD | rs2074478 | 32.98833642 |
| CD | rs62421044 | 31.808472 |
| CD | rs1990760 | 31.54190303 |
| CD | rs11652075 | 31.27935367 |
| CD | rs12447534 | 31.0861904 |
| CD | rs117950837 | 30.31483062 |
| CD | rs11564177 | 30.03806355 |
| CD | rs2476601 | 317.0948351 |
| CD | rs8283 | 226.3014613 |
| CD | rs2074488 | 178.4507845 |
| CD | rs4711211 | 144.0976519 |

| | | |
|---|---|---|
| CD | rs106287 | 115.8059851 |
| CD | rs6922431 | 76.20597332 |
| CD | rs456261 | 61.10810475 |
| CD | rs2532919 | 53.34238971 |
| CD | rs6901724 | 51.3848525 |
| CD | rs6920220 | 49.05173894 |
| CD | rs10040327 | 42.61910456 |
| CD | rs3909134 | 40.93414998 |
| CD | rs4810485 | 33.93781964 |
| CD | rs3087243 | 31.27446019 |
| CD | rs11757182 | 30.80494096 |
| CD | rs2267632 | 30.76140683 |
| CD | rs2476601 | 869.340315 |
| CD | rs3130379 | 272.8979258 |
| CD | rs1571878 | 165.34496 |
| CD | rs3893538 | 140.3479735 |
| CD | rs6456964 | 133.7007259 |
| CD | rs8026898 | 129.8023912 |
| CD | rs7752903 | 126.8912923 |
| CD | rs34695944 | 100.9924468 |
| CD | rs4239702 | 100.9924468 |
| CD | rs29243 | 76.07991245 |
| CD | rs9348832 | 68.80725792 |
| CD | rs1858036 | 66.93076603 |
| CD | rs3087243 | 64.82443647 |
| CD | rs17404424 | 61.55848945 |
| CD | rs11933540 | 60.50338484 |
| CD | rs2561477 | 57.74592653 |
| CD | rs2301888 | 55.86258863 |
| CD | rs1893592 | 55.73265308 |
| CD | rs6712515 | 55.73265308 |
| CD | rs11889341 | 55.45614566 |
| CD | rs3806624 | 55.28948982 |
| CD | rs8032939 | 44.37181597 |
| CD | rs3128935 | 43.43043586 |
| CD | rs11574914 | 40.06789519 |
| CD | rs62097857 | 38.20344257 |
| CD | rs4409785 | 37.10507058 |
| CD | rs1953126 | 36.62845648 |
| CD | rs947474 | 33.38311413 |
| CD | rs73081554 | 31.68823116 |
| CD | rs9268145 | 1121.704453 |
| CD | rs2476601 | 275.0492786 |
| CD | rs10484565 | 159.9238633 |
| CD | rs1029237 | 143.5033085 |
| CD | rs1431403 | 138.0943099 |
| CD | rs544167 | 132.6617645 |
| CD | rs2524084 | 117.9188209 |
| CD | rs13196329 | 106.592422 |
| CD | rs6922431 | 91.49959803 |
| CD | rs2267644 | 89.36491131 |

| | | |
|---|---|---|
| CD | rs620202 | 85.36086947 |
| CD | rs71624119 | 83.75814367 |
| CD | rs2296330 | 69.52736813 |
| CD | rs2531804 | 54.63181621 |
| CD | rs361525 | 53.6871644 |
| CD | rs462618 | 53.19226861 |
| CD | rs6920220 | 40.63258014 |
| CD | rs17630466 | 40.07416584 |
| CD | rs13426947 | 37.96899322 |
| CD | rs194679 | 37.77708423 |
| CD | rs8026898 | 35.76860251 |
| CD | rs10209110 | 32.6104239 |
| CD | rs2812378 | 32.32107356 |
| CD | rs2228145 | 32.30745226 |
| CD | rs2240339 | 32.24777667 |
| CD | rs2476601 | 678.2551357 |
| CD | rs3749946 | 98.08874685 |
| CD | rs3087243 | 82.77339454 |
| CD | rs17264332 | 78.73552445 |
| CD | rs68191 | 73.15273165 |
| CD | rs8026898 | 71.78450094 |
| CD | rs34046593 | 69.13361899 |
| CD | rs17404424 | 66.33736053 |
| CD | rs1571878 | 61.30077778 |
| CD | rs4239702 | 57.07239393 |
| CD | rs34695944 | 56.98157996 |
| CD | rs11574914 | 54.57048351 |
| CD | rs12764378 | 54.10525646 |
| CD | rs13426947 | 49.1263451 |
| CD | rs8032939 | 49.12630299 |
| CD | rs9653442 | 48.33154658 |
| CD | rs12539741 | 45.97130691 |
| CD | rs2069235 | 39.67457119 |
| CD | rs2561477 | 38.60115167 |
| CD | rs2451258 | 38.13561104 |
| CD | rs9368696 | 37.32477138 |
| CD | rs2240336 | 36.66918076 |
| CD | rs10796038 | 35.97381764 |
| CD | rs12232497 | 34.82901639 |
| CD | rs592390 | 34.7234128 |
| CD | rs61828284 | 33.11201994 |
| CD | rs1893592 | 32.8805961 |
| CD | rs10985070 | 31.81033859 |
| CD | rs9310852 | 30.58230767 |
| CD | rs624988 | 29.87842993 |
| CD | rs4936059 | 29.87840789 |
| CD | rs73081554 | 29.83663305 |
| CD | rs9268145 | 1581.710785 |
| CD | rs2476601 | 302.4266715 |
| CD | rs8283 | 224.880682 |
| CD | rs2074488 | 168.2536436 |

| | | |
|---|---|---|
| CD | rs4711211 | 143.8448016 |
| CD | rs106287 | 119.7872959 |
| CD | rs6922431 | 74.83083318 |
| CD | rs456261 | 57.33781905 |
| CD | rs2532919 | 52.74731825 |
| CD | rs6901724 | 49.46524787 |
| CD | rs6920220 | 46.04012992 |
| CD | rs10040327 | 42.6826205 |
| CD | rs3909134 | 40.09318172 |
| CD | rs3087243 | 35.19521763 |
| CD | rs2267632 | 30.81277589 |
| CD | rs11757182 | 30.51698516 |
| CD | rs4810485 | 29.25598476 |
| CD | rs2476601 | 678.1835787 |
| CD | rs8192585 | 231.0436375 |
| CD | rs3087243 | 82.81707269 |
| CD | rs17264332 | 78.80610491 |
| CD | rs68191 | 73.16315931 |
| CD | rs8026898 | 71.82587412 |
| CD | rs11933540 | 69.06149303 |
| CD | rs7738250 | 68.84871266 |
| CD | rs17404424 | 66.29695683 |
| CD | rs1571878 | 61.29400579 |
| CD | rs4239702 | 57.07597389 |
| CD | rs34695944 | 56.92861801 |
| CD | rs11574914 | 54.61975304 |
| CD | rs12764378 | 54.04717811 |
| CD | rs13426947 | 49.11796565 |
| CD | rs8032939 | 49.11329476 |
| CD | rs9653442 | 48.37975309 |
| CD | rs2069235 | 39.62299901 |
| CD | rs2561477 | 38.57806634 |
| CD | rs2451258 | 38.19013214 |
| CD | rs9368696 | 37.29145521 |
| CD | rs2240336 | 36.59887734 |
| CD | rs10796038 | 35.95196798 |
| CD | rs592390 | 34.74349906 |
| CD | rs61828284 | 33.11008605 |
| CD | rs9469591 | 32.9190277 |
| CD | rs1893592 | 32.88498791 |
| CD | rs10985070 | 31.79931288 |
| CD | rs9310852 | 30.57771989 |
| CD | rs624988 | 29.90069577 |
| CD | rs4936059 | 29.85748686 |
| CD | rs73081554 | 29.83558431 |
| CD | rs3957146 | 389.9863469 |
| CD | rs2071463 | 46.07410256 |
| CD | rs2476601 | 41.76513956 |
| CD | rs1077394 | 41.62166062 |
| CD | rs1419675 | 34.68512776 |
| CD | rs4274624 | 293.2513845 |

| | | |
|---|---|---|
| CD | rs1143679 | 212.0016865 |
| CD | rs35000415 | 200.2294151 |
| CD | rs6889239 | 76.51123259 |
| CD | rs58721818 | 75.6557835 |
| CD | rs2299851 | 61.01868032 |
| CD | rs2431697 | 58.01521611 |
| CD | rs6679677 | 52.39196829 |
| CD | rs7768653 | 48.6189269 |
| CD | rs353608 | 44.22170731 |
| CD | rs58688157 | 44.19823106 |
| CD | rs4916215 | 43.15163271 |
| CD | rs7097397 | 42.11576829 |
| CD | rs78517564 | 40.56838264 |
| CD | rs2459611 | 33.36980543 |
| CD | rs73068668 | 29.96640854 |
| CD | rs2187668 | 91.39273059 |
| CD | rs11229 | 72.57258846 |
| CD | rs7574865 | 57.2268362 |
| CD | rs10488631 | 53.6121581 |
| CD | rs3132631 | 52.23451543 |
| CD | rs16891725 | 50.9436309 |
| CD | rs853676 | 40.64794627 |
| CD | rs3749971 | 38.3082367 |
| CD | rs1024582 | 73.96598273 |
| CD | rs1615350 | 47.86839844 |
| CD | rs11682175 | 45.46470849 |
| CD | rs2414718 | 40.26133231 |
| CD | rs11874716 | 37.32210249 |
| CD | rs11210892 | 34.75563863 |
| CD | rs3849046 | 32.87391647 |
| CD | rs10108725 | 30.29467814 |
| CD | rs9268145 | 983.2118523 |
| CD | rs485502 | 413.8945438 |
| CD | rs2476601 | 255.0132983 |
| CD | rs2071463 | 222.2042068 |
| CD | rs1419675 | 109.3444597 |
| CD | rs3184504 | 65.09399728 |
| CD | rs11066320 | 50.67509336 |
| CD | rs705702 | 45.1584 |
| CD | rs11571297 | 32.42802122 |
| CD | rs3957146 | 721.0693728 |
| CD | rs2071463 | 80.77267157 |
| CD | rs2476601 | 75.94293215 |
| CD | rs2256539 | 59.24542041 |
| CD | rs6905949 | 52.83961648 |
| CD | rs3842727 | 892.1879561 |
| CD | rs6679677 | 554.8636935 |
| CD | rs3184504 | 196.000884 |
| CD | rs61839660 | 171.9010403 |
| CD | rs1701704 | 137.7539958 |
| CD | rs11066320 | 120.9991893 |

| | | |
|---|---|---|
| CD | rs12927355 | 94.09039781 |
| CD | rs3087243 | 87.76771775 |
| CD | rs8056814 | 80.41995498 |
| CD | rs2111485 | 75.41625824 |
| CD | rs1893217 | 64 |
| CD | rs11203203 | 62.32704479 |
| CD | rs12416116 | 61.73474733 |
| CD | rs516246 | 56.64517909 |
| CD | rs72928038 | 56.24953121 |
| CD | rs34593439 | 55.57010009 |
| CD | rs4820830 | 50.48474486 |
| CD | rs6827756 | 47.53720135 |
| CD | rs56994090 | 46.09686261 |
| CD | rs7239671 | 45.18840856 |
| CD | rs2611215 | 45.15835268 |
| CD | rs2304256 | 43.81151828 |
| CD | rs151233 | 43.25597164 |
| CD | rs6043409 | 39.6899937 |
| CD | rs72727394 | 39.34692084 |
| CD | rs2045258 | 37.91982455 |
| CD | rs1574285 | 36.63460047 |
| CD | rs41295121 | 33.99306255 |
| CD | rs7893467 | 33.23180199 |
| CD | rs12150079 | 32.49014245 |
| CD | rs229533 | 31.71466584 |
| CD | rs60918750 | 31.59219765 |
| CD | rs62447205 | 31.0406647 |
| CD | rs9585056 | 30.5125418 |
| CD | rs4849135 | 29.98855034 |
| CD | rs113010081 | 29.88453674 |
| CD | rs402072 | 29.82858008 |
| CD | rs2596560 | 687.9581449 |
| CD | rs6679677 | 355.8566023 |
| CD | rs3129809 | 273.9222027 |
| CD | rs6913635 | 214.3114623 |
| CD | rs9275601 | 199.7820048 |
| CD | rs9276943 | 189.7241355 |
| CD | rs9267536 | 117.9153838 |
| CD | rs9467704 | 117.0559971 |
| CD | rs11571297 | 68.67304029 |
| CD | rs741172 | 62.15305571 |
| CD | rs2394392 | 61.12668492 |
| CD | rs12722495 | 59.41840278 |
| CD | rs3094138 | 41.00227505 |
| CD | rs8056814 | 40.49865583 |
| CD | rs2111485 | 40.43523998 |
| CD | rs202520 | 37.75526428 |
| CD | rs194749 | 33.9793569 |
| CD | rs2596560 | 1498.833347 |
| CD | rs1894406 | 838.3230436 |
| CD | rs387608 | 465.5808626 |

| | | |
|---|---|---|
| CD | rs2535319 | 391.6576889 |
| CD | rs2071463 | 366.3098416 |
| CD | rs2476601 | 331.0880737 |
| CD | rs149943 | 288.6743954 |
| CD | rs3871466 | 150.5002563 |
| CD | rs4759229 | 104.2639664 |
| CD | rs12722496 | 103.4254097 |
| CD | rs12927355 | 72.04860879 |
| CD | rs80054410 | 53.56278678 |
| CD | rs2111485 | 45.43818071 |
| CD | rs12982646 | 43.70844043 |
| CD | rs151233 | 43.61333085 |
| CD | rs7122407 | 43.17035171 |
| CD | rs13415465 | 39.54962553 |
| CD | rs4238595 | 36.37755316 |
| CD | rs34725611 | 35.43176379 |
| CD | rs2281808 | 30.63143581 |
| CD | rs9911533 | 30.57604133 |
| CD | rs3024505 | 30.00770331 |
| CD | rs9268145 | 949.1737935 |
| CD | rs485502 | 392.5124358 |
| CD | rs2476601 | 233.2373085 |
| CD | rs2071463 | 197.2503956 |
| CD | rs1419675 | 99.79392071 |
| CD | rs3184504 | 60.28203987 |
| CD | rs11066320 | 44.06965108 |
| CD | rs705699 | 42.94820984 |
| CD | rs9277932 | 40.11111111 |
| CD | rs34337125 | 30.32751438 |
| CD | rs10484565 | 461.6877968 |
| CD | rs34171662 | 190.7876263 |
| CD | rs2476601 | 190.1168939 |
| CD | rs3135333 | 131.6059263 |
| CD | rs2256539 | 107.9631666 |
| CD | rs3025643 | 86.4151886 |
| CD | rs9268145 | 978.2427824 |
| CD | rs485502 | 404.8277578 |
| CD | rs2476601 | 241.5913733 |
| CD | rs2071463 | 205.091692 |
| CD | rs2256539 | 122.6913534 |
| CD | rs3184504 | 57.63071073 |
| CD | rs11066320 | 44.08734918 |
| CD | rs11571297 | 36.55441957 |
| CD | rs41176 | 33.42431445 |
| CD | rs2237897 | 97.08044983 |
| CD | rs11558471 | 53.82105098 |
| CD | rs10938397 | 48.61892562 |
| CD | rs80134320 | 32.86787202 |
| CD | rs5215 | 32.59371901 |
| CD | rs10974438 | 32.47991229 |
| CD | rs28624681 | 54.80590109 |

| | | |
|---|---|---|
| CD | rs10938397 | 50.32507853 |
| CD | rs11558471 | 49.11577078 |
| CD | rs76177300 | 42.17234647 |
| CD | rs498475 | 32.4526337 |
| CD | rs1053924 | 31.09893974 |
| CD | rs2383208 | 199.2238757 |
| CD | rs13266634 | 92.73731617 |
| CD | rs11708067 | 45.64088074 |
| CD | rs864745 | 45.64088074 |
| CD | rs5215 | 36.62845648 |
| CD | rs7756992 | 276.4972058 |
| CD | rs10811661 | 256.3266347 |
| CD | rs849135 | 192.515625 |
| CD | rs11708067 | 125.9092753 |
| CD | rs17334919 | 119.2873535 |
| CD | rs780094 | 87.4477721 |
| CD | rs5215 | 86.26083693 |
| CD | rs7729395 | 73.63785156 |
| CD | rs340874 | 73.5364984 |
| CD | rs17168486 | 62.30918968 |
| CD | rs10974438 | 62.0944 |
| CD | rs6795735 | 58.42822293 |
| CD | rs2857605 | 55.75111111 |
| CD | rs12970134 | 48.12890625 |
| CD | rs10401969 | 47.95302165 |
| CD | rs853974 | 46.64269112 |
| CD | rs2071479 | 42.48040175 |
| CD | rs67232546 | 38.54340278 |
| CD | rs2237892 | 37.38894073 |
| CD | rs1265099 | 37.30898466 |
| CD | rs9940149 | 37.27423823 |
| CD | rs940904 | 36.14472347 |
| CD | rs17405722 | 35.50853819 |
| CD | rs16988333 | 32.84171598 |
| CD | rs7561798 | 30.86419753 |
| CD | rs7756992 | 164.8529188 |
| CD | rs13266634 | 107.9001563 |
| CD | rs864745 | 72.94083272 |
| CD | rs11708067 | 72.90532544 |
| CD | rs5215 | 65.69529086 |
| CD | rs2943641 | 58.51256536 |
| CD | rs60980157 | 55.99747507 |
| CD | rs1260326 | 54.60634171 |
| CD | rs2191349 | 50.52520088 |
| CD | rs7202877 | 46.97263534 |
| CD | rs340874 | 44.44444444 |
| CD | rs1077394 | 43.32639 |
| CD | rs35658696 | 40.86371257 |
| CD | rs35720761 | 37.52849375 |
| CD | rs4607517 | 33.4084 |
| CD | rs10146997 | 32.87111111 |

| | | |
|---|---|---|
| CD | rs17782313 | 32.15405475 |
| CD | rs6795735 | 30.39490997 |
| CD | rs10758593 | 29.73884444 |
| CD | rs1727307 | 24.51457466 |
| CD | rs2237897 | 86.14160156 |
| CD | rs3129871 | 55.65424074 |
| CD | rs11558471 | 49.52476199 |
| CD | rs10938397 | 43.0033284 |
| CD | rs76177300 | 40.2824649 |
| CD | rs2256594 | 38.14080425 |
| CD | rs498475 | 33.48979767 |
| CD | rs5215 | 31.74889053 |
| CD | rs10974438 | 29.78915189 |
| CD | rs2237897 | 96.74337269 |
| CD | rs11558471 | 54.04236669 |
| CD | rs10938397 | 47.07484568 |
| CD | rs76177300 | 35.02394842 |
| CD | rs5215 | 32.53223594 |
| CD | rs7901695 | 952.6896859 |
| CD | rs7756992 | 248.6686391 |
| CD | rs13266634 | 176.733564 |
| CD | rs864745 | 136.232666 |
| CD | rs7501939 | 95.85041623 |
| CD | rs1260326 | 93.44444444 |
| CD | rs11708067 | 91.25277008 |
| CD | rs2943641 | 77.02027178 |
| CD | rs2191349 | 66.15111111 |
| CD | rs10758593 | 61.6611126 |
| CD | rs60980157 | 60.0625 |
| CD | rs35658696 | 56.79347511 |
| CD | rs340874 | 55.39263639 |
| CD | rs35720761 | 52.67022841 |
| CD | rs2395163 | 46.63452216 |
| CD | rs7202877 | 43.53278776 |
| CD | rs17782313 | 39.87020408 |
| CD | rs10146997 | 36.30834977 |
| CD | rs730497 | 34.5337601 |
| CD | rs1727307 | 34.17751479 |
| CD | rs4607103 | 31.58123388 |
| CD | rs9388489 | 26.88023669 |
| CD | rs1065461 | 26.69444444 |
| CD | rs665268 | 26.01 |
| CD | rs1800961 | 23.73854938 |
| CD | rs2237897 | 97.23333581 |
| CD | rs11558471 | 52.82174745 |
| CD | rs10938397 | 47.20198903 |
| CD | rs76177300 | 35.69032402 |
| CD | rs5215 | 32.84987997 |
| CD | rs7901695 | 979.3217993 |
| CD | rs7756992 | 256.4778347 |
| CD | rs4402960 | 193.8845271 |

| | | |
|---|---|---|
| CD | rs13266634 | 174.0071367 |
| CD | rs864745 | 116.307929 |
| CD | rs11708067 | 115.7003616 |
| CD | rs7501939 | 94.45410156 |
| CD | rs2943641 | 76.37240076 |
| CD | rs1260326 | 69.78674556 |
| CD | rs60980157 | 60.60326871 |
| CD | rs340874 | 59.51020408 |
| CD | rs7202877 | 53.77777778 |
| CD | rs35658696 | 48.76029388 |
| CD | rs35720761 | 48.06222263 |
| CD | rs10758593 | 40.17924037 |
| CD | rs2395163 | 39.1426693 |
| CD | rs6905288 | 38.05940828 |
| CD | rs4607103 | 36.17412308 |
| CD | rs1727307 | 32.84829583 |
| CD | rs7961581 | 31.29510607 |
| CD | rs1065461 | 27.49159971 |
| CD | rs9388489 | 26.51481399 |
| CD | rs1800961 | 25.43430241 |
| CD | rs730497 | 24.17361111 |
| CD | rs1421085 | 72.49375232 |
| CD | rs13266634 | 64.67040509 |
| CD | rs2383208 | 51.83339471 |
| CD | rs11708067 | 46.60171771 |
| CD | rs115505614 | 41.62012014 |
| CD | rs72802342 | 37.12698404 |
| CD | rs2014830 | 31.99926034 |
| CD | rs12912777 | 31.52083868 |
| CD | rs515071 | 30.72245884 |
| CD | rs1635852 | 30.60674781 |
| CD | rs179252 | 124.0609175 |
| CD | rs2476601 | 93.1268274 |
| CD | rs3094067 | 84.34972299 |
| CD | rs11571297 | 52.87566475 |
| CD | rs2855426 | 44.11338828 |
| CD | rs7516666 | 443.202828 |
| CD | rs9869760 | 373.559354 |
| CD | rs2836836 | 368.6366667 |
| CD | rs12428112 | 72.89417023 |
| CD | rs12421579 | 53.9085254 |
| CD | rs12151689 | 50.28327229 |
| CD | rs6516819 | 46.83519595 |
| CD | rs2001823 | 46.74806385 |
| CD | rs965513 | 46.40110627 |
| CD | rs6060124 | 44.50478237 |
| CD | rs4149570 | 44.15782176 |
| CD | rs869987 | 43.57851929 |
| CD | rs3809983 | 41.5960671 |
| CD | rs10801825 | 41.44580956 |
| CD | rs932311 | 40.65684167 |

| | | |
|---|---|---|
| CD | rs9290550 | 39.40310821 |
| CD | rs241449 | 39.16755826 |
| CD | rs2526401 | 38.62141278 |
| CD | rs1868534 | 38.11722773 |
| CD | rs12782349 | 37.66344954 |
| CD | rs3828193 | 36.26520651 |
| CD | rs2516677 | 34.65804705 |
| CD | rs8049383 | 33.47773309 |
| CD | rs9355255 | 32.72510609 |
| CD | rs950910 | 30.34197834 |
| CD | rs406113 | 30.18413893 |
| UC | rs2261033 | 1309.70595 |
| UC | rs34521355 | 619.4259078 |
| UC | rs3891175 | 340.6548467 |
| UC | rs17312661 | 329.0355684 |
| UC | rs2074475 | 251.7351789 |
| UC | rs27529 | 208.270196 |
| UC | rs4672505 | 207.3744313 |
| UC | rs9380120 | 202.8458428 |
| UC | rs7453920 | 187.3840192 |
| UC | rs2523443 | 163.8322774 |
| UC | rs7746199 | 153.3312682 |
| UC | rs17194627 | 146.3295001 |
| UC | rs3857546 | 143.5570397 |
| UC | rs7771335 | 111.647554 |
| UC | rs362521 | 104.9229818 |
| UC | rs9262222 | 90.92558971 |
| UC | rs2836883 | 69.83216632 |
| UC | rs9393646 | 68.78743718 |
| UC | rs41299637 | 63.25861617 |
| UC | rs6600247 | 62.56710603 |
| UC | rs9901869 | 60.88986422 |
| UC | rs2239842 | 59.29114202 |
| UC | rs11190133 | 56.79210306 |
| UC | rs4129267 | 53.00824023 |
| UC | rs11624293 | 41.03960526 |
| UC | rs1041926 | 40.96172978 |
| UC | rs1860545 | 39.82087767 |
| UC | rs35164067 | 39.41475927 |
| UC | rs2927613 | 38.00117864 |
| UC | rs12615545 | 37.26600201 |
| UC | rs1801274 | 36.74091877 |
| UC | rs1250550 | 36.5824539 |
| UC | rs7191548 | 34.00021582 |
| UC | rs7282490 | 33.76681929 |
| UC | rs1128905 | 33.54905971 |
| UC | rs6915101 | 33.53582202 |
| UC | rs4676410 | 32.86114726 |
| UC | rs11065898 | 29.83296349 |
| UC | rs6881706 | 122.0334473 |
| UC | rs56375023 | 115.5625 |

| | | |
|---|---|---|
| UC | rs7224129 | 88.09633733 |
| UC | rs848 | 67.78025896 |
| UC | rs12365699 | 57.60420776 |
| UC | rs2228145 | 41.35820452 |
| UC | rs6011033 | 40.11111111 |
| UC | rs249677 | 38.48204539 |
| UC | rs45613035 | 37.73469388 |
| UC | rs4845604 | 34.16680839 |
| UC | rs17664743 | 33.34655822 |
| UC | rs12740374 | 74.62841521 |
| UC | rs10774625 | 55.96924896 |
| UC | rs11591147 | 42.23149781 |
| UC | rs943689 | 15331.36608 |
| UC | rs174535 | 14925.55714 |
| UC | rs11742240 | 13095.58337 |
| UC | rs6866614 | 12003.57035 |
| UC | rs4771332 | 10973.31764 |
| UC | rs72743461 | 8550.412667 |
| UC | rs17454584 | 8543.773292 |
| UC | rs17668708 | 6537.486118 |
| UC | rs57585717 | 5200.061381 |
| UC | rs62626322 | 4884.227913 |
| UC | rs4795399 | 12651.68545 |
| UC | rs11065979 | 8666.64348 |
| UC | rs7100984 | 7384.392732 |
| UC | rs9391997 | 6447.57544 |
| UC | rs912425 | 6359.282939 |
| UC | rs10786050 | 6096.274685 |
| UC | rs449454 | 6010.474415 |
| UC | rs705700 | 5925.1174 |
| UC | rs10995245 | 5865.850642 |
| UC | rs35570272 | 5785.519507 |
| UC | rs3997849 | 5673.511839 |
| UC | rs12365699 | 5421.749137 |
| UC | rs4917131 | 5333.142509 |
| UC | rs11236797 | 5009.390268 |
| UC | rs4845604 | 4729.476999 |
| UC | rs55743914 | 4442.172618 |
| UC | rs2738783 | 4177.728664 |
| UC | rs72743461 | 3725.129751 |
| UC | rs9275602 | 3457.002739 |
| UC | rs17611877 | 2190.374927 |
| UC | rs4705869 | 2030.281908 |
| UC | rs45613035 | 1816.028893 |
| UC | rs10118244 | 1220.032642 |
| UC | rs4065275 | 60.0625 |
| UC | rs4851582 | 37.83680368 |
| UC | rs17228058 | 31.19476369 |
| UC | rs56375023 | 180.8381491 |
| UC | rs848 | 129.3111611 |
| UC | rs943689 | 105.8320661 |

| | | |
|---|---|---|
| UC | rs35570272 | 57.79741471 |
| UC | rs17454584 | 57.42021622 |
| UC | rs2229094 | 46.45601899 |
| UC | rs2296618 | 41.30204088 |
| UC | rs11742240 | 37.99541679 |
| UC | rs6922431 | 37.29855251 |
| UC | rs12365699 | 37.04297066 |
| UC | rs3130196 | 36.07438813 |
| UC | rs1800797 | 35.8317146 |
| UC | rs2255088 | 35.22255884 |
| UC | rs11042902 | 33.78044012 |
| UC | rs7219483 | 33.40236018 |
| UC | rs2275806 | 30.53114472 |
| UC | rs10912564 | 30.48300256 |
| UC | rs4787426 | 30.05105399 |
| UC | rs11679146 | 75.00572944 |
| UC | rs12949100 | 70.13310017 |
| UC | rs10900809 | 45.65419851 |
| UC | rs72743461 | 40.34409125 |
| UC | rs34972832 | 34.07344531 |
| UC | rs3781094 | 32.19627276 |
| UC | rs76830965 | 751.4926184 |
| UC | rs2523554 | 95.59110318 |
| UC | rs17552787 | 73.16962599 |
| UC | rs17282175 | 62.12034288 |
| UC | rs913678 | 56.484542 |
| UC | rs9261425 | 48.62943421 |
| UC | rs436857 | 42.88770149 |
| UC | rs10513548 | 32.5228789 |
| UC | rs2305479 | 187.7155729 |
| UC | rs3771180 | 86.39709541 |
| UC | rs17293632 | 64.6798294 |
| UC | rs2155219 | 62.33605557 |
| UC | rs20541 | 59.28971257 |
| UC | rs2596464 | 54.22461405 |
| UC | rs2325291 | 51.14465884 |
| UC | rs2855812 | 39.80707175 |
| UC | rs7705042 | 37.63508136 |
| UC | rs2523716 | 32.21965276 |
| UC | rs241429 | 30.72053745 |
| UC | rs12740374 | 137.8754864 |
| UC | rs10774625 | 99.02658713 |
| UC | rs11591147 | 67.90742618 |
| UC | rs2681472 | 50.46833153 |
| UC | rs72743461 | 43.38995462 |
| UC | rs17115100 | 37.29062542 |
| UC | rs13402621 | 37.12083809 |
| UC | rs748431 | 30.2782117 |
| UC | rs2160203 | 101.5586578 |
| UC | rs20541 | 52.13554725 |
| UC | rs879606 | 36.57155192 |

| | | |
|---|---|---|
| UC | rs10806425 | 34.9088659 |
| UC | rs62322662 | 33.49912916 |
| UC | rs6010620 | 63.17508918 |
| UC | rs17513531 | 34.8043543 |
| UC | rs61839660 | 33.13625904 |
| UC | rs2476601 | 695.271424 |
| UC | rs3184504 | 255.6445679 |
| UC | rs11066320 | 212.835679 |
| UC | rs2596503 | 156.4693752 |
| UC | rs11571297 | 145.3073015 |
| UC | rs9277557 | 128.376993 |
| UC | rs28780083 | 77.82161548 |
| UC | rs10905669 | 66.97251145 |
| UC | rs1990760 | 56.08345679 |
| UC | rs229541 | 54.76 |
| UC | rs13203715 | 50.30862245 |
| UC | rs4073285 | 39.33471172 |
| UC | rs75438091 | 37.34567901 |
| UC | rs2293158 | 33.0625 |
| UC | rs7205474 | 30.53515161 |
| UC | rs9392026 | 100.500894 |
| UC | rs3778607 | 81.22837799 |
| UC | rs3087243 | 76.72820712 |
| UC | rs72928038 | 71.13282576 |
| UC | rs162298 | 54.06345131 |
| UC | rs2049218 | 46.42533891 |
| UC | rs2476601 | 39.76934144 |
| UC | rs4682103 | 32.62425174 |
| UC | rs10994397 | 38.50900594 |
| UC | rs7296288 | 32.95192315 |
| UC | rs12576775 | 30.86928367 |
| UC | rs7258465 | 120.6668962 |
| UC | rs7017073 | 58.16604444 |
| UC | rs9952980 | 54.07905325 |
| UC | rs4971059 | 43.890625 |
| UC | rs3749971 | 33.78515625 |
| UC | rs61744064 | 32.92125773 |
| UC | rs17426269 | 32.06719849 |
| UC | rs666930 | 30.33736458 |
| UC | rs414851 | 10679.99029 |
| UC | rs17076598 | 8944.242115 |
| UC | rs3759333 | 7956.728479 |
| UC | rs26433 | 7862.218435 |
| UC | rs3800393 | 6135.983387 |
| UC | rs12606193 | 4627.748927 |
| UC | rs11864719 | 4513.373015 |
| UC | rs17450969 | 4142.522261 |
| UC | rs592719 | 3790.1959 |
| UC | rs6880335 | 2343.940734 |
| UC | rs2109897 | 2066.055573 |
| UC | rs12650313 | 1930.49442 |

| | | |
|---|---|---|
| UC | rs10519288 | 1613.473299 |
| UC | rs16839999 | 578.9153819 |
| UC | rs17238590 | 527.3553142 |
| UC | rs16941631 | 341.6692449 |
| UC | rs72905734 | 32.99332008 |
| UC | rs4468572 | 65.71142838 |
| UC | rs2681472 | 42.76643653 |
| UC | rs11065979 | 40.54015268 |
| UC | rs11191416 | 34.33208586 |
| UC | rs515135 | 30.64899745 |
| UC | rs599839 | 39.75093937 |
| UC | rs1122608 | 37.37777487 |
| UC | rs2306374 | 30.49755064 |
| UC | rs11591147 | 45.84997549 |
| UC | rs653178 | 45.11184822 |
| UC | rs12705390 | 42.67808621 |
| UC | rs11591147 | 42.77237626 |
| UC | rs653178 | 37.6372512 |
| UC | rs12705390 | 35.29240055 |
| UC | rs9392026 | 108.0394805 |
| UC | rs3778607 | 84.45035746 |
| UC | rs72928038 | 53.06870018 |
| UC | rs3087243 | 52.44553257 |
| UC | rs204295 | 42.22106729 |
| UC | rs2476601 | 33.29128367 |
| UC | rs9851967 | 95.52135384 |
| UC | rs17810546 | 75.06089693 |
| UC | rs13151961 | 74.42239571 |
| UC | rs653178 | 56.36192249 |
| UC | rs2816316 | 50.11751 |
| UC | rs13098911 | 44.51210622 |
| UC | rs11221335 | 43.53962169 |
| UC | rs1018326 | 34.73256097 |
| UC | rs1738074 | 30.61653045 |
| UC | rs13003464 | 29.74740267 |
| UC | rs416352 | 1479.738185 |
| UC | rs1265099 | 1479.736498 |
| UC | rs1264622 | 1479.735003 |
| UC | rs3117326 | 1479.733708 |
| UC | rs521977 | 1479.72934 |
| UC | rs9366717 | 1290.54482 |
| UC | rs2523534 | 1240.027985 |
| UC | rs1264344 | 1172.828436 |
| UC | rs13219354 | 1156.538829 |
| UC | rs2523693 | 1137.865427 |
| UC | rs13198474 | 920.7933941 |
| UC | rs3130357 | 843.5232897 |
| UC | rs23544 | 841.2168935 |
| UC | rs16869648 | 647.6503087 |
| UC | rs1383265 | 557.4883494 |
| UC | rs9296068 | 324.1139529 |

| | | |
|---|---|---|
| UC | rs2030519 | 217.6129001 |
| UC | rs13132308 | 168.1506364 |
| UC | rs2235982 | 165.2298147 |
| UC | rs4947332 | 154.9639815 |
| UC | rs2017525 | 143.4783783 |
| UC | rs17264332 | 129.6121552 |
| UC | rs76830965 | 117.2233821 |
| UC | rs1323292 | 107.0991357 |
| UC | rs4148881 | 102.2553076 |
| UC | rs3184504 | 88.37382666 |
| UC | rs2097282 | 86.92046105 |
| UC | rs2296748 | 83.2303368 |
| UC | rs6934794 | 83.09811547 |
| UC | rs499384 | 81.213784 |
| UC | rs55743914 | 77.79128697 |
| UC | rs1250552 | 69.41738357 |
| UC | rs990171 | 68.56934627 |
| UC | rs12530421 | 66.9080154 |
| UC | rs1018326 | 66.76226128 |
| UC | rs13003464 | 66.0759525 |
| UC | rs182429 | 64.75391564 |
| UC | rs1980422 | 63.72965887 |
| UC | rs6910898 | 58.48921424 |
| UC | rs11066320 | 55.09582463 |
| UC | rs17404424 | 53.86812827 |
| UC | rs61907765 | 52.94579441 |
| UC | rs4947256 | 52.88211844 |
| UC | rs1929848 | 52.16257946 |
| UC | rs16894128 | 49.55212246 |
| UC | rs4445406 | 47.52775009 |
| UC | rs10892258 | 45.25034448 |
| UC | rs7104791 | 45.08247447 |
| UC | rs4821124 | 42.9125254 |
| UC | rs2267632 | 41.90765533 |
| UC | rs12068671 | 41.16791264 |
| UC | rs11875687 | 40.54953979 |
| UC | rs1570760 | 40.35551091 |
| UC | rs1050976 | 36.13798535 |
| UC | rs6933319 | 35.44836417 |
| UC | rs1893592 | 35.20800313 |
| UC | rs7616215 | 33.45953222 |
| UC | rs1378938 | 33.32651768 |
| UC | rs1107943 | 33.28780669 |
| UC | rs6715106 | 33.18500134 |
| UC | rs61579022 | 32.85683346 |
| UC | rs16894722 | 31.86578322 |
| UC | rs11801183 | 31.82516941 |
| UC | rs79758729 | 31.38524655 |
| UC | rs10800746 | 31.00741953 |
| UC | rs744254 | 30.68406801 |
| UC | rs10513548 | 30.03920125 |

| | | |
|---|---|---|
| UC | rs11851414 | 29.834606 |
| UC | rs3130380 | 2488.026097 |
| UC | rs3130834 | 1895.744874 |
| UC | rs9469220 | 1626.502217 |
| UC | rs9268368 | 1390.544471 |
| UC | rs9276612 | 1193.007016 |
| UC | rs13219354 | 1156.678473 |
| UC | rs4540292 | 1067.331181 |
| UC | rs13198474 | 920.515888 |
| UC | rs16869648 | 647.6977466 |
| UC | rs2395296 | 549.4338273 |
| UC | rs2395349 | 405.216881 |
| UC | rs3734840 | 291.3839027 |
| UC | rs2229092 | 230.1286855 |
| UC | rs11753326 | 209.9604293 |
| UC | rs3097657 | 171.3483583 |
| UC | rs13205699 | 137.8277567 |
| UC | rs9258557 | 131.7905808 |
| UC | rs9380294 | 129.0496726 |
| UC | rs9501234 | 118.5916253 |
| UC | rs17351888 | 100 |
| UC | rs2296748 | 83.22947696 |
| UC | rs6934794 | 83.10086982 |
| UC | rs12530421 | 66.91249607 |
| UC | rs12527282 | 54.34636206 |
| UC | rs16894128 | 49.54785641 |
| UC | rs9658119 | 49.18193628 |
| UC | rs10892258 | 45.25278712 |
| UC | rs7104791 | 45.07798795 |
| UC | rs12665489 | 44.715982 |
| UC | rs2267632 | 41.91254141 |
| UC | rs7761745 | 38.36563639 |
| UC | rs1611697 | 36.36086417 |
| UC | rs10752747 | 34.17580435 |
| UC | rs7162232 | 33.28148309 |
| UC | rs16894722 | 31.86599681 |
| UC | rs2441467 | 31.80993363 |
| UC | rs11757750 | 31.25940326 |
| UC | rs13030124 | 31.13618677 |
| UC | rs11979905 | 31.0695865 |
| UC | rs9851967 | 95.32510474 |
| UC | rs17810546 | 74.65691017 |
| UC | rs13151961 | 73.67023001 |
| UC | rs653178 | 56.0625039 |
| UC | rs2816316 | 49.85991365 |
| UC | rs13098911 | 44.23540633 |
| UC | rs1018326 | 34.4837366 |
| UC | rs1738074 | 30.77181067 |
| UC | rs13003464 | 29.37484965 |
| UC | rs13091753 | 66.34364786 |
| UC | rs6933404 | 51.49762868 |

| | | |
|---|---|---|
| UC | rs13132933 | 46.43165397 |
| UC | rs1468791 | 45.04994231 |
| UC | rs11221335 | 43.39365361 |
| UC | rs9819226 | 38.95214004 |
| UC | rs1323292 | 34.38686922 |
| UC | rs9851967 | 95.32510474 |
| UC | rs17810546 | 74.65691017 |
| UC | rs13151961 | 73.67023001 |
| UC | rs653178 | 56.0625039 |
| UC | rs2816316 | 49.85991365 |
| UC | rs13098911 | 44.23540633 |
| UC | rs11221335 | 43.39365361 |
| UC | rs1018326 | 34.4837366 |
| UC | rs1738074 | 30.77181067 |
| UC | rs13003464 | 29.37484965 |
| UC | rs6983267 | 91.83428037 |
| UC | rs4939827 | 70.97770955 |
| UC | rs16892766 | 35.04778755 |
| UC | rs3957146 | 670.2345679 |
| UC | rs2523504 | 82.30249168 |
| UC | rs2237897 | 76.41833716 |
| UC | rs2476601 | 66.35707816 |
| UC | rs11558471 | 45.99794138 |
| UC | rs10938397 | 43.04966681 |
| UC | rs17187735 | 38.90640625 |
| UC | rs76177300 | 36.51612245 |
| UC | rs10974438 | 36.23801588 |
| UC | rs34337125 | 32.2624 |
| UC | rs9765960 | 30.15685148 |
| UC | rs5215 | 30.13785923 |
| UC | rs3957146 | 537.1139442 |
| UC | rs2476601 | 77.30128082 |
| UC | rs34171662 | 69.12311447 |
| UC | rs17187735 | 44.72780591 |
| UC | rs2296329 | 34.84133448 |
| UC | rs878898 | 33.08058423 |
| UC | rs3957146 | 659.0029432 |
| UC | rs2523504 | 80.02994887 |
| UC | rs2237897 | 77.04938272 |
| UC | rs2476601 | 64.58958694 |
| UC | rs11558471 | 46.1041 |
| UC | rs10938397 | 45.21876302 |
| UC | rs11708067 | 41.95053254 |
| UC | rs17187735 | 37.98478668 |
| UC | rs10974438 | 37.07724733 |
| UC | rs76177300 | 35.31430141 |
| UC | rs56258397 | 32.76333321 |
| UC | rs34337125 | 29.97285991 |
| UC | rs5215 | 29.58027905 |
| UC | rs9273363 | 219.6369847 |
| UC | rs1421085 | 95.67277557 |

| | | |
|---|---|---|
| UC | rs849142 | 62.07355484 |
| UC | rs1260326 | 51.3808817 |
| UC | rs697239 | 47.68540027 |
| UC | rs5215 | 41.04837291 |
| UC | rs1800961 | 36.67443398 |
| UC | rs3131064 | 33.03226561 |
| UC | rs2476601 | 30.65820455 |
| UC | rs646123 | 29.81313658 |
| UC | rs3957146 | 609.261834 |
| UC | rs2237897 | 81.56643184 |
| UC | rs2523504 | 78.25443787 |
| UC | rs2476601 | 65.34027778 |
| UC | rs4402960 | 54.57349241 |
| UC | rs10938397 | 47.24893958 |
| UC | rs11558471 | 42.25 |
| UC | rs76177300 | 36.1630802 |
| UC | rs17187735 | 34.8239935 |
| UC | rs853974 | 34.56629013 |
| UC | rs5215 | 31.49062117 |
| UC | rs9765960 | 30.74881374 |
| UC | rs3957146 | 446.3578212 |
| UC | rs2476601 | 67.27738411 |
| UC | rs34018127 | 64.05307899 |
| UC | rs3873444 | 58.75570162 |
| UC | rs2107202 | 35.36190334 |
| UC | rs17187735 | 33.03913015 |
| UC | rs4795399 | 213.1525795 |
| UC | rs2197415 | 166.0926748 |
| UC | rs72743461 | 164.0231007 |
| UC | rs1321859 | 104.035551 |
| UC | rs2621322 | 76.05759964 |
| UC | rs1117490 | 66.23317075 |
| UC | rs17454584 | 59.77524667 |
| UC | rs917115 | 49.25872218 |
| UC | rs35570272 | 48.80394015 |
| UC | rs72799426 | 43.89843058 |
| UC | rs174535 | 39.46309793 |
| UC | rs2338819 | 37.18354855 |
| UC | rs429916 | 36.72699383 |
| UC | rs7771953 | 36.00951948 |
| UC | rs13208164 | 35.02525737 |
| UC | rs41284471 | 34.87325903 |
| UC | rs805287 | 34.14574701 |
| UC | rs2296618 | 33.32483733 |
| UC | rs3781094 | 32.70625219 |
| UC | rs6729966 | 31.35010616 |
| UC | rs12365699 | 29.98577268 |
| UC | rs9375459 | 159.8205842 |
| UC | rs11072508 | 124.9436252 |
| UC | rs1052486 | 89.3645148 |
| UC | rs7838131 | 58.06523158 |

| | | |
|---|---|---|
| UC | rs11864054 | 45.13135868 |
| UC | rs169080 | 44.7912553 |
| UC | rs2246490 | 39.93143088 |
| UC | rs11229555 | 37.49582107 |
| UC | rs27433 | 37.12780233 |
| UC | rs35633336 | 37.00116955 |
| UC | rs57786342 | 32.27754701 |
| UC | rs10072700 | 32.14303234 |
| UC | rs6905288 | 31.8339008 |
| UC | rs74580656 | 31.26109983 |
| UC | rs4146810 | 31.07499639 |
| UC | rs696 | 30.38757938 |
| UC | rs13003198 | 29.72938169 |
| UC | rs11690644 | 247.8241064 |
| UC | rs11742240 | 198.5205289 |
| UC | rs61839660 | 137.9976273 |
| UC | rs72743461 | 90.09936414 |
| UC | rs4845604 | 81.45904723 |
| UC | rs45613035 | 77.33398792 |
| UC | rs2844787 | 69.45999167 |
| UC | rs11171739 | 67.83227124 |
| UC | rs653178 | 63.85847713 |
| UC | rs2228145 | 57.33363979 |
| UC | rs905670 | 52.97274528 |
| UC | rs10912564 | 52.80210761 |
| UC | rs17057868 | 49.83749708 |
| UC | rs10903115 | 47.60590435 |
| UC | rs16858573 | 46.35375184 |
| UC | rs5029949 | 36.47550325 |
| UC | rs12365699 | 35.85211005 |
| UC | rs4939489 | 34.84480371 |
| UC | rs2255088 | 33.70206102 |
| UC | rs2893907 | 32.23625983 |
| UC | rs647397 | 31.11630209 |
| UC | rs2422254 | 30.01641747 |
| UC | rs34463936 | 177.358569 |
| UC | rs72743461 | 152.2769204 |
| UC | rs962993 | 121.0171896 |
| UC | rs61839660 | 106.7358089 |
| UC | rs4845604 | 79.72036465 |
| UC | rs905670 | 77.67955622 |
| UC | rs849335 | 68.72827897 |
| UC | rs45613035 | 66.44838124 |
| UC | rs10903115 | 58.32674249 |
| UC | rs2228145 | 56.70650655 |
| UC | rs6025 | 51.33053693 |
| UC | rs17057868 | 51.16765851 |
| UC | rs10912564 | 45.20357895 |
| UC | rs249677 | 44.6207053 |
| UC | rs12365699 | 43.94522771 |
| UC | rs6011033 | 43.92054271 |

| | | |
|---|---|---|
| UC | rs5029949 | 41.58508412 |
| UC | rs4410871 | 39.48207075 |
| UC | rs2255088 | 38.23508331 |
| UC | rs16858573 | 38.20229185 |
| UC | rs2893907 | 35.54250255 |
| UC | rs11256311 | 33.1705323 |
| UC | rs1177289 | 31.81903734 |
| UC | rs28360011 | 31.51135145 |
| UC | rs72799426 | 31.00951592 |
| UC | rs114420077 | 30.05833404 |
| UC | rs17538393 | 29.7510625 |
| UC | rs4939827 | 89.14101097 |
| UC | rs6983267 | 62.09929775 |
| UC | rs10936599 | 32.11321132 |
| UC | rs11591147 | 118.0998178 |
| UC | rs646776 | 82.44194362 |
| UC | rs499883 | 68.2473268 |
| UC | rs1367117 | 65.30387144 |
| UC | rs4953023 | 48.02547037 |
| UC | rs12916 | 36.36180677 |
| UC | rs2476601 | 401.7161224 |
| UC | rs11571297 | 97.87548308 |
| UC | rs115084931 | 50.83753946 |
| UC | rs1990760 | 40.90932262 |
| UC | rs10905668 | 38.66578512 |
| UC | rs28780083 | 37.02999096 |
| UC | rs11964080 | 33.75907955 |
| UC | rs11102634 | 32.18297214 |
| UC | rs4409785 | 30.37418735 |
| UC | rs1800961 | 47.48195767 |
| UC | rs1260326 | 37.88932141 |
| UC | rs1169286 | 34.98169542 |
| UC | rs11591147 | 81.82562262 |
| UC | rs646776 | 54.243225 |
| UC | rs1367117 | 46.10485028 |
| UC | rs499883 | 45.39801457 |
| UC | rs688 | 32.57346817 |
| UC | rs12916 | 31.60633551 |
| UC | rs646776 | 188.9443931 |
| UC | rs1367117 | 69.49341629 |
| UC | rs1260326 | 68.65754422 |
| UC | rs11591147 | 60.64810791 |
| UC | rs4245791 | 58.01732088 |
| UC | rs12916 | 36.35360002 |
| UC | rs2244608 | 31.25966387 |
| UC | rs2240326 | 96.02888374 |
| UC | rs773109 | 55.63788661 |
| UC | rs2232423 | 45.70598484 |
| UC | rs1716171 | 42.30125524 |
| UC | rs6722661 | 41.55473241 |
| UC | rs205262 | 41.19322308 |

| | | |
|---|---|---|
| UC | rs329122 | 35.14855924 |
| UC | rs3172494 | 33.61569586 |
| UC | rs9615905 | 32.46700519 |
| UC | rs2164300 | 30.0858308 |
| UC | rs17293632 | 139.3426276 |
| UC | rs9847710 | 99.500625 |
| UC | rs72707023 | 53.31755073 |
| UC | rs3072 | 42.40868531 |
| UC | rs1262776 | 41.24493827 |
| UC | rs10460504 | 38.9138322 |
| UC | rs10838738 | 36.58774539 |
| UC | rs2476601 | 269.7717944 |
| UC | rs3087243 | 80.62801427 |
| UC | rs9277557 | 47.87360086 |
| UC | rs2111485 | 33.52242176 |
| UC | rs10974437 | 31.90387634 |
| UC | rs7599564 | 31.55092802 |
| UC | rs28780083 | 29.84229219 |
| UC | rs2476601 | 273.398267 |
| UC | rs3087243 | 82.113389 |
| UC | rs9277557 | 47.24587132 |
| UC | rs117846960 | 34.64073817 |
| UC | rs10974437 | 31.82431474 |
| UC | rs7599564 | 31.81913311 |
| UC | rs28780083 | 30.25 |
| UC | rs2476601 | 259.5679012 |
| UC | rs3184504 | 103.8145002 |
| UC | rs11066320 | 85.7971076 |
| UC | rs11571297 | 65.74141709 |
| UC | rs9277557 | 56.33244777 |
| UC | rs10174238 | 34.62689481 |
| UC | rs2523719 | 32.02530992 |
| UC | rs2476601 | 438.5166638 |
| UC | rs11571297 | 109.5888321 |
| UC | rs9277542 | 72.78222656 |
| UC | rs229541 | 61.12396694 |
| UC | rs1990760 | 49.64430603 |
| UC | rs7757496 | 47.48803739 |
| UC | rs28780083 | 45.47419205 |
| UC | rs10905669 | 42.41402443 |
| UC | rs877636 | 39.78698225 |
| UC | rs10748781 | 38.37418749 |
| UC | rs4409785 | 38.28515625 |
| UC | rs210179 | 32.49974432 |
| UC | rs11102634 | 29.96121884 |
| UC | rs2476601 | 475.24 |
| UC | rs11571297 | 117.9034028 |
| UC | rs9277542 | 80.21359689 |
| UC | rs229541 | 61.44965527 |
| UC | rs1990760 | 59.69873621 |
| UC | rs28780083 | 43.34027778 |

| | | |
|---|---|---|
| UC | rs10748781 | 41.61307444 |
| UC | rs4409785 | 40.74418114 |
| UC | rs41295131 | 39.76626436 |
| UC | rs10517086 | 39.55230713 |
| UC | rs210192 | 30.25 |
| UC | rs2476601 | 437.9855611 |
| UC | rs11571297 | 110.4375797 |
| UC | rs9277542 | 78.765625 |
| UC | rs229541 | 62.98586777 |
| UC | rs1990760 | 51.73436579 |
| UC | rs28780083 | 47.55251736 |
| UC | rs2442719 | 44.72265625 |
| UC | rs10748781 | 41.1173438 |
| UC | rs877636 | 41.09138725 |
| UC | rs4409785 | 40.02319637 |
| UC | rs6448432 | 38.95840278 |
| UC | rs210179 | 32.49974432 |
| UC | rs11102634 | 30.82967382 |
| UC | rs12567136 | 174.6834486 |
| UC | rs11072508 | 135.5905439 |
| UC | rs7838131 | 67.47701147 |
| UC | rs3764769 | 46.70771246 |
| UC | rs169080 | 42.21912725 |
| UC | rs35633336 | 36.17134696 |
| UC | rs736801 | 34.30657189 |
| UC | rs10409243 | 33.90872489 |
| UC | rs4713488 | 33.54699275 |
| UC | rs27433 | 32.72883438 |
| UC | rs696 | 31.46061655 |
| UC | rs11229555 | 31.07838325 |
| UC | rs11591147 | 57.5872865 |
| UC | rs12705390 | 53.29 |
| UC | rs653178 | 34.93654337 |
| UC | rs2495477 | 33.45215486 |
| UC | rs1044506 | 33.04486432 |
| UC | rs3957146 | 779.2267995 |
| UC | rs1121980 | 104.1940192 |
| UC | rs2523504 | 96.85840278 |
| UC | rs2237897 | 77.00957934 |
| UC | rs2476601 | 76.44430241 |
| UC | rs10938397 | 50.73202207 |
| UC | rs9765960 | 43.71837714 |
| UC | rs28624681 | 41.5308642 |
| UC | rs11558471 | 41.00698594 |
| UC | rs76177300 | 38.86915577 |
| UC | rs4148881 | 36.39290296 |
| UC | rs7100984 | 32.33560758 |
| UC | rs34337125 | 31.75901825 |
| UC | rs4273712 | 31.66446423 |
| UC | rs3957146 | 778.6116998 |
| UC | rs1121980 | 104.3452703 |

| | | |
|---|---|---|
| UC | rs2523504 | 97.8627826 |
| UC | rs2237897 | 80.90911642 |
| UC | rs2476601 | 76.23985406 |
| UC | rs10938397 | 51.51751245 |
| UC | rs11558471 | 42.72404959 |
| UC | rs9765960 | 41.58026159 |
| UC | rs76177300 | 39.43181862 |
| UC | rs4148881 | 34.65790843 |
| UC | rs34337125 | 33.16906722 |
| UC | rs853974 | 31.17361111 |
| UC | rs7100984 | 30.8025 |
| UC | rs9271100 | 1153.78976 |
| UC | rs2857700 | 930.1440187 |
| UC | rs9469220 | 808.7316572 |
| UC | rs9266629 | 238.0532385 |
| UC | rs722788 | 129.5756482 |
| UC | rs12927355 | 114.0780906 |
| UC | rs9277641 | 104.8002141 |
| UC | rs2104286 | 95.65667554 |
| UC | rs13194491 | 79.16415234 |
| UC | rs1813375 | 74.64878281 |
| UC | rs2394423 | 72.05492404 |
| UC | rs41286801 | 64.78177037 |
| UC | rs1800693 | 64.71669962 |
| UC | rs8070345 | 64.71669962 |
| UC | rs1920296 | 59.88255844 |
| UC | rs11554159 | 53.94065835 |
| UC | rs1077667 | 53.28033723 |
| UC | rs2255214 | 52.36224806 |
| UC | rs4976646 | 51.53151101 |
| UC | rs3748817 | 50.68870328 |
| UC | rs7762619 | 49.77234597 |
| UC | rs3129269 | 49.43856505 |
| UC | rs17066096 | 47.45176818 |
| UC | rs12087340 | 47.25406297 |
| UC | rs11172342 | 46.2049889 |
| UC | rs2071292 | 46.07892099 |
| UC | rs74796499 | 41.9955859 |
| UC | rs6498184 | 39.62359077 |
| UC | rs34383631 | 39.44266262 |
| UC | rs1870071 | 38.54918016 |
| UC | rs1021156 | 38.15633434 |
| UC | rs2288904 | 37.9201961 |
| UC | rs1014486 | 36.84544637 |
| UC | rs9967792 | 36.2725284 |
| UC | rs11154801 | 36.20743486 |
| UC | rs4410871 | 36.14245546 |
| UC | rs55838263 | 36.0921596 |
| UC | rs842639 | 35.61351815 |
| UC | rs71624119 | 35.10542979 |
| UC | rs7923837 | 34.81640662 |

| | | |
|---|---|---|
| UC | rs941816 | 34.81320201 |
| UC | rs6881706 | 34.0714384 |
| UC | rs6880778 | 32.36681908 |
| UC | rs9380294 | 32.22777182 |
| UC | rs917116 | 31.70531526 |
| UC | rs4796791 | 31.5258197 |
| UC | rs6933319 | 30.42722174 |
| UC | rs3748080 | 29.73972414 |
| UC | rs67297943 | 29.52202688 |
| UC | rs9271100 | 1151.631086 |
| UC | rs2857700 | 921.3435307 |
| UC | rs9469220 | 814.5751003 |
| UC | rs9266629 | 240.2826901 |
| UC | rs722788 | 128.8075972 |
| UC | rs12927355 | 114.9205234 |
| UC | rs9277641 | 105.0513448 |
| UC | rs2104286 | 96.81623181 |
| UC | rs3135333 | 88.71538787 |
| UC | rs13194491 | 78.0008487 |
| UC | rs1813375 | 74.60467438 |
| UC | rs2394423 | 73.02581602 |
| UC | rs8070345 | 65.6342256 |
| UC | rs1800693 | 65.15622698 |
| UC | rs41286801 | 64.88992935 |
| UC | rs212407 | 62.57878754 |
| UC | rs57271503 | 61.6464492 |
| UC | rs1920296 | 60.66954042 |
| UC | rs11554159 | 53.5042742 |
| UC | rs1077667 | 52.88306485 |
| UC | rs1323292 | 52.80705178 |
| UC | rs2255214 | 52.07945023 |
| UC | rs4976646 | 50.76723335 |
| UC | rs3748817 | 50.28489445 |
| UC | rs7762619 | 49.75718908 |
| UC | rs12087340 | 47.63652841 |
| UC | rs17066096 | 47.3591021 |
| UC | rs11172342 | 46.43729685 |
| UC | rs2071292 | 46.00408962 |
| UC | rs74796499 | 42.14598704 |
| UC | rs6498184 | 40.39956714 |
| UC | rs1021156 | 38.45620754 |
| UC | rs1870071 | 38.42876024 |
| UC | rs34383631 | 38.42542586 |
| UC | rs2745400 | 37.80488558 |
| UC | rs2288904 | 37.41094708 |
| UC | rs1014486 | 37.03575872 |
| UC | rs2364482 | 36.66893084 |
| UC | rs55838263 | 36.65497369 |
| UC | rs842639 | 36.29036119 |
| UC | rs9967792 | 36.17935672 |
| UC | rs4410871 | 35.99321002 |

| | | |
|---|---|---|
| UC | rs11154801 | 35.65969173 |
| UC | rs71624119 | 35.38911173 |
| UC | rs941816 | 34.40743197 |
| UC | rs7923837 | 34.36020519 |
| UC | rs6881706 | 34.24060615 |
| UC | rs9380294 | 32.18739863 |
| UC | rs6880778 | 31.8103382 |
| UC | rs4796791 | 31.68833603 |
| UC | rs917116 | 31.42801881 |
| UC | rs1059288 | 31.3452092 |
| UC | rs6933319 | 30.45318115 |
| UC | rs67297943 | 29.78362904 |
| UC | rs3134954 | 1116.320754 |
| UC | rs2844670 | 388.3801045 |
| UC | rs16896742 | 229.2913396 |
| UC | rs404860 | 163.5528652 |
| UC | rs9276711 | 122.2844658 |
| UC | rs9348876 | 79.48075962 |
| UC | rs17500468 | 62.48042532 |
| UC | rs4648356 | 57.68892884 |
| UC | rs7200786 | 56.26968676 |
| UC | rs4613763 | 56.06171561 |
| UC | rs13194491 | 53.66838073 |
| UC | rs11154801 | 50.04225264 |
| UC | rs1077667 | 49.37731631 |
| UC | rs669607 | 44.17564017 |
| UC | rs1738074 | 43.06640625 |
| UC | rs396090 | 42.91176937 |
| UC | rs1800693 | 40.65524787 |
| UC | rs17066096 | 39.43963441 |
| UC | rs2119704 | 39.39924413 |
| UC | rs11581062 | 39.23439198 |
| UC | rs2293370 | 37.18024985 |
| UC | rs9282641 | 36.49615105 |
| UC | rs650258 | 36.2064142 |
| UC | rs1335532 | 35.95597677 |
| UC | rs3118470 | 35.93785068 |
| UC | rs2248359 | 34.11927075 |
| UC | rs11129295 | 32.58482078 |
| UC | rs4902647 | 30.27350884 |
| UC | rs874628 | 29.99117201 |
| UC | rs2857700 | 1043.603238 |
| UC | rs4148874 | 686.7725682 |
| UC | rs2844787 | 124.5897171 |
| UC | rs11256593 | 115.2953852 |
| UC | rs28732150 | 94.64364232 |
| UC | rs2071463 | 86.33740546 |
| UC | rs11749040 | 71.01785716 |
| UC | rs438613 | 69.08375957 |
| UC | rs1026916 | 55.32806096 |
| UC | rs6032662 | 53.31217807 |

| | | |
|---|---|---|
| UC | rs1077667 | 51.19208668 |
| UC | rs2546890 | 50.76912939 |
| UC | rs35540610 | 48.76956315 |
| UC | rs631204 | 47.18824872 |
| UC | rs17124032 | 47.00466038 |
| UC | rs1860545 | 46.81876026 |
| UC | rs1738074 | 46.34664316 |
| UC | rs701006 | 45.74151198 |
| UC | rs13327021 | 45.25801287 |
| UC | rs1250551 | 44.41118521 |
| UC | rs59655222 | 43.73604334 |
| UC | rs72928038 | 42.02554183 |
| UC | rs1014486 | 41.21439181 |
| UC | rs7592560 | 39.75841812 |
| UC | rs12365699 | 39.58166659 |
| UC | rs9843355 | 38.78762253 |
| UC | rs10063294 | 37.0867685 |
| UC | rs35703946 | 36.03508161 |
| UC | rs3130171 | 34.77546197 |
| UC | rs354033 | 32.47876532 |
| UC | rs7855251 | 30.03961833 |
| UC | rs2232423 | 78.44897959 |
| UC | rs2568958 | 75.37396694 |
| UC | rs13037326 | 40.02498959 |
| UC | rs4141983 | 32.93761815 |
| UC | rs17641524 | 32.03987184 |
| UC | rs9831648 | 31.53254438 |
| UC | rs2671427 | 38.71455701 |
| UC | rs2666957 | 35.19131503 |
| UC | rs7153386 | 29.98211979 |
| UC | rs7553711 | 48.40858921 |
| UC | rs34593439 | 36.4330083 |
| UC | rs10995245 | 29.79195949 |
| UC | rs962993 | 42.02303291 |
| UC | rs17027258 | 39.09494618 |
| UC | rs34540843 | 30.8216409 |
| UC | rs3774354 | 46.03364845 |
| UC | rs56116847 | 32.06390625 |
| UC | rs10758594 | 32.0620678 |
| UC | rs7774434 | 250.1841762 |
| UC | rs6679356 | 119.6655099 |
| UC | rs10488631 | 97.61563466 |
| UC | rs10947260 | 67.70253747 |
| UC | rs2293370 | 61.57651862 |
| UC | rs12924729 | 58.18593463 |
| UC | rs4938573 | 55.0940682 |
| UC | rs243325 | 48.73339793 |
| UC | rs17641524 | 46.30242557 |
| UC | rs2069235 | 44.75387889 |
| UC | rs9303277 | 44.48022503 |
| UC | rs860413 | 43.28748258 |

| | | |
|---|---|---|
| UC | rs2304256 | 41.72722106 |
| UC | rs7665090 | 37.70934918 |
| UC | rs1800693 | 36.1412363 |
| UC | rs911263 | 35.74633426 |
| UC | rs11065987 | 30.58355034 |
| UC | rs7775055 | 220.8673499 |
| UC | rs72678531 | 167.6055727 |
| UC | rs522127 | 94.11121064 |
| UC | rs35188261 | 92.56254669 |
| UC | rs9295947 | 65.38650962 |
| UC | rs2293370 | 65.18043975 |
| UC | rs17122453 | 64.94228033 |
| UC | rs1646019 | 60.6790643 |
| UC | rs1800693 | 59.57663455 |
| UC | rs10931468 | 56.99870129 |
| UC | rs12708715 | 56.80966557 |
| UC | rs8067378 | 56.35437544 |
| UC | rs7665090 | 55.69208254 |
| UC | rs2267407 | 54.86217665 |
| UC | rs6871748 | 53.76214643 |
| UC | rs12134279 | 48.13705887 |
| UC | rs911263 | 41.83114872 |
| UC | rs1014486 | 41.68009932 |
| UC | rs3998159 | 38.752453 |
| UC | rs17564829 | 35.83475138 |
| UC | rs11065979 | 35.26890051 |
| UC | rs34725611 | 34.50840924 |
| UC | rs11064157 | 34.07174106 |
| UC | rs11117431 | 32.86547623 |
| UC | rs2523647 | 30.51916156 |
| UC | rs10847864 | 50.65622172 |
| UC | rs620490 | 38.17939058 |
| UC | rs4488803 | 32.58745991 |
| UC | rs6983267 | 643.4891844 |
| UC | rs10840603 | 302.6889838 |
| UC | rs651164 | 157.9591942 |
| UC | rs1058319 | 103.4157778 |
| UC | rs17749618 | 51.33301038 |
| UC | rs2305498 | 46.5124 |
| UC | rs2523733 | 114.6894657 |
| UC | rs2071463 | 68.61710623 |
| UC | rs9263875 | 55.15399286 |
| UC | rs74817271 | 43.29014502 |
| UC | rs12713428 | 33.00060592 |
| UC | rs13201757 | 1019.640594 |
| UC | rs9391734 | 397.5892951 |
| UC | rs28780084 | 205.7241026 |
| UC | rs2199874 | 103.7710131 |
| UC | rs7741091 | 87.83161454 |
| UC | rs926070 | 66.26200654 |
| UC | rs4845454 | 63.07969313 |

| | | |
|---|---|---|
| UC | rs6457109 | 62.21317999 |
| UC | rs17728338 | 60.48454624 |
| UC | rs2546890 | 50.60564096 |
| UC | rs141417497 | 47.92730231 |
| UC | rs72669160 | 46.61059326 |
| UC | rs1416173 | 45.30658757 |
| UC | rs114835245 | 44.79300763 |
| UC | rs78580783 | 42.2007555 |
| UC | rs9461362 | 42.00074802 |
| UC | rs68191 | 39.83862541 |
| UC | rs77840275 | 39.65505709 |
| UC | rs892085 | 39.02536227 |
| UC | rs30376 | 38.63362758 |
| UC | rs9399137 | 37.54827919 |
| UC | rs3868078 | 34.72041834 |
| UC | rs80174646 | 34.37441395 |
| UC | rs9262155 | 33.9142097 |
| UC | rs2074478 | 32.98833642 |
| UC | rs62421044 | 31.808472 |
| UC | rs1990760 | 31.54190303 |
| UC | rs11652075 | 31.27935367 |
| UC | rs12447534 | 31.0861904 |
| UC | rs117950837 | 30.31483062 |
| UC | rs11564177 | 30.03806355 |
| UC | rs2476601 | 317.0948351 |
| UC | rs8283 | 226.3014613 |
| UC | rs2074488 | 178.4507845 |
| UC | rs4711211 | 144.0976519 |
| UC | rs106287 | 115.8059851 |
| UC | rs6922431 | 76.20597332 |
| UC | rs456261 | 61.10810475 |
| UC | rs2532919 | 53.34238971 |
| UC | rs6901724 | 51.3848525 |
| UC | rs6920220 | 49.05173894 |
| UC | rs10040327 | 42.61910456 |
| UC | rs3909134 | 40.93414998 |
| UC | rs4810485 | 33.93781964 |
| UC | rs3087243 | 31.27446019 |
| UC | rs11757182 | 30.80494096 |
| UC | rs2267632 | 30.76140683 |
| UC | rs2476601 | 869.340315 |
| UC | rs3130379 | 272.8979258 |
| UC | rs1571878 | 165.34496 |
| UC | rs3893538 | 140.3479735 |
| UC | rs6456964 | 133.7007259 |
| UC | rs8026898 | 129.8023912 |
| UC | rs7752903 | 126.8912923 |
| UC | rs34695944 | 100.9924468 |
| UC | rs4239702 | 100.9924468 |
| UC | rs29243 | 76.07991245 |
| UC | rs9348832 | 68.80725792 |

| | | |
|---|---|---|
| UC | rs1858036 | 66.93076603 |
| UC | rs3087243 | 64.82443647 |
| UC | rs17404424 | 61.55848945 |
| UC | rs11933540 | 60.50338484 |
| UC | rs2561477 | 57.74592653 |
| UC | rs2301888 | 55.86258863 |
| UC | rs1893592 | 55.73265308 |
| UC | rs6712515 | 55.73265308 |
| UC | rs11889341 | 55.45614566 |
| UC | rs3806624 | 55.28948982 |
| UC | rs8032939 | 44.37181597 |
| UC | rs3128935 | 43.43043586 |
| UC | rs11574914 | 40.06789519 |
| UC | rs62097857 | 38.20344257 |
| UC | rs4409785 | 37.10507058 |
| UC | rs1953126 | 36.62845648 |
| UC | rs947474 | 33.38311413 |
| UC | rs73081554 | 31.68823116 |
| UC | rs9268145 | 1121.704453 |
| UC | rs2476601 | 275.0492786 |
| UC | rs10484565 | 159.9238633 |
| UC | rs1029237 | 143.5033085 |
| UC | rs1431403 | 138.0943099 |
| UC | rs544167 | 132.6617645 |
| UC | rs2524084 | 117.9188209 |
| UC | rs13196329 | 106.592422 |
| UC | rs6922431 | 91.49959803 |
| UC | rs2267644 | 89.36491131 |
| UC | rs620202 | 85.36086947 |
| UC | rs71624119 | 83.75814367 |
| UC | rs2296330 | 69.52736813 |
| UC | rs2531804 | 54.63181621 |
| UC | rs361525 | 53.6871644 |
| UC | rs462618 | 53.19226861 |
| UC | rs6920220 | 40.63258014 |
| UC | rs17630466 | 40.07416584 |
| UC | rs13426947 | 37.96899322 |
| UC | rs194679 | 37.77708423 |
| UC | rs8026898 | 35.76860251 |
| UC | rs10209110 | 32.6104239 |
| UC | rs2812378 | 32.32107356 |
| UC | rs2228145 | 32.30745226 |
| UC | rs2240339 | 32.24777667 |
| UC | rs2476601 | 678.2551357 |
| UC | rs3749946 | 98.08874685 |
| UC | rs3087243 | 82.77339454 |
| UC | rs17264332 | 78.73552445 |
| UC | rs68191 | 73.15273165 |
| UC | rs8026898 | 71.78450094 |
| UC | rs34046593 | 69.13361899 |
| UC | rs17404424 | 66.33736053 |

| | | |
|---|---|---|
| UC | rs1571878 | 61.30077778 |
| UC | rs4239702 | 57.07239393 |
| UC | rs34695944 | 56.98157996 |
| UC | rs11574914 | 54.57048351 |
| UC | rs12764378 | 54.10525646 |
| UC | rs4713358 | 49.58459824 |
| UC | rs13426947 | 49.1263451 |
| UC | rs8032939 | 49.12630299 |
| UC | rs9653442 | 48.33154658 |
| UC | rs12539741 | 45.97130691 |
| UC | rs2069235 | 39.67457119 |
| UC | rs2561477 | 38.60115167 |
| UC | rs2451258 | 38.13561104 |
| UC | rs9368696 | 37.32477138 |
| UC | rs2240336 | 36.66918076 |
| UC | rs10796038 | 35.97381764 |
| UC | rs12232497 | 34.82901639 |
| UC | rs592390 | 34.7234128 |
| UC | rs61828284 | 33.11201994 |
| UC | rs1893592 | 32.8805961 |
| UC | rs10985070 | 31.81033859 |
| UC | rs9310852 | 30.58230767 |
| UC | rs624988 | 29.87842993 |
| UC | rs4936059 | 29.87840789 |
| UC | rs73081554 | 29.83663305 |
| UC | rs9268145 | 1581.710785 |
| UC | rs2476601 | 302.4266715 |
| UC | rs8283 | 224.880682 |
| UC | rs2074488 | 168.2536436 |
| UC | rs4711211 | 143.8448016 |
| UC | rs106287 | 119.7872959 |
| UC | rs6922431 | 74.83083318 |
| UC | rs456261 | 57.33781905 |
| UC | rs2532919 | 52.74731825 |
| UC | rs6901724 | 49.46524787 |
| UC | rs6920220 | 46.04012992 |
| UC | rs10040327 | 42.6826205 |
| UC | rs3909134 | 40.09318172 |
| UC | rs3087243 | 35.19521763 |
| UC | rs2267632 | 30.81277589 |
| UC | rs11757182 | 30.51698516 |
| UC | rs4810485 | 29.25598476 |
| UC | rs2476601 | 678.1835787 |
| UC | rs8192585 | 231.0436375 |
| UC | rs3087243 | 82.81707269 |
| UC | rs17264332 | 78.80610491 |
| UC | rs68191 | 73.16315931 |
| UC | rs8026898 | 71.82587412 |
| UC | rs11933540 | 69.06149303 |
| UC | rs7738250 | 68.84871266 |
| UC | rs17404424 | 66.29695683 |

| | | |
|---|---|---|
| UC | rs1571878 | 61.29400579 |
| UC | rs4239702 | 57.07597389 |
| UC | rs34695944 | 56.92861801 |
| UC | rs11574914 | 54.61975304 |
| UC | rs12764378 | 54.04717811 |
| UC | rs13426947 | 49.11796565 |
| UC | rs8032939 | 49.11329476 |
| UC | rs9653442 | 48.37975309 |
| UC | rs2069235 | 39.62299901 |
| UC | rs2561477 | 38.57806634 |
| UC | rs2451258 | 38.19013214 |
| UC | rs9368696 | 37.29145521 |
| UC | rs2240336 | 36.59887734 |
| UC | rs10796038 | 35.95196798 |
| UC | rs592390 | 34.74349906 |
| UC | rs61828284 | 33.11008605 |
| UC | rs9469591 | 32.9190277 |
| UC | rs1893592 | 32.88498791 |
| UC | rs10985070 | 31.79931288 |
| UC | rs9310852 | 30.57771989 |
| UC | rs624988 | 29.90069577 |
| UC | rs4936059 | 29.85748686 |
| UC | rs73081554 | 29.83558431 |
| UC | rs3957146 | 389.9863469 |
| UC | rs2071463 | 46.07410256 |
| UC | rs2476601 | 41.76513956 |
| UC | rs1077394 | 41.62166062 |
| UC | rs1419675 | 34.68512776 |
| UC | rs4274624 | 293.2513845 |
| UC | rs1143679 | 212.0016865 |
| UC | rs35000415 | 200.2294151 |
| UC | rs6889239 | 76.51123259 |
| UC | rs58721818 | 75.6557835 |
| UC | rs2299851 | 61.01868032 |
| UC | rs2431697 | 58.01521611 |
| UC | rs6679677 | 52.39196829 |
| UC | rs7768653 | 48.6189269 |
| UC | rs353608 | 44.22170731 |
| UC | rs58688157 | 44.19823106 |
| UC | rs4916215 | 43.15163271 |
| UC | rs7097397 | 42.11576829 |
| UC | rs78517564 | 40.56838264 |
| UC | rs2459611 | 33.36980543 |
| UC | rs73068668 | 29.96640854 |
| UC | rs2187668 | 91.39273059 |
| UC | rs11229 | 72.57258846 |
| UC | rs7574865 | 57.2268362 |
| UC | rs10488631 | 53.6121581 |
| UC | rs3132631 | 52.23451543 |
| UC | rs16891725 | 50.9436309 |
| UC | rs853676 | 40.64794627 |

| | | |
|---|---|---|
| UC | rs3749971 | 38.3082367 |
| UC | rs1024582 | 73.96598273 |
| UC | rs1615350 | 47.86839844 |
| UC | rs11682175 | 45.46470849 |
| UC | rs2414718 | 40.26133231 |
| UC | rs11874716 | 37.32210249 |
| UC | rs11210892 | 34.75563863 |
| UC | rs3849046 | 32.87391647 |
| UC | rs10108725 | 30.29467814 |
| UC | rs9268145 | 983.2118523 |
| UC | rs485502 | 413.8945438 |
| UC | rs2476601 | 255.0132983 |
| UC | rs2071463 | 222.2042068 |
| UC | rs1419675 | 109.3444597 |
| UC | rs3184504 | 65.09399728 |
| UC | rs11066320 | 50.67509336 |
| UC | rs705702 | 45.1584 |
| UC | rs11571297 | 32.42802122 |
| UC | rs3957146 | 721.0693728 |
| UC | rs2071463 | 80.77267157 |
| UC | rs2476601 | 75.94293215 |
| UC | rs2256539 | 59.24542041 |
| UC | rs6905949 | 52.83961648 |
| UC | rs3842727 | 892.1879561 |
| UC | rs6679677 | 554.8636935 |
| UC | rs3184504 | 196.000884 |
| UC | rs61839660 | 171.9010403 |
| UC | rs1701704 | 137.7539958 |
| UC | rs11066320 | 120.9991893 |
| UC | rs12927355 | 94.09039781 |
| UC | rs3087243 | 87.76771775 |
| UC | rs8056814 | 80.41995498 |
| UC | rs2111485 | 75.41625824 |
| UC | rs1893217 | 64 |
| UC | rs11203203 | 62.32704479 |
| UC | rs12416116 | 61.73474733 |
| UC | rs516246 | 56.64517909 |
| UC | rs72928038 | 56.24953121 |
| UC | rs34593439 | 55.57010009 |
| UC | rs4820830 | 50.48474486 |
| UC | rs6827756 | 47.53720135 |
| UC | rs56994090 | 46.09686261 |
| UC | rs7239671 | 45.18840856 |
| UC | rs2611215 | 45.15835268 |
| UC | rs2304256 | 43.81151828 |
| UC | rs151233 | 43.25597164 |
| UC | rs6043409 | 39.6899937 |
| UC | rs72727394 | 39.34692084 |
| UC | rs2045258 | 37.91982455 |
| UC | rs1574285 | 36.63460047 |
| UC | rs41295121 | 33.99306255 |

| | | |
|---|---|---|
| UC | rs7893467 | 33.23180199 |
| UC | rs12150079 | 32.49014245 |
| UC | rs229533 | 31.71466584 |
| UC | rs60918750 | 31.59219765 |
| UC | rs62447205 | 31.0406647 |
| UC | rs9585056 | 30.5125418 |
| UC | rs4849135 | 29.98855034 |
| UC | rs113010081 | 29.88453674 |
| UC | rs402072 | 29.82858008 |
| UC | rs2596560 | 687.9581449 |
| UC | rs6679677 | 355.8566023 |
| UC | rs3129809 | 273.9222027 |
| UC | rs6913635 | 214.3114623 |
| UC | rs9275601 | 199.7820048 |
| UC | rs9276943 | 189.7241355 |
| UC | rs9267536 | 117.9153838 |
| UC | rs9467704 | 117.0559971 |
| UC | rs11571297 | 68.67304029 |
| UC | rs741172 | 62.15305571 |
| UC | rs2394392 | 61.12668492 |
| UC | rs12722495 | 59.41840278 |
| UC | rs3094138 | 41.00227505 |
| UC | rs8056814 | 40.49865583 |
| UC | rs2111485 | 40.43523998 |
| UC | rs202520 | 37.75526428 |
| UC | rs194749 | 33.9793569 |
| UC | rs2596560 | 1498.833347 |
| UC | rs1894406 | 838.3230436 |
| UC | rs387608 | 465.5808626 |
| UC | rs2535319 | 391.6576889 |
| UC | rs2071463 | 366.3098416 |
| UC | rs2476601 | 331.0880737 |
| UC | rs149943 | 288.6743954 |
| UC | rs3871466 | 150.5002563 |
| UC | rs4759229 | 104.2639664 |
| UC | rs12722496 | 103.4254097 |
| UC | rs12927355 | 72.04860879 |
| UC | rs80054410 | 53.56278678 |
| UC | rs2111485 | 45.43818071 |
| UC | rs12982646 | 43.70844043 |
| UC | rs151233 | 43.61333085 |
| UC | rs7122407 | 43.17035171 |
| UC | rs13415465 | 39.54962553 |
| UC | rs4238595 | 36.37755316 |
| UC | rs34725611 | 35.43176379 |
| UC | rs2281808 | 30.63143581 |
| UC | rs9911533 | 30.57604133 |
| UC | rs3024505 | 30.00770331 |
| UC | rs9268145 | 949.1737935 |
| UC | rs485502 | 392.5124358 |
| UC | rs2476601 | 233.2373085 |

| | | |
|---|---|---|
| UC | rs2071463 | 197.2503956 |
| UC | rs1419675 | 99.79392071 |
| UC | rs3184504 | 60.28203987 |
| UC | rs11066320 | 44.06965108 |
| UC | rs705699 | 42.94820984 |
| UC | rs9277932 | 40.11111111 |
| UC | rs34337125 | 30.32751438 |
| UC | rs10484565 | 461.6877968 |
| UC | rs34171662 | 190.7876263 |
| UC | rs2476601 | 190.1168939 |
| UC | rs3135333 | 131.6059263 |
| UC | rs2256539 | 107.9631666 |
| UC | rs3025643 | 86.4151886 |
| UC | rs9268145 | 978.2427824 |
| UC | rs485502 | 404.8277578 |
| UC | rs2476601 | 241.5913733 |
| UC | rs2071463 | 205.091692 |
| UC | rs2256539 | 122.6913534 |
| UC | rs3184504 | 57.63071073 |
| UC | rs11066320 | 44.08734918 |
| UC | rs11571297 | 36.55441957 |
| UC | rs41176 | 33.42431445 |
| UC | rs2237897 | 97.08044983 |
| UC | rs11558471 | 53.82105098 |
| UC | rs10938397 | 48.61892562 |
| UC | rs80134320 | 32.86787202 |
| UC | rs5215 | 32.59371901 |
| UC | rs10974438 | 32.47991229 |
| UC | rs28624681 | 54.80590109 |
| UC | rs10938397 | 50.32507853 |
| UC | rs11558471 | 49.11577078 |
| UC | rs76177300 | 42.17234647 |
| UC | rs498475 | 32.4526337 |
| UC | rs1053924 | 31.09893974 |
| UC | rs2383208 | 199.2238757 |
| UC | rs13266634 | 92.73731617 |
| UC | rs11708067 | 45.64088074 |
| UC | rs864745 | 45.64088074 |
| UC | rs5215 | 36.62845648 |
| UC | rs7756992 | 276.4972058 |
| UC | rs10811661 | 256.3266347 |
| UC | rs849135 | 192.515625 |
| UC | rs11708067 | 125.9092753 |
| UC | rs17334919 | 119.2873535 |
| UC | rs780094 | 87.4477721 |
| UC | rs5215 | 86.26083693 |
| UC | rs7729395 | 73.63785156 |
| UC | rs340874 | 73.5364984 |
| UC | rs17168486 | 62.30918968 |
| UC | rs10974438 | 62.0944 |
| UC | rs6795735 | 58.42822293 |

| | | |
|---|---|---|
| UC | rs2857605 | 55.75111111 |
| UC | rs12970134 | 48.12890625 |
| UC | rs10401969 | 47.95302165 |
| UC | rs853974 | 46.64269112 |
| UC | rs2071479 | 42.48040175 |
| UC | rs67232546 | 38.54340278 |
| UC | rs2237892 | 37.38894073 |
| UC | rs1265099 | 37.30898466 |
| UC | rs9940149 | 37.27423823 |
| UC | rs940904 | 36.14472347 |
| UC | rs17405722 | 35.50853819 |
| UC | rs16988333 | 32.84171598 |
| UC | rs7561798 | 30.86419753 |
| UC | rs7756992 | 164.8529188 |
| UC | rs13266634 | 107.9001563 |
| UC | rs864745 | 72.94083272 |
| UC | rs11708067 | 72.90532544 |
| UC | rs5215 | 65.69529086 |
| UC | rs2943641 | 58.51256536 |
| UC | rs60980157 | 55.99747507 |
| UC | rs1260326 | 54.60634171 |
| UC | rs2191349 | 50.52520088 |
| UC | rs7202877 | 46.97263534 |
| UC | rs340874 | 44.44444444 |
| UC | rs1077394 | 43.32639 |
| UC | rs35658696 | 40.86371257 |
| UC | rs35720761 | 37.52849375 |
| UC | rs4607517 | 33.4084 |
| UC | rs10146997 | 32.87111111 |
| UC | rs17782313 | 32.15405475 |
| UC | rs6795735 | 30.39490997 |
| UC | rs10758593 | 29.73884444 |
| UC | rs1727307 | 24.51457466 |
| UC | rs2237897 | 86.14160156 |
| UC | rs3129871 | 55.65424074 |
| UC | rs11558471 | 49.52476199 |
| UC | rs10938397 | 43.0033284 |
| UC | rs76177300 | 40.2824649 |
| UC | rs2256594 | 38.14080425 |
| UC | rs498475 | 33.48979767 |
| UC | rs5215 | 31.74889053 |
| UC | rs10974438 | 29.78915189 |
| UC | rs2237897 | 96.74337269 |
| UC | rs11558471 | 54.04236669 |
| UC | rs10938397 | 47.07484568 |
| UC | rs76177300 | 35.02394842 |
| UC | rs5215 | 32.53223594 |
| UC | rs7901695 | 952.6896859 |
| UC | rs7756992 | 248.6686391 |
| UC | rs13266634 | 176.733564 |
| UC | rs864745 | 136.232666 |

| | | |
|---|---|---|
| UC | rs7501939 | 95.85041623 |
| UC | rs1260326 | 93.44444444 |
| UC | rs11708067 | 91.25277008 |
| UC | rs2943641 | 77.02027178 |
| UC | rs2191349 | 66.15111111 |
| UC | rs10758593 | 61.6611126 |
| UC | rs60980157 | 60.0625 |
| UC | rs35658696 | 56.79347511 |
| UC | rs340874 | 55.39263639 |
| UC | rs35720761 | 52.67022841 |
| UC | rs2395163 | 46.63452216 |
| UC | rs7202877 | 43.53278776 |
| UC | rs17782313 | 39.87020408 |
| UC | rs10146997 | 36.30834977 |
| UC | rs730497 | 34.5337601 |
| UC | rs1727307 | 34.17751479 |
| UC | rs4607103 | 31.58123388 |
| UC | rs9388489 | 26.88023669 |
| UC | rs1065461 | 26.69444444 |
| UC | rs665268 | 26.01 |
| UC | rs1800961 | 23.73854938 |
| UC | rs2237897 | 97.23333581 |
| UC | rs11558471 | 52.82174745 |
| UC | rs10938397 | 47.20198903 |
| UC | rs76177300 | 35.69032402 |
| UC | rs5215 | 32.84987997 |
| UC | rs7901695 | 979.3217993 |
| UC | rs7756992 | 256.4778347 |
| UC | rs4402960 | 193.8845271 |
| UC | rs13266634 | 174.0071367 |
| UC | rs864745 | 116.307929 |
| UC | rs11708067 | 115.7003616 |
| UC | rs7501939 | 94.45410156 |
| UC | rs2943641 | 76.37240076 |
| UC | rs1260326 | 69.78674556 |
| UC | rs60980157 | 60.60326871 |
| UC | rs340874 | 59.51020408 |
| UC | rs7202877 | 53.77777778 |
| UC | rs35658696 | 48.76029388 |
| UC | rs35720761 | 48.06222263 |
| UC | rs10758593 | 40.17924037 |
| UC | rs2395163 | 39.1426693 |
| UC | rs6905288 | 38.05940828 |
| UC | rs4607103 | 36.17412308 |
| UC | rs1727307 | 32.84829583 |
| UC | rs7961581 | 31.29510607 |
| UC | rs1065461 | 27.49159971 |
| UC | rs9388489 | 26.51481399 |
| UC | rs1800961 | 25.43430241 |
| UC | rs730497 | 24.17361111 |
| UC | rs1421085 | 72.49375232 |

| | | |
|---|---|---|
| UC | rs13266634 | 64.67040509 |
| UC | rs2383208 | 51.83339471 |
| UC | rs11708067 | 46.60171771 |
| UC | rs115505614 | 41.62012014 |
| UC | rs72802342 | 37.12698404 |
| UC | rs2014830 | 31.99926034 |
| UC | rs12912777 | 31.52083868 |
| UC | rs515071 | 30.72245884 |
| UC | rs1635852 | 30.60674781 |
| UC | rs179252 | 124.0609175 |
| UC | rs2476601 | 93.1268274 |
| UC | rs3094067 | 84.34972299 |
| UC | rs11571297 | 52.87566475 |
| UC | rs2855426 | 44.11338828 |
| UC | rs7516666 | 443.202828 |
| UC | rs9869760 | 373.559354 |
| UC | rs2836836 | 368.6366667 |
| UC | rs12428112 | 72.89417023 |
| UC | rs12421579 | 53.9085254 |
| UC | rs12151689 | 50.28327229 |
| UC | rs6516819 | 46.83519595 |
| UC | rs2001823 | 46.74806385 |
| UC | rs965513 | 46.40110627 |
| UC | rs6060124 | 44.50478237 |
| UC | rs4149570 | 44.15782176 |
| UC | rs869987 | 43.57851929 |
| UC | rs3809983 | 41.5960671 |
| UC | rs10801825 | 41.44580956 |
| UC | rs932311 | 40.65684167 |
| UC | rs9290550 | 39.40310821 |
| UC | rs241449 | 39.16755826 |
| UC | rs2526401 | 38.62141278 |
| UC | rs1868534 | 38.11722773 |
| UC | rs12782349 | 37.66344954 |
| UC | rs3828193 | 36.26520651 |
| UC | rs2516677 | 34.65804705 |
| UC | rs8049383 | 33.47773309 |
| UC | rs9355255 | 32.72510609 |
| UC | rs950910 | 30.34197834 |
| UC | rs406113 | 30.18413893 |

**entary Table 11. Forward direction MR analysis F**

| Exposure | SNP | F_statistic |
|---|---|---|
| IBD | rs7547569 | 774.768 |
| IBD | rs6111031 | 320.323 |
| IBD | rs7711427 | 294.734 |
| IBD | rs10748781 | 282.372 |
| IBD | rs9273363 | 258.696 |
| IBD | rs11793497 | 241.661 |
| IBD | rs10761659 | 234.947 |
| IBD | rs9836291 | 233.634 |
| IBD | rs272882 | 232.792 |
| IBD | rs11236797 | 229.110 |
| IBD | rs4151651 | 228.735 |
| IBD | rs3024493 | 223.391 |
| IBD | rs56167332 | 220.464 |
| IBD | rs2836883 | 212.792 |
| IBD | rs10758669 | 212.135 |
| IBD | rs59655222 | 196.166 |
| IBD | rs4795397 | 192.672 |
| IBD | rs6708373 | 182.434 |
| IBD | rs2143178 | 166.282 |
| IBD | rs10800309 | 161.210 |
| IBD | rs7194886 | 158.398 |
| IBD | rs7608910 | 158.346 |
| IBD | rs7523442 | 158.224 |
| IBD | rs7848647 | 153.383 |
| IBD | rs6062496 | 145.038 |
| IBD | rs17800987 | 136.352 |
| IBD | rs11745587 | 131.557 |
| IBD | rs1297258 | 129.460 |
| IBD | rs8127691 | 128.442 |
| IBD | rs2847278 | 119.455 |
| IBD | rs12318183 | 118.074 |
| IBD | rs34779708 | 108.514 |
| IBD | rs6556417 | 105.170 |
| IBD | rs11743851 | 99.941 |
| IBD | rs11152949 | 96.913 |
| IBD | rs744166 | 96.007 |
| IBD | rs62037363 | 92.612 |
| IBD | rs1388585 | 92.465 |
| IBD | rs3806308 | 91.558 |
| IBD | rs7738430 | 89.940 |
| IBD | rs9889296 | 86.563 |
| IBD | rs74346889 | 86.438 |
| IBD | rs13407913 | 86.126 |
| IBD | rs1420098 | 85.972 |
| IBD | rs395157 | 85.583 |
| IBD | rs35164067 | 85.230 |
| IBD | rs17293632 | 85.191 |
| IBD | rs1250566 | 84.072 |

| | | |
|---|---|---|
| IBD | rs4643314 | 82.546 |
| IBD | rs72634258 | 82.173 |
| IBD | rs2024092 | 77.828 |
| IBD | rs10055349 | 77.816 |
| IBD | rs2488401 | 77.355 |
| IBD | rs9878063 | 76.594 |
| IBD | rs9457247 | 76.265 |
| IBD | rs4676410 | 75.119 |
| IBD | rs2179070 | 73.825 |
| IBD | rs4845604 | 73.141 |
| IBD | rs367569 | 72.218 |
| IBD | rs6561151 | 71.021 |
| IBD | rs1567009 | 70.828 |
| IBD | rs11117431 | 68.955 |
| IBD | rs6588248 | 68.327 |
| IBD | rs1551399 | 67.059 |
| IBD | rs12720356 | 66.173 |
| IBD | rs4845140 | 66.079 |
| IBD | rs740287 | 65.779 |
| IBD | rs1990623 | 64.315 |
| IBD | rs7097656 | 63.965 |
| IBD | rs77059113 | 63.782 |
| IBD | rs11677953 | 62.318 |
| IBD | rs2279990 | 62.210 |
| IBD | rs1003342 | 62.080 |
| IBD | rs780094 | 61.758 |
| IBD | rs181826 | 61.674 |
| IBD | rs1363907 | 61.314 |
| IBD | rs6933404 | 60.955 |
| IBD | rs1419632 | 60.462 |
| IBD | rs2395022 | 60.271 |
| IBD | rs4917129 | 60.001 |
| IBD | rs11641184 | 59.731 |
| IBD | rs17694108 | 59.521 |
| IBD | rs11230563 | 58.845 |
| IBD | rs3776414 | 57.978 |
| IBD | rs12718244 | 57.516 |
| IBD | rs1517352 | 57.236 |
| IBD | rs516246 | 55.095 |
| IBD | rs2328546 | 54.855 |
| IBD | rs7657746 | 54.174 |
| IBD | rs3806157 | 53.032 |
| IBD | rs559928 | 53.006 |
| IBD | rs9557207 | 52.896 |
| IBD | rs35256947 | 52.706 |
| IBD | rs2283789 | 52.229 |
| IBD | rs2393904 | 51.845 |
| IBD | rs941823 | 51.786 |
| IBD | rs974801 | 51.524 |
| IBD | rs1265100 | 51.289 |
| IBD | rs1292053 | 50.866 |

| | | |
|---|---|---|
| IBD | rs2006788 | 50.345 |
| IBD | rs2621323 | 48.875 |
| IBD | rs4976646 | 48.541 |
| IBD | rs12722515 | 47.864 |
| IBD | rs11229555 | 47.601 |
| IBD | rs6740462 | 47.468 |
| IBD | rs259964 | 47.047 |
| IBD | rs2974935 | 46.564 |
| IBD | rs2108225 | 45.854 |
| IBD | rs2153283 | 45.488 |
| IBD | rs55693740 | 45.488 |
| IBD | rs13107612 | 45.384 |
| IBD | rs2050392 | 45.100 |
| IBD | rs2297559 | 45.087 |
| IBD | rs17119 | 44.799 |
| IBD | rs2538470 | 44.177 |
| IBD | rs17780256 | 44.054 |
| IBD | rs12103 | 44.002 |
| IBD | rs11713774 | 43.654 |
| IBD | rs2270395 | 43.111 |
| IBD | rs12585310 | 43.082 |
| IBD | rs913678 | 43.046 |
| IBD | rs4703855 | 42.476 |
| IBD | rs11691685 | 42.445 |
| IBD | rs61959439 | 42.222 |
| IBD | rs6456426 | 42.214 |
| IBD | rs6074022 | 42.180 |
| IBD | rs7240004 | 41.794 |
| IBD | rs2497318 | 41.223 |
| IBD | rs34920465 | 41.044 |
| IBD | rs8055876 | 40.444 |
| IBD | rs4973341 | 40.439 |
| IBD | rs2688608 | 39.841 |
| IBD | rs752508 | 39.757 |
| IBD | rs1990760 | 39.339 |
| IBD | rs2816958 | 39.296 |
| IBD | rs314313 | 39.154 |
| IBD | rs2517689 | 38.546 |
| IBD | rs13204742 | 38.529 |
| IBD | rs470079 | 38.386 |
| IBD | rs67643815 | 38.190 |
| IBD | rs1847472 | 38.127 |
| IBD | rs254562 | 38.110 |
| IBD | rs3853824 | 37.834 |
| IBD | rs6651252 | 37.513 |
| IBD | rs1182188 | 37.176 |
| IBD | rs648541 | 36.941 |
| IBD | rs17668708 | 36.834 |
| IBD | rs3184504 | 36.828 |
| IBD | rs3749946 | 36.592 |
| IBD | rs3801835 | 36.573 |

| | | |
|---|---|---|
| IBD | rs1535 | 35.329 |
| IBD | rs1569328 | 35.052 |
| IBD | rs72978783 | 34.788 |
| IBD | rs4743820 | 34.725 |
| IBD | rs2769267 | 34.575 |
| IBD | rs56163845 | 34.304 |
| IBD | rs2284553 | 34.259 |
| IBD | rs10878386 | 34.001 |
| IBD | rs7773324 | 33.888 |
| IBD | rs12411259 | 33.779 |
| IBD | rs17679361 | 33.777 |
| IBD | rs2274351 | 33.554 |
| IBD | rs34856868 | 32.881 |
| IBD | rs453779 | 32.697 |
| IBD | rs10142466 | 32.685 |
| IBD | rs4692386 | 32.468 |
| IBD | rs12928665 | 32.345 |
| IBD | rs2143606 | 32.211 |
| IBD | rs4767956 | 32.138 |
| IBD | rs72924296 | 32.127 |
| IBD | rs7236492 | 32.113 |
| IBD | rs907611 | 31.939 |
| IBD | rs4802307 | 31.787 |
| IBD | rs7011507 | 31.464 |
| IBD | rs1528602 | 31.317 |
| IBD | rs4664304 | 30.977 |
| IBD | rs6058869 | 30.959 |
| IBD | rs2945412 | 30.862 |
| IBD | rs17651741 | 30.835 |
| IBD | rs7015630 | 30.775 |
| IBD | rs7404095 | 30.644 |
| IBD | rs12599586 | 30.368 |
| IBD | rs34804116 | 30.341 |
| IBD | rs11190087 | 30.051 |
| IBD | rs113010081 | 30.050 |
| IBD | rs12796489 | 309.453 |
| IBD | rs55808324 | 70.284 |
| IBD | rs6025 | 31.051 |
| CD | rs7517847 | 724.541 |
| CD | rs7711427 | 395.535 |
| CD | rs6738490 | 349.989 |
| CD | rs7194886 | 347.591 |
| CD | rs17622378 | 247.976 |
| CD | rs6111031 | 242.807 |
| CD | rs12766391 | 233.639 |
| CD | rs10748781 | 228.071 |
| CD | rs11236797 | 224.699 |
| CD | rs1896707 | 199.098 |
| CD | rs11793497 | 192.341 |
| CD | rs56167332 | 181.491 |
| CD | rs1990623 | 166.402 |

| | | |
|---|---|---|
| CD | rs2413583 | 156.184 |
| CD | rs10758669 | 148.249 |
| CD | rs3197999 | 145.090 |
| CD | rs9264942 | 138.144 |
| CD | rs11743851 | 136.708 |
| CD | rs4795397 | 120.992 |
| CD | rs10055349 | 120.582 |
| CD | rs7848647 | 118.225 |
| CD | rs12131796 | 118.023 |
| CD | rs34779708 | 117.821 |
| CD | rs76906269 | 113.419 |
| CD | rs1250573 | 111.019 |
| CD | rs2847278 | 110.825 |
| CD | rs4643314 | 110.114 |
| CD | rs1297258 | 108.484 |
| CD | rs11152949 | 108.415 |
| CD | rs9889296 | 107.812 |
| CD | rs3024505 | 107.239 |
| CD | rs6561151 | 106.903 |
| CD | rs2024092 | 106.069 |
| CD | rs8127691 | 102.427 |
| CD | rs9457247 | 99.385 |
| CD | rs7608910 | 98.693 |
| CD | rs26528 | 95.765 |
| CD | rs12411259 | 95.569 |
| CD | rs13001325 | 95.251 |
| CD | rs6062496 | 93.624 |
| CD | rs780094 | 93.272 |
| CD | rs13407913 | 91.791 |
| CD | rs921720 | 91.489 |
| CD | rs516246 | 86.601 |
| CD | rs35164067 | 84.869 |
| CD | rs438475 | 84.733 |
| CD | rs17293632 | 84.575 |
| CD | rs2945412 | 84.185 |
| CD | rs11117431 | 82.437 |
| CD | rs6556417 | 81.193 |
| CD | rs12041056 | 80.126 |
| CD | rs1946410 | 77.070 |
| CD | rs4655709 | 76.328 |
| CD | rs7097656 | 75.378 |
| CD | rs7775228 | 73.904 |
| CD | rs2270395 | 73.737 |
| CD | rs1567009 | 73.669 |
| CD | rs1528602 | 72.444 |
| CD | rs12949918 | 71.056 |
| CD | rs9491892 | 70.878 |
| CD | rs2476601 | 70.862 |
| CD | rs2284553 | 70.104 |
| CD | rs1646019 | 69.262 |
| CD | rs4151651 | 69.012 |

| | | |
|---|---|---|
| CD | rs925255 | 68.832 |
| CD | rs6456426 | 68.353 |
| CD | rs212388 | 67.816 |
| CD | rs2488401 | 67.530 |
| CD | rs77981966 | 67.421 |
| CD | rs395157 | 67.277 |
| CD | rs34787213 | 66.901 |
| CD | rs6651252 | 66.307 |
| CD | rs1363907 | 66.291 |
| CD | rs181826 | 61.453 |
| CD | rs2836878 | 61.421 |
| CD | rs2058813 | 60.770 |
| CD | rs752508 | 60.347 |
| CD | rs13109404 | 60.203 |
| CD | rs11713774 | 59.729 |
| CD | rs1292053 | 58.798 |
| CD | rs4917129 | 58.452 |
| CD | rs68191 | 56.581 |
| CD | rs3801810 | 56.174 |
| CD | rs11159833 | 55.908 |
| CD | rs34804116 | 54.895 |
| CD | rs9257828 | 54.441 |
| CD | rs61839660 | 53.086 |
| CD | rs714027 | 52.899 |
| CD | rs102275 | 52.654 |
| CD | rs2227551 | 52.316 |
| CD | rs2283789 | 52.191 |
| CD | rs3776414 | 52.188 |
| CD | rs10800309 | 51.168 |
| CD | rs6740462 | 49.761 |
| CD | rs2153283 | 49.131 |
| CD | rs6074022 | 48.898 |
| CD | rs6908425 | 47.764 |
| CD | rs72727394 | 47.580 |
| CD | rs4802307 | 46.526 |
| CD | rs56163845 | 46.450 |
| CD | rs2006788 | 46.022 |
| CD | rs28999107 | 45.825 |
| CD | rs11691685 | 45.738 |
| CD | rs12107418 | 45.258 |
| CD | rs7195296 | 44.486 |
| CD | rs2230365 | 44.169 |
| CD | rs12720356 | 43.940 |
| CD | rs7438704 | 43.920 |
| CD | rs11679753 | 43.354 |
| CD | rs12718244 | 43.333 |
| CD | rs11175963 | 43.245 |
| CD | rs2188958 | 42.941 |
| CD | rs1569328 | 42.672 |
| CD | rs9554587 | 42.188 |
| CD | rs41295117 | 42.050 |

| | | |
|---|---|---|
| CD | rs6738394 | 42.033 |
| CD | rs1847472 | 41.653 |
| CD | rs76181804 | 41.585 |
| CD | rs3853824 | 41.509 |
| CD | rs1517352 | 41.288 |
| CD | rs11264426 | 41.030 |
| CD | rs61959448 | 41.029 |
| CD | rs2621323 | 40.948 |
| CD | rs36016881 | 40.899 |
| CD | rs727563 | 40.585 |
| CD | rs6738825 | 39.765 |
| CD | rs2395022 | 39.589 |
| CD | rs6827756 | 39.506 |
| CD | rs559928 | 39.241 |
| CD | rs9494844 | 39.027 |
| CD | rs4767956 | 38.978 |
| CD | rs367254 | 38.391 |
| CD | rs71624119 | 38.143 |
| CD | rs10276381 | 38.091 |
| CD | rs2735076 | 37.983 |
| CD | rs2143606 | 37.673 |
| CD | rs303429 | 37.671 |
| CD | rs7015630 | 37.530 |
| CD | rs2641348 | 37.394 |
| CD | rs35320439 | 37.346 |
| CD | rs7969592 | 37.256 |
| CD | rs2538470 | 37.237 |
| CD | rs7773324 | 37.209 |
| CD | rs640466 | 36.803 |
| CD | rs259964 | 35.895 |
| CD | rs1990684 | 35.561 |
| CD | rs17391694 | 35.449 |
| CD | rs3130186 | 35.063 |
| CD | rs17694108 | 35.004 |
| CD | rs10798069 | 34.507 |
| CD | rs76606004 | 33.904 |
| CD | rs7758080 | 33.460 |
| CD | rs7236492 | 33.026 |
| CD | rs1267501 | 32.902 |
| CD | rs76546301 | 32.882 |
| CD | rs974801 | 32.379 |
| CD | rs16870166 | 32.239 |
| CD | rs3184504 | 31.799 |
| CD | rs568617 | 31.755 |
| CD | rs224140 | 31.314 |
| CD | rs1486418 | 31.290 |
| CD | rs915286 | 30.995 |
| CD | rs13204048 | 30.779 |
| CD | rs314313 | 30.776 |
| CD | rs3812609 | 30.737 |
| CD | rs4703855 | 30.688 |

| | | |
|---|---|---|
| CD | rs4976646 | 30.428 |
| CD | rs12194548 | 29.876 |
| CD | rs12796489 | 225.783 |
| CD | rs16917597 | 32.477 |
| UC | rs9271255 | 425.836 |
| UC | rs6426833 | 341.048 |
| UC | rs7547569 | 288.883 |
| UC | rs2836883 | 237.370 |
| UC | rs3024493 | 191.603 |
| UC | rs6111031 | 187.151 |
| UC | rs1801274 | 182.422 |
| UC | rs3806308 | 170.568 |
| UC | rs10748783 | 169.913 |
| UC | rs9836291 | 165.217 |
| UC | rs12318183 | 164.093 |
| UC | rs59655222 | 134.716 |
| UC | rs3095301 | 128.384 |
| UC | rs4795397 | 123.648 |
| UC | rs10758669 | 123.587 |
| UC | rs7738430 | 116.602 |
| UC | rs56167332 | 115.156 |
| UC | rs11793497 | 113.426 |
| UC | rs272882 | 110.764 |
| UC | rs4380874 | 106.273 |
| UC | rs7608910 | 100.392 |
| UC | rs9272105 | 100.094 |
| UC | rs61893460 | 93.253 |
| UC | rs6920220 | 93.178 |
| UC | rs10761659 | 86.363 |
| UC | rs661054 | 84.873 |
| UC | rs2108225 | 82.636 |
| UC | rs4676410 | 81.388 |
| UC | rs10799838 | 80.528 |
| UC | rs4366152 | 78.551 |
| UC | rs6062496 | 78.237 |
| UC | rs2816958 | 77.806 |
| UC | rs4845604 | 77.707 |
| UC | rs1431403 | 71.835 |
| UC | rs34659678 | 69.993 |
| UC | rs1359946 | 69.042 |
| UC | rs4812833 | 67.735 |
| UC | rs7282490 | 67.549 |
| UC | rs35223180 | 64.355 |
| UC | rs1297256 | 62.973 |
| UC | rs9611131 | 61.779 |
| UC | rs1182188 | 61.249 |
| UC | rs2394250 | 61.235 |
| UC | rs10185424 | 59.133 |
| UC | rs4728142 | 58.614 |
| UC | rs9941524 | 58.387 |
| UC | rs3774937 | 56.890 |

| | | |
|---|---|---|
| UC | rs7633840 | 54.942 |
| UC | rs941823 | 54.718 |
| UC | rs34920465 | 53.893 |
| UC | rs8096327 | 53.780 |
| UC | rs17780256 | 51.805 |
| UC | rs4845140 | 51.767 |
| UC | rs7711427 | 48.291 |
| UC | rs17694108 | 47.275 |
| UC | rs395157 | 46.586 |
| UC | rs10910092 | 45.638 |
| UC | rs12720356 | 45.321 |
| UC | rs9891119 | 45.262 |
| UC | rs10010325 | 44.806 |
| UC | rs6556417 | 44.179 |
| UC | rs2070729 | 43.346 |
| UC | rs9368696 | 42.107 |
| UC | rs76546301 | 41.728 |
| UC | rs1990760 | 40.693 |
| UC | rs13136827 | 40.155 |
| UC | rs7240004 | 40.032 |
| UC | rs254562 | 39.799 |
| UC | rs2395022 | 39.755 |
| UC | rs483905 | 39.574 |
| UC | rs11150589 | 39.498 |
| UC | rs11641184 | 38.999 |
| UC | rs1405108 | 38.369 |
| UC | rs12142199 | 38.100 |
| UC | rs17679361 | 38.077 |
| UC | rs113010081 | 37.527 |
| UC | rs2488401 | 37.108 |
| UC | rs2240064 | 36.052 |
| UC | rs11676348 | 35.900 |
| UC | rs1517352 | 35.879 |
| UC | rs4946717 | 35.658 |
| UC | rs11578752 | 35.631 |
| UC | rs4976646 | 35.526 |
| UC | rs76904798 | 35.332 |
| UC | rs6088747 | 34.715 |
| UC | rs4743820 | 34.601 |
| UC | rs17552904 | 34.504 |
| UC | rs1076137 | 34.119 |
| UC | rs1077773 | 33.846 |
| UC | rs9911533 | 33.444 |
| UC | rs17800987 | 33.278 |
| UC | rs4747886 | 32.924 |
| UC | rs907611 | 32.838 |
| UC | rs151723 | 32.683 |
| UC | rs2497318 | 32.576 |
| UC | rs11229555 | 32.463 |
| UC | rs913678 | 32.440 |
| UC | rs13430791 | 32.195 |

| | | |
|---|---|---|
| UC | rs12718244 | 32.171 |
| UC | rs79045992 | 32.142 |
| UC | rs59418206 | 32.119 |
| UC | rs79823250 | 32.027 |
| UC | rs7404095 | 32.022 |
| UC | rs10460566 | 31.932 |
| UC | rs10494343 | 31.833 |
| UC | rs11230563 | 31.591 |
| UC | rs4712520 | 31.302 |
| UC | rs4973341 | 31.263 |
| UC | rs6700241 | 31.221 |
| UC | rs12661782 | 31.070 |
| UC | rs1646019 | 30.974 |
| UC | rs11083840 | 30.459 |
| UC | rs16939788 | 30.429 |
| UC | rs13255292 | 30.241 |
| UC | rs3776414 | 30.100 |
| UC | rs17736589 | 29.991 |
| UC | rs17771967 | 29.921 |
| UC | rs2274351 | 29.758 |
| UC | rs12796489 | 146.127 |
| UC | rs411326 | 114.410 |
| UC | rs55808324 | 36.572 |
